# Being more satisfied with romantic relationship status is associated with increased mental wellbeing in people with experience of psychosis

**DOI:** 10.3389/fpsyt.2023.1232973

**Published:** 2023-09-28

**Authors:** Rebecca White, Gillian Haddock, Maria Haarmans, Filippo Varese

**Affiliations:** ^1^Division of Psychology and Mental Health, School of Health Sciences, Faculty of Biology, Medicine and Health, Manchester Academic Health Science Centre, The University of Manchester, Manchester, United Kingdom; ^2^Greater Manchester Mental Health NHS Foundation Trust, Manchester Academic Health Science Centre, The University of Manchester, Manchester, United Kingdom; ^3^Department of Sociology, School of Social Sciences, Faculty of Humanities, The University of Manchester, Manchester, United Kingdom; ^4^School of Psychology, University of Liverpool, Liverpool, United Kingdom

**Keywords:** psychosis, romantic relationships, mental wellbeing, self-esteem, loneliness, internalised stigma, attachment

## Abstract

**Aims:**

Romantic relationships represent one of the most salient sources of social support. In general population studies, they are associated with both physical and psychological benefits. Research suggests that for people with psychosis, romantic relationships may also have a positive impact on a range of outcomes, but the reasons for these associations are still unclear. This study aims to investigate whether satisfaction with romantic relationships status is associated with better wellbeing outcomes in people with experience of psychosis and explore three possible psychological mediators of this relationship.

**Methods:**

Participants who had previously sought support for psychosis (*n* = 190) completed an online survey including measures of relationship status satisfaction (the Satisfaction with Relationship Scale) as well as measures of psychotic symptoms (the CAPE-42), general well-being (Short Warwick-Edinburgh Mental Wellbeing Scale) and several psychological variables relevant to the pathway between romantic relationships and well-being outcomes, namely loneliness, internalised stigma, self-esteem and attachment.

**Results:**

Fearful attachment and partner criticism were negatively associated with relationship status satisfaction. Having a partner was positively associated with relationship status satisfaction. Higher levels of relationship status satisfaction were associated with lower psychotic symptoms and higher mental wellbeing. This relationship was mediated by loneliness, internalised stigma, and self-esteem.

**Conclusion:**

Mental health services should be mindful of the associations between romantic relationship satisfaction and wellbeing. Service users with a fearful attachment style may particularly benefit from support in this area.

## 1. Introduction

Within the general population, research suggests romantic relationships are associated with physical and psychological benefits. Being in a relationship has been associated with higher levels of wellbeing compared to being single (1). The link between relationship status and wellbeing has been shown to be partially mediated by employment/education status, self-esteem, neuroticism and social support. It is also thought that relationship quality is plays an important role in wellbeing for those in romantic relationships (1). Indeed, high quality marriages (defined by high satisfaction and low levels of conflict) have been linked to better physical health outcomes (2). Furthermore, symptoms of severe mental health difficulties, including the experience of psychosis, have been found to be more prevalent amongst those who have never been married or are separated, compared to those are married (3).

Within clinical populations, whilst it is generally accepted that supportive social relationships aid recovery from mental health difficulties (4–6), less is known about the role of romantic/intimate relationships. Existing research suggests that romantic relationships may increase confidence, facilitate recovery and be seen as an observable sign of recovery by others (7, 8). There is also evidence to suggest that for psychiatric inpatients, *not* having a partner is associated with lower self-esteem (9) and higher levels of internalised stigma (10, 11). Furthermore, feeling unworthy of love has been associated with internalised stigma dimensions (12) and higher levels of internalised stigma have been linked to increased symptom severity in those with a mental health diagnosis (13). It is also possible that having a romantic partner may increase social support and reduce loneliness, which may, in turn, reduce symptomatology (14, 15).

However, not all relationships are beneficial to wellbeing—some may even be detrimental. The effect a romantic relationship has on an individual’s wellbeing is likely to depend on the quality of the relationship. For example, although much of the expressed emotion (EE) literature involves family dyads, given the robust findings whereby more critical comments from relatives are associated with increased relapse rates (16, 17), it is likely that partners who are highly critical will have a similar impact on the mental health of people who experience psychosis. Similarly, attachment style is also likely to influence satisfaction and perceptions of support within a relationship. Those who are uncomfortable relying on others and value independence over close relationships (18) may prefer not to be involved in romantic relationships. Additionally, individuals who have an avoidant attachment style may not benefit from relationships in the same way as those who are securely attached. Those with avoidant attachment styles may be more likely to use indirect methods to seek support and seek less support, regardless of their level of distress (19). This can be problematic as indirect requests for support have been shown to result in less helpful responses, compared to direct requests. Additionally, those seeking support perceive their relationships to be of higher quality when their partner is a more effective caregiver (19). Attachment is particularly important to consider for people with experience of psychosis as insecure attachment styles are more common in this group than in non-clinical samples, with a fearful attachment style (i.e., high anxiety, high avoidance) being most prevalent (20).

Currently there is very limited research which has explored the association between romantic relationships and mental wellbeing for people with experience of psychosis. The mechanisms via which such relationships might influence wellbeing are also unknown (21). Additionally, most existing studies only consider the role of relationship status. Other potentially influential variables such as relationship quality and satisfaction, have been neglected (22). Therefore, in the current cross-sectional study we examined the association between relationship status, fearful attachment, partner criticism and satisfaction with relationship status. Satisfaction with current romantic relationship status was the primary measure of romantic relationships. In acknowledgement that some people may prefer to be single and to ensure that findings were relevant to those without a partner, people who were single, as well as those in a relationship, were of interest in this study. This was seen as especially important given existing evidence which suggests people with experience of psychosis are less likely to have a romantic partner than the general population (21). The primary aim was to understand whether satisfaction with relationship status (regardless of whether an individual is single or in a relationship) was associated with better mental wellbeing in people who experience psychosis. Wellbeing outcomes considered were general mental wellbeing and symptoms of psychosis. The role of self-esteem, loneliness, and internalised stigma as possible mediators between satisfaction with romantic relationship status and wellbeing was also considered. The following hypotheses were tested:

*H1*: Being in a relationship will be positively associated with satisfaction with current relationship status and fearful attachment will be negatively associated with satisfaction with current relationship status.

*H2*: For those who are in a relationship, higher levels of fearful attachment and criticism from a partner will be associated with lower scores for satisfaction with relationship status.

*H3*: Greater satisfaction with current romantic relationship status will be associated with higher wellbeing (i.e., higher mental wellbeing scores and lower scores for psychotic experiences).

*H4*: The relationship between romantic relationship satisfaction and wellbeing will be mediated by self-esteem, loneliness, and internalised stigma.

## 2. Materials and methods

### 2.1. Participants

The target population was people with lived experience of psychosis, who self-reported ever having sought/received support for these experiences. Participants were eligible to take part if they were aged at least 16 years old and endorsed at least one of the following: had ever received a schizophrenia spectrum diagnosis or had their mental health difficulties described as psychosis by a mental health professional; ever sought or received mental health support from a professional organisation or service for the experience of psychosis; spent time in a psychiatric inpatient ward for the experience of psychosis; ever been prescribed or taken antipsychotic medication for the experience of psychosis. These criteria were assessed via a series of screening questions, completed by potential participants prior to the study.

Prior to recruitment a power analysis using the Monte Carlo Power Analysis for Indirect Effects application (23) and correlations from existing literature (24–26) indicated a sample of 152 would enable the reliable detection of significant effects in a simple one mediator model at the recommended power of 0.80. Additionally, 150 participants provides adequate power to detect medium effects in regression models with up to 10 predictor variables (27).

### 2.2. Measures

#### 2.2.1. Demographic information

A short questionnaire elicited participants’ age, ethnicity, gender, sexual orientation, current relationship status and history, employment status and whether they were currently receiving professional support for the experience of psychosis. Regarding current relationship status, participants were asked to select one of the following: single; dating/seeing someone but not ‘officially’ in a relationship; in a relationship, not living together; in a relationship living together, married/civil partnership, living together; married/civil partnership, not living together, separated but still legally married or widowed. Participants were also given the option to self-describe their relationship status if they preferred.

#### 2.2.2. The satisfaction with relationship scale

The satisfaction with relationship scale (ReSta) is a five item self-report measure of relationship status satisfaction [(28)-adapted]. Participants are asked to rate their level of agreement with statements on a four-point scale from (0 = ‘not at all’, 3 = ‘to a great extent’). Scores are added together with higher scores indicating higher satisfaction with current relationship status. ReSta has been shown to have good validity and reliability in a general population sample (28). Following consultation with the author of the scale and people with lived experience of psychosis, items of the scale were tailored according to whether participants indicated they were single or had a partner when answering the demographic information questions. For example, *‘In general, how satisfied are you with your current status’* from the original scale became ‘*In general, how satisfied are you with being single’* or ‘*In general, how satisfied are you with being in a relationship’,* depending on whether the participant identified as single or in a relationship based on their responses on the demographic questionnaire. Participants were presented with both versions of the scale and given instructions on which to complete depending on their relationship status. Internal consistency for the ReSta single and partner scales in this study were excellent (α = 0.91 and α = 0.92, respectively).

#### 2.2.3. Perceived criticism

Participants who indicated they were in a relationship also completed a single item: *‘How critical do you think your partner is of you?’* (29). Participants rated perceived criticism on a 10-point Likert scale (0 = ‘not at all critical’, 10 = ‘very critical’). The single item measure of Perceived criticism (PC) has been shown to be correlated with spouses’ overall expressed emotion scores as measured by the Camberwell Family Interview (r = 0.51) and has been used extensively in a variety of clinical populations (29).

#### 2.2.4. Relationships questionnaire

Participants were asked to read four short paragraphs describing four attachment styles [secure, dismissing, preoccupied and fearful; (30)]. Participants indicated which one described them best and also rated how representative each paragraph was of them using a seven-point Likert (1 = ‘not at all like me’, 7 = ‘very much like me’). The Relationships questionnaire (RQ) has been widely used in psychosis research (31) and the format it is suitable for online studies (32).

#### 2.2.5. The short Warwick-Edinburgh mental wellbeing scale

The short Warwick-Edinburgh mental wellbeing scale (SWEMWBS) aims to measure different aspects of positive mental health (e.g., *‘I’ve been feeling relaxed’, ‘I’ve been dealing with problems well’* and *‘I’ve been feeling optimistic about the future’*) (33). The scale consists of 7 positively worded items. Participants indicated on a five-point Likert scale how often each statement has applied to them over the past 2 weeks (1 = ‘none of the time’, 5 = ‘all of the time’). Higher scores on the scale represent a higher level of mental well-being. SWEMWBS has been validated in a UK adult population (33) and previously used with clinical populations (34). The SWEMWBS showed excellent internal consistency in this study (α = 0.90).

#### 2.2.6. The community assessment of psychic experiences

The community assessment of psychic experiences (CAPE) was used to measure the frequency of positive, negative, and depressive symptoms associated with psychosis (35). The scale consists of 42 self-report items such as *‘Do you ever feel as if some people are not what they seem to be?’* and *‘Do you ever feel that you experience few or no emotions at important events?’* Respondents indicated the frequency at which they experience each item (1 = ‘never’, 4 = ‘nearly always’). The CAPE has been shown to have good reliability and validity when used with general population samples (36) and in clinical populations (37, 38). All subscales of the CAPE showed good internal consistency in this study: positive subscale (α = 0.93), depressive subscale (α = 0.90), negative subscale (α = 0.90).

#### 2.2.7. Mediator variables

##### 2.2.7.1. Self-esteem rating scale—short form

The Self-esteem rating scale—short form (SERS-SF) is a 20-item measure of explicit self-esteem (39). The measure contains items which assess positive (e.g., *‘I feel that I make a good impression on* others’) and negative (e.g., *‘I feel inferior to other people’*) beliefs about the self. Participants are asked to rate how often each of the statements reflects their feelings on a seven-point Likert scale (1 = ‘never’, 7 = ‘always’). The SERS-SF has been shown to be valid and reliable for use with clinical populations (39). Additionally, the internal consistency of the SERS-SF in this study was excellent (α = 0.95).

##### 2.2.7.2. Loneliness scale

Participants completed Hughes et al.’s three item loneliness scale which aims to measure feelings of social isolation via items such as: ‘*How often do you feel that you lack companionship?’* (40). Items are rated on a three-point scale (1 = ‘hardly ever’, 3 = ‘often’). Scores are totalled with higher scores indicating higher levels of loneliness. The scale has been shown to have good reliability and validity in the general population (40) and has also been used with clinical populations (15, 41). The internal consistency of the Loneliness scale (LS) in this study was good (α = 0.81).

##### 2.2.7.3. Internalised stigma of mental illness inventory—10-item version

Internalised stigma of mental illness inventory—10-item version (ISMI-10) measures internalised stigma by assessing alienation, discrimination experience, social withdrawal, stereotype endorsement and stigma resistance (42). Items include: *‘I do not socialise as much as I used to because my mental illness might make me look or behave ‘weird’* and *‘I cannot contribute anything to society because I have a mental illness’.* Respondents rate their agreement with each item on a four-point scale (1 = ‘strongly disagree’, 4 = ‘strongly agree’). Scores are added together and dived by the total number of items answered with high scores represented more severe internalised stigma. The ISMI-10 has been shown to have good psychometric properties (42) and has been used in clinical populations (43, 44). The internal consistency of the ISMI-10 in this study was good (α = 0.83). Following consultation with people who had lived experience of psychosis, a note was added prior to this measure in recognition that not all those who experience psychosis consider themselves ‘mentally ill’.

### 2.3. Procedure

Participants were recruited online via adverts on social media (e.g., Twitter, Facebook), relevant websites/organisations (e.g., MQ participate, National Paranoia Network), participating healthcare organisations and by contacting people who had previously participated in research and had consented to hear about future studies. Recruitment took place from January to December 2020. Participants had the option to complete the questionnaires online or on paper. Measures were ordered so that the outcome and independent variables, which were most important to the analysis, were completed first. Measures were completed in the following order: screening questions, demographic information, CAPE, SWEMWBS, ReSta, PC, LS, ISMI-10, SERS-SF and finally the RQ.

The protocol for this study was independently reviewed by experts in the field, external to the project. The study was given ethical approval by North West – Preston Research Ethics Committee (ref:19/NW/0665). Online study data were collected and managed using REDCap (Research Electronic Data Capture) tools hosted at the University of Manchester (45, 46). In line with Health Research Authority guidance for self-completion surveys, a proportionate approach to consent was taken (47). A participant information sheet was presented, and completion/return of the survey was taken as an indication of consent. As a token of appreciation and following consultation with people with lived experience of psychosis, participants were offered entry into a prize draw to win one of six £50 shopping vouchers at the end of the study.

### 2.4. Statistical analysis

Data cleaning and analysis was conducted using R version 4.0.3 (48). Participants who did not pass the screening questions were removed as were participants who were missing more than 55% of data. For analysis, demographic variables relationship status, gender, ethnicity, sexuality, and employment were dichotomised. Regarding relationship status, those who did not report being in a relationship, i.e., participants who endorsed either being single, dating (but not in a relationship) or separated were categorised as not currently having a partner. All other responses were categorised as having a partner. Where participants chose to self-describe, the description given was reviewed by the first author and they were allocated to the group thought to best fit the description given. Gender was dichotomised as male or female. Unfortunately, there were not enough participants who identified their gender as being outside this dichotomy to conduct meaningful analysis which included these respondents. Ethnicity was dichotomised as white and people of the global majority (PGM) i.e., Black, Asian and other people of colour, including participants with mixed ethnicity. Sexuality was dichotomised as heterosexual and LGBQ+ (lesbian, gay, bisexual, queer plus all other sexualities). Finally, employment was dichotomised as working/full-time education and unemployed. The working/full-time education group included participants who were employees, self-employed, in full-time education or looking after home/family. Those who are unemployed, in receipt of sickness or disability benefits or retired were classed as unemployed. Composite variables for the measures were also created. Missing data were replaced with mean score values when creating composite variables for the ReSta, SWEMWBS and SERS-SF. Raw data scores from the SWEMWBS were then converted to metric scores using the SWEMWBS conversion table (33). For the three-item loneliness scale, four participants with missing data were removed from the analysis. For the CAPE subscales and ISMI-10 a mean score of completed items was calculated.

Bivariate associations between the variables were tested using correlational analysis. To test the first hypothesis, hierarchical regression was used to establish whether relationship status was associated with satisfaction with relationship status. In the first step of the model, relationship status was entered as a predictor. In the second step of the model fearful attachment was added and in the final stage of the model socio-demographic variables: gender, age, ethnicity, sexuality, and employment were added. To test the second hypothesis, a second regression model was built using only the data from those who had a partner. Fearful attachment and perceived criticism were entered in the first two steps followed by socio-demographic covariates. To test the third hypothesis, further hierarchical regression models were created to assess whether satisfaction with relationship status predicted mental wellbeing and CAPE scores. In step one, ReSta score was included as a predictor and in step two socio-demographic variables were added. For all the above correlational and regression analyses, a value of p of below 0.05 was considered significant.

**Figure 1:**
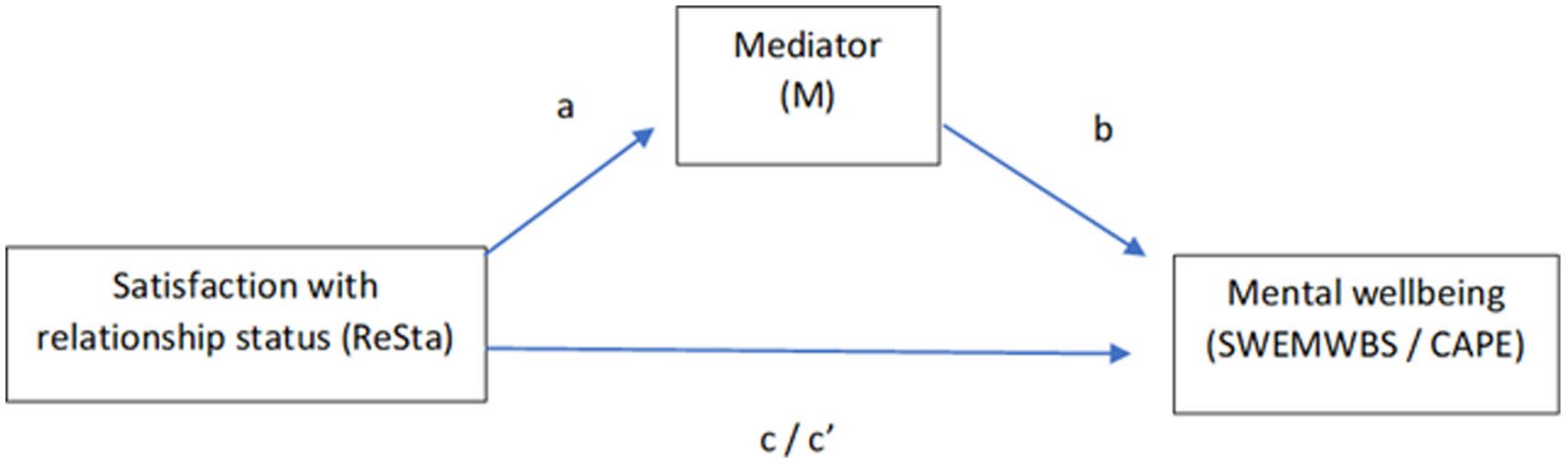
Single mediation models tested. Self-esteem (SERS-SF), loneliness (LS) and internalised stigma (ISMI-10) were tested as possible mediators.

To test the fourth hypothesis, a series of single mediations (Figure 1) were estimated to examine the indirect effect of satisfaction with relationship status on mental wellbeing and CAPE scores via self-esteem, loneliness, and internalised stigma. Analyses were conducted using the Mediation package for R which can accommodate parametric and non-parametric models as well as dichotomous variables. The significance of the indirect effects was tested using bootstrapped confidence intervals (CIs) of 5,000 bootstrap draws. As 12 models were tested, a Bonferroni correction was applied meaning only values < 0.004 were considered significant. For all analyses, tests to confirm assumptions of multicollinearity, linearity, normality, homogeneity, and homoscedasticity were performed. Participants with missing data and outliers were removed. Simple models were tested first before adding covariates. R Markdown documents for all the analyses conducted can be found in the Supplementary material.

## 3. Results

In total, 276 people started the survey having read the participant information, and 232 completed and were identified as eligible to participate by the subsequent screening questionnaires. Overall, 190 (68.8%) provided responses to at least 55% of the items and were included in the analysis. From the data available, non-responders did not differ significantly from those included in the analysis in terms of age *t*(16.41) = −1.25 *p* = 0.23 or gender χ^2^(1) = 0.06, *p* = 0.81. However non-responders differed significantly from participants in being more likely to be in a romantic relationship. 86% of non-participants (n = 12) had a partner compared to 44% of participants (*n* = 79), χ^2^(1) = 9.01, *p* < 0.01. Participants’ ages ranged from 16 to 66 years (*M* = 37.51, *SD* = 12.81). Of 152 participants who indicated which attachment style described them best, 28 (18%) selected secure, 73 (48%) selected fearful, 27 (18%) selected preoccupied and 24 (16%) selected dismissive. Further information for the sample is presented in Table 1 and descriptive statistics for the measures are presented in Table 2.

**Table 1:** Demographic information.

		*n*	%
Gender	Female	105	55.3
	Male	69	36.3
	Non-binary	4	2.1
	Intersex, Trans Genderfae	1	0.5
	‘Every sex’	1	0.5
	Missing	10	5.3
Ethnicity	White—British	148	77.9
	People of the global majority (PGM)	31	16.3
	Missing	11	5.8
Sexuality	Heterosexual	132	69.5
	Bisexual	24	12.6
	Gay/Lesbian	9	4.7
	Pansexual	3	1.6
	Queer	3	1.6
	Bi-romantic asexual	1	0.5
	Quoisexual, Aromantic, Lesbian	1	0.5
	‘Anything’	1	0.5
	Prefer not to say/missing	16	8.4
Relationship status	Single	89	46.8
	Dating, not ‘officially’ in a relationship	5	2.6
	In a relationship, not living together	27	14.2
	In a relationship, living together	21	11.1
	Married, living together	30	15.8
	Separated, still legally married	2	1.1
	Prefer to self-describe	5	2.6
	Missing	11	5.8
Employment	Employee	56	29.5
	Self-employed	5	2.6
	Unemployed	26	13.7
	Full time education	21	11.1
	Looking after home/family	6	3.2
	In receipt of sickness/disability benefits	58	30.5
	Retired	5	2.6
	Missing	13	6.8
Current inpatient	Yes	3	1.6
	No	173	91.1
	Missing	14	7.4
Currently receiving support from MH services	Yes	141	74.2
	No	32	16.8
	Missing	17	8.9

**Table 2:** Descriptive statistics.

	*n*	*M (SD)*	*Med.*	Min.	Max.	Skew.	Kurt.
Relationship status satisfaction (ReSta)	190	9.73 (4.69)	10.50	0.00	15.00	−0.58	−0.92
Mental wellbeing (SWEMWBS)	189	18.94 (4.68)	17.98	7.00	35.00	0.34	0.68
Positive symptoms (CAPE)	190	2.00 (0.64)	1.92	1.00	3.85	0.60	−0.26
Negative symptoms (CAPE)	190	2.34 (0.62)	2.29	1.00	4.00	0.33	−0.21
Depressive symptoms (CAPE)	190	2.53 (0.74)	2.50	1.00	4.00	0.15	−1.00
Loneliness (LS)	183	6.53 (1.91)	6.00	3.00	9.00	−0.30	−1.04
Internalised stigma (ISMI-10)	187	2.38 (0.57)	2.40	1.10	3.70	−0.10	−0.53
Secure attachment (RQ)	172	3.64 (2.06)	4.00	1.00	7.00	0.13	−1.29
Fearful attachment (RQ)	171	4.70 (1.99)	5.00	1.00	7.00	−0.55	−0.89
Preoccupied attachment (RQ)	170	3.86 (1.96)	4.00	1.00	7.00	0.02	−1.09
Dismissive attachment (RQ)	168	3.55 (1.95)	4.00	1.00	7.00	0.12	−1.15

ReSta, Satisfaction with Relationship Scale; SWEMWBS, Short Warwick Edinburgh Mental Wellbeing Scale; CAPE, Community Assessment of Psychic Experiences; LS, Loneliness Scale; ISMI-10, Internalised Stigma of Mental Illness Inventory—10 item version; SERS-SF, Self Esteem Rating Scale—Short Form; RQ, Relationship Questionnaire.

Table 3 shows the results of correlation analyses conducted between the variables. Satisfaction with romantic relationship status was found to be significantly, positively associated with mental wellbeing and self-esteem. Similarly, satisfaction with relationship status was found to be significantly, negatively associated with the CAPE depressive subscale, loneliness, internalised stigma, and fearful attachment.

**Table 3:** Correlation matrix.

		1	2	3	4	5	6	7	8	9	10	11
1.	Relationship status satisfaction (ReSta)											
2.	Mental wellbeing (SWEMWBS)	0.28^***^										
3.	Positive symptoms (CAPE)	−0.05	−0.49^***^									
4.	Negative symptoms (CAPE)	−0.10	−0.64^***^	0.59^***^								
5.	Depressive symptoms (CAPE)	−0.18^**^	−0.75^***^	0.66^***^	0.71^***^							
6.	Loneliness (LS)	−0.57^***^	−0.54^***^	0.36^***^	0.42 ^***^	0.55 ^***^						
7.	Internalised stigma (ISMI-10)	−0.20^**^	−0.60^***^	0.50^***^	0.56^***^	0.57^***^	0.46^***^					
8.	Self-esteem (SERS-SF)	0.24^**^	0.79^***^	−0.57^***^	−0.62^***^	−0.76^***^	−0.59^***^	−0.68^***^				
9.	Secure attachment (RQ)	0.13	0.41^***^	−0.35^***^	−0.41^***^	−0.39^***^	−0.33^***^	−0.44^***^	0.51^***^			
10.	Fearful attachment (RQ)	−0.17^*^	−0.41^***^	0.33^***^	0.26^***^	0.45^***^	0.44^***^	0.35^***^	−0.55^***^	−0.38^***^		
11.	Preoccupied attachment (RQ)	−0.15^*^	−0.06	0.09	0.12	0.20 ^**^	0.26^***^	0.15 ^*^	−0.20^**^	0.02	0.10	
12.	Dismissive attachment (RQ)	0.03	0.15	−0.09	−0.06	−0.20^**^	−0.14	−0.02	0.16 ^*^	−0.04	−0.05	−0.23^**^

ReSta, Satisfaction with Relationship Scale; SWEMWBS, Short Warwick Edinburgh Mental Wellbeing Scale; CAPE, Community Assessment of Psychic Experiences; LS, Loneliness Scale; ISMI-10, Internalised Stigma of Mental Illness Inventory—10 item version; SERS-SF, Self Esteem Rating Scale—Short Form; RQ, Relationship Questionnaire. ^*^p < 0.05, ^**^p < 0.01, ^***^p < 0.001.

To explore hypothesis one, the association between relationship status and satisfaction with romantic relationship status was investigated using hierarchical regression analysis (Table 4). The total number of participants with data included in the model was 148. At step one, the inclusion of relationship status accounted for 27% of the explained variance in satisfaction with relationship status. The inclusion of fearful attachment at step two significantly increased the prediction of satisfaction with relationship status scores (31% of the variance), *F*(1, 145) = 8.26, *p* < 0.01. However, the inclusion of demographic variables did not result in further overall improvement in the prediction of ReSta scores *F*(5, 140) = 1.55, *p* = 0.18. In the final model, being in a relationship and female gender was significantly associated with higher scores for relationship status satisfaction. Fearful attachment was significantly negatively associated with relationship status satisfaction, whereby those higher in fearful attachment were less satisfied with their current relationship status.

**Table 4:** The effect of relationship status and fearful attachment on satisfaction with relationship status (ReSta).

	*R^2^*	*B*	*SE B*	*β*	*p*
**Satisfaction with Relationship Status (ReSta)**					
*Step 1*	0.27				<0.001
Constant		7.64	0.45		<0.001
Relationship status		4.77	0.65	0.52	<0.001
*Step 2*	0.31				<0.001
Constant		9.80	0.87		<0.001
Relationship status		4.70	0.64	0.51	<0.001
Fearful attachment		−0.45	0.16	−0.20	0.005
*Step 3*	0.34				<0.001
Constant		10.75	1.65		<0.001
Relationship status		4.41	0.69	0.48	<0.001
Fearful attachment		−0.57	0.17	−0.25	<0.001
Gender		−1.60	0.67	−0.17	0.018
Age		0.00	0.03	0.01	0.902
Ethnicity		0.30	0.87	0.02	0.731
Sexuality		0.86	0.87	0.08	0.324
Employment		0.04	0.71	0.00	0.958

Relationship status dichotomised: single 0, partner 1. Gender dichotomised: female 0, male 1. Adjusted R^2^: step 1 = 0.26, step 2 = 0.30, step 3 = 0.31.

To test hypothesis two, regression analysis was conducted on a subgroup of 70 participants who were in a relationship (Table 5). At step one, the inclusion of fearful attachment accounted for 8% of the explained variance in satisfaction with relationship status. In step two of the model, the inclusion of partner criticism significantly increased the explained variance in satisfaction with relationship status scores (22% of the variance), *F*(1, 67) = 11.63, *p* = 0.001. However, in this step, fearful attachment was no longer significantly associated with satisfaction with relationship status scores. The inclusion of socio-demographic variables at step three did not significantly increase the variance explained, *F*(5, 62) = 0.44, *p* = 0.822. In the final model, only partner criticism was associated with relationship satisfaction. Specifically, those who rated their partner as more critical, reported being less satisfied with their current romantic relationship status.

**Table 5:** The effect of fearful attachment and criticism on satisfaction with relationship status (ReSta) in those with a partner.

	*R^2^*	*B*	*SE B*	*β*	*p*
**Satisfaction with Relationship Status (ReSta)**					
*Step 1*	0.08				0.015
Constant		14.52	0.91		<0.001
Fearful attachment		−0.46	0.18	−0.29	0.015
*Step 2*	0.22				<0.001
Constant		15.58	0.91		<0.001
Fearful attachment		−0.23	0.18	−0.14	0.227
Criticism		−0.42	0.12	−0.40	0.001
*Step 3*	0.25				0.011
Constant		16.29	1.87		<0.001
Fearful attachment		−0.24	0.21	−0.15	0.248
Criticism		−0.43	0.13	−0.41	0.002
Gender		0.20	0.78	0.03	0.795
Age		−0.02	0.03	−0.08	0.508
Ethnicity		0.43	1.19	0.04	0.716
Sexuality		0.82	0.90	0.12	0.361
Employment		−0.14	0.76	−0.02	0.853

Adjusted R^2^: step 1 = 0.07, step 2 = 0.20, step 3 = 0.16.

**Figure 2:**
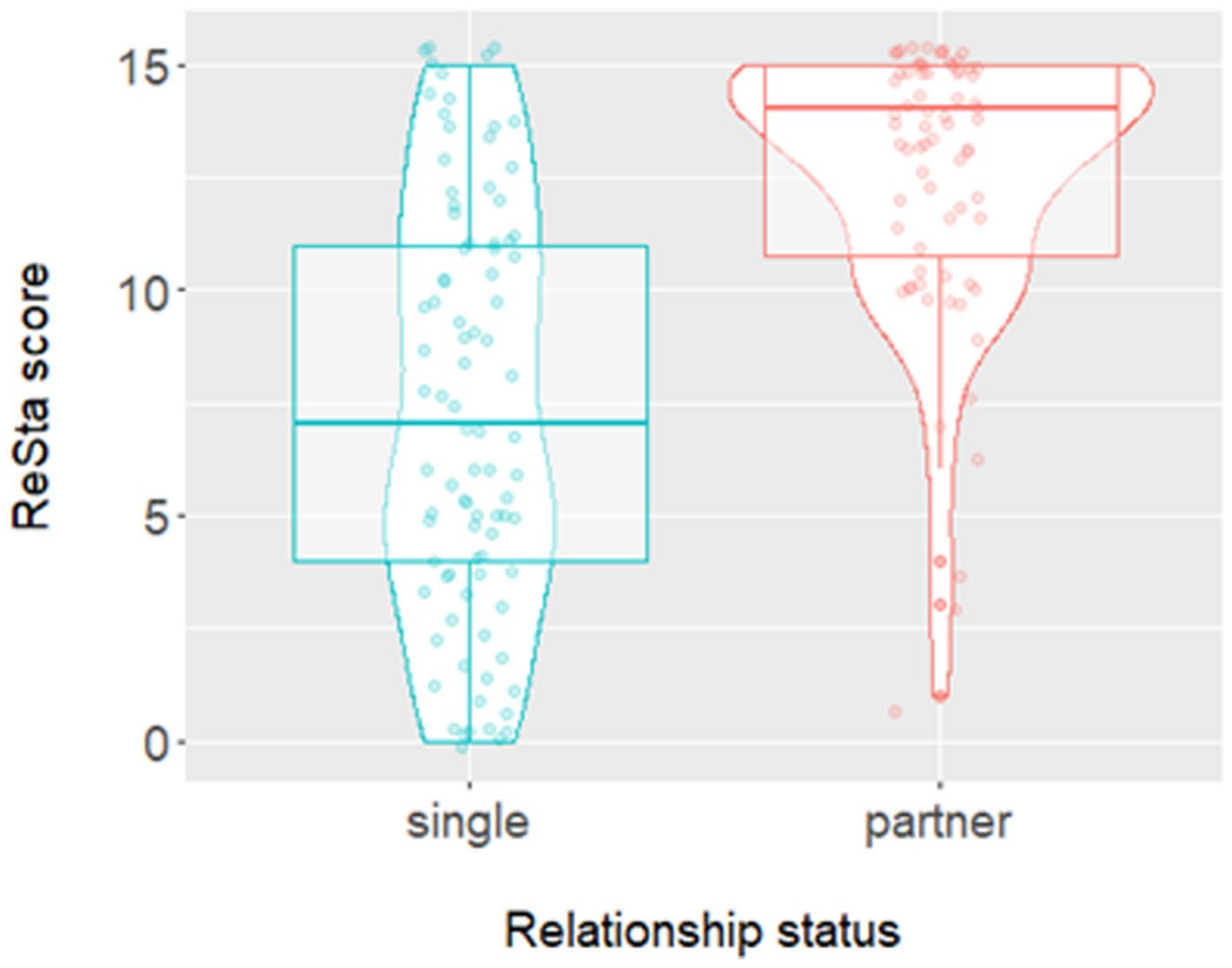
A violin plot with observations to show the relationship between relationship status and satisfaction with relationship status.

To explore the association between relationship status satisfaction and wellbeing outcomes, further hierarchical regression analyses were conducted. Table 6 shows the effect of satisfaction with relationship status on mental wellbeing. Data from 162 participants were included in this analysis. At step one, the inclusion of satisfaction with relationship status accounted for 11% of the explained variance in mental wellbeing. In step two, the addition of socio-demographic variables significantly increased the amount variance in mental wellbeing scores explained by the model (32% of the variance), *F*(6, 154) = 8.00, *p* < 0.001. In the final model, higher satisfaction with relationship status was associated with higher mental wellbeing scores. Being male as well as working or being in full-time education was also linked to higher mental wellbeing scores, whereas identifying as LGBQ+ was associated with lower scores. Unexpectedly, having a partner seemed to be negatively associated with mental wellbeing. Further analysis was conducted to explore the possibility that relationship status was acting as a suppressor variable in this model [i.e., an independent variable appearing to have a significant effect because another correlated independent variable in the model is being held constant (49)]. A Welch’s two sample *t*-test revealed an association between ReSta and relationship status. On average, participants who had a partner were significantly more satisfied with their romantic relationship status than participants without a partner [*M* = 12.53, *SD* = 3.05 vs. *M* = 7.28, *SD* = 4.64; *t*(154.53) = 8.65, *p* < 0.001], furthermore the effect size was large (*d* = 1.31). A visualisation of the data can be seen at Figure 2. Relationship status satisfaction and mental wellbeing scores were also significantly positively correlated (*r* = 0.31, *p* < 0.001). However, the effect of relationship status on mental wellbeing was negligible (*d* = 0.01) and a *t*-test showed no significant difference in the mean mental wellbeing scores of those with a partner (*M =* 18.95, *SD* = 4.15) compared to those without a partner [*M* = 18.92, *SD =* 4.80; *t*(159.02) = 0.05, *p* = 0.964]. The analyses confirmed that relationship status was a suppressor variable and only appeared to be significantly negatively associated with mental wellbeing because the effect of relationship status satisfaction on mental wellbeing was being held constant.

**Table 6:** The effect of satisfaction with relationship status (ReSta) on mental wellbeing (SWEMWBS).

	*R^2^*	*B*	*SE B*	*β*	*p*
**Mental wellbeing (SWEMWBS)**					
*Step 1*	0.11				<0.001
Constant		15.95	0.75		<0.001
Relationship status satisfaction (ReSta)		0.31	0.07	0.33	<0.001
*Step 2*	0.32				<0.001
Constant		13.91	1.41		<0.001
Relationship status satisfaction (ReSta)		0.50	0.08	0.53	<0.001
Gender		1.84	0.63	0.20	0.004
Age		0.00	0.03	−0.01	0.919
Ethnicity		0.42	0.83	0.03	0.611
Sexuality		−2.71	0.81	−0.25	<0.001
Relationship status		−3.22	0.76	−0.36	<0.001
Employment		3.01	0.67	0.33	<0.001

Relationship status dichotomised: single 0, partner 1. Gender dichotomised: female 0, male 1. Sexuality dichotomised: heterosexual 0, LGBQ+ 1. Employment: unemployed 0, employed 1. Adjusted R^2^: step 1 = 0.010, step 2 = 0.29.

Table 7 presents the results on hierarchical regression analysis of satisfaction with relationship status on CAPE depressive scores. Complete data from 165 participants were included in the analysis. At step one, the inclusion of satisfaction with romantic relationship status accounted for 5% of the explained variance in CAPE depressive scores. In step two the inclusion of sociodemographic covariates significantly increased the amount of variance in CAPE depressive scores explained by the model to 31%, *F*(6, 157) = 10.05, *p* < 0.001. In the final model higher satisfaction with relationship status was associated with lower CAPE depressive scores. Being male, older and working or being in full-time education was also linked to lower CAPE depressive scores. Identifying as LGBQ+ was associated with higher CAPE depressive scores. As in the previous analysis, having a partner seemed to be positively associated with CAPE depressive scores. Further analyses were carried out to check whether relationship status was a suppressor variable. As described above, there was a significant association between satisfaction with relationship status and relationship status [*t*(157.55) = 8.37, *p* < 0.001] whereby, on average, those with a partner were more satisfied with their relationship status than those without a partner (*M* = 12.48, *SD* = 3.06 vs. *M* = 7.41*, SD* = 4.68, respectively). The effect size for this association was large (*d* = 1.25). There was also a significant negative correlation between satisfaction with relationship status and CAPE depressive scores (*r* = − 0.22, *p* = 0.004). However mean CAPE depressive scores did not differ significantly between those with a partner (*M* = 2.57, *SD* = 0.73) and those without (*M* = 2.51, *SD* = 0.77), *t*(158.16) = 0.47, *p* = 0.641 and the effect size was negligible (*d = 0*.07). Therefore, as in the previous model, it was concluded relationship status was a suppressor variable.

**Table 7:** The effect of satisfaction with relationship status (ReSta) onCAPE depressive scores.

	*R^2^*	*B*	*SE B*	*β*	*p*
**CAPE depressive score**					
*Step 1*	0.05				0.004
Constant		2.88	0.13		<0.001
Relationship status satisfaction (ReSta)		−0.04	0.01	−0.22	0.004
*Step 2*	0.31				<0.001
Constant		3.70	0.24		<0.001
Relationship status satisfaction (ReSta)		−0.07	0.01	−0.42	<0.001
Gender		−0.45	0.11	−0.29	<0.001
Age		−0.01	0.00	−0.16	0.033
Ethnicity		−0.18	0.14	−0.09	0.192
Sexuality		0.42	0.13	0.23	0.002
Relationship status		0.44	0.12	0.29	<0.001
Employment		−0.48	0.11	−0.32	<0.001

To test hypothesis four, mediation analysis considered the pathways between satisfaction with romantic relationship status satisfaction and all outcome variables (SWEMWBS and all CAPE subscales) as all were shown to be significantly correlated with the mediator variables. Simple mediation models were tested initially and then, where significant, with socio-demographic covariates. Results of mediation analysis after the inclusion of covariates gender, age, ethnicity, sexuality, relationship status and employment are presented in Table 8. Significant indirect effects were found for all models. Results suggest that higher satisfaction with romantic relationship status is associated with higher scores for mental wellbeing and lower scores for positive, negative, and depressive symptoms of psychosis, via lower internalised stigma and loneliness and higher self-esteem.

**Table 8:** Point estimates [95% CI] for the total, direct and indirect effects of relationship status satisfaction (ReSta) on mental wellbeing (SWEMWBS) and CAPE subscale scores.

Outcome variable	Mediator	*n*	Indirect effect	*p*	Direct effect	*p*	Total effect	*p*
Mental wellbeing (SWEMWBS)	Internalised stigma (ISMI-10)	159	0.15 [0.07, 0.25]	<0.001	0.38 [0.25, 0.51]	<0.001	0.53 [0.38, 0.68]	<0.001
	Loneliness (LS)	157	0.32 [0.20, 0.45]	<0.001	0.19 [0.02, 0.36]	0.026	0.51 [0.36, 0.67]	<0.001
	Self-esteem (SERS-SF)	153	0.33 [0.21, 0.46]	<0.001	0.19[0.06, 0.30]	0.002	0.52[0.35, 0.67]	<0.001
CAPE positive	Internalised stigma (ISMI-10)	160	−0.02 [−0.04, −0.01]	0.001	−0.01 [−0.03, 0.02]	0.582	−0.03 [−0.06, 0.00]	0.039
	Loneliness (LS)	158	−0.04 [−0.06, −0.02]	<0.001	0.01 [−0.02, 0.04]	0.540	−0.03 [−0.06, 0.00]	0.03
	Self-esteem (SERS-SF)	153	−0.03 [−0.05, −0.02]	<0.001	0.00 [− 0.02, 0.03]	0.937	−0.03 [−0.06, 0.00]	0.023
CAPE negative	Internalised stigma (ISMI-10)	160	−0.02 [−0.04, −0.01]	<0.001	−0.01 [−0.03, 0.01]	0.152	−0.04 [−0.06, −0.02]	<0.001
	Loneliness (LS)	158	−0.05 [−0.06, −0.03]	<0.001	0.00 [−0.02, 0.03]	0.930	−0.04 [−0.07, −0.02]	<0.001
	Self-esteem (SERS-SF)	153	−0.04 [−0.05, −0.02]	<0.001	−0.01 [−0.03, 0.01]	0.386	−0.05 [−0.07, −0.02]	<0.001
CAPE depressive	Internalised stigma (ISMI-10)	161	−0.03 [−0.05, −0.01]	<0.001	−0.04 [−0.06, −0.02]	<0.001	−0.07 [−0.10, −0.05]	<0.001
	Loneliness (LS)	158	−0.06 [−0.08, −0.04]	<0.001	−0.01 [−0.04, 0.01]	0.290	−0.08 [−0.10, −0.05]	<0.001
	Self-esteem (SERS-SF)	152	−0.05 [−0.07, −0.03]	<0.001	−0.03 [−0.04, −0.01]	0.004	−0.08 [−0.10, −0.05]	<0.001

ReSta, Satisfaction with Relationship Scale; SWEMWBS, Short Warwick Edinburgh Mental Wellbeing Scale; CAPE, Community Assessment of Psychic Experiences; LS, Loneliness Scale; ISMI-10, Internalised Stigma of Mental Illness Inventory—10 item version; SERS-SF, Self Esteem Rating Scale—Short Form.

## 4. Discussion

This study sought to explore the association between relationship status satisfaction and mental wellbeing outcomes in people who have experienced psychosis. Results suggested that having a romantic partner, scoring lower in fearful attachment style and being female are all associated with higher satisfaction with romantic relationship status scores. Interestingly, sub-group analysis of those in a romantic relationship found that fearful attachment and gender were not associated with relationship status satisfaction. For those who were in a relationship, only perceived criticism from a partner was associated with relationship satisfaction. Specifically, those who rated their partner as more critical were less satisfied with their romantic relationship status. Satisfaction with romantic relationship status was also found to be associated with mental wellbeing and CAPE depressive scores but not CAPE positive or negative scores. Further analysis found that this association remained when socio-demographic variables were included in the models. Male gender, identifying as heterosexual and working/being in full time education were also associated with higher mental wellbeing and lower CAPE depressive scores. Age was negatively associated with CAPE depressive scores. Mediation analysis found significant indirect effects between relationship status satisfaction, mental wellbeing and all CAPE subscales via internalised stigma, loneliness, and self-esteem.

Previous studies have identified intimate relationships as a predominant area of unmet need for people who experience psychosis (50, 51). The present study found having a partner was positively associated with relationship status satisfaction. This finding may be attributed to the universal importance placed on romantic relationships. Having a partner is embedded within social goals and developmental milestones for many (52), thus those who aspire to have a partner, but are single may be dissatisfied with their relationship status. Alternatively, when one or both people in a romantic relationship are no longer happy with the partnership, the relationship may end. As such the finding may simply be explained by the fact that those dissatisfied in their romantic relationship, chose to become single. As can be seen in Figure 2, some participants were highly satisfied with being single. This serves as a reminder that, measuring *satisfaction with relationship status* or relationship quality, is likely to be more meaningful and give greater insights compared to simply measuring relationship status.

Female gender and lower levels of fearful attachment were also associated with higher satisfaction with relationship status scores. Fearful attachment is characterised by high avoidance and anxiety regarding intimacy. Individuals with a fearful attachment style are worried about rejection which may prevent them forming a relationship, even if one is desired. Additionally, those in a relationship may not benefit from relationships in the same way as those who are securely attached due to using less effective strategies to seek support (19). The finding that female participants were more satisfied with their romantic relationship status may be explained in two ways. Firstly, the onset of psychosis typically occurs earlier in men than women (53). Men may be more likely to experience psychosis during a time that is typically important for experiencing first relationships and developing related skills. As a result, men may be less skilled at forming and maintaining relationships than women later in life, thus more likely to be single even when they wish to be in a relationship. Alternatively, women may be more comfortable being single than men. Recent qualitative studies have found heterosexual women minimised the importance of relationships, or avoided relationships due to previous abuse from partners and the larger caretaking burden placed on women in relationships with men (54, 55).

When considering those who were in a relationship, only partner’s perceived criticism was associated with romantic relationship satisfaction. This is in line with wider literature which has reported strong negative correlations between criticism and relationship satisfaction (56). In clinical populations, high levels of criticism from family members has also been linked to increased rates of relapse (17). In dyad studies of EE involving people who have experienced psychosis, family members tend to be parents (57), with results often assumed to be generalisable to other social contacts. However other studies have indicated romantic partners display lower EE than parents (58). This is important to note as qualitative research suggests mental health practitioners often think about the romantic relationships of services users with psychosis as turbulent or ‘risky’ (59). Whilst this may be the case for some, it is imperative to remember that the majority of those with a partner in this study were highly satisfied with their relationship status.

In this study, satisfaction with relationship status was not significantly associated with positive or negative symptoms of psychosis. However, those who were more satisfied with their romantic relationship status reported significantly better mental wellbeing and fewer depressive symptoms of psychosis. Although relationship status satisfaction only accounted for a relatively small amount of the variance in mental wellbeing and depression, the association remained significant when socio-demographic variables were accounted for. Gender, sexuality and employment were also significantly associated for both outcomes and age was significantly associated with depression only. Findings related to sexuality are in line with previous literature which suggests people who identify as LGBQ+ *and* experience psychosis face intersectional discrimination and may therefore have worse mental health outcomes (60). Similar findings may have been expected for ethnicity (61) but were not found, possibly due to the majority of the sample in this study being white. Regarding employment, general and professional activities have been identified as a domain of wellbeing for people who experience psychosis (62). Involvement in valued roles may improve wellbeing in multiple ways such as connecting with and being valued by others, developing strengths and abilities, as well as improved finances (63, 64). Men were found to have better mental wellbeing and fewer depressive symptoms than women. This is in line with previous studies that have found women report more symptoms of depression than men (65). Higher rates of depression in women have been attributed to numerous factors including: men being less likely to recognise and seek help for experiences of depression, biological factors, increased stress and violence experienced by girls and women, as well as other gender inequalities (66). Finally, given the high prevalence of childhood adversity in those who experience psychosis (67) and side effects associated with anti-psychotic medication—such as weight gain, cardiovascular and metabolic conditions (68, 69); a tentative explanation for the finding that being older was linked to lower scores for depression is that the association between depression and childhood trauma, BMI and long-lasting physical conditions has been shown to be stronger in those who are younger (70).

Feeling dissatisfied with one’s romantic relationship status – either through being single and desiring a partner *or* having an unsatisfactory relationship – was associated with higher levels of loneliness and internalised stigma and lower self-esteem. Within satisfactory relationships, having a partner may reduce loneliness by providing companionship and a sense of being valued by others (71). Those in supportive relationships may also experience increased self-esteem due to adopting the positive views their partners have about them (72). Additionally, being somebody’s partner may be a valued aspect of identity. Individuals who experience psychosis may develop an identity of ‘someone who is mentally ill’, ‘a patient’ or ‘service user’. Thoits suggested that such a role identity could lead to individuals experiencing internalised stigma due to the negative stereotypes applied to such labels by the media, others and therefore, themselves (73). Indeed, qualitative findings suggest that people with lived experience of psychosis perceive themselves as undesirable partners because of their diagnosis (22). As such having a satisfactory relationship and identifying as a ‘partner’, ‘boyfriend’, or girlfriend’ is likely to be of benefit, especially because roles which require a high level of commitment are thought to be particularly beneficial when not stressful. For people experiencing psychosis, having numerous roles may make it easier to recognise that mental illness is only a small part of one’s identity which could protect against internalised stigma. Additionally, identification with more roles has been linked to increased self-esteem (74), which has been negatively associated with persecutory delusions (75). In this way, role identity theory is also applicable to those who are satisfied identifying as ‘single’ too.

Despite an association not being found between relationship status satisfaction and positive or negative symptoms of psychosis, it was still appropriate to test for indirect pathways between these variables (76). Internalised stigma, loneliness and self-esteem were all found to mediate the association between satisfaction with relationship status and all wellbeing outcomes. Although no direct associations were found between relationship status satisfaction and positive or negative symptoms of psychosis, there were small significant effects when pathways via internalised stigma, loneliness and self-esteem were considered, indicating that the associations between the symptoms of psychosis are subtle and therefore may potentially be missed in research designs that do not specifically aim to investigate such relationships.

### 4.1. Limitations

This paper has several limitations that must be considered when interpreting the results. Firstly, the analysis is cross-sectional meaning the direction of influence cannot be assumed. Satisfying romantic relationships may be protective against the symptoms of psychosis but equally, psychotic experiences may be a source of tension, leading to dissatisfaction within romantic relationships. Similarly, it is possible that bi-directional relationships exist between relationship satisfaction and mediator variables. For example, previous research has found low self-esteem to be predictive of subsequent perceived decreases in relationship quality (77). It has been suggested that people with higher self-esteem may be more likely to behave in a way which maintains relationship satisfaction. A diary study found those with high self-esteem were more likely to engage in positive behaviours towards their partner to re-establish closeness after feeling rejected or hurt by them. Comparatively, those low in self-esteem, reported displaying more negative behaviours (e.g., criticising, ignoring, insulting) towards their partner (78). More longitudinal research is needed to explore these potentially bi-directional relationships.

Secondly, although demographic covariates were included in the analysis, it is possible that results are due to unaccounted for confounding variables, such as childhood adversity, pre psychosis functioning or other unknown variables. Additionally, correlations and regression analyses were conducted without a correction being applied to the *p* value that was considered significant, increasing the risk of a type one error for these tests.

Thirdly, a convenience sample was used meaning results are likely to be subject to self-selection bias. Although paper-based surveys were available, much of recruitment was done online via social media, meaning those who do not use these platforms are less likely to be represented in the results. Additionally, information about participants’ demographics and diagnoses were not corroborated and it was not possible to know how many participants were currently experiencing psychosis. Similarly, although outliers were removed from the analysis, recommended measures to check for inattentive responding, i.e., participants completing measures randomly or without reading items, were not included (79). Due to data collection being online, true non-responder bias was not possible to calculate, however comparison of those who completed the demographic information but did not provide enough data to be included in the analysis showed that non responders were more likely to have a partner than participants. Furthermore, the inclusion criteria were broad meaning participants in this study represent a heterogeneous group. For example, people with a primary schizophrenia spectrum diagnosis as well as those without a formal diagnosis were eligible to participate and no stipulations were placed on the duration of experience. The relationship between the variables of interest in this study may differ depending on the nature of an individual’s experience of psychosis. As such, those conducting future research may wish to recruit a more homogenous sample to better understanding of the experience of specific groups. Finally, most participants were white and heterosexual, therefore comparisons based on ethnicity and sexual orientation may be underpowered. Dichotomising demographic variables means the analysis does not account for the diversity within the groups created. For example, dichotomising sexuality meant all those who identified at LGBQ+ were grouped together. This is problematic as it does not account for or recognise that different groups within this community will have different experiences. Similarly, dichotomising ethnicity as ‘white’ and ‘people of the global majority’ does not account for the wide diversity within these two groups. Dichotomising gender meant participants who identified as trans or non-binary were not included in the analysis. Unfortunately, the total sample and sub-groups were too small to allow for meaningful analysis to be done separately.

### 4.2. Implications

Future research should aim to test the associations found in this paper longitudinally. Additionally, given the gender and sexuality differences found, future studies may wish to focus on recruiting participants with specific demographics and types of relationship experience to explore these associations more thoroughly. Regarding clinical practice, results indicate romantic relationships are associated with mental wellbeing and therefore should be considered as an area for support by mental health professionals. Service users with a fearful attachment style may be particularly dissatisfied with their romantic relationship status and benefit from support in this area. However, findings from qualitative interviews with service users with experience of psychosis indicate that support with romantic relationship issues may not always be welcomed by people with experience of psychosis (22). This finding appeared to be particularly related to the power imbalance between mental health professionals and service users. Support with romantic relationships was only acceptable to participants in this study once a trusting, therapeutic alliance had been established. Where this is the case, clinicians should approach conversations about romantic relationships sensitively and maintain service user’s autonomy over the direction of the conversation and decisions regarding intimate relationships (22).

## Data availability statement

The raw data supporting the conclusions of this article will be made available by the authors, without undue reservation.

## Ethics statement

This study was approved by the North West - Preston Research Ethics Committee (ref: 19/NW/0665). The study was conducted in accordance with the local legislation and institutional requirements. Written informed consent for participation was not required from the participants or the participants’ legal guardians/next of kin because a proportionate approach to consent was taken.

## Author contributions

RW, GH and FV were responsible for funding acquisition and design of the study. RW collected and analysed the data with supervision and support from GH and FV. RW wrote the manuscript, feedback on which was provided by MH, GH and FV. All authors contributed to the article and approved the submitted version.
