# Supplementary material for: Being more satisfied with romantic relationship status is associated with increased mental wellbeing in people with experience of psychosis
Source: Front Psychiatry. 2023 Sep 28;14:1232973. doi: 10.3389/fpsyt.2023.1232973 (PMC10569177; doi:10.3389/fpsyt.2023.1232973)

Hypothesis 2

Rebecca White

25/01/2022

### Load packages and data set

library(tidyverse)

## -- Attaching packages --------------------------------------- tidyverse 1.3.0 --

## v ggplot2 3.3.2 v purrr 0.3.4
## v tibble 3.0.4 v dplyr 1.0.2
## v tidyr 1.1.2 v stringr 1.4.0
## v readr 1.4.0 v forcats 0.5.0

## -- Conflicts ------------------------------------------ tidyverse_conflicts() --
## x dplyr::filter() masks stats::filter()
## x dplyr::lag() masks stats::lag()

library(QuantPsyc)

## Loading required package: boot

## Loading required package: MASS

##
## Attaching package: 'MASS'

## The following object is masked from 'package:dplyr':
##
## select

##
## Attaching package: 'QuantPsyc'

## The following object is masked from 'package:base':
##
## norm

library(car)

## Loading required package: carData

##
## Attaching package: 'car'

## The following object is masked from 'package:boot':
##
## logit

## The following object is masked from 'package:dplyr':
##
## recode

## The following object is masked from 'package:purrr':
##
## some

library(readr)

Dataset_190_obs_2_9_21 <- read_csv("Z:/Online study IRAS ID 271957/Online analysis/Dataset_190_obs_2.9.21.csv")

## Warning: Missing column names filled in: 'X1' [1]

##
## -- Column specification --------------------------------------------------------
## cols(
## .default = col_double(),
## redcap_survey_identifier = col_logical(),
## pis_timestamp = col_datetime(format = ""),
## screening_questions_timestamp = col_datetime(format = ""),
## demographic_information_timestamp = col_datetime(format = ""),
## nationality = col_character(),
## ethnicity_other = col_character(),
## gender_self_describe = col_character(),
## sexual_orientation_selfdescribe = col_character(),
## rr_selfdescribe = col_character(),
## last_rr_end = col_character(),
## current_rr_length = col_character(),
## the_community_assessment_of_psychic_experiences_ca_timestamp = col_datetime(format = ""),
## the_short_warwick_mental_health_wellbeing_scale_timestamp = col_datetime(format = ""),
## adapted_satisfaction_with_relationships_scale_rest_timestamp = col_datetime(format = ""),
## three_item_loneliness_scale_timestamp = col_datetime(format = ""),
## internalised_stigma_of_mental_illness_inventory_10_timestamp = col_datetime(format = ""),
## multidimensional_scale_of_perceived_social_support_timestamp = col_datetime(format = ""),
## self_esteem_rating_scale_short_form_serssf_timestamp = col_datetime(format = ""),
## relationships_questionnaire_timestamp = col_datetime(format = ""),
## Screening_Qs_result = col_character()
## # ... with 7 more columns
## )
## i Use `spec()` for the full column specifications.

### Create data frame to work from

data.frame2 <- data.frame(Dataset_190_obs_2_9_21$Resta.total,
 Dataset_190_obs_2_9_21$GenderF,
 Dataset_190_obs_2_9_21$age,
 Dataset_190_obs_2_9_21$EthnicityF,
 Dataset_190_obs_2_9_21$SexualityF,
 Dataset_190_obs_2_9_21$R_Status_simplified,
 Dataset_190_obs_2_9_21$rr_selfdescribe,
 Dataset_190_obs_2_9_21$EmploymentF,
 Dataset_190_obs_2_9_21$ethnicity_other)


#rename columns
names(data.frame2)[names(data.frame2) ==
 "Dataset_190_obs_2_9_21.SWEMWBS_metric"]<- "SWEMWBS"
names(data.frame2)[names(data.frame2) ==
 "Dataset_190_obs_2_9_21.Resta.total"]<- "Resta"
names(data.frame2)[names(data.frame2) ==
 "Dataset_190_obs_2_9_21.GenderF"]<- "gender"
names(data.frame2)[names(data.frame2) ==
 "Dataset_190_obs_2_9_21.age"]<- "age"
names(data.frame2)[names(data.frame2) ==
 "Dataset_190_obs_2_9_21.EthnicityF"]<- "ethnicity"
names(data.frame2)[names(data.frame2) ==
 "Dataset_190_obs_2_9_21.SexualityF"]<- "sexuality"
names(data.frame2)[names(data.frame2) ==
 "Dataset_190_obs_2_9_21.R_Status_simplified"]<- "relationship.status"
names(data.frame2)[names(data.frame2) ==
 "Dataset_190_obs_2_9_21.EmploymentF"]<- "employment"

#Add in fearful attachment and criticism
data.frame2$fear_attach <- Dataset_190_obs_2_9_21$fearful_rating
data.frame2$criticism <- Dataset_190_obs_2_9_21$ee_criticism

#CREATE DICHOTOMOUS VARIABLES
#GENDER
#Gender is categorised as 1= female, 2 = male, 3 = prefer not to say, 4 = prefer to self describe
table(data.frame2$gender)

##
## female male Self_describe
## 105 69 6

#remove 'prefer not to say & self describe

data.frame2$gender <- factor(data.frame2$gender,
 c("female","male"), c("female", "male"))
table(data.frame2$gender)

##
## female male
## 105 69

#ETHNICITY
table(data.frame2$ethnicity)

##
## asian black mixed other white
## 10 11 5 5 148

data.frame2 %>%
 mutate(ethnicity.dicotomised = case_when(ethnicity == "other" & Dataset_190_obs_2_9_21.ethnicity_other == "White Scottish" ~ "white",
 ethnicity == "white" ~ "white",
 ethnicity == "mixed" ~ "PGM",
 ethnicity == "asian" ~ "PGM",
 ethnicity == "black" ~ "PGM",
 ethnicity == "chinese" ~ "PGM",
 ethnicity == "other" ~ "PGM")) -> data.frame2

table(data.frame2$ethnicity.dicotomised)

##
## PGM white
## 29 150

#SEXUALITY
table(data.frame2$sexuality)

##
## bisexual gay/lesbian heterosexual prefer not to say
## 24 9 132 6
## self-describe
## 9

#checked what participants have written for self-describe sexuality & happy to put all in non-heterosexual group

data.frame2 %>%
 mutate(sexuality.dicotomised = case_when(sexuality == "heterosexual" ~ "heterosexual",
 sexuality == "bisexual" ~ "LGBQ+",
 sexuality == "gay/lesbian" ~ "LGBQ+",
 sexuality == "self-describe" ~ "LGBQ+")) -> data.frame2
table(data.frame2$sexuality.dicotomised)

##
## heterosexual LGBQ+
## 132 42

#RELATIONSHIP STATUS, single vs partner
table(data.frame2$relationship.status)

##
## dating partner self describe separated single
## 5 78 5 2 89

data.frame2 %>%
 mutate(relationship.dicotomised = case_when(relationship.status == "single" ~ "single",
 relationship.status == "dating" ~ "single",
 relationship.status == "separated" ~ "single",
 relationship.status == "widowed" ~ "single",
 relationship.status == "partner" ~ "partner",
 relationship.status == "self describe" & Dataset_190_obs_2_9_21.rr_selfdescribe == "Living with a wife, queerplatonic partner, and steady, with one long-distance relationship as well" ~ "partner",
 relationship.status == "self describe" ~ "single")) -> data.frame2

table(data.frame2$relationship.dicotomised)

##
## partner single
## 79 100

#EMPLOYMENT , paid employment/ education vs not
table(data.frame2$employment)

##
## employee FT education
## 56 21
## looking after home/family rec. sickness/disability benefits
## 6 58
## retired self-employed
## 5 5
## unemployed
## 26

data.frame2 %>%
 mutate(employment.dicotomised = case_when ( employment == "employee" ~ "working/FT.education",
 employment == "self-employed" ~ "working/FT.education",
 employment == "FT education" ~ "working/FT.education",
 employment == "unemployed" ~ "unemployed",
 employment == "looking after home/family" ~ "working/FT.education",
 employment == "rec. sickness/disability benefits" ~ "unemployed",
 employment == "retired" ~ "unemployed")) -> data.frame2

table(data.frame2$employment.dicotomised)

##
## unemployed working/FT.education
## 89 88

#remove columns that are mainly NA e.g. rr_self describe so they don't interfere with next stages
drops <- c("Dataset_190_obs_2_9_21.rr_selfdescribe", "Dataset_190_obs_2_9_21.ethnicity_other")
data.frame2[ , !(names(data.frame2) %in% drops)] -> data.frame2


#Create dummy variables
#gender
Female0_v_Male1 <- c(0, 1)
contrasts(data.frame2$gender) <- cbind(Female0_v_Male1)

#convert new dictomised variables to factors
names <- c('ethnicity.dicotomised', 'sexuality.dicotomised', 'relationship.dicotomised', 'employment.dicotomised')
data.frame2[,names]<- lapply(data.frame2[,names],factor)
str(data.frame2)

## 'data.frame': 190 obs. of 13 variables:
## $ Resta : num 15 14 15 13 10 10 14 13 15 14 ...
## $ gender : Factor w/ 2 levels "female","male": 1 1 1 1 1 2 2 1 1 1 ...
## ..- attr(*, "contrasts")= num [1:2, 1] 0 1
## .. ..- attr(*, "dimnames")=List of 2
## .. .. ..$ : chr [1:2] "female" "male"
## .. .. ..$ : chr "Female0_v_Male1"
## $ age : num 32 22 31 27 45 50 25 32 25 39 ...
## $ ethnicity : chr "white" "white" "mixed" "white" ...
## $ sexuality : chr "heterosexual" "bisexual" "heterosexual" "heterosexual" ...
## $ relationship.status : chr "partner" "partner" "partner" "partner" ...
## $ employment : chr "FT education" "employee" "employee" "FT education" ...
## $ fear_attach : num 2 6 5 5 7 5 6 7 2 5 ...
## $ criticism : num 3 3 10 4 7 7 1 7 1 8 ...
## $ ethnicity.dicotomised : Factor w/ 2 levels "PGM","white": 2 2 1 2 2 1 2 2 NA 2 ...
## $ sexuality.dicotomised : Factor w/ 2 levels "heterosexual",..: 1 2 1 1 1 1 NA 1 NA 1 ...
## $ relationship.dicotomised: Factor w/ 2 levels "partner","single": 1 1 1 1 1 1 1 1 1 1 ...
## $ employment.dicotomised : Factor w/ 2 levels "unemployed","working/FT.education": 2 2 2 2 2 2 1 1 2 2 ...

#ethnicity
White0_v_PGM1 <- c(1, 0)
contrasts(data.frame2$ethnicity.dicotomised) <- cbind(White0_v_PGM1)

#sexuality
heter0_v_LGBQ1 <- c(0, 1)
contrasts(data.frame2$sexuality.dicotomised) <- cbind(heter0_v_LGBQ1)

#relationship status
single0_v_partner1 <- c(1,0)
contrasts(data.frame2$relationship.dicotomised) <- cbind(single0_v_partner1)

#employment status
unemploy.0_v_work1 <- c(0,1)
contrasts(data.frame2$employment.dicotomised) <- cbind(unemploy.0_v_work1)


## convert dichotomous variables into numeric values -
## this will allow for beta scores to be generated

#gender
data.frame2 %>%
 mutate (gender.num = case_when ( gender == "male" ~ 1,
 gender == "female" ~ 0)) -> data.frame2

#relationship status
data.frame2 %>%
 mutate (rel.num = case_when (relationship.dicotomised == "single" ~ 0,
 relationship.dicotomised == "partner" ~ 1)) -> data.frame2

#ethnicity
data.frame2 %>%
 mutate (ethnicity.num = case_when ( ethnicity.dicotomised == "white" ~ 0,
 ethnicity.dicotomised == "PGM" ~ 1)) -> data.frame2

#sexuality
data.frame2 %>%
 mutate (sex.num = case_when ( sexuality.dicotomised == "heterosexual" ~ 0,
 sexuality.dicotomised == "LGBQ+" ~ 1)) -> data.frame2

#employment
data.frame2 %>%
 mutate (employ.num = case_when ( employment.dicotomised == "unemployed" ~ 0,
 employment.dicotomised == "working/FT.education" ~ 1)) -> data.frame2

#Remove missing data so models can be compared
data.frame2 <- na.omit(data.frame2)
table(data.frame2$nmiss)

## < table of extent 0 >

#Keep only participants who have a partner
table(data.frame2$relationship.dicotomised)

##
## partner single
## 70 14

H2_data <- data.frame2

H2_data %>%
 filter(!(relationship.dicotomised == "single")) -> H2_data

### Build model

**Step 1**

Mod1 <- lm(Resta ~ fear_attach, data = H2_data)
summary(Mod1)

##
## Call:
## lm(formula = Resta ~ fear_attach, data = H2_data)
##
## Residuals:
## Min 1Q Median 3Q Max
## -10.2930 -1.2930 0.8602 2.1490 3.7070
##
## Coefficients:
## Estimate Std. Error t value Pr(>|t|)
## (Intercept) 14.5249 0.9147 15.879 <2e-16 ***
## fear_attach -0.4617 0.1843 -2.505 0.0147 *
## ---
## Signif. codes: 0 '***' 0.001 '**' 0.01 '*' 0.05 '.' 0.1 ' ' 1
##
## Residual standard error: 2.979 on 68 degrees of freedom
## Multiple R-squared: 0.08448, Adjusted R-squared: 0.07101
## F-statistic: 6.274 on 1 and 68 DF, p-value: 0.01465

lm.beta(Mod1)

## fear_attach
## -0.290648

**Step 2**

Mod2 <- lm(Resta ~ fear_attach + criticism, data = H2_data)
summary(Mod2)

##
## Call:
## lm(formula = Resta ~ fear_attach + criticism, data = H2_data)
##
## Residuals:
## Min 1Q Median 3Q Max
## -9.6785 -0.9987 0.2738 1.5523 4.7017
##
## Coefficients:
## Estimate Std. Error t value Pr(>|t|)
## (Intercept) 15.5839 0.9055 17.209 <2e-16 ***
## fear_attach -0.2256 0.1849 -1.221 0.2265
## criticism -0.4157 0.1219 -3.411 0.0011 **
## ---
## Signif. codes: 0 '***' 0.001 '**' 0.01 '*' 0.05 '.' 0.1 ' ' 1
##
## Residual standard error: 2.77 on 67 degrees of freedom
## Multiple R-squared: 0.2199, Adjusted R-squared: 0.1966
## F-statistic: 9.444 on 2 and 67 DF, p-value: 0.0002437

lm.beta(Mod2)

## fear_attach criticism
## -0.1420375 -0.3968914

**Step 3**

Mod3 <- lm(Resta ~ fear_attach + criticism + gender.num + age +
 ethnicity.num + sex.num + employ.num, data = H2_data)
summary(Mod3)

##
## Call:
## lm(formula = Resta ~ fear_attach + criticism + gender.num + age +
## ethnicity.num + sex.num + employ.num, data = H2_data)
##
## Residuals:
## Min 1Q Median 3Q Max
## -9.3144 -1.1244 0.3616 1.5389 4.5586
##
## Coefficients:
## Estimate Std. Error t value Pr(>|t|)
## (Intercept) 16.28522 1.86762 8.720 2.25e-12 ***
## fear_attach -0.23893 0.20501 -1.165 0.24831
## criticism -0.42691 0.13247 -3.223 0.00203 **
## gender.num 0.20276 0.77517 0.262 0.79452
## age -0.02172 0.03258 -0.666 0.50757
## ethnicity.num 0.43462 1.18720 0.366 0.71555
## sex.num 0.82499 0.89648 0.920 0.36100
## employ.num -0.14150 0.76010 -0.186 0.85293
## ---
## Signif. codes: 0 '***' 0.001 '**' 0.01 '*' 0.05 '.' 0.1 ' ' 1
##
## Residual standard error: 2.83 on 62 degrees of freedom
## Multiple R-squared: 0.2464, Adjusted R-squared: 0.1613
## F-statistic: 2.896 on 7 and 62 DF, p-value: 0.011

lm.beta(Mod3)

## fear_attach criticism gender.num age ethnicity.num
## -0.15040779 -0.40755037 0.03136804 -0.08224593 0.04249551
## sex.num employ.num
## 0.11529895 -0.02140875

###Compare models

anova(Mod1, Mod2)

## Analysis of Variance Table
##
## Model 1: Resta ~ fear_attach
## Model 2: Resta ~ fear_attach + criticism
## Res.Df RSS Df Sum of Sq F Pr(>F)
## 1 68 603.32
## 2 67 514.07 1 89.252 11.633 0.001102 **
## ---
## Signif. codes: 0 '***' 0.001 '**' 0.01 '*' 0.05 '.' 0.1 ' ' 1

anova(Mod2, Mod3)

## Analysis of Variance Table
##
## Model 1: Resta ~ fear_attach + criticism
## Model 2: Resta ~ fear_attach + criticism + gender.num + age + ethnicity.num +
## sex.num + employ.num
## Res.Df RSS Df Sum of Sq F Pr(>F)
## 1 67 514.07
## 2 62 496.60 5 17.462 0.436 0.8217

###check assumptions

**Outliers and influential cases**

H2_data$residuals <- resid(Mod3)
H2_data$standardized.residuals <- rstandard(Mod3)
H2_data$studentized.residuals <- rstudent(Mod3)
H2_data$cooks.distance <- cooks.distance(Mod3)
H2_data$dfbeta <- dfbeta(Mod3)
H2_data$dffit <- dffits(Mod3)
H2_data$leverage <- hatvalues(Mod3)
H2_data$covariance <- covratio(Mod3)

H2_data$standardized.residuals > 2 | H2_data$standardized.residuals < -2

## [1] FALSE FALSE FALSE FALSE FALSE FALSE FALSE FALSE FALSE FALSE FALSE TRUE
## [13] FALSE FALSE FALSE FALSE FALSE FALSE FALSE FALSE FALSE FALSE FALSE FALSE
## [25] FALSE FALSE FALSE FALSE FALSE FALSE FALSE FALSE FALSE FALSE FALSE FALSE
## [37] FALSE FALSE FALSE FALSE FALSE FALSE FALSE FALSE FALSE FALSE FALSE FALSE
## [49] FALSE FALSE FALSE FALSE FALSE FALSE FALSE TRUE FALSE TRUE FALSE FALSE
## [61] TRUE FALSE FALSE FALSE FALSE FALSE FALSE TRUE FALSE FALSE

# would expect 95% to be within this range (so approx 67/70)

H2_data$large.residual <- H2_data$standardized.residuals > 2 | H2_data$standardized.residuals < -2
sum(H2_data$large.residual) # 5 cases have a large residual

## [1] 5

H2_data[H2_data$large.residual, c( "standardized.residuals" )]

## [1] -3.535776 -2.151392 -2.016911 -2.365265 -2.972116

One has standardised residual +/- 2.5, One has standardised residual larger than +/- 3

Look at leverage and cooks distance for these cases

H2_data[H2_data$large.residual, c("cooks.distance", "leverage", "covariance" )]

## cooks.distance leverage covariance
## 12 0.24095851 0.13359324 0.2169505
## 56 0.06615803 0.10261533 0.6822651
## 58 0.04316727 0.07824999 0.7179605
## 61 0.03187247 0.04359033 0.5588438
## 68 0.32732889 0.22865943 0.4317393

Cooks distance < 1 so these cases are not having an undue influence on the model

Leverage can be calculated as 0.11 (k+1/n = 8/70) one has leverage values are twice as large as this (0.23) but none are three times as large (0.34)

Covariance 1 - [3(k+1) / n] -> 0.66 1 + [3(k+1) / n] -> 1.34

12, 61 and 68 have CVR values outside these boundaries. However, given Cooks Distance not overly concerned about these.

**Check assumption of independence**

dwt(Mod3)

## lag Autocorrelation D-W Statistic p-value
## 1 0.1365918 1.717617 0.262
## Alternative hypothesis: rho != 0

**Assumption of no multicollinearity**

vif(Mod3) #VIF

## fear_attach criticism gender.num age ethnicity.num
## 1.370324 1.315727 1.183165 1.252849 1.108592
## sex.num employ.num
## 1.291482 1.088164

1/vif(Mod3) #tolerance

## fear_attach criticism gender.num age ethnicity.num
## 0.7297543 0.7600361 0.8451906 0.7981808 0.9020453
## sex.num employ.num
## 0.7743041 0.9189790

mean(vif(Mod3))

## [1] 1.230043

**Check assumptions about the residuals**

plot(Mod3)


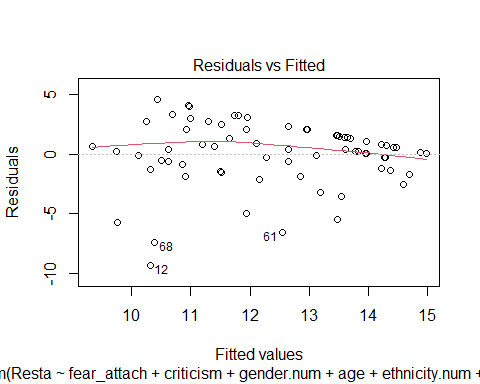

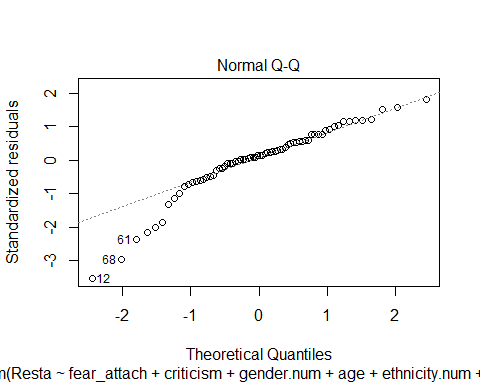

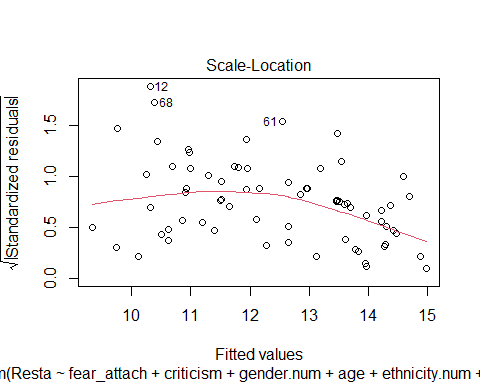

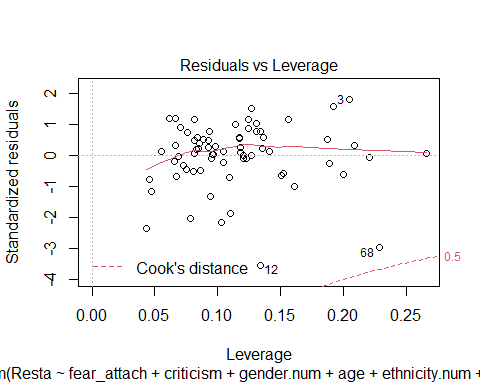


hist(H2_data$standardized.residuals)


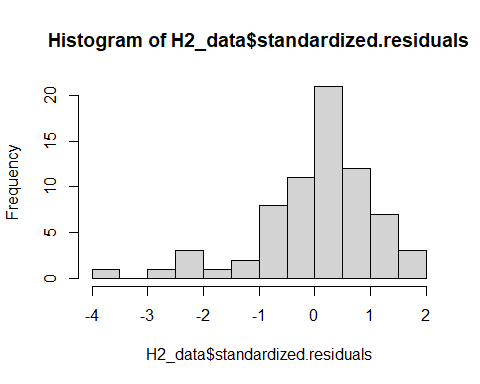


hist(H2_data$studentized.residuals)


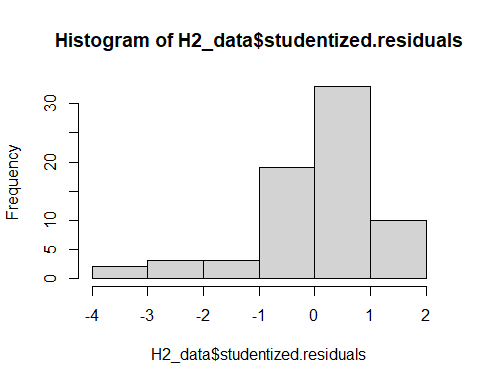

Supplement: Supplementary file 1 [file Data_Sheet_1.DOCX]
