# Supplementary material for: Being more satisfied with romantic relationship status is associated with increased mental wellbeing in people with experience of psychosis
Source: Front Psychiatry. 2023 Sep 28;14:1232973. doi: 10.3389/fpsyt.2023.1232973 (PMC10569177; doi:10.3389/fpsyt.2023.1232973)

Hypothesis 1

Rebecca White

19/01/2022

### Load packages and data set

library(readr)
library(tidyverse)

## -- Attaching packages --------------------------------------- tidyverse 1.3.0 --

## v ggplot2 3.3.2 v dplyr 1.0.2
## v tibble 3.0.4 v stringr 1.4.0
## v tidyr 1.1.2 v forcats 0.5.0
## v purrr 0.3.4

## -- Conflicts ------------------------------------------ tidyverse_conflicts() --
## x dplyr::filter() masks stats::filter()
## x dplyr::lag() masks stats::lag()

library(QuantPsyc)

## Loading required package: boot

## Loading required package: MASS

##
## Attaching package: 'MASS'

## The following object is masked from 'package:dplyr':
##
## select

##
## Attaching package: 'QuantPsyc'

## The following object is masked from 'package:base':
##
## norm

library(car)

## Loading required package: carData

##
## Attaching package: 'car'

## The following object is masked from 'package:boot':
##
## logit

## The following object is masked from 'package:dplyr':
##
## recode

## The following object is masked from 'package:purrr':
##
## some

Dataset_190_obs_2_9_21 <- read_csv("Z:/Online study IRAS ID 271957/Online analysis/Dataset_190_obs_2.9.21.csv")

## Warning: Missing column names filled in: 'X1' [1]

##
## -- Column specification --------------------------------------------------------
## cols(
## .default = col_double(),
## redcap_survey_identifier = col_logical(),
## pis_timestamp = col_datetime(format = ""),
## screening_questions_timestamp = col_datetime(format = ""),
## demographic_information_timestamp = col_datetime(format = ""),
## nationality = col_character(),
## ethnicity_other = col_character(),
## gender_self_describe = col_character(),
## sexual_orientation_selfdescribe = col_character(),
## rr_selfdescribe = col_character(),
## last_rr_end = col_character(),
## current_rr_length = col_character(),
## the_community_assessment_of_psychic_experiences_ca_timestamp = col_datetime(format = ""),
## the_short_warwick_mental_health_wellbeing_scale_timestamp = col_datetime(format = ""),
## adapted_satisfaction_with_relationships_scale_rest_timestamp = col_datetime(format = ""),
## three_item_loneliness_scale_timestamp = col_datetime(format = ""),
## internalised_stigma_of_mental_illness_inventory_10_timestamp = col_datetime(format = ""),
## multidimensional_scale_of_perceived_social_support_timestamp = col_datetime(format = ""),
## self_esteem_rating_scale_short_form_serssf_timestamp = col_datetime(format = ""),
## relationships_questionnaire_timestamp = col_datetime(format = ""),
## Screening_Qs_result = col_character()
## # ... with 7 more columns
## )
## i Use `spec()` for the full column specifications.

View(Dataset_190_obs_2_9_21)

### Create data frame to work from

data.frame1 <- data.frame(Dataset_190_obs_2_9_21$Resta.total,
 Dataset_190_obs_2_9_21$GenderF,
 Dataset_190_obs_2_9_21$age,
 Dataset_190_obs_2_9_21$EthnicityF,
 Dataset_190_obs_2_9_21$SexualityF,
 Dataset_190_obs_2_9_21$R_Status_simplified,
 Dataset_190_obs_2_9_21$rr_selfdescribe,
 Dataset_190_obs_2_9_21$EmploymentF,
 Dataset_190_obs_2_9_21$ethnicity_other)


#rename columns
names(data.frame1)[names(data.frame1) ==
 "Dataset_190_obs_2_9_21.SWEMWBS_metric"]<- "SWEMWBS"
names(data.frame1)[names(data.frame1) ==
 "Dataset_190_obs_2_9_21.Resta.total"]<- "Resta"
names(data.frame1)[names(data.frame1) ==
 "Dataset_190_obs_2_9_21.GenderF"]<- "gender"
names(data.frame1)[names(data.frame1) ==
 "Dataset_190_obs_2_9_21.age"]<- "age"
names(data.frame1)[names(data.frame1) ==
 "Dataset_190_obs_2_9_21.EthnicityF"]<- "ethnicity"
names(data.frame1)[names(data.frame1) ==
 "Dataset_190_obs_2_9_21.SexualityF"]<- "sexuality"
names(data.frame1)[names(data.frame1) ==
 "Dataset_190_obs_2_9_21.R_Status_simplified"]<- "relationship.status"
names(data.frame1)[names(data.frame1) ==
 "Dataset_190_obs_2_9_21.EmploymentF"]<- "employment"

#Add in fearful attachment
data.frame1$fear_attach <- Dataset_190_obs_2_9_21$fearful_rating

#CREATE DICOTOMOUS VARIABLES
#GENDER
#Gender is categorised as 1= female, 2 = male, 3 = prefer not to say, 4 = prefer to self describe
table(data.frame1$gender)

##
## female male Self_describe
## 105 69 6

#remove 'prefer not to say & self describe

data.frame1$gender <- factor(data.frame1$gender,
 c("female","male"), c("female", "male"))
table(data.frame1$gender)

##
## female male
## 105 69

#ETHNICITY
#white british/irish/any other white background vs PGM
table(data.frame1$ethnicity)

##
## asian black mixed other white
## 10 11 5 5 148

data.frame1 %>%
 mutate(ethnicity.dicotomised = case_when(ethnicity == "other" & Dataset_190_obs_2_9_21.ethnicity_other == "White Scottish" ~ "white",
 ethnicity == "white" ~ "white",
 ethnicity == "mixed" ~ "PGM",
 ethnicity == "asian" ~ "PGM",
 ethnicity == "black" ~ "PGM",
 ethnicity == "chinese" ~ "PGM",
 ethnicity == "other" ~ "PGM")) -> data.frame1

table(data.frame1$ethnicity.dicotomised)

##
## PGM white
## 29 150

#SEXUALITY
table(data.frame1$sexuality)

##
## bisexual gay/lesbian heterosexual prefer not to say
## 24 9 132 6
## self-describe
## 9

#checked what participants have written for self-describe sexuality & happy to put all in non-heterosexual group

data.frame1 %>%
 mutate(sexuality.dicotomised = case_when(sexuality == "heterosexual" ~ "heterosexual",
 sexuality == "bisexual" ~ "LGBQ+",
 sexuality == "gay/lesbian" ~ "LGBQ+",
 sexuality == "self-describe" ~ "LGBQ+")) -> data.frame1
table(data.frame1$sexuality.dicotomised)

##
## heterosexual LGBQ+
## 132 42

#RELATIONSHIP STATUS, single vs partner
table(data.frame1$relationship.status)

##
## dating partner self describe separated single
## 5 78 5 2 89

data.frame1 %>%
 mutate(relationship.dicotomised = case_when(relationship.status == "single" ~ "single",
 relationship.status == "dating" ~ "single",
 relationship.status == "separated" ~ "single",
 relationship.status == "widowed" ~ "single",
 relationship.status == "partner" ~ "partner",
 relationship.status == "self describe" & Dataset_190_obs_2_9_21.rr_selfdescribe == "Living with a wife, queerplatonic partner, and steady, with one long-distance relationship as well" ~ "partner",
 relationship.status == "self describe" ~ "single")) -> data.frame1

table(data.frame1$relationship.dicotomised)

##
## partner single
## 79 100

#EMPLOYMENT , work/education vs not
table(data.frame1$employment)

##
## employee FT education
## 56 21
## looking after home/family rec. sickness/disability benefits
## 6 58
## retired self-employed
## 5 5
## unemployed
## 26

data.frame1 %>%
 mutate(employment.dicotomised = case_when ( employment == "employee" ~ "working/FT.education",
 employment == "self-employed" ~ "working/FT.education",
 employment == "FT education" ~ "working/FT.education",
 employment == "unemployed" ~ "unemployed",
 employment == "looking after home/family" ~ "working/FT.education",
 employment == "rec. sickness/disability benefits" ~ "unemployed",
 employment == "retired" ~ "unemployed")) -> data.frame1

table(data.frame1$employment.dicotomised)

##
## unemployed working/FT.education
## 89 88

#remove columns that are mainly NA e.g. rr_self describe so they don't interfere with next stages
drops <- c("Dataset_190_obs_2_9_21.rr_selfdescribe", "Dataset_190_obs_2_9_21.ethnicity_other")
data.frame1[ , !(names(data.frame1) %in% drops)] -> data.frame1


#Create dummy variables
#gender


Female0_v_Male1 <- c(0, 1)
contrasts(data.frame1$gender) <- cbind(Female0_v_Male1)


#convert new dictomised variables to factors
names <- c('ethnicity.dicotomised', 'sexuality.dicotomised', 'relationship.dicotomised', 'employment.dicotomised')
data.frame1[,names]<- lapply(data.frame1[,names],factor)
str(data.frame1)

## 'data.frame': 190 obs. of 12 variables:
## $ Resta : num 15 14 15 13 10 10 14 13 15 14 ...
## $ gender : Factor w/ 2 levels "female","male": 1 1 1 1 1 2 2 1 1 1 ...
## ..- attr(*, "contrasts")= num [1:2, 1] 0 1
## .. ..- attr(*, "dimnames")=List of 2
## .. .. ..$ : chr [1:2] "female" "male"
## .. .. ..$ : chr "Female0_v_Male1"
## $ age : num 32 22 31 27 45 50 25 32 25 39 ...
## $ ethnicity : chr "white" "white" "mixed" "white" ...
## $ sexuality : chr "heterosexual" "bisexual" "heterosexual" "heterosexual" ...
## $ relationship.status : chr "partner" "partner" "partner" "partner" ...
## $ employment : chr "FT education" "employee" "employee" "FT education" ...
## $ fear_attach : num 2 6 5 5 7 5 6 7 2 5 ...
## $ ethnicity.dicotomised : Factor w/ 2 levels "PGM","white": 2 2 1 2 2 1 2 2 NA 2 ...
## $ sexuality.dicotomised : Factor w/ 2 levels "heterosexual",..: 1 2 1 1 1 1 NA 1 NA 1 ...
## $ relationship.dicotomised: Factor w/ 2 levels "partner","single": 1 1 1 1 1 1 1 1 1 1 ...
## $ employment.dicotomised : Factor w/ 2 levels "unemployed","working/FT.education": 2 2 2 2 2 2 1 1 2 2 ...

#ethnicity
White0_v_PGM1 <- c(1, 0)
contrasts(data.frame1$ethnicity.dicotomised) <- cbind(White0_v_PGM1)


#sexuality
heter0_v_LGBQ1 <- c(0, 1)
contrasts(data.frame1$sexuality.dicotomised) <- cbind(heter0_v_LGBQ1)


#relationship status
single0_v_partner1 <- c(1,0)
contrasts(data.frame1$relationship.dicotomised) <- cbind(single0_v_partner1)


#employment status
unemploy.0_v_work1 <- c(0,1)
contrasts(data.frame1$employment.dicotomised) <- cbind(unemploy.0_v_work1)


## convert dichotomous variables into numeric values -
## this will allow for beta scores to be generated

#gender
data.frame1 %>%
 mutate (gender.num = case_when ( gender == "male" ~ 1,
 gender == "female" ~ 0)) -> data.frame1

#relationship status
data.frame1 %>%
 mutate (rel.num = case_when (relationship.dicotomised == "single" ~ 0,
 relationship.dicotomised == "partner" ~ 1)) -> data.frame1

#ethnicity
data.frame1 %>%
 mutate (ethnicity.num = case_when ( ethnicity.dicotomised == "white" ~ 0,
 ethnicity.dicotomised == "PGM" ~ 1)) -> data.frame1

#sexuality
data.frame1 %>%
 mutate (sex.num = case_when ( sexuality.dicotomised == "heterosexual" ~ 0,
 sexuality.dicotomised == "LGBQ+" ~ 1)) -> data.frame1

#employment
data.frame1 %>%
 mutate (employ.num = case_when ( employment.dicotomised == "unemployed" ~ 0,
 employment.dicotomised == "working/FT.education" ~ 1)) -> data.frame1


#Remove missing data
data.frame1 <- na.omit(data.frame1)
table(data.frame1$nmiss)

## < table of extent 0 >

### Build model

**Step 1**

Mod1 <- lm(Resta ~ rel.num, data = data.frame1)
summary(Mod1)

##
## Call:
## lm(formula = Resta ~ rel.num, data = data.frame1)
##
## Residuals:
## Min 1Q Median 3Q Max
## -11.4143 -2.6410 0.5857 2.5857 7.3590
##
## Coefficients:
## Estimate Std. Error t value Pr(>|t|)
## (Intercept) 7.6410 0.4477 17.066 < 2e-16 ***
## rel.num 4.7733 0.6510 7.332 1.44e-11 ***
## ---
## Signif. codes: 0 '***' 0.001 '**' 0.01 '*' 0.05 '.' 0.1 ' ' 1
##
## Residual standard error: 3.954 on 146 degrees of freedom
## Multiple R-squared: 0.2691, Adjusted R-squared: 0.2641
## F-statistic: 53.76 on 1 and 146 DF, p-value: 1.438e-11

lm.beta(Mod1)

## rel.num
## 0.5187537

**Step 2**

Mod2 <- lm(Resta ~ rel.num + fear_attach, data = data.frame1)
summary(Mod2)

##
## Call:
## lm(formula = Resta ~ rel.num + fear_attach, data = data.frame1)
##
## Residuals:
## Min 1Q Median 3Q Max
## -10.3111 -2.1076 0.6126 2.4755 8.3840
##
## Coefficients:
## Estimate Std. Error t value Pr(>|t|)
## (Intercept) 9.7959 0.8680 11.286 < 2e-16 ***
## rel.num 4.6951 0.6360 7.382 1.12e-11 ***
## fear_attach -0.4543 0.1581 -2.873 0.00467 **
## ---
## Signif. codes: 0 '***' 0.001 '**' 0.01 '*' 0.05 '.' 0.1 ' ' 1
##
## Residual standard error: 3.86 on 145 degrees of freedom
## Multiple R-squared: 0.3085, Adjusted R-squared: 0.2989
## F-statistic: 32.34 on 2 and 145 DF, p-value: 2.431e-12

lm.beta(Mod2)

## rel.num fear_attach
## 0.5102542 -0.1986187

**Step 3**

Mod3<- lm(Resta ~ rel.num + fear_attach + gender.num + age + ethnicity.num +
 sex.num + employ.num, data = data.frame1)

summary(Mod3)

##
## Call:
## lm(formula = Resta ~ rel.num + fear_attach + gender.num + age +
## ethnicity.num + sex.num + employ.num, data = data.frame1)
##
## Residuals:
## Min 1Q Median 3Q Max
## -10.3710 -2.4907 0.5013 2.5076 8.1135
##
## Coefficients:
## Estimate Std. Error t value Pr(>|t|)
## (Intercept) 10.745408 1.650131 6.512 1.24e-09 ***
## rel.num 4.411692 0.692647 6.369 2.55e-09 ***
## fear_attach -0.566214 0.165042 -3.431 0.000791 ***
## gender.num -1.598520 0.670011 -2.386 0.018380 *
## age 0.003524 0.028648 0.123 0.902276
## ethnicity.num 0.299083 0.867549 0.345 0.730804
## sex.num 0.857275 0.866964 0.989 0.324455
## employ.num 0.037660 0.713948 0.053 0.958007
## ---
## Signif. codes: 0 '***' 0.001 '**' 0.01 '*' 0.05 '.' 0.1 ' ' 1
##
## Residual standard error: 3.824 on 140 degrees of freedom
## Multiple R-squared: 0.3447, Adjusted R-squared: 0.3119
## F-statistic: 10.52 on 7 and 140 DF, p-value: 1.384e-10

lm.beta(Mod3)

## rel.num fear_attach gender.num age ethnicity.num
## 0.479458770 -0.247564312 -0.171677367 0.009500745 0.024392914
## sex.num employ.num
## 0.075019081 0.004098487

### Compare models

anova(Mod1, Mod2)

## Analysis of Variance Table
##
## Model 1: Resta ~ rel.num
## Model 2: Resta ~ rel.num + fear_attach
## Res.Df RSS Df Sum of Sq F Pr(>F)
## 1 146 2282.9
## 2 145 2159.9 1 122.99 8.2567 0.004671 **
## ---
## Signif. codes: 0 '***' 0.001 '**' 0.01 '*' 0.05 '.' 0.1 ' ' 1

anova(Mod2, Mod3)

## Analysis of Variance Table
##
## Model 1: Resta ~ rel.num + fear_attach
## Model 2: Resta ~ rel.num + fear_attach + gender.num + age + ethnicity.num +
## sex.num + employ.num
## Res.Df RSS Df Sum of Sq F Pr(>F)
## 1 145 2159.9
## 2 140 2046.8 5 113.15 1.5479 0.1789

Step 2 significantly increases the prediction of ReSta scores compared to Step 1. However Step 3 did not result in a further overall improvement in the prediction of ReSta scores.

### Check assumptions

**Outliers and influential cases**

data.frame1$residuals <- resid(Mod3)
data.frame1$standardized.residuals <- rstandard(Mod3)
data.frame1$studentized.residuals <- rstudent(Mod3)
data.frame1$cooks.distance <- cooks.distance(Mod3)
data.frame1$dfbeta <- dfbeta(Mod3)
data.frame1$dffit <- dffits(Mod3)
data.frame1$leverage <- hatvalues(Mod3)
data.frame1$covariance <- covratio(Mod3)

data.frame1$standardized.residuals > 2 | data.frame1$standardized.residuals < -2

## [1] FALSE FALSE FALSE FALSE FALSE FALSE FALSE FALSE FALSE FALSE FALSE FALSE
## [13] FALSE FALSE FALSE FALSE TRUE FALSE FALSE FALSE FALSE FALSE FALSE FALSE
## [25] FALSE FALSE FALSE FALSE FALSE FALSE FALSE FALSE FALSE FALSE FALSE FALSE
## [37] FALSE FALSE FALSE FALSE FALSE FALSE FALSE FALSE FALSE FALSE FALSE FALSE
## [49] FALSE FALSE FALSE FALSE FALSE FALSE FALSE FALSE FALSE FALSE FALSE FALSE
## [61] FALSE FALSE FALSE FALSE FALSE FALSE FALSE FALSE FALSE FALSE FALSE FALSE
## [73] TRUE FALSE FALSE FALSE FALSE FALSE TRUE FALSE FALSE FALSE FALSE FALSE
## [85] FALSE FALSE FALSE FALSE FALSE FALSE FALSE TRUE FALSE FALSE FALSE FALSE
## [97] FALSE FALSE FALSE FALSE FALSE FALSE FALSE FALSE FALSE FALSE FALSE FALSE
## [109] FALSE FALSE FALSE FALSE FALSE FALSE FALSE FALSE FALSE TRUE FALSE FALSE
## [121] FALSE FALSE FALSE FALSE TRUE FALSE FALSE FALSE FALSE FALSE FALSE FALSE
## [133] FALSE FALSE FALSE FALSE FALSE FALSE FALSE FALSE FALSE TRUE FALSE FALSE
## [145] TRUE FALSE FALSE FALSE

# would expect 95% to be within this range (so approx 141/148)

data.frame1$large.residual <- data.frame1$standardized.residuals > 2 | data.frame1$standardized.residuals < -2
sum(data.frame1$large.residual) # 8 cases had a large residual

## [1] 8

data.frame1[data.frame1$large.residual, c("standardized.residuals" )]

## [1] -2.366889 -2.070354 -2.247146 -2.086783 -2.255856 -2.018236 2.188953
## [8] -2.831831

One has standardised residual +/- 2.5, None have a standardised residual larger than +/- 3

Look at leverage and cooks distance for these participants

data.frame1[data.frame1$large.residual, c("cooks.distance", "leverage", "covariance" )]

## cooks.distance leverage covariance
## 21 0.04521801 0.06065553 0.8131953
## 87 0.02580396 0.04594723 0.8655597
## 94 0.04382926 0.06492863 0.8441673
## 107 0.04768305 0.08054349 0.8945184
## 145 0.01712215 0.02621141 0.8087211
## 153 0.02369553 0.04446900 0.8751362
## 173 0.03842283 0.06028414 0.8529363
## 179 0.09023586 0.08258487 0.7201120

Cooks distance < 1 so this participant’s data is not having an undue influence on the model

Leverage can be calculated as 0.05 (k+1/n = 8/148), where k = the number of predictors in the model. None have leverage values are twice as large as this (0.11)

Covariance 1 - [3(k+1) / n] -> 0.84 1 + [3(k+1) / n] -> 0.16

73, 92 125, 142 have Covariance values outside these boundries. However, given Cooks Distance not overly concerned about these.

**Check assumption of independence**

dwt(Mod3)

## lag Autocorrelation D-W Statistic p-value
## 1 -0.05107342 2.098488 0.556
## Alternative hypothesis: rho != 0

**Assumption of no multicollinearity**

vif(Mod3) #VIF

## rel.num fear_attach gender.num age ethnicity.num
## 1.210628 1.112499 1.106236 1.274486 1.069614
## sex.num employ.num
## 1.229701 1.289765

1/vif(Mod3) #tolerance

## rel.num fear_attach gender.num age ethnicity.num
## 0.8260175 0.8988769 0.9039661 0.7846299 0.9349170
## sex.num employ.num
## 0.8132059 0.7753350

mean(vif(Mod3))

## [1] 1.184704

**Check assumptions about the residuals**

plot(Mod3)


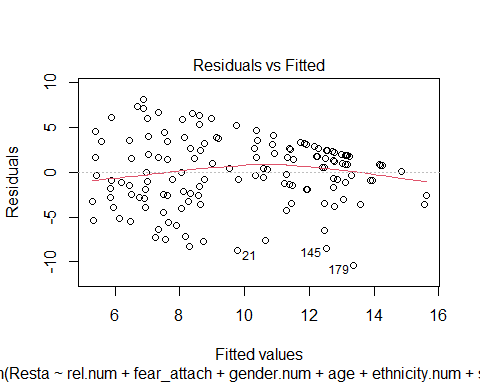

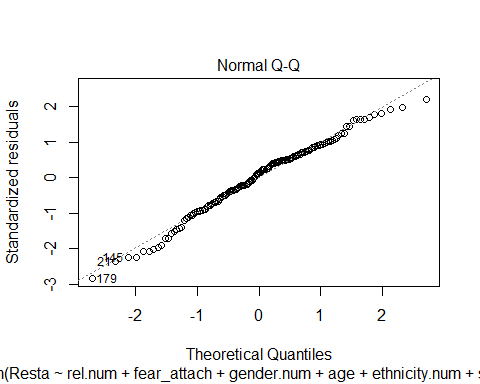

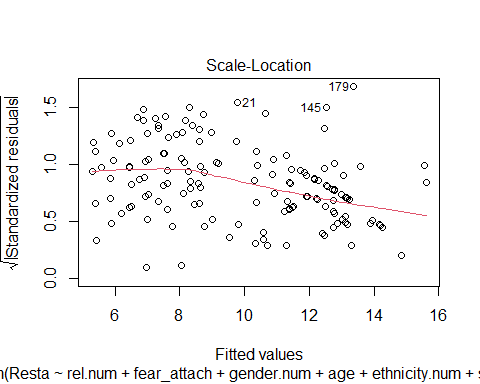

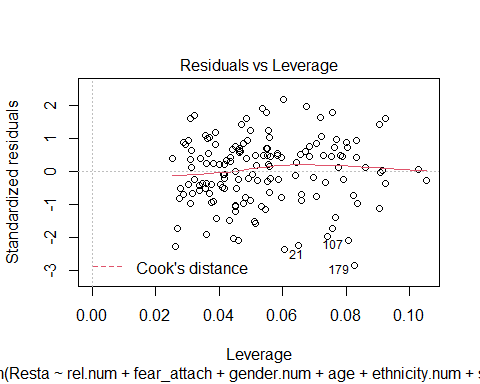


hist(data.frame1$standardized.residuals)


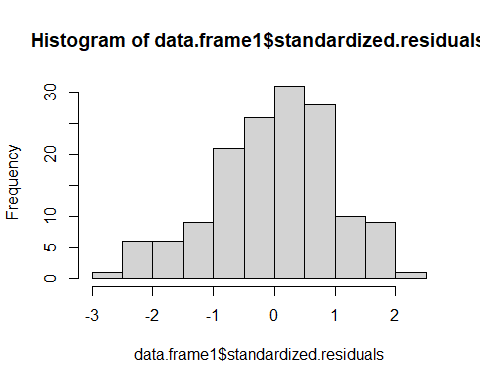


hist(data.frame1$studentized.residuals)


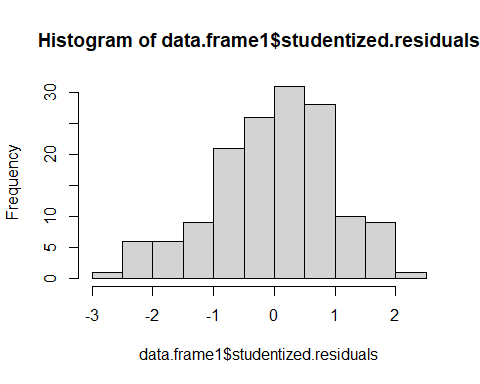

Supplement: Supplementary file 2 [file Data_Sheet_2.DOCX]
