# Supplementary material for: Being more satisfied with romantic relationship status is associated with increased mental wellbeing in people with experience of psychosis
Source: Front Psychiatry. 2023 Sep 28;14:1232973. doi: 10.3389/fpsyt.2023.1232973 (PMC10569177; doi:10.3389/fpsyt.2023.1232973)
Supplement: Supplementary file 3 [file Data_Sheet_3.DOCX]

Correlations

Rebecca White

22/12/2021

Load packages and dataset

library(readr)
library(tidyverse)

## -- Attaching packages --------------------------------------- tidyverse 1.3.0 --

## v ggplot2 3.3.2 v dplyr 1.0.2
## v tibble 3.0.4 v stringr 1.4.0
## v tidyr 1.1.2 v forcats 0.5.0
## v purrr 0.3.4

## -- Conflicts ------------------------------------------ tidyverse_conflicts() --
## x dplyr::filter() masks stats::filter()
## x dplyr::lag() masks stats::lag()

Dataset_190_obs_2_9_21 <- read_csv("Z:/Online study IRAS ID 271957/Online analysis/Dataset_190_obs_2.9.21.csv")

## Warning: Missing column names filled in: 'X1' [1]

##
## -- Column specification --------------------------------------------------------
## cols(
## .default = col_double(),
## redcap_survey_identifier = col_logical(),
## pis_timestamp = col_datetime(format = ""),
## screening_questions_timestamp = col_datetime(format = ""),
## demographic_information_timestamp = col_datetime(format = ""),
## nationality = col_character(),
## ethnicity_other = col_character(),
## gender_self_describe = col_character(),
## sexual_orientation_selfdescribe = col_character(),
## rr_selfdescribe = col_character(),
## last_rr_end = col_character(),
## current_rr_length = col_character(),
## the_community_assessment_of_psychic_experiences_ca_timestamp = col_datetime(format = ""),
## the_short_warwick_mental_health_wellbeing_scale_timestamp = col_datetime(format = ""),
## adapted_satisfaction_with_relationships_scale_rest_timestamp = col_datetime(format = ""),
## three_item_loneliness_scale_timestamp = col_datetime(format = ""),
## internalised_stigma_of_mental_illness_inventory_10_timestamp = col_datetime(format = ""),
## multidimensional_scale_of_perceived_social_support_timestamp = col_datetime(format = ""),
## self_esteem_rating_scale_short_form_serssf_timestamp = col_datetime(format = ""),
## relationships_questionnaire_timestamp = col_datetime(format = ""),
## Screening_Qs_result = col_character()
## # ... with 7 more columns
## )
## i Use `spec()` for the full column specifications.

View(Dataset_190_obs_2_9_21)

Create new dataframe and remove ‘Inf’ values from the ISMI column

ismi.df <- Dataset_190_obs_2_9_21

ismi.df %>%
 filter (! (ISMI_total =="Inf")) -> ismi.df

**ReSta** Check distribution and then calculate correlations between ReSta and other variables

H3 predicts that ReSta will be positively correlated with SWEMWBS and negatively correlated with CAPE subscales

hist(Dataset_190_obs_2_9_21$Resta.total)


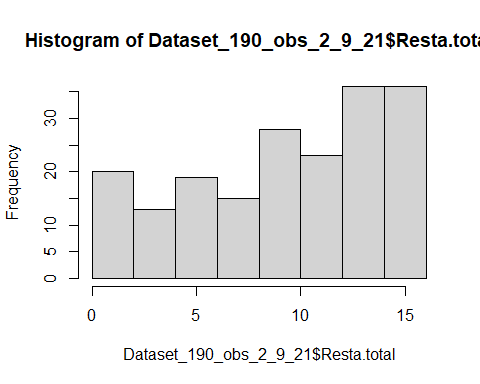


shapiro.test(Dataset_190_obs_2_9_21$Resta.total)

##
## Shapiro-Wilk normality test
##
## data: Dataset_190_obs_2_9_21$Resta.total
## W = 0.89823, p-value = 4.125e-10

#SWEMWBS
cor.test(Dataset_190_obs_2_9_21$SWEMWBS_metric, Dataset_190_obs_2_9_21$Resta.total,
 method = "spearman",
 alternative = "greater")

## Warning in cor.test.default(Dataset_190_obs_2_9_21$SWEMWBS_metric,
## Dataset_190_obs_2_9_21$Resta.total, : Cannot compute exact p-value with ties

##
## Spearman's rank correlation rho
##
## data: Dataset_190_obs_2_9_21$SWEMWBS_metric and Dataset_190_obs_2_9_21$Resta.total
## S = 805655, p-value = 3.748e-05
## alternative hypothesis: true rho is greater than 0
## sample estimates:
## rho
## 0.2839764

#CAPE subscales
cor.test(Dataset_190_obs_2_9_21$CAPE_negative, Dataset_190_obs_2_9_21$Resta.total,
 method = "spearman", alternative = "less" )

## Warning in cor.test.default(Dataset_190_obs_2_9_21$CAPE_negative,
## Dataset_190_obs_2_9_21$Resta.total, : Cannot compute exact p-value with ties

##
## Spearman's rank correlation rho
##
## data: Dataset_190_obs_2_9_21$CAPE_negative and Dataset_190_obs_2_9_21$Resta.total
## S = 1262694, p-value = 0.07549
## alternative hypothesis: true rho is less than 0
## sample estimates:
## rho
## -0.1045887

cor.test(Dataset_190_obs_2_9_21$CAPE_positive, Dataset_190_obs_2_9_21$Resta.total,
 method = "spearman", alternative = "less" )

## Warning in cor.test.default(Dataset_190_obs_2_9_21$CAPE_positive,
## Dataset_190_obs_2_9_21$Resta.total, : Cannot compute exact p-value with ties

##
## Spearman's rank correlation rho
##
## data: Dataset_190_obs_2_9_21$CAPE_positive and Dataset_190_obs_2_9_21$Resta.total
## S = 1196945, p-value = 0.2595
## alternative hypothesis: true rho is less than 0
## sample estimates:
## rho
## -0.04707196

cor.test(Dataset_190_obs_2_9_21$CAPE_depressive, Dataset_190_obs_2_9_21$Resta.total,
 method = "spearman", alternative = "less" )

## Warning in cor.test.default(Dataset_190_obs_2_9_21$CAPE_depressive,
## Dataset_190_obs_2_9_21$Resta.total, : Cannot compute exact p-value with ties

##
## Spearman's rank correlation rho
##
## data: Dataset_190_obs_2_9_21$CAPE_depressive and Dataset_190_obs_2_9_21$Resta.total
## S = 1349497, p-value = 0.006344
## alternative hypothesis: true rho is less than 0
## sample estimates:
## rho
## -0.1805225

#Loneliness
cor.test(Dataset_190_obs_2_9_21$LonelinessTotal, Dataset_190_obs_2_9_21$Resta.total,
 method = "spearman")

## Warning in cor.test.default(Dataset_190_obs_2_9_21$LonelinessTotal,
## Dataset_190_obs_2_9_21$Resta.total, : Cannot compute exact p-value with ties

##
## Spearman's rank correlation rho
##
## data: Dataset_190_obs_2_9_21$LonelinessTotal and Dataset_190_obs_2_9_21$Resta.total
## S = 1606658, p-value < 2.2e-16
## alternative hypothesis: true rho is not equal to 0
## sample estimates:
## rho
## -0.5730204

#Internalised stigma
cor.test(ismi.df$ISMI_total, ismi.df$Resta.total,
 method = "spearman")

## Warning in cor.test.default(ismi.df$ISMI_total, ismi.df$Resta.total, method =
## "spearman"): Cannot compute exact p-value with ties

##
## Spearman's rank correlation rho
##
## data: ismi.df$ISMI_total and ismi.df$Resta.total
## S = 1308864, p-value = 0.005815
## alternative hypothesis: true rho is not equal to 0
## sample estimates:
## rho
## -0.2009731

#Self-esteem
cor.test(Dataset_190_obs_2_9_21$SERS_total, Dataset_190_obs_2_9_21$Resta.total,
 method = "spearman")

## Warning in cor.test.default(Dataset_190_obs_2_9_21$SERS_total,
## Dataset_190_obs_2_9_21$Resta.total, : Cannot compute exact p-value with ties

##
## Spearman's rank correlation rho
##
## data: Dataset_190_obs_2_9_21$SERS_total and Dataset_190_obs_2_9_21$Resta.total
## S = 741920, p-value = 0.001379
## alternative hypothesis: true rho is not equal to 0
## sample estimates:
## rho
## 0.2366844

#Social support
cor.test(Dataset_190_obs_2_9_21$mspss_ff, Dataset_190_obs_2_9_21$Resta.total,
 method = "spearman")

## Warning in cor.test.default(Dataset_190_obs_2_9_21$mspss_ff,
## Dataset_190_obs_2_9_21$Resta.total, : Cannot compute exact p-value with ties

##
## Spearman's rank correlation rho
##
## data: Dataset_190_obs_2_9_21$mspss_ff and Dataset_190_obs_2_9_21$Resta.total
## S = 803025, p-value = 0.01152
## alternative hypothesis: true rho is not equal to 0
## sample estimates:
## rho
## 0.1874356

#Attachment
cor.test(Dataset_190_obs_2_9_21$secure_rating, Dataset_190_obs_2_9_21$Resta.total,
 method = "spearman")

## Warning in cor.test.default(Dataset_190_obs_2_9_21$secure_rating,
## Dataset_190_obs_2_9_21$Resta.total, : Cannot compute exact p-value with ties

##
## Spearman's rank correlation rho
##
## data: Dataset_190_obs_2_9_21$secure_rating and Dataset_190_obs_2_9_21$Resta.total
## S = 740017, p-value = 0.09586
## alternative hypothesis: true rho is not equal to 0
## sample estimates:
## rho
## 0.1273859

cor.test(Dataset_190_obs_2_9_21$fearful_rating, Dataset_190_obs_2_9_21$Resta.total,
 method = "spearman")

## Warning in cor.test.default(Dataset_190_obs_2_9_21$fearful_rating,
## Dataset_190_obs_2_9_21$Resta.total, : Cannot compute exact p-value with ties

##
## Spearman's rank correlation rho
##
## data: Dataset_190_obs_2_9_21$fearful_rating and Dataset_190_obs_2_9_21$Resta.total
## S = 978927, p-value = 0.02229
## alternative hypothesis: true rho is not equal to 0
## sample estimates:
## rho
## -0.174703

cor.test(Dataset_190_obs_2_9_21$preocc_rating, Dataset_190_obs_2_9_21$Resta.total,
 method = "spearman")

## Warning in cor.test.default(Dataset_190_obs_2_9_21$preocc_rating,
## Dataset_190_obs_2_9_21$Resta.total, : Cannot compute exact p-value with ties

##
## Spearman's rank correlation rho
##
## data: Dataset_190_obs_2_9_21$preocc_rating and Dataset_190_obs_2_9_21$Resta.total
## S = 942326, p-value = 0.04957
## alternative hypothesis: true rho is not equal to 0
## sample estimates:
## rho
## -0.1508555

cor.test(Dataset_190_obs_2_9_21$dismiss_rating, Dataset_190_obs_2_9_21$Resta.total,
 method = "spearman")

## Warning in cor.test.default(Dataset_190_obs_2_9_21$dismiss_rating,
## Dataset_190_obs_2_9_21$Resta.total, : Cannot compute exact p-value with ties

##
## Spearman's rank correlation rho
##
## data: Dataset_190_obs_2_9_21$dismiss_rating and Dataset_190_obs_2_9_21$Resta.total
## S = 763750, p-value = 0.6662
## alternative hypothesis: true rho is not equal to 0
## sample estimates:
## rho
## 0.03352681

**SWEMWBS** Check distribution and then calculate correlations between SWEMWBS and other variables

hist(Dataset_190_obs_2_9_21$SWEMWBS_metric)


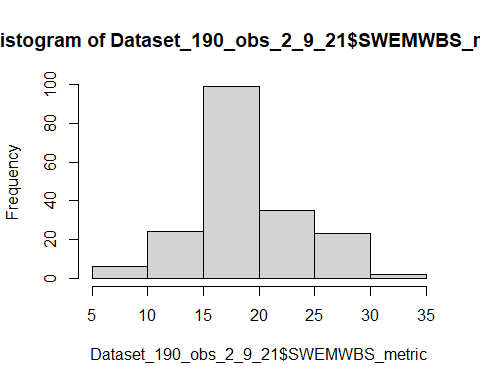


shapiro.test(Dataset_190_obs_2_9_21$SWEMWBS_metric)

##
## Shapiro-Wilk normality test
##
## data: Dataset_190_obs_2_9_21$SWEMWBS_metric
## W = 0.97358, p-value = 0.001206

#CAPE subscales
cor.test(Dataset_190_obs_2_9_21$SWEMWBS_metric,Dataset_190_obs_2_9_21$CAPE_positive,
 method = "spearman")

## Warning in cor.test.default(Dataset_190_obs_2_9_21$SWEMWBS_metric,
## Dataset_190_obs_2_9_21$CAPE_positive, : Cannot compute exact p-value with ties

##
## Spearman's rank correlation rho
##
## data: Dataset_190_obs_2_9_21$SWEMWBS_metric and Dataset_190_obs_2_9_21$CAPE_positive
## S = 1681164, p-value = 4.986e-13
## alternative hypothesis: true rho is not equal to 0
## sample estimates:
## rho
## -0.4941293

cor.test(Dataset_190_obs_2_9_21$SWEMWBS_metric,Dataset_190_obs_2_9_21$CAPE_negative,
 method = "spearman")

## Warning in cor.test.default(Dataset_190_obs_2_9_21$SWEMWBS_metric,
## Dataset_190_obs_2_9_21$CAPE_negative, : Cannot compute exact p-value with ties

##
## Spearman's rank correlation rho
##
## data: Dataset_190_obs_2_9_21$SWEMWBS_metric and Dataset_190_obs_2_9_21$CAPE_negative
## S = 1843800, p-value < 2.2e-16
## alternative hypothesis: true rho is not equal to 0
## sample estimates:
## rho
## -0.638671

cor.test(Dataset_190_obs_2_9_21$SWEMWBS_metric, Dataset_190_obs_2_9_21$CAPE_depressive, method = "spearman")

## Warning in cor.test.default(Dataset_190_obs_2_9_21$SWEMWBS_metric,
## Dataset_190_obs_2_9_21$CAPE_depressive, : Cannot compute exact p-value with ties

##
## Spearman's rank correlation rho
##
## data: Dataset_190_obs_2_9_21$SWEMWBS_metric and Dataset_190_obs_2_9_21$CAPE_depressive
## S = 1969043, p-value < 2.2e-16
## alternative hypothesis: true rho is not equal to 0
## sample estimates:
## rho
## -0.7499807

#Loneliness
cor.test(Dataset_190_obs_2_9_21$SWEMWBS_metric, Dataset_190_obs_2_9_21$LonelinessTotal, method = "spearman")

## Warning in cor.test.default(Dataset_190_obs_2_9_21$SWEMWBS_metric,
## Dataset_190_obs_2_9_21$LonelinessTotal, : Cannot compute exact p-value with ties

##
## Spearman's rank correlation rho
##
## data: Dataset_190_obs_2_9_21$SWEMWBS_metric and Dataset_190_obs_2_9_21$LonelinessTotal
## S = 1569965, p-value = 4.573e-15
## alternative hypothesis: true rho is not equal to 0
## sample estimates:
## rho
## -0.5370955

#Internalised stigma
cor.test(ismi.df$SWEMWBS_metric, ismi.df$ISMI_total, method = "spearman")

## Warning in cor.test.default(ismi.df$SWEMWBS_metric, ismi.df$ISMI_total, : Cannot
## compute exact p-value with ties

##
## Spearman's rank correlation rho
##
## data: ismi.df$SWEMWBS_metric and ismi.df$ISMI_total
## S = 1717546, p-value < 2.2e-16
## alternative hypothesis: true rho is not equal to 0
## sample estimates:
## rho
## -0.6015234

#Self-esteem
cor.test(Dataset_190_obs_2_9_21$SWEMWBS_metric, Dataset_190_obs_2_9_21$SERS_total,
 method = "spearman")

## Warning in cor.test.default(Dataset_190_obs_2_9_21$SWEMWBS_metric,
## Dataset_190_obs_2_9_21$SERS_total, : Cannot compute exact p-value with ties

##
## Spearman's rank correlation rho
##
## data: Dataset_190_obs_2_9_21$SWEMWBS_metric and Dataset_190_obs_2_9_21$SERS_total
## S = 201711, p-value < 2.2e-16
## alternative hypothesis: true rho is not equal to 0
## sample estimates:
## rho
## 0.7889742

#Social support
cor.test(Dataset_190_obs_2_9_21$SWEMWBS_metric, Dataset_190_obs_2_9_21$mspss_ff,
 method = "spearman")

## Warning in cor.test.default(Dataset_190_obs_2_9_21$SWEMWBS_metric,
## Dataset_190_obs_2_9_21$mspss_ff, : Cannot compute exact p-value with ties

##
## Spearman's rank correlation rho
##
## data: Dataset_190_obs_2_9_21$SWEMWBS_metric and Dataset_190_obs_2_9_21$mspss_ff
## S = 571647, p-value = 9.23e-09
## alternative hypothesis: true rho is not equal to 0
## sample estimates:
## rho
## 0.411868

#Attachment
cor.test(Dataset_190_obs_2_9_21$SWEMWBS_metric, Dataset_190_obs_2_9_21$secure_rating, method = "spearman")

## Warning in cor.test.default(Dataset_190_obs_2_9_21$SWEMWBS_metric,
## Dataset_190_obs_2_9_21$secure_rating, : Cannot compute exact p-value with ties

##
## Spearman's rank correlation rho
##
## data: Dataset_190_obs_2_9_21$SWEMWBS_metric and Dataset_190_obs_2_9_21$secure_rating
## S = 492494, p-value = 2.789e-08
## alternative hypothesis: true rho is not equal to 0
## sample estimates:
## rho
## 0.4090124

cor.test(Dataset_190_obs_2_9_21$SWEMWBS_metric, Dataset_190_obs_2_9_21$fearful_rating, method = "spearman")

## Warning in cor.test.default(Dataset_190_obs_2_9_21$SWEMWBS_metric,
## Dataset_190_obs_2_9_21$fearful_rating, : Cannot compute exact p-value with ties

##
## Spearman's rank correlation rho
##
## data: Dataset_190_obs_2_9_21$SWEMWBS_metric and Dataset_190_obs_2_9_21$fearful_rating
## S = 1152696, p-value = 3.402e-08
## alternative hypothesis: true rho is not equal to 0
## sample estimates:
## rho
## -0.4077783

cor.test(Dataset_190_obs_2_9_21$SWEMWBS_metric, Dataset_190_obs_2_9_21$preocc_rating, method = "spearman")

## Warning in cor.test.default(Dataset_190_obs_2_9_21$SWEMWBS_metric,
## Dataset_190_obs_2_9_21$preocc_rating, : Cannot compute exact p-value with ties

##
## Spearman's rank correlation rho
##
## data: Dataset_190_obs_2_9_21$SWEMWBS_metric and Dataset_190_obs_2_9_21$preocc_rating
## S = 852170, p-value = 0.4435
## alternative hypothesis: true rho is not equal to 0
## sample estimates:
## rho
## -0.05933271

cor.test(Dataset_190_obs_2_9_21$SWEMWBS_metric, Dataset_190_obs_2_9_21$dismiss_rating, method = "spearman")

## Warning in cor.test.default(Dataset_190_obs_2_9_21$SWEMWBS_metric,
## Dataset_190_obs_2_9_21$dismiss_rating, : Cannot compute exact p-value with ties

##
## Spearman's rank correlation rho
##
## data: Dataset_190_obs_2_9_21$SWEMWBS_metric and Dataset_190_obs_2_9_21$dismiss_rating
## S = 658657, p-value = 0.05073
## alternative hypothesis: true rho is not equal to 0
## sample estimates:
## rho
## 0.1514512

**CAPE positive** Check distribution and then calculate correlations between CAPE positive and other variables

hist(Dataset_190_obs_2_9_21$CAPE_positive)


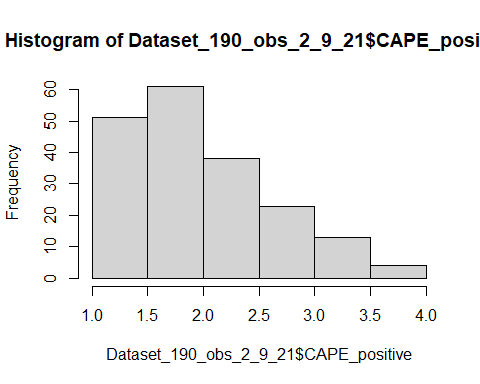


shapiro.test(Dataset_190_obs_2_9_21$CAPE_positive)

##
## Shapiro-Wilk normality test
##
## data: Dataset_190_obs_2_9_21$CAPE_positive
## W = 0.96, p-value = 3.206e-05

#CAPE subscales
cor.test(Dataset_190_obs_2_9_21$CAPE_negative, Dataset_190_obs_2_9_21$CAPE_positive,
 method = "spearman")

## Warning in cor.test.default(Dataset_190_obs_2_9_21$CAPE_negative,
## Dataset_190_obs_2_9_21$CAPE_positive, : Cannot compute exact p-value with ties

##
## Spearman's rank correlation rho
##
## data: Dataset_190_obs_2_9_21$CAPE_negative and Dataset_190_obs_2_9_21$CAPE_positive
## S = 471998, p-value < 2.2e-16
## alternative hypothesis: true rho is not equal to 0
## sample estimates:
## rho
## 0.5871018

cor.test(Dataset_190_obs_2_9_21$CAPE_depressive, Dataset_190_obs_2_9_21$CAPE_positive, method = "spearman")

## Warning in cor.test.default(Dataset_190_obs_2_9_21$CAPE_depressive,
## Dataset_190_obs_2_9_21$CAPE_positive, : Cannot compute exact p-value with ties

##
## Spearman's rank correlation rho
##
## data: Dataset_190_obs_2_9_21$CAPE_depressive and Dataset_190_obs_2_9_21$CAPE_positive
## S = 389036, p-value < 2.2e-16
## alternative hypothesis: true rho is not equal to 0
## sample estimates:
## rho
## 0.6596764

#Loneliness
cor.test(Dataset_190_obs_2_9_21$LonelinessTotal, Dataset_190_obs_2_9_21$CAPE_positive, method = "spearman")

## Warning in cor.test.default(Dataset_190_obs_2_9_21$LonelinessTotal,
## Dataset_190_obs_2_9_21$CAPE_positive, : Cannot compute exact p-value with ties

##
## Spearman's rank correlation rho
##
## data: Dataset_190_obs_2_9_21$LonelinessTotal and Dataset_190_obs_2_9_21$CAPE_positive
## S = 650384, p-value = 4.332e-07
## alternative hypothesis: true rho is not equal to 0
## sample estimates:
## rho
## 0.363233

#Internalised stigma
cor.test(ismi.df$ISMI_total, ismi.df$CAPE_positive, method = "spearman")

## Warning in cor.test.default(ismi.df$ISMI_total, ismi.df$CAPE_positive, method =
## "spearman"): Cannot compute exact p-value with ties

##
## Spearman's rank correlation rho
##
## data: ismi.df$ISMI_total and ismi.df$CAPE_positive
## S = 543998, p-value = 2.879e-13
## alternative hypothesis: true rho is not equal to 0
## sample estimates:
## rho
## 0.5008439

#Self-esteem
cor.test(Dataset_190_obs_2_9_21$SERS_total, Dataset_190_obs_2_9_21$CAPE_positive,
 method = "spearman")

## Warning in cor.test.default(Dataset_190_obs_2_9_21$SERS_total,
## Dataset_190_obs_2_9_21$CAPE_positive, : Cannot compute exact p-value with ties

##
## Spearman's rank correlation rho
##
## data: Dataset_190_obs_2_9_21$SERS_total and Dataset_190_obs_2_9_21$CAPE_positive
## S = 1526910, p-value < 2.2e-16
## alternative hypothesis: true rho is not equal to 0
## sample estimates:
## rho
## -0.5709437

#Social support
cor.test(Dataset_190_obs_2_9_21$mspss_ff, Dataset_190_obs_2_9_21$CAPE_positive,
 method = "spearman")

## Warning in cor.test.default(Dataset_190_obs_2_9_21$mspss_ff,
## Dataset_190_obs_2_9_21$CAPE_positive, : Cannot compute exact p-value with ties

##
## Spearman's rank correlation rho
##
## data: Dataset_190_obs_2_9_21$mspss_ff and Dataset_190_obs_2_9_21$CAPE_positive
## S = 1434116, p-value = 1.845e-10
## alternative hypothesis: true rho is not equal to 0
## sample estimates:
## rho
## -0.4511524

#Attachment
cor.test(Dataset_190_obs_2_9_21$secure_rating, Dataset_190_obs_2_9_21$CAPE_positive,
 method = "spearman")

## Warning in cor.test.default(Dataset_190_obs_2_9_21$secure_rating,
## Dataset_190_obs_2_9_21$CAPE_positive, : Cannot compute exact p-value with ties

##
## Spearman's rank correlation rho
##
## data: Dataset_190_obs_2_9_21$secure_rating and Dataset_190_obs_2_9_21$CAPE_positive
## S = 1147310, p-value = 2.055e-06
## alternative hypothesis: true rho is not equal to 0
## sample estimates:
## rho
## -0.3528868

cor.test(Dataset_190_obs_2_9_21$fearful_rating, Dataset_190_obs_2_9_21$CAPE_positive,method = "spearman")

## Warning in cor.test.default(Dataset_190_obs_2_9_21$fearful_rating,
## Dataset_190_obs_2_9_21$CAPE_positive, : Cannot compute exact p-value with ties

##
## Spearman's rank correlation rho
##
## data: Dataset_190_obs_2_9_21$fearful_rating and Dataset_190_obs_2_9_21$CAPE_positive
## S = 561850, p-value = 1.373e-05
## alternative hypothesis: true rho is not equal to 0
## sample estimates:
## rho
## 0.3257859

cor.test(Dataset_190_obs_2_9_21$preocc_rating, Dataset_190_obs_2_9_21$CAPE_positive,
 method = "spearman")

## Warning in cor.test.default(Dataset_190_obs_2_9_21$preocc_rating,
## Dataset_190_obs_2_9_21$CAPE_positive, : Cannot compute exact p-value with ties

##
## Spearman's rank correlation rho
##
## data: Dataset_190_obs_2_9_21$preocc_rating and Dataset_190_obs_2_9_21$CAPE_positive
## S = 743382, p-value = 0.2322
## alternative hypothesis: true rho is not equal to 0
## sample estimates:
## rho
## 0.09211303

cor.test(Dataset_190_obs_2_9_21$dismiss_rating, Dataset_190_obs_2_9_21$CAPE_positive, method = "spearman")

## Warning in cor.test.default(Dataset_190_obs_2_9_21$dismiss_rating,
## Dataset_190_obs_2_9_21$CAPE_positive, : Cannot compute exact p-value with ties

##
## Spearman's rank correlation rho
##
## data: Dataset_190_obs_2_9_21$dismiss_rating and Dataset_190_obs_2_9_21$CAPE_positive
## S = 865288, p-value = 0.2208
## alternative hypothesis: true rho is not equal to 0
## sample estimates:
## rho
## -0.09496274

**CAPE negative** Check distribution and then calculate correlations between CAPE negative and other variables. CAPE negative scores are normally distrbuted, check distribution of other variables before calculating correlations

hist(Dataset_190_obs_2_9_21$CAPE_negative)


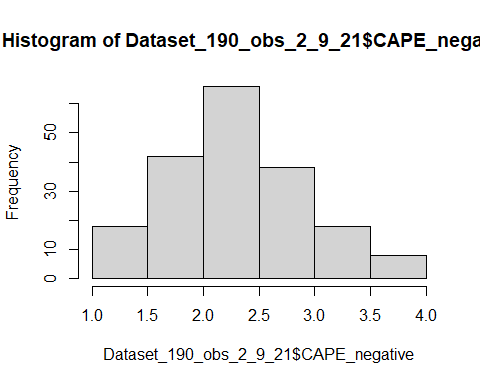


shapiro.test(Dataset_190_obs_2_9_21$CAPE_negative)

##
## Shapiro-Wilk normality test
##
## data: Dataset_190_obs_2_9_21$CAPE_negative
## W = 0.98707, p-value = 0.07991

#CAPE depressive
hist(Dataset_190_obs_2_9_21$CAPE_depressive)


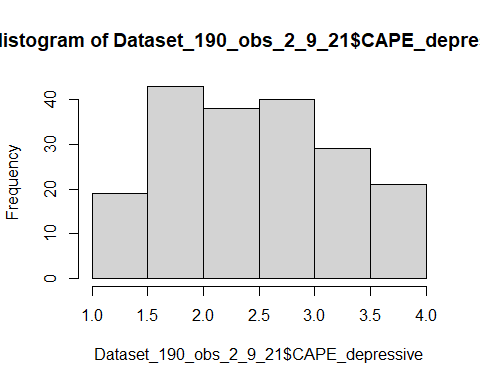


shapiro.test(Dataset_190_obs_2_9_21$CAPE_depressive) # CAPE depressive scores are not normally distributed

##
## Shapiro-Wilk normality test
##
## data: Dataset_190_obs_2_9_21$CAPE_depressive
## W = 0.96837, p-value = 0.0002717

cor.test(Dataset_190_obs_2_9_21$CAPE_negative, Dataset_190_obs_2_9_21$CAPE_depressive, method = "spearman")

## Warning in cor.test.default(Dataset_190_obs_2_9_21$CAPE_negative,
## Dataset_190_obs_2_9_21$CAPE_depressive, : Cannot compute exact p-value with ties

##
## Spearman's rank correlation rho
##
## data: Dataset_190_obs_2_9_21$CAPE_negative and Dataset_190_obs_2_9_21$CAPE_depressive
## S = 328352, p-value < 2.2e-16
## alternative hypothesis: true rho is not equal to 0
## sample estimates:
## rho
## 0.7127616

#Loneliness
hist(Dataset_190_obs_2_9_21$LonelinessTotal)


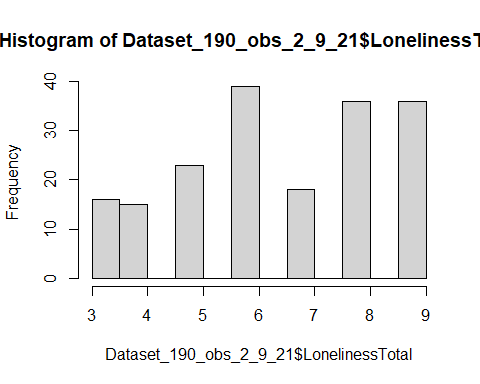


shapiro.test(Dataset_190_obs_2_9_21$LonelinessTotal) # loneliness scores are not normally distributed

##
## Shapiro-Wilk normality test
##
## data: Dataset_190_obs_2_9_21$LonelinessTotal
## W = 0.913, p-value = 6.184e-09

cor.test(Dataset_190_obs_2_9_21$LonelinessTotal, Dataset_190_obs_2_9_21$CAPE_negative, method = "spearman")

## Warning in cor.test.default(Dataset_190_obs_2_9_21$LonelinessTotal,
## Dataset_190_obs_2_9_21$CAPE_negative, : Cannot compute exact p-value with ties

##
## Spearman's rank correlation rho
##
## data: Dataset_190_obs_2_9_21$LonelinessTotal and Dataset_190_obs_2_9_21$CAPE_negative
## S = 588494, p-value = 2.254e-09
## alternative hypothesis: true rho is not equal to 0
## sample estimates:
## rho
## 0.4238269

#Internalised stigma
hist(ismi.df$ISMI_total)


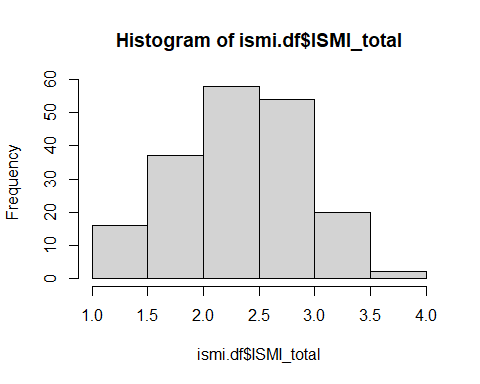


shapiro.test(ismi.df$ISMI_total) #internalised stigma scores are normally distrbuted

##
## Shapiro-Wilk normality test
##
## data: ismi.df$ISMI_total
## W = 0.9872, p-value = 0.08853

cor.test(ismi.df$ISMI_total, ismi.df$CAPE_negative, method = "pearson")

##
## Pearson's product-moment correlation
##
## data: ismi.df$ISMI_total and ismi.df$CAPE_negative
## t = 9.2184, df = 185, p-value < 2.2e-16
## alternative hypothesis: true correlation is not equal to 0
## 95 percent confidence interval:
## 0.4541005 0.6520374
## sample estimates:
## cor
## 0.5610364

#Self esteem
hist(Dataset_190_obs_2_9_21$SERS_total)


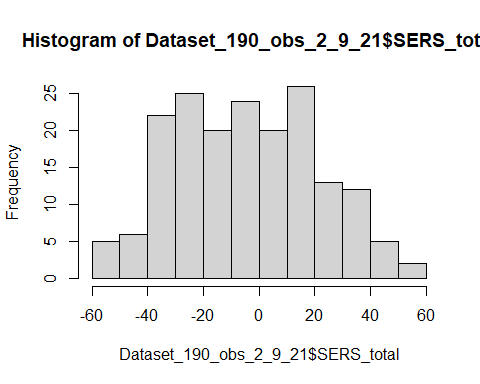


shapiro.test(Dataset_190_obs_2_9_21$SERS_total) # self esteem scores are not normally distributed

##
## Shapiro-Wilk normality test
##
## data: Dataset_190_obs_2_9_21$SERS_total
## W = 0.98402, p-value = 0.03764

cor.test(Dataset_190_obs_2_9_21$SERS_total, Dataset_190_obs_2_9_21$CAPE_negative,
 method = "spearman")

## Warning in cor.test.default(Dataset_190_obs_2_9_21$SERS_total,
## Dataset_190_obs_2_9_21$CAPE_negative, : Cannot compute exact p-value with ties

##
## Spearman's rank correlation rho
##
## data: Dataset_190_obs_2_9_21$SERS_total and Dataset_190_obs_2_9_21$CAPE_negative
## S = 1573981, p-value < 2.2e-16
## alternative hypothesis: true rho is not equal to 0
## sample estimates:
## rho
## -0.6193725

#Social support
hist(Dataset_190_obs_2_9_21$mspss_ff)


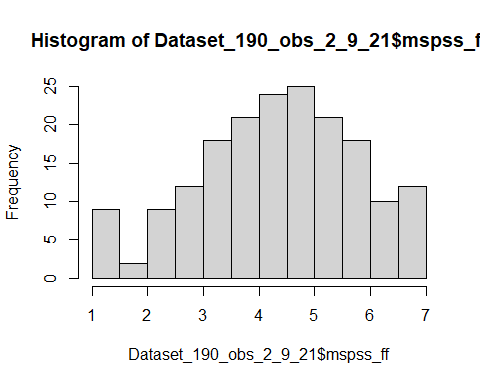


shapiro.test(Dataset_190_obs_2_9_21$mspss_ff) # social support scores are not normally distributed

##
## Shapiro-Wilk normality test
##
## data: Dataset_190_obs_2_9_21$mspss_ff
## W = 0.98215, p-value = 0.02046

cor.test(Dataset_190_obs_2_9_21$mspss_ff, Dataset_190_obs_2_9_21$CAPE_negative,
 method = "spearman")

## Warning in cor.test.default(Dataset_190_obs_2_9_21$mspss_ff,
## Dataset_190_obs_2_9_21$CAPE_negative, : Cannot compute exact p-value with ties

##
## Spearman's rank correlation rho
##
## data: Dataset_190_obs_2_9_21$mspss_ff and Dataset_190_obs_2_9_21$CAPE_negative
## S = 1357900, p-value = 2.14e-07
## alternative hypothesis: true rho is not equal to 0
## sample estimates:
## rho
## -0.3740308

#Attachment
hist(Dataset_190_obs_2_9_21$secure_rating)


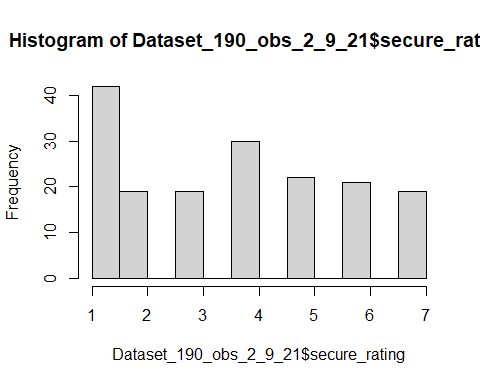


shapiro.test(Dataset_190_obs_2_9_21$secure_rating) # not normally distributed

##
## Shapiro-Wilk normality test
##
## data: Dataset_190_obs_2_9_21$secure_rating
## W = 0.90011, p-value = 2.185e-09

cor.test(Dataset_190_obs_2_9_21$secure_rating, Dataset_190_obs_2_9_21$CAPE_negative,
 method = "spearman")

## Warning in cor.test.default(Dataset_190_obs_2_9_21$secure_rating,
## Dataset_190_obs_2_9_21$CAPE_negative, : Cannot compute exact p-value with ties

##
## Spearman's rank correlation rho
##
## data: Dataset_190_obs_2_9_21$secure_rating and Dataset_190_obs_2_9_21$CAPE_negative
## S = 1196360, p-value = 2.19e-08
## alternative hypothesis: true rho is not equal to 0
## sample estimates:
## rho
## -0.4107248

hist(Dataset_190_obs_2_9_21$fearful_rating)


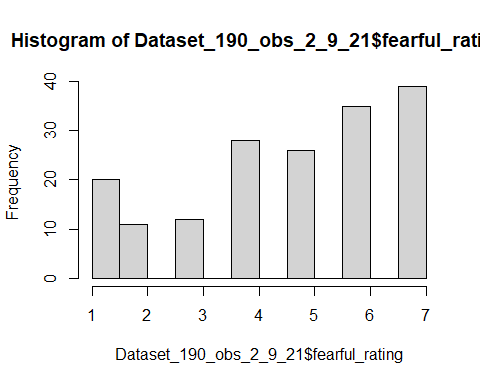


shapiro.test(Dataset_190_obs_2_9_21$fearful_rating) # not normally distributed

##
## Shapiro-Wilk normality test
##
## data: Dataset_190_obs_2_9_21$fearful_rating
## W = 0.88628, p-value = 3.82e-10

cor.test(Dataset_190_obs_2_9_21$fearful_rating, Dataset_190_obs_2_9_21$CAPE_negative, method = "spearman")

## Warning in cor.test.default(Dataset_190_obs_2_9_21$fearful_rating,
## Dataset_190_obs_2_9_21$CAPE_negative, : Cannot compute exact p-value with ties

##
## Spearman's rank correlation rho
##
## data: Dataset_190_obs_2_9_21$fearful_rating and Dataset_190_obs_2_9_21$CAPE_negative
## S = 615480, p-value = 0.0005527
## alternative hypothesis: true rho is not equal to 0
## sample estimates:
## rho
## 0.2614296

hist(Dataset_190_obs_2_9_21$preocc_rating)


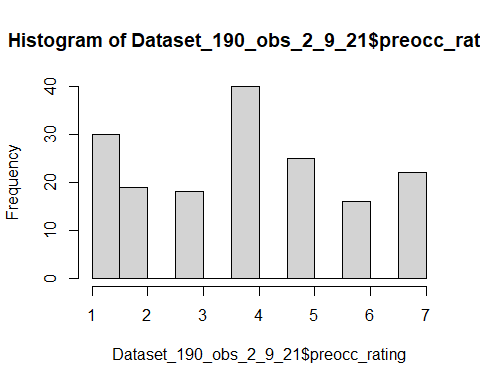


shapiro.test(Dataset_190_obs_2_9_21$preocc_rating) # not normally distributed

##
## Shapiro-Wilk normality test
##
## data: Dataset_190_obs_2_9_21$preocc_rating
## W = 0.91981, p-value = 4.606e-08

cor.test(Dataset_190_obs_2_9_21$preocc_rating, Dataset_190_obs_2_9_21$CAPE_negative,
 method = "spearman")

## Warning in cor.test.default(Dataset_190_obs_2_9_21$preocc_rating,
## Dataset_190_obs_2_9_21$CAPE_negative, : Cannot compute exact p-value with ties

##
## Spearman's rank correlation rho
##
## data: Dataset_190_obs_2_9_21$preocc_rating and Dataset_190_obs_2_9_21$CAPE_negative
## S = 721743, p-value = 0.1237
## alternative hypothesis: true rho is not equal to 0
## sample estimates:
## rho
## 0.1185408

hist(Dataset_190_obs_2_9_21$dismiss_rating)


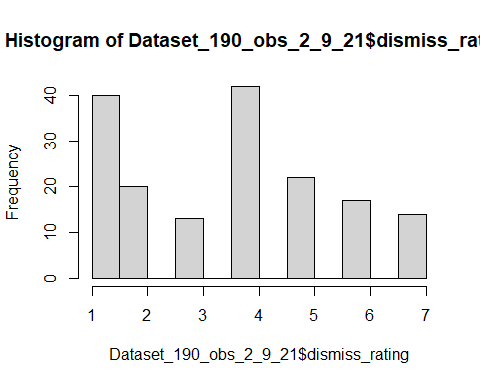


shapiro.test(Dataset_190_obs_2_9_21$dismiss_rating) # not normally distributed

##
## Shapiro-Wilk normality test
##
## data: Dataset_190_obs_2_9_21$dismiss_rating
## W = 0.90579, p-value = 6.635e-09

cor.test(Dataset_190_obs_2_9_21$dismiss_rating, Dataset_190_obs_2_9_21$CAPE_negative, method = "spearman")

## Warning in cor.test.default(Dataset_190_obs_2_9_21$dismiss_rating,
## Dataset_190_obs_2_9_21$CAPE_negative, : Cannot compute exact p-value with ties

##
## Spearman's rank correlation rho
##
## data: Dataset_190_obs_2_9_21$dismiss_rating and Dataset_190_obs_2_9_21$CAPE_negative
## S = 838013, p-value = 0.4364
## alternative hypothesis: true rho is not equal to 0
## sample estimates:
## rho
## -0.06044785

**CAPE Depressive** Calculate correlations between CAPE depressive and other variables. Established above that CAPE depressive scores are not normally distributed

#Loneliness
cor.test(Dataset_190_obs_2_9_21$LonelinessTotal, Dataset_190_obs_2_9_21$CAPE_depressive, method = "spearman")

## Warning in cor.test.default(Dataset_190_obs_2_9_21$LonelinessTotal,
## Dataset_190_obs_2_9_21$CAPE_depressive, : Cannot compute exact p-value with ties

##
## Spearman's rank correlation rho
##
## data: Dataset_190_obs_2_9_21$LonelinessTotal and Dataset_190_obs_2_9_21$CAPE_depressive
## S = 457048, p-value = 5.114e-16
## alternative hypothesis: true rho is not equal to 0
## sample estimates:
## rho
## 0.5525209

#Internalised stigma
cor.test(ismi.df$ISMI_total, ismi.df$CAPE_depressive, method = "spearman")

## Warning in cor.test.default(ismi.df$ISMI_total, ismi.df$CAPE_depressive, :
## Cannot compute exact p-value with ties

##
## Spearman's rank correlation rho
##
## data: ismi.df$ISMI_total and ismi.df$CAPE_depressive
## S = 464427, p-value < 2.2e-16
## alternative hypothesis: true rho is not equal to 0
## sample estimates:
## rho
## 0.5738556

#Self esteem
cor.test(Dataset_190_obs_2_9_21$SERS_total, Dataset_190_obs_2_9_21$CAPE_depressive,
 method = "spearman")

## Warning in cor.test.default(Dataset_190_obs_2_9_21$SERS_total,
## Dataset_190_obs_2_9_21$CAPE_depressive, : Cannot compute exact p-value with ties

##
## Spearman's rank correlation rho
##
## data: Dataset_190_obs_2_9_21$SERS_total and Dataset_190_obs_2_9_21$CAPE_depressive
## S = 1708403, p-value < 2.2e-16
## alternative hypothesis: true rho is not equal to 0
## sample estimates:
## rho
## -0.7576701

#Social support
cor.test(Dataset_190_obs_2_9_21$mspss_ff, Dataset_190_obs_2_9_21$CAPE_depressive,
 method = "spearman")

## Warning in cor.test.default(Dataset_190_obs_2_9_21$mspss_ff,
## Dataset_190_obs_2_9_21$CAPE_depressive, : Cannot compute exact p-value with ties

##
## Spearman's rank correlation rho
##
## data: Dataset_190_obs_2_9_21$mspss_ff and Dataset_190_obs_2_9_21$CAPE_depressive
## S = 1373213, p-value = 5.965e-08
## alternative hypothesis: true rho is not equal to 0
## sample estimates:
## rho
## -0.3895256

#Attachment
cor.test(Dataset_190_obs_2_9_21$secure_rating, Dataset_190_obs_2_9_21$CAPE_depressive, method = "spearman")

## Warning in cor.test.default(Dataset_190_obs_2_9_21$secure_rating,
## Dataset_190_obs_2_9_21$CAPE_depressive, : Cannot compute exact p-value with ties

##
## Spearman's rank correlation rho
##
## data: Dataset_190_obs_2_9_21$secure_rating and Dataset_190_obs_2_9_21$CAPE_depressive
## S = 1181586, p-value = 9.439e-08
## alternative hypothesis: true rho is not equal to 0
## sample estimates:
## rho
## -0.3933045

cor.test(Dataset_190_obs_2_9_21$fearful_rating, Dataset_190_obs_2_9_21$CAPE_depressive, method = "spearman")

## Warning in cor.test.default(Dataset_190_obs_2_9_21$fearful_rating,
## Dataset_190_obs_2_9_21$CAPE_depressive, : Cannot compute exact p-value with ties

##
## Spearman's rank correlation rho
##
## data: Dataset_190_obs_2_9_21$fearful_rating and Dataset_190_obs_2_9_21$CAPE_depressive
## S = 457034, p-value = 5.683e-10
## alternative hypothesis: true rho is not equal to 0
## sample estimates:
## rho
## 0.4515633

cor.test(Dataset_190_obs_2_9_21$preocc_rating, Dataset_190_obs_2_9_21$CAPE_depressive, method = "spearman")

## Warning in cor.test.default(Dataset_190_obs_2_9_21$preocc_rating,
## Dataset_190_obs_2_9_21$CAPE_depressive, : Cannot compute exact p-value with ties

##
## Spearman's rank correlation rho
##
## data: Dataset_190_obs_2_9_21$preocc_rating and Dataset_190_obs_2_9_21$CAPE_depressive
## S = 652529, p-value = 0.007909
## alternative hypothesis: true rho is not equal to 0
## sample estimates:
## rho
## 0.2030716

cor.test(Dataset_190_obs_2_9_21$dismiss_rating, Dataset_190_obs_2_9_21$CAPE_depressive, method = "spearman")

## Warning in cor.test.default(Dataset_190_obs_2_9_21$dismiss_rating,
## Dataset_190_obs_2_9_21$CAPE_depressive, : Cannot compute exact p-value with ties

##
## Spearman's rank correlation rho
##
## data: Dataset_190_obs_2_9_21$dismiss_rating and Dataset_190_obs_2_9_21$CAPE_depressive
## S = 947865, p-value = 0.00954
## alternative hypothesis: true rho is not equal to 0
## sample estimates:
## rho
## -0.1994586

**Loneliness** Calculate correlations between Loneliness and other variables. Established above that Loneliness scores are not normally distributed

#Internalised stigma
cor.test(ismi.df$ISMI_total, ismi.df$LonelinessTotal, method = "spearman")

## Warning in cor.test.default(ismi.df$ISMI_total, ismi.df$LonelinessTotal, :
## Cannot compute exact p-value with ties

##
## Spearman's rank correlation rho
##
## data: ismi.df$ISMI_total and ismi.df$LonelinessTotal
## S = 542902, p-value = 6.695e-11
## alternative hypothesis: true rho is not equal to 0
## sample estimates:
## rho
## 0.4596545

#Self esteem
cor.test(Dataset_190_obs_2_9_21$SERS_total, Dataset_190_obs_2_9_21$LonelinessTotal,
 method = "spearman")

## Warning in cor.test.default(Dataset_190_obs_2_9_21$SERS_total,
## Dataset_190_obs_2_9_21$LonelinessTotal, : Cannot compute exact p-value with ties

##
## Spearman's rank correlation rho
##
## data: Dataset_190_obs_2_9_21$SERS_total and Dataset_190_obs_2_9_21$LonelinessTotal
## S = 1419364, p-value < 2.2e-16
## alternative hypothesis: true rho is not equal to 0
## sample estimates:
## rho
## -0.5890771

#Social support
cor.test(Dataset_190_obs_2_9_21$mspss_ff, Dataset_190_obs_2_9_21$LonelinessTotal,
 method = "spearman")

## Warning in cor.test.default(Dataset_190_obs_2_9_21$mspss_ff,
## Dataset_190_obs_2_9_21$LonelinessTotal, : Cannot compute exact p-value with ties

##
## Spearman's rank correlation rho
##
## data: Dataset_190_obs_2_9_21$mspss_ff and Dataset_190_obs_2_9_21$LonelinessTotal
## S = 1259792, p-value = 6.77e-07
## alternative hypothesis: true rho is not equal to 0
## sample estimates:
## rho
## -0.363152

#Attachment
cor.test(Dataset_190_obs_2_9_21$secure_rating, Dataset_190_obs_2_9_21$LonelinessTotal, method = "spearman")

## Warning in cor.test.default(Dataset_190_obs_2_9_21$secure_rating,
## Dataset_190_obs_2_9_21$LonelinessTotal, : Cannot compute exact p-value with ties

##
## Spearman's rank correlation rho
##
## data: Dataset_190_obs_2_9_21$secure_rating and Dataset_190_obs_2_9_21$LonelinessTotal
## S = 1049114, p-value = 1.461e-05
## alternative hypothesis: true rho is not equal to 0
## sample estimates:
## rho
## -0.3275822

cor.test(Dataset_190_obs_2_9_21$fearful_rating, Dataset_190_obs_2_9_21$LonelinessTotal, method = "spearman")

## Warning in cor.test.default(Dataset_190_obs_2_9_21$fearful_rating,
## Dataset_190_obs_2_9_21$LonelinessTotal, : Cannot compute exact p-value with ties

##
## Spearman's rank correlation rho
##
## data: Dataset_190_obs_2_9_21$fearful_rating and Dataset_190_obs_2_9_21$LonelinessTotal
## S = 436773, p-value = 3.441e-09
## alternative hypothesis: true rho is not equal to 0
## sample estimates:
## rho
## 0.4373045

cor.test(Dataset_190_obs_2_9_21$preocc_rating, Dataset_190_obs_2_9_21$LonelinessTotal, method = "spearman")

## Warning in cor.test.default(Dataset_190_obs_2_9_21$preocc_rating,
## Dataset_190_obs_2_9_21$LonelinessTotal, : Cannot compute exact p-value with ties

##
## Spearman's rank correlation rho
##
## data: Dataset_190_obs_2_9_21$preocc_rating and Dataset_190_obs_2_9_21$LonelinessTotal
## S = 564929, p-value = 0.0007544
## alternative hypothesis: true rho is not equal to 0
## sample estimates:
## rho
## 0.2589682

cor.test(Dataset_190_obs_2_9_21$dismiss_rating, Dataset_190_obs_2_9_21$LonelinessTotal, method = "spearman")

## Warning in cor.test.default(Dataset_190_obs_2_9_21$dismiss_rating,
## Dataset_190_obs_2_9_21$LonelinessTotal, : Cannot compute exact p-value with ties

##
## Spearman's rank correlation rho
##
## data: Dataset_190_obs_2_9_21$dismiss_rating and Dataset_190_obs_2_9_21$LonelinessTotal
## S = 838832, p-value = 0.07159
## alternative hypothesis: true rho is not equal to 0
## sample estimates:
## rho
## -0.1410658

**Internalised stigma** Calculate correlations between internalised stigma and other variables. Established above that internalised stigma scores are normally distributed, however other variables are not.

#Self-esteem
cor.test(ismi.df$SERS_total, ismi.df$ISMI_total, method = "spearman")

## Warning in cor.test.default(ismi.df$SERS_total, ismi.df$ISMI_total, method =
## "spearman"): Cannot compute exact p-value with ties

##
## Spearman's rank correlation rho
##
## data: ismi.df$SERS_total and ismi.df$ISMI_total
## S = 1633693, p-value < 2.2e-16
## alternative hypothesis: true rho is not equal to 0
## sample estimates:
## rho
## -0.6808056

#Social support
cor.test(ismi.df$mspss_ff, ismi.df$ISMI_total, method = "spearman")

## Warning in cor.test.default(ismi.df$mspss_ff, ismi.df$ISMI_total, method =
## "spearman"): Cannot compute exact p-value with ties

##
## Spearman's rank correlation rho
##
## data: ismi.df$mspss_ff and ismi.df$ISMI_total
## S = 1387511, p-value = 1.703e-08
## alternative hypothesis: true rho is not equal to 0
## sample estimates:
## rho
## -0.4039942

#Attachment
cor.test(ismi.df$secure_rating,ismi.df$ISMI_total, method = "spearman")

## Warning in cor.test.default(ismi.df$secure_rating, ismi.df$ISMI_total, method =
## "spearman"): Cannot compute exact p-value with ties

##
## Spearman's rank correlation rho
##
## data: ismi.df$secure_rating and ismi.df$ISMI_total
## S = 1217452, p-value = 2.342e-09
## alternative hypothesis: true rho is not equal to 0
## sample estimates:
## rho
## -0.4355969

cor.test(ismi.df$fearful_rating, ismi.df$ISMI_total, method = "spearman")

## Warning in cor.test.default(ismi.df$fearful_rating, ismi.df$ISMI_total, : Cannot
## compute exact p-value with ties

##
## Spearman's rank correlation rho
##
## data: ismi.df$fearful_rating and ismi.df$ISMI_total
## S = 545176, p-value = 3.615e-06
## alternative hypothesis: true rho is not equal to 0
## sample estimates:
## rho
## 0.3457937

cor.test(ismi.df$preocc_rating, ismi.df$ISMI_total, method = "spearman")

## Warning in cor.test.default(ismi.df$preocc_rating, ismi.df$ISMI_total, method =
## "spearman"): Cannot compute exact p-value with ties

##
## Spearman's rank correlation rho
##
## data: ismi.df$preocc_rating and ismi.df$ISMI_total
## S = 692540, p-value = 0.04466
## alternative hypothesis: true rho is not equal to 0
## sample estimates:
## rho
## 0.1542067

cor.test(ismi.df$dismiss_rating, ismi.df$ISMI_total, method = "spearman")

## Warning in cor.test.default(ismi.df$dismiss_rating, ismi.df$ISMI_total, : Cannot
## compute exact p-value with ties

##
## Spearman's rank correlation rho
##
## data: ismi.df$dismiss_rating and ismi.df$ISMI_total
## S = 805205, p-value = 0.8076
## alternative hypothesis: true rho is not equal to 0
## sample estimates:
## rho
## -0.01893224

**Self esteem** Calculate correlations between self-esteem and other variables. Established above that self-esteem scores are not normally distributed

#Social support
cor.test(Dataset_190_obs_2_9_21$mspss_ff, Dataset_190_obs_2_9_21$SERS_total,
 method = "spearman")

## Warning in cor.test.default(Dataset_190_obs_2_9_21$mspss_ff,
## Dataset_190_obs_2_9_21$SERS_total, : Cannot compute exact p-value with ties

##
## Spearman's rank correlation rho
##
## data: Dataset_190_obs_2_9_21$mspss_ff and Dataset_190_obs_2_9_21$SERS_total
## S = 513225, p-value = 6.709e-11
## alternative hypothesis: true rho is not equal to 0
## sample estimates:
## rho
## 0.4630747

#Attachment
cor.test(Dataset_190_obs_2_9_21$SERS_total, Dataset_190_obs_2_9_21$secure_rating,
 method = "spearman")

## Warning in cor.test.default(Dataset_190_obs_2_9_21$SERS_total,
## Dataset_190_obs_2_9_21$secure_rating, : Cannot compute exact p-value with ties

##
## Spearman's rank correlation rho
##
## data: Dataset_190_obs_2_9_21$SERS_total and Dataset_190_obs_2_9_21$secure_rating
## S = 416716, p-value = 1.054e-12
## alternative hypothesis: true rho is not equal to 0
## sample estimates:
## rho
## 0.5086162

cor.test(Dataset_190_obs_2_9_21$SERS_total, Dataset_190_obs_2_9_21$fearful_rating,
 method = "spearman")

## Warning in cor.test.default(Dataset_190_obs_2_9_21$SERS_total,
## Dataset_190_obs_2_9_21$fearful_rating, : Cannot compute exact p-value with ties

##
## Spearman's rank correlation rho
##
## data: Dataset_190_obs_2_9_21$SERS_total and Dataset_190_obs_2_9_21$fearful_rating
## S = 1288950, p-value = 1.027e-14
## alternative hypothesis: true rho is not equal to 0
## sample estimates:
## rho
## -0.5467272

cor.test(Dataset_190_obs_2_9_21$SERS_total, Dataset_190_obs_2_9_21$preocc_rating,
 method = "spearman")

## Warning in cor.test.default(Dataset_190_obs_2_9_21$SERS_total,
## Dataset_190_obs_2_9_21$preocc_rating, : Cannot compute exact p-value with ties

##
## Spearman's rank correlation rho
##
## data: Dataset_190_obs_2_9_21$SERS_total and Dataset_190_obs_2_9_21$preocc_rating
## S = 985745, p-value = 0.007659
## alternative hypothesis: true rho is not equal to 0
## sample estimates:
## rho
## -0.2038826

cor.test(Dataset_190_obs_2_9_21$SERS_total, Dataset_190_obs_2_9_21$dismiss_rating,
 method = "spearman")

## Warning in cor.test.default(Dataset_190_obs_2_9_21$SERS_total,
## Dataset_190_obs_2_9_21$dismiss_rating, : Cannot compute exact p-value with ties

##
## Spearman's rank correlation rho
##
## data: Dataset_190_obs_2_9_21$SERS_total and Dataset_190_obs_2_9_21$dismiss_rating
## S = 665672, p-value = 0.04128
## alternative hypothesis: true rho is not equal to 0
## sample estimates:
## rho
## 0.1576369

**Social support** Calculate correlations between social support and attachment. Established above that social support scores are not normally distributed

cor.test(Dataset_190_obs_2_9_21$mspss_ff, Dataset_190_obs_2_9_21$secure_rating,
 method = "spearman")

## Warning in cor.test.default(Dataset_190_obs_2_9_21$mspss_ff,
## Dataset_190_obs_2_9_21$secure_rating, : Cannot compute exact p-value with ties

##
## Spearman's rank correlation rho
##
## data: Dataset_190_obs_2_9_21$mspss_ff and Dataset_190_obs_2_9_21$secure_rating
## S = 434402, p-value = 1.15e-11
## alternative hypothesis: true rho is not equal to 0
## sample estimates:
## rho
## 0.4877618

cor.test(Dataset_190_obs_2_9_21$mspss_ff, Dataset_190_obs_2_9_21$fearful_rating,
 method = "spearman")

## Warning in cor.test.default(Dataset_190_obs_2_9_21$mspss_ff,
## Dataset_190_obs_2_9_21$fearful_rating, : Cannot compute exact p-value with ties

##
## Spearman's rank correlation rho
##
## data: Dataset_190_obs_2_9_21$mspss_ff and Dataset_190_obs_2_9_21$fearful_rating
## S = 1012376, p-value = 0.004775
## alternative hypothesis: true rho is not equal to 0
## sample estimates:
## rho
## -0.2148419

cor.test(Dataset_190_obs_2_9_21$mspss_ff, Dataset_190_obs_2_9_21$preocc_rating,
 method = "spearman")

## Warning in cor.test.default(Dataset_190_obs_2_9_21$mspss_ff,
## Dataset_190_obs_2_9_21$preocc_rating, : Cannot compute exact p-value with ties

##
## Spearman's rank correlation rho
##
## data: Dataset_190_obs_2_9_21$mspss_ff and Dataset_190_obs_2_9_21$preocc_rating
## S = 824851, p-value = 0.9239
## alternative hypothesis: true rho is not equal to 0
## sample estimates:
## rho
## -0.007384529

cor.test(Dataset_190_obs_2_9_21$mspss_ff, Dataset_190_obs_2_9_21$dismiss_rating,
 method = "spearman")

## Warning in cor.test.default(Dataset_190_obs_2_9_21$mspss_ff,
## Dataset_190_obs_2_9_21$dismiss_rating, : Cannot compute exact p-value with ties

##
## Spearman's rank correlation rho
##
## data: Dataset_190_obs_2_9_21$mspss_ff and Dataset_190_obs_2_9_21$dismiss_rating
## S = 876250, p-value = 0.1602
## alternative hypothesis: true rho is not equal to 0
## sample estimates:
## rho
## -0.1088353

**Attachment** Calculate correlations between attachment types. Established above that all attachment scores are not normally distributed

#Secure
cor.test(Dataset_190_obs_2_9_21$fearful_rating, Dataset_190_obs_2_9_21$secure_rating, method = "spearman")

## Warning in cor.test.default(Dataset_190_obs_2_9_21$fearful_rating,
## Dataset_190_obs_2_9_21$secure_rating, : Cannot compute exact p-value with ties

##
## Spearman's rank correlation rho
##
## data: Dataset_190_obs_2_9_21$fearful_rating and Dataset_190_obs_2_9_21$secure_rating
## S = 1110158, p-value = 3.463e-07
## alternative hypothesis: true rho is not equal to 0
## sample estimates:
## rho
## -0.3800382

cor.test(Dataset_190_obs_2_9_21$preocc_rating, Dataset_190_obs_2_9_21$secure_rating,
 method = "spearman")

## Warning in cor.test.default(Dataset_190_obs_2_9_21$preocc_rating,
## Dataset_190_obs_2_9_21$secure_rating, : Cannot compute exact p-value with ties

##
## Spearman's rank correlation rho
##
## data: Dataset_190_obs_2_9_21$preocc_rating and Dataset_190_obs_2_9_21$secure_rating
## S = 790141, p-value = 0.8186
## alternative hypothesis: true rho is not equal to 0
## sample estimates:
## rho
## 0.01777539

cor.test(Dataset_190_obs_2_9_21$dismiss_rating, Dataset_190_obs_2_9_21$secure_rating, method = "spearman")

## Warning in cor.test.default(Dataset_190_obs_2_9_21$dismiss_rating,
## Dataset_190_obs_2_9_21$secure_rating, : Cannot compute exact p-value with ties

##
## Spearman's rank correlation rho
##
## data: Dataset_190_obs_2_9_21$dismiss_rating and Dataset_190_obs_2_9_21$secure_rating
## S = 818928, p-value = 0.6404
## alternative hypothesis: true rho is not equal to 0
## sample estimates:
## rho
## -0.03629717

#Fearful
cor.test(Dataset_190_obs_2_9_21$fearful_rating, Dataset_190_obs_2_9_21$preocc_rating, method = "spearman")

## Warning in cor.test.default(Dataset_190_obs_2_9_21$fearful_rating,
## Dataset_190_obs_2_9_21$preocc_rating, : Cannot compute exact p-value with ties

##
## Spearman's rank correlation rho
##
## data: Dataset_190_obs_2_9_21$fearful_rating and Dataset_190_obs_2_9_21$preocc_rating
## S = 720042, p-value = 0.1746
## alternative hypothesis: true rho is not equal to 0
## sample estimates:
## rho
## 0.1049147

cor.test(Dataset_190_obs_2_9_21$fearful_rating, Dataset_190_obs_2_9_21$dismiss_rating, method = "spearman")

## Warning in cor.test.default(Dataset_190_obs_2_9_21$fearful_rating,
## Dataset_190_obs_2_9_21$dismiss_rating, : Cannot compute exact p-value with ties

##
## Spearman's rank correlation rho
##
## data: Dataset_190_obs_2_9_21$fearful_rating and Dataset_190_obs_2_9_21$dismiss_rating
## S = 830155, p-value = 0.5156
## alternative hypothesis: true rho is not equal to 0
## sample estimates:
## rho
## -0.05050437

#Preoccupied & dismissing
cor.test(Dataset_190_obs_2_9_21$preocc_rating, Dataset_190_obs_2_9_21$dismiss_rating, method = "spearman")

## Warning in cor.test.default(Dataset_190_obs_2_9_21$preocc_rating,
## Dataset_190_obs_2_9_21$dismiss_rating, : Cannot compute exact p-value with ties

##
## Spearman's rank correlation rho
##
## data: Dataset_190_obs_2_9_21$preocc_rating and Dataset_190_obs_2_9_21$dismiss_rating
## S = 969868, p-value = 0.003046
## alternative hypothesis: true rho is not equal to 0
## sample estimates:
## rho
## -0.2273014
