# Supplementary material for: Being more satisfied with romantic relationship status is associated with increased mental wellbeing in people with experience of psychosis
Source: Front Psychiatry. 2023 Sep 28;14:1232973. doi: 10.3389/fpsyt.2023.1232973 (PMC10569177; doi:10.3389/fpsyt.2023.1232973)
Supplement: Supplementary file 4 [file Data_Sheet_4.DOCX]

Descriptive statistics

Rebecca White

21/12/2021

Load packages and dataset

library(psych)
library(readr)
library(tidyverse)

## -- Attaching packages --------------------------------------- tidyverse 1.3.0 --

## v ggplot2 3.3.2 v dplyr 1.0.2
## v tibble 3.0.4 v stringr 1.4.0
## v tidyr 1.1.2 v forcats 0.5.0
## v purrr 0.3.4

## -- Conflicts ------------------------------------------ tidyverse_conflicts() --
## x ggplot2::%+%() masks psych::%+%()
## x ggplot2::alpha() masks psych::alpha()
## x dplyr::filter() masks stats::filter()
## x dplyr::lag() masks stats::lag()

Dataset_190_obs_2_9_21 <- read_csv("Z:/Online study IRAS ID 271957/Online analysis/Dataset_190_obs_2.9.21.csv")

## Warning: Missing column names filled in: 'X1' [1]

##
## -- Column specification --------------------------------------------------------
## cols(
## .default = col_double(),
## redcap_survey_identifier = col_logical(),
## pis_timestamp = col_datetime(format = ""),
## screening_questions_timestamp = col_datetime(format = ""),
## demographic_information_timestamp = col_datetime(format = ""),
## nationality = col_character(),
## ethnicity_other = col_character(),
## gender_self_describe = col_character(),
## sexual_orientation_selfdescribe = col_character(),
## rr_selfdescribe = col_character(),
## last_rr_end = col_character(),
## current_rr_length = col_character(),
## the_community_assessment_of_psychic_experiences_ca_timestamp = col_datetime(format = ""),
## the_short_warwick_mental_health_wellbeing_scale_timestamp = col_datetime(format = ""),
## adapted_satisfaction_with_relationships_scale_rest_timestamp = col_datetime(format = ""),
## three_item_loneliness_scale_timestamp = col_datetime(format = ""),
## internalised_stigma_of_mental_illness_inventory_10_timestamp = col_datetime(format = ""),
## multidimensional_scale_of_perceived_social_support_timestamp = col_datetime(format = ""),
## self_esteem_rating_scale_short_form_serssf_timestamp = col_datetime(format = ""),
## relationships_questionnaire_timestamp = col_datetime(format = ""),
## Screening_Qs_result = col_character()
## # ... with 7 more columns
## )
## i Use `spec()` for the full column specifications.

View(Dataset_190_obs_2_9_21)

**Satisfaction with Relationship Scale (ReSta)**

describe(Dataset_190_obs_2_9_21$Resta.total)

## vars n mean sd median trimmed mad min max range skew kurtosis se
## X1 1 190 9.73 4.69 10.5 10.18 5.19 0 15 15 -0.58 -0.92 0.34

**Short Warwick Edinburgh Mental Wellbeing Scale (SWEMWBS)**

describe(Dataset_190_obs_2_9_21$SWEMWBS_metric)

## vars n mean sd median trimmed mad min max range skew kurtosis se
## X1 1 189 18.94 4.68 17.98 18.78 3.94 7 35 28 0.34 0.68 0.34

**Community Assessment of Psychic Experiences (CAPE) subscales**

describe(Dataset_190_obs_2_9_21$CAPE_positive)

## vars n mean sd median trimmed mad min max range skew kurtosis se
## X1 1 190 2 0.64 1.92 1.96 0.7 1 3.85 2.85 0.6 -0.26 0.05

describe(Dataset_190_obs_2_9_21$CAPE_negative)

## vars n mean sd median trimmed mad min max range skew kurtosis se
## X1 1 190 2.34 0.62 2.29 2.31 0.64 1 4 3 0.33 -0.21 0.04

describe(Dataset_190_obs_2_9_21$CAPE_depressive)

## vars n mean sd median trimmed mad min max range skew kurtosis se
## X1 1 190 2.53 0.74 2.5 2.51 0.93 1 4 3 0.15 -1 0.05

**Loneliness Scale (LS)**

describe(Dataset_190_obs_2_9_21$LonelinessTotal)

## vars n mean sd median trimmed mad min max range skew kurtosis se
## X1 1 183 6.53 1.91 6 6.65 2.97 3 9 6 -0.3 -1.04 0.14

**Internalised Stigma of Mental Illness Inventory - 10 item version (ISMI-10)**

ismi <- data.frame(Dataset_190_obs_2_9_21$ISMI_total)

ismi %>%
 filter(! (Dataset_190_obs_2_9_21.ISMI_total == "Inf")) -> ismi

describe(ismi$Dataset_190_obs_2_9_21.ISMI_total)

## vars n mean sd median trimmed mad min max range skew kurtosis se
## X1 1 187 2.38 0.57 2.4 2.39 0.59 1.1 3.7 2.6 -0.1 -0.53 0.04

**Multidimensional Scale of Perceived Social Support (MSPSS) - friends and family subscale only**

describe(Dataset_190_obs_2_9_21$mspss_ff)

## vars n mean sd median trimmed mad min max range skew kurtosis se
## X1 1 181 4.4 1.44 4.5 4.44 1.48 1 7 6 -0.27 -0.48 0.11

**Attachment, as measured by the Relationship Questionnaire (RQ)**

describe(Dataset_190_obs_2_9_21$secure_rating)

## vars n mean sd median trimmed mad min max range skew kurtosis se
## X1 1 172 3.64 2.06 4 3.55 2.97 1 7 6 0.13 -1.29 0.16

describe(Dataset_190_obs_2_9_21$fearful_rating)

## vars n mean sd median trimmed mad min max range skew kurtosis se
## X1 1 171 4.7 1.99 5 4.87 1.48 1 7 6 -0.55 -0.89 0.15

describe(Dataset_190_obs_2_9_21$preocc_rating)

## vars n mean sd median trimmed mad min max range skew kurtosis se
## X1 1 170 3.86 1.96 4 3.83 2.97 1 7 6 0.02 -1.09 0.15

describe(Dataset_190_obs_2_9_21$dismiss_rating)

## vars n mean sd median trimmed mad min max range skew kurtosis se
## X1 1 168 3.55 1.95 4 3.46 2.97 1 7 6 0.12 -1.15 0.15
