# Supplementary material for: Being more satisfied with romantic relationship status is associated with increased mental wellbeing in people with experience of psychosis
Source: Front Psychiatry. 2023 Sep 28;14:1232973. doi: 10.3389/fpsyt.2023.1232973 (PMC10569177; doi:10.3389/fpsyt.2023.1232973)
Supplement: Supplementary file 5 [file Data_Sheet_5.DOCX]

Non-response bias

Rebecca White

20/12/2021

Install tidyverse package, load and rename data set

library(tidyverse)

## -- Attaching packages --------------------------------------- tidyverse 1.3.0 --

## v ggplot2 3.3.2 v purrr 0.3.4
## v tibble 3.0.4 v dplyr 1.0.2
## v tidyr 1.1.2 v stringr 1.4.0
## v readr 1.4.0 v forcats 0.5.0

## -- Conflicts ------------------------------------------ tidyverse_conflicts() --
## x dplyr::filter() masks stats::filter()
## x dplyr::lag() masks stats::lag()

library(gmodels)

## Warning: package 'gmodels' was built under R version 4.0.5

library(readr)

DoesBeingMoreSatisfi_DATA_2021_07_06 <- read_csv("Z:/Online study IRAS ID 271957/Online analysis/Raw data/DoesBeingMoreSatisfi_DATA_2021-07-06.csv")

##
## -- Column specification --------------------------------------------------------
## cols(
## .default = col_double(),
## redcap_survey_identifier = col_logical(),
## pis_timestamp = col_datetime(format = ""),
## screening_questions_timestamp = col_datetime(format = ""),
## demographic_information_timestamp = col_character(),
## nationality = col_character(),
## ethnicity_other = col_character(),
## gender_self_describe = col_character(),
## sexual_orientation_selfdescribe = col_character(),
## rr_status = col_character(),
## rr_selfdescribe = col_character(),
## last_rr_end = col_character(),
## current_rr_length = col_character(),
## the_community_assessment_of_psychic_experiences_ca_timestamp = col_datetime(format = ""),
## the_short_warwick_mental_health_wellbeing_scale_timestamp = col_datetime(format = ""),
## adapted_satisfaction_with_relationships_scale_rest_timestamp = col_datetime(format = ""),
## three_item_loneliness_scale_timestamp = col_datetime(format = ""),
## internalised_stigma_of_mental_illness_inventory_10_timestamp = col_datetime(format = ""),
## multidimensional_scale_of_perceived_social_support_timestamp = col_datetime(format = ""),
## self_esteem_rating_scale_short_form_serssf_timestamp = col_datetime(format = ""),
## relationships_questionnaire_timestamp = col_datetime(format = "")
## )
## i Use `spec()` for the full column specifications.

View(DoesBeingMoreSatisfi_DATA_2021_07_06)

data <- DoesBeingMoreSatisfi_DATA_2021_07_06

Indentify how many participants passed the screening questions. Create a new column titled ‘Screening_Qs_result’ to be populated with ‘screening passed’ when participants have passed the three screening questions

data %>%
 mutate(Screening_Qs_result = case_when(over16 + (diagnosis | support | medication) == 2 ~ "screening passed" )) -> data

table(data$Screening_Qs_result)

##
## screening passed
## 232

232 participants passed the screening questions

Remove participants who did not pass the screening questions . Create a new dataset containing data from participants who passed the screening questions only

Dataset_screening_passed <- data[!is.na(data$Screening_Qs_result), ]

Identify how many items the remaining participants missed

apply(Dataset_screening_passed, 1, function(X) sum(is.na(X)))

## [1] 22 21 17 152 24 24 152 21 152 151 138 23 140 16 19 152 22 23
## [19] 152 24 22 21 151 28 23 24 48 152 25 22 21 21 20 152 24 25
## [37] 17 22 25 20 23 19 152 23 90 23 24 24 24 25 20 18 24 152
## [55] 19 24 19 20 20 139 139 25 23 63 22 21 152 20 152 24 13 17
## [73] 23 24 23 18 17 19 17 23 19 24 20 152 139 60 21 21 17 21
## [91] 138 21 16 20 20 139 20 152 152 17 139 34 24 19 15 152 152 152
## [109] 21 27 19 21 19 19 15 25 28 18 27 22 22 15 150 94 152 26
## [127] 23 22 24 24 14 20 26 20 21 17 39 36 20 20 140 24 19 140
## [145] 21 21 26 22 23 141 57 23 15 23 43 140 24 64 141 21 57 152
## [163] 19 35 41 33 37 35 19 79 151 21 16 82 16 21 20 152 34 35
## [181] 152 20 21 25 23 74 21 23 23 19 22 151 25 35 60 22 152 19
## [199] 23 17 23 24 28 21 19 24 21 22 23 18 18 31 21 16 25 35
## [217] 53 47 40 24 23 24 33 24 45 41 41 46 43 46 84 37

Dataset_screening_passed$nmiss <- apply(Dataset_screening_passed, 1, function(X) sum(is.na(X)))
table(Dataset_screening_passed$nmiss)

##
## 13 14 15 16 17 18 19 20 21 22 23 24 25 26 27 28 31 33 34 35
## 1 1 4 5 9 5 16 16 23 13 21 23 9 3 2 3 1 2 2 5
## 36 37 39 40 41 43 45 46 47 48 53 57 60 63 64 74 79 82 84 90
## 1 2 1 1 3 2 1 2 1 1 1 2 2 1 1 1 1 1 1 1
## 94 138 139 140 141 150 151 152
## 1 2 5 4 2 1 4 22

Participants who were missing fewer than 90 items were included in the analysis. Create new column in data set titled ‘retain’ to identify if participants have less than 90 missing items (TRUE - less than 90 missing items, participant will be included in the analysis; FALSE - 90 or more missing items, participants considered ‘non-responders’ and will not be included in the analysis)

Dataset_screening_passed$retain <- Dataset_screening_passed$nmiss < 90
table(Dataset_screening_passed$retain)

##
## FALSE TRUE
## 42 190

**Test whether the two groups differed by age**

age.test <- t.test(age ~ retain, data = Dataset_screening_passed)
age.test

##
## Welch Two Sample t-test
##
## data: age by retain
## t = -1.2465, df = 16.413, p-value = 0.2301
## alternative hypothesis: true difference in means is not equal to 0
## 95 percent confidence interval:
## -11.620622 3.003862
## sample estimates:
## mean in group FALSE mean in group TRUE
## 33.20000 37.50838

Non-responders did not differ significantly from those included in the anaylsis in terms of age

**Test whether the two groups differed by male/female gender** Gender is categorised as 1= female, 2 = male, 3 = prefer not to say, 4 = prefer to self describe. Give data these labels first

Dataset_screening_passed$GenderF <- factor(Dataset_screening_passed$gender,
 c(1,2,3,4), c("female", "male", "prefer_not_to_say", "Self_describe"))

table(Dataset_screening_passed$gender, Dataset_screening_passed$GenderF)

##
## female male prefer_not_to_say Self_describe
## 1 113 0 0 0
## 2 0 75 0 0
## 4 0 0 0 7

Dataset_screening_passed$GenderF <- factor(Dataset_screening_passed$GenderF,
 c("female","male"), c("female", "male"))
table(Dataset_screening_passed$GenderF)

##
## female male
## 113 75

library(gmodels)
CrossTable(Dataset_screening_passed$retain, Dataset_screening_passed$GenderF,
 fisher = TRUE, chisq = TRUE, expected = TRUE, sresid =TRUE, format = "SPSS")

##
## Cell Contents
## |-------------------------|
## | Count |
## | Expected Values |
## | Chi-square contribution |
## | Row Percent |
## | Column Percent |
## | Total Percent |
## | Std Residual |
## |-------------------------|
##
## Total Observations in Table: 188
##
## | Dataset_screening_passed$GenderF
## Dataset_screening_passed$retain | female | male | Row Total |
## --------------------------------|-----------|-----------|-----------|
## FALSE | 8 | 6 | 14 |
## | 8.415 | 5.585 | |
## | 0.020 | 0.031 | |
## | 57.143% | 42.857% | 7.447% |
## | 7.080% | 8.000% | |
## | 4.255% | 3.191% | |
## | -0.143 | 0.176 | |
## --------------------------------|-----------|-----------|-----------|
## TRUE | 105 | 69 | 174 |
## | 104.585 | 69.415 | |
## | 0.002 | 0.002 | |
## | 60.345% | 39.655% | 92.553% |
## | 92.920% | 92.000% | |
## | 55.851% | 36.702% | |
## | 0.041 | -0.050 | |
## --------------------------------|-----------|-----------|-----------|
## Column Total | 113 | 75 | 188 |
## | 60.106% | 39.894% | |
## --------------------------------|-----------|-----------|-----------|
##
##
## Statistics for All Table Factors
##
##
## Pearson's Chi-squared test
## ------------------------------------------------------------
## Chi^2 = 0.05540259 d.f. = 1 p = 0.8139157
##
## Pearson's Chi-squared test with Yates' continuity correction
## ------------------------------------------------------------
## Chi^2 = 2.168725e-31 d.f. = 1 p = 1
##
##
## Fisher's Exact Test for Count Data
## ------------------------------------------------------------
## Sample estimate odds ratio: 0.876816
##
## Alternative hypothesis: true odds ratio is not equal to 1
## p = 1
## 95% confidence interval: 0.2540721 3.208207
##
## Alternative hypothesis: true odds ratio is less than 1
## p = 0.5124513
## 95% confidence interval: 0 2.639313
##
## Alternative hypothesis: true odds ratio is greater than 1
## p = 0.70166
## 95% confidence interval: 0.3032891 Inf
##
##
##
## Minimum expected frequency: 5.585106

Non-responders did not differ significantly from those included in the anaylsis in terms of male/female gender

**Test whether the two groups differed by relationship status (single vs partner)**

Dataset_screening_passed$R_Status_simplified <- factor(Dataset_screening_passed$rr_status,
 c(1,2,3,4,5,6,9,7,8), c("single","dating","partner", "partner", "partner","partner", "separated", "widowed", "self describe"))

table(Dataset_screening_passed$rr_status, Dataset_screening_passed$R_Status_simplified)

##
## single dating partner separated widowed self describe
## 1 91 0 0 0 0 0
## 2 0 5 0 0 0 0
## 3 0 0 32 0 0 0
## 4 0 0 25 0 0 0
## 5 0 0 33 0 0 0
## 8 0 0 0 0 0 5
## 9 0 0 0 2 0 0
## Separated 0 0 0 0 0 0

Dataset_screening_passed %>%
 mutate(relationship.dicotomised = case_when(R_Status_simplified == "single" ~ "single", R_Status_simplified == "dating" ~ "single", R_Status_simplified == "separated" ~ "single", R_Status_simplified == "widowed" ~ "single", R_Status_simplified == "partner" ~ "partner", R_Status_simplified == "self describe" & rr_selfdescribe == "Living with a wife, queerplatonic partner, and steady, with one long-distance relationship as well" ~ "partner", R_Status_simplified == "self describe" ~ "single")) -> Dataset_screening_passed

table(Dataset_screening_passed$relationship.dicotomised)

##
## partner single
## 91 102

CrossTable(Dataset_screening_passed$retain,Dataset_screening_passed$relationship.dicotomised, fisher = TRUE, chisq = TRUE, expected = TRUE, sresid =TRUE, format = "SPSS")

##
## Cell Contents
## |-------------------------|
## | Count |
## | Expected Values |
## | Chi-square contribution |
## | Row Percent |
## | Column Percent |
## | Total Percent |
## | Std Residual |
## |-------------------------|
##
## Total Observations in Table: 193
##
## | Dataset_screening_passed$relationship.dicotomised
## Dataset_screening_passed$retain | partner | single | Row Total |
## --------------------------------|-----------|-----------|-----------|
## FALSE | 12 | 2 | 14 |
## | 6.601 | 7.399 | |
## | 4.416 | 3.940 | |
## | 85.714% | 14.286% | 7.254% |
## | 13.187% | 1.961% | |
## | 6.218% | 1.036% | |
## | 2.101 | -1.985 | |
## --------------------------------|-----------|-----------|-----------|
## TRUE | 79 | 100 | 179 |
## | 84.399 | 94.601 | |
## | 0.345 | 0.308 | |
## | 44.134% | 55.866% | 92.746% |
## | 86.813% | 98.039% | |
## | 40.933% | 51.813% | |
## | -0.588 | 0.555 | |
## --------------------------------|-----------|-----------|-----------|
## Column Total | 91 | 102 | 193 |
## | 47.150% | 52.850% | |
## --------------------------------|-----------|-----------|-----------|
##
##
## Statistics for All Table Factors
##
##
## Pearson's Chi-squared test
## ------------------------------------------------------------
## Chi^2 = 9.008866 d.f. = 1 p = 0.002686731
##
## Pearson's Chi-squared test with Yates' continuity correction
## ------------------------------------------------------------
## Chi^2 = 7.417503 d.f. = 1 p = 0.006459239
##
##
## Fisher's Exact Test for Count Data
## ------------------------------------------------------------
## Sample estimate odds ratio: 7.525633
##
## Alternative hypothesis: true odds ratio is not equal to 1
## p = 0.003893945
## 95% confidence interval: 1.605604 71.33493
##
## Alternative hypothesis: true odds ratio is less than 1
## p = 0.9997071
## 95% confidence interval: 0 48.25936
##
## Alternative hypothesis: true odds ratio is greater than 1
## p = 0.002600806
## 95% confidence interval: 1.928796 Inf
##
##
##
## Minimum expected frequency: 6.601036

Non responders were more likely than participants to have a partner.
