# Supplementary material for: Being more satisfied with romantic relationship status is associated with increased mental wellbeing in people with experience of psychosis
Source: Front Psychiatry. 2023 Sep 28;14:1232973. doi: 10.3389/fpsyt.2023.1232973 (PMC10569177; doi:10.3389/fpsyt.2023.1232973)
Supplement: Supplementary file 6 [file Data_Sheet_6.DOCX]

Cross-sectional analysis

Rebecca White

19/08/2021

Install packages

library(readr)
library(glue)
library(tidyverse)

## -- Attaching packages --------------------------------------- tidyverse 1.3.0 --

## v ggplot2 3.3.2 v dplyr 1.0.2
## v tibble 3.0.4 v stringr 1.4.0
## v tidyr 1.1.2 v forcats 0.5.0
## v purrr 0.3.4

## -- Conflicts ------------------------------------------ tidyverse_conflicts() --
## x dplyr::collapse() masks glue::collapse()
## x dplyr::filter() masks stats::filter()
## x dplyr::lag() masks stats::lag()

library(dplyr)
library(psych)

##
## Attaching package: 'psych'

## The following objects are masked from 'package:ggplot2':
##
## %+%, alpha

library(haven)

Import dataset

DoesBeingMoreSatisfi_DATA <- read_csv("Raw data/DoesBeingMoreSatisfi_DATA_2021-07-06.csv")

##
## -- Column specification --------------------------------------------------------
## cols(
## .default = col_double(),
## redcap_survey_identifier = col_logical(),
## pis_timestamp = col_datetime(format = ""),
## screening_questions_timestamp = col_datetime(format = ""),
## demographic_information_timestamp = col_character(),
## nationality = col_character(),
## ethnicity_other = col_character(),
## gender_self_describe = col_character(),
## sexual_orientation_selfdescribe = col_character(),
## rr_status = col_character(),
## rr_selfdescribe = col_character(),
## last_rr_end = col_character(),
## current_rr_length = col_character(),
## the_community_assessment_of_psychic_experiences_ca_timestamp = col_datetime(format = ""),
## the_short_warwick_mental_health_wellbeing_scale_timestamp = col_datetime(format = ""),
## adapted_satisfaction_with_relationships_scale_rest_timestamp = col_datetime(format = ""),
## three_item_loneliness_scale_timestamp = col_datetime(format = ""),
## internalised_stigma_of_mental_illness_inventory_10_timestamp = col_datetime(format = ""),
## multidimensional_scale_of_perceived_social_support_timestamp = col_datetime(format = ""),
## self_esteem_rating_scale_short_form_serssf_timestamp = col_datetime(format = ""),
## relationships_questionnaire_timestamp = col_datetime(format = "")
## )
## i Use `spec()` for the full column specifications.

Ensure only those who passed the screening questions are included in the data set Create a variable ‘screening passed’ and only retain rows where participants were over 16 years old and been given diagnosis/received support/taken medication for psychosis

DoesBeingMoreSatisfi_DATA %>%
 mutate(Screening_Qs_result = case_when(over16 + (diagnosis | support | medication) == 2 ~ "screening passed" )) -> DoesBeingMoreSatisfi_DATA

Remove any rows where screening wasn’t passed

Dataset_screening_passed <- DoesBeingMoreSatisfi_DATA[!is.na(DoesBeingMoreSatisfi_DATA$Screening_Qs_result), ]

Remove rows where lots of data is missing See how many participants are missing data

Dataset_screening_passed$nmiss <- apply(Dataset_screening_passed, 1, function(X) sum(is.na(X)))
table(Dataset_screening_passed$nmiss)

##
## 13 14 15 16 17 18 19 20 21 22 23 24 25 26 27 28 31 33 34 35
## 1 1 4 5 9 5 16 16 23 13 21 23 9 3 2 3 1 2 2 5
## 36 37 39 40 41 43 45 46 47 48 53 57 60 63 64 74 79 82 84 90
## 1 2 1 1 3 2 1 2 1 1 1 2 2 1 1 1 1 1 1 1
## 94 138 139 140 141 150 151 152
## 1 2 5 4 2 1 4 22

Remove rows where 90 or more variables are missing

Dataset_screening_passed$retain <- Dataset_screening_passed$nmiss < 90
table(Dataset_screening_passed$retain)

##
## FALSE TRUE
## 42 190

Dataset_Missing_Removed <- subset(Dataset_screening_passed, retain == TRUE)

190 rows remain in the dataset

**Demographic variables**

##### Age

describe(Dataset_Missing_Removed$age)

## vars n mean sd median trimmed mad min max range skew kurtosis se
## X1 1 179 37.51 12.81 36 36.9 14.83 16 66 50 0.38 -0.89 0.96

##### Gender

Gender is categorised as 1= female, 2 = male, 3 = prefer not to say, 4 = prefer to self describe Replace numbers with description

Dataset_Missing_Removed$GenderF <- factor(Dataset_Missing_Removed$gender, c(1,2,3,4), c("female", "male", "prefer_not_to_say", "Self_describe"))

table(Dataset_Missing_Removed$gender, Dataset_Missing_Removed$GenderF)

##
## female male prefer_not_to_say Self_describe
## 1 105 0 0 0
## 2 0 69 0 0
## 4 0 0 0 6

##### Ethnicity

1, White - British | 2, White - Irish | 3, Any other white background | 4, Mixed - White and Black Caribbean | 5, Mixed - White and Black African | 6, Mixed - White and Asian | 7, Any other mixed background | 8, Asian or Asian British - Indian | 9, Asian or Asian British - Pakistani | 10, Asian or Asian British - Bangladeshi | 11, Any other Asian/Asian British background | 12, Black or Black British - Caribbean | 13, Black or Black British - African | 14, Any other Black/Black British background | 15, Chinese | 16, Any other (please describe)

Reduce number of groups for the analysis

Dataset_Missing_Removed$EthnicityF <- factor(Dataset_Missing_Removed$ethnicity, c(1, 2, 3, 4, 5, 6, 7, 8, 9, 10, 11, 12, 13, 14, 15, 16), c("white", "white", "white", "mixed", "mixed", "mixed", "mixed", "asian","asian", "asian", "asian", "black", "black", "black", "chinese", "other"))
table(Dataset_Missing_Removed$EthnicityF)

##
## white mixed asian black chinese other
## 148 5 10 11 0 5

##### Sexual orientation

1, Bi | 2, Gay/Lesbian | 3, Heterosexual/straight | 4, Prefer not to say | 5, Prefer to self-describe

Replace numbers with description

Dataset_Missing_Removed$SexualityF <- factor(Dataset_Missing_Removed$sexual_orientation, c(1, 2, 3, 4, 5), c("bisexual", "gay/lesbian", "heterosexual", "prefer not to say", "self-describe"))

table(Dataset_Missing_Removed$SexualityF)

##
## bisexual gay/lesbian heterosexual prefer not to say
## 24 9 132 6
## self-describe
## 9

##### Relationship status

Relationship status is categorised as: 1, Single | 2, Dating/seeing someone but not ‘officially’ in a relationship | 3, In a relationship, not living together | 4, In a relationship, living together | 5, Married / civil partnership, living together | 6, Married / civil partnership, not living together | 9, Separated, but still legally married| 7, Widowed | 8, Prefer to self-describe

Reduce number of groups for the analysis - group in a relationship living together/not living together and married/civil partnership as in a relationship

Dataset_Missing_Removed$R_Status_simplified <- factor(Dataset_Missing_Removed$rr_status, c(1,2,3,4,5,6,9,7,8), c("single","dating","partner", "partner", "partner","partner", "separated", "widowed", "self describe"))

table(Dataset_Missing_Removed$rr_status, Dataset_Missing_Removed$R_Status_simplified)

##
## single dating partner separated widowed self describe
## 1 89 0 0 0 0 0
## 2 0 5 0 0 0 0
## 3 0 0 27 0 0 0
## 4 0 0 21 0 0 0
## 5 0 0 30 0 0 0
## 8 0 0 0 0 0 5
## 9 0 0 0 2 0 0

##### Employment

1, Employee | 2, Self Employed | 3, Unemployed | 4, Full-time education at school, college or university | 5, Looking after family/home | 6, Receipt of sickness or disability benefits | 7, Retired

Reduce groups for analysis

Dataset_Missing_Removed$EmploymentF <- factor(Dataset_Missing_Removed$employment, c(1,2,3,4,5,6,7), c("employee", "self-employed", "unemployed","FT education", "looking after home/family", "rec. sickness/disability benefits", "retired"))

table(Dataset_Missing_Removed$EmploymentF)

##
## employee self-employed
## 56 5
## unemployed FT education
## 26 21
## looking after home/family rec. sickness/disability benefits
## 6 58
## retired
## 5

**Check Cronbach’s Alpha and create composite variables**

**CAPE-42** [CAPE-42 Instructions](http://www.cape42.homestead.com/files/CAPEdimensionscore2003.pdf) on how to calculate scores for each of the subscales state that scores should be calculated by adding items together and dividing by the number of items completed

For each dimension (negative, positive, depressive) create data frame to calculate the number of negative items missing for each participant

CAPE negative first…

CAPE_negative.missing <- data.frame(Dataset_Missing_Removed$cape_3, Dataset_Missing_Removed$cape_4, Dataset_Missing_Removed$cape_8, Dataset_Missing_Removed$cape_16, Dataset_Missing_Removed$cape_18, Dataset_Missing_Removed$cape_21, Dataset_Missing_Removed$cape_23, Dataset_Missing_Removed$cape_25, Dataset_Missing_Removed$cape_27, Dataset_Missing_Removed$cape_29, Dataset_Missing_Removed$cape_32, Dataset_Missing_Removed$cape_35, Dataset_Missing_Removed$cape_36, Dataset_Missing_Removed$cape_37)

Cronbachs alpha

alpha(CAPE_negative.missing)

##
## Reliability analysis
## Call: alpha(x = CAPE_negative.missing)
##
## raw_alpha std.alpha G6(smc) average_r S/N ase mean sd median_r
## 0.9 0.9 0.92 0.4 9.4 0.01 2.3 0.62 0.39
##
## lower alpha upper 95% confidence boundaries
## 0.88 0.9 0.92
##
## Reliability if an item is dropped:
## raw_alpha std.alpha G6(smc) average_r S/N
## Dataset_Missing_Removed.cape_3 0.90 0.90 0.91 0.40 8.8
## Dataset_Missing_Removed.cape_4 0.90 0.90 0.92 0.42 9.3
## Dataset_Missing_Removed.cape_8 0.90 0.90 0.91 0.40 8.8
## Dataset_Missing_Removed.cape_16 0.90 0.90 0.92 0.41 9.0
## Dataset_Missing_Removed.cape_18 0.89 0.89 0.90 0.39 8.4
## Dataset_Missing_Removed.cape_21 0.90 0.90 0.91 0.40 8.8
## Dataset_Missing_Removed.cape_23 0.90 0.90 0.92 0.42 9.3
## Dataset_Missing_Removed.cape_25 0.89 0.89 0.91 0.39 8.5
## Dataset_Missing_Removed.cape_27 0.90 0.90 0.91 0.41 9.0
## Dataset_Missing_Removed.cape_29 0.89 0.89 0.91 0.39 8.5
## Dataset_Missing_Removed.cape_32 0.89 0.89 0.91 0.39 8.4
## Dataset_Missing_Removed.cape_35 0.90 0.90 0.91 0.41 9.1
## Dataset_Missing_Removed.cape_36 0.89 0.89 0.91 0.39 8.4
## Dataset_Missing_Removed.cape_37 0.90 0.90 0.91 0.40 8.7
## alpha se var.r med.r
## Dataset_Missing_Removed.cape_3 0.011 0.015 0.38
## Dataset_Missing_Removed.cape_4 0.010 0.013 0.41
## Dataset_Missing_Removed.cape_8 0.011 0.014 0.40
## Dataset_Missing_Removed.cape_16 0.011 0.015 0.41
## Dataset_Missing_Removed.cape_18 0.011 0.011 0.39
## Dataset_Missing_Removed.cape_21 0.011 0.013 0.39
## Dataset_Missing_Removed.cape_23 0.010 0.014 0.41
## Dataset_Missing_Removed.cape_25 0.011 0.013 0.38
## Dataset_Missing_Removed.cape_27 0.011 0.013 0.41
## Dataset_Missing_Removed.cape_29 0.011 0.014 0.38
## Dataset_Missing_Removed.cape_32 0.011 0.013 0.38
## Dataset_Missing_Removed.cape_35 0.011 0.012 0.40
## Dataset_Missing_Removed.cape_36 0.011 0.013 0.38
## Dataset_Missing_Removed.cape_37 0.011 0.014 0.39
##
## Item statistics
## n raw.r std.r r.cor r.drop mean sd
## Dataset_Missing_Removed.cape_3 189 0.67 0.67 0.64 0.60 2.4 0.88
## Dataset_Missing_Removed.cape_4 187 0.54 0.55 0.50 0.46 2.5 0.92
## Dataset_Missing_Removed.cape_8 187 0.65 0.65 0.62 0.58 2.1 0.91
## Dataset_Missing_Removed.cape_16 190 0.61 0.61 0.57 0.54 2.2 0.87
## Dataset_Missing_Removed.cape_18 189 0.76 0.75 0.75 0.71 2.8 0.90
## Dataset_Missing_Removed.cape_21 188 0.68 0.67 0.65 0.61 2.8 0.89
## Dataset_Missing_Removed.cape_23 187 0.55 0.56 0.50 0.47 1.8 0.89
## Dataset_Missing_Removed.cape_25 189 0.75 0.74 0.72 0.68 2.4 1.01
## Dataset_Missing_Removed.cape_27 188 0.61 0.62 0.59 0.55 2.1 0.81
## Dataset_Missing_Removed.cape_29 188 0.74 0.74 0.72 0.68 2.2 0.91
## Dataset_Missing_Removed.cape_32 188 0.74 0.75 0.75 0.69 2.2 0.87
## Dataset_Missing_Removed.cape_35 188 0.61 0.60 0.57 0.53 2.3 0.96
## Dataset_Missing_Removed.cape_36 188 0.76 0.75 0.74 0.71 2.5 0.90
## Dataset_Missing_Removed.cape_37 189 0.69 0.67 0.64 0.61 2.5 1.03
##
## Non missing response frequency for each item
## 1 2 3 4 miss
## Dataset_Missing_Removed.cape_3 0.16 0.44 0.29 0.11 0.01
## Dataset_Missing_Removed.cape_4 0.12 0.44 0.27 0.17 0.02
## Dataset_Missing_Removed.cape_8 0.26 0.42 0.22 0.09 0.02
## Dataset_Missing_Removed.cape_16 0.23 0.45 0.24 0.08 0.00
## Dataset_Missing_Removed.cape_18 0.08 0.32 0.38 0.23 0.01
## Dataset_Missing_Removed.cape_21 0.07 0.30 0.38 0.25 0.01
## Dataset_Missing_Removed.cape_23 0.45 0.35 0.14 0.06 0.02
## Dataset_Missing_Removed.cape_25 0.21 0.39 0.23 0.18 0.01
## Dataset_Missing_Removed.cape_27 0.26 0.47 0.23 0.04 0.01
## Dataset_Missing_Removed.cape_29 0.26 0.41 0.23 0.09 0.01
## Dataset_Missing_Removed.cape_32 0.20 0.46 0.25 0.09 0.01
## Dataset_Missing_Removed.cape_35 0.22 0.40 0.24 0.14 0.01
## Dataset_Missing_Removed.cape_36 0.11 0.45 0.27 0.17 0.01
## Dataset_Missing_Removed.cape_37 0.19 0.33 0.26 0.22 0.01

Identify how many NAs are in each observation

apply(CAPE_negative.missing, 1, function(X) sum(is.na(X)))

## [1] 0 0 0 0 0 1 0 0 0 0 0 0 0 0 0 0 0 0 0 0 0 0 0 0 0 0 0 0 0 0 0 0 0 0 0 0 0
## [38] 0 0 0 0 0 0 0 0 0 0 0 0 0 0 0 0 0 0 0 0 0 0 0 0 0 0 0 0 0 0 0 0 0 0 0 0 0
## [75] 0 0 1 0 0 1 0 0 0 0 0 0 0 1 1 0 0 0 0 0 0 0 0 0 0 0 0 0 0 0 0 0 0 0 0 0 0
## [112] 0 0 1 0 0 0 0 0 0 0 0 0 0 0 0 0 0 0 0 0 0 0 0 0 0 0 0 0 0 0 0 0 1 0 1 0 0
## [149] 0 0 0 0 0 8 0 0 0 0 0 1 0 0 0 0 0 0 0 0 0 0 0 0 0 0 0 0 0 0 0 0 0 0 0 0 0
## [186] 0 0 0 8 0

Create a new column that says how many NAs are in each observation(to be used for formula later)

Dataset_Missing_Removed$CAPE_negative.missing <- apply(CAPE_negative.missing, 1, function(X) sum(is.na(X)))

CAPE depressive

CAPE_depressive.missing <- data.frame(Dataset_Missing_Removed$cape_1, Dataset_Missing_Removed$cape_9, Dataset_Missing_Removed$cape_12, Dataset_Missing_Removed$cape_14, Dataset_Missing_Removed$cape_19, Dataset_Missing_Removed$cape_38, Dataset_Missing_Removed$cape_39, Dataset_Missing_Removed$cape_40)

Crohbach’s alpha

alpha(CAPE_depressive.missing)

##
## Reliability analysis
## Call: alpha(x = CAPE_depressive.missing)
##
## raw_alpha std.alpha G6(smc) average_r S/N ase mean sd median_r
## 0.9 0.91 0.91 0.54 9.5 0.011 2.5 0.74 0.52
##
## lower alpha upper 95% confidence boundaries
## 0.88 0.9 0.92
##
## Reliability if an item is dropped:
## raw_alpha std.alpha G6(smc) average_r S/N
## Dataset_Missing_Removed.cape_1 0.89 0.89 0.89 0.53 7.9
## Dataset_Missing_Removed.cape_9 0.90 0.90 0.90 0.56 8.8
## Dataset_Missing_Removed.cape_12 0.89 0.89 0.89 0.53 8.1
## Dataset_Missing_Removed.cape_14 0.89 0.89 0.89 0.53 7.9
## Dataset_Missing_Removed.cape_19 0.90 0.90 0.90 0.57 9.3
## Dataset_Missing_Removed.cape_38 0.89 0.89 0.89 0.55 8.5
## Dataset_Missing_Removed.cape_39 0.88 0.88 0.88 0.52 7.5
## Dataset_Missing_Removed.cape_40 0.90 0.90 0.90 0.56 8.9
## alpha se var.r med.r
## Dataset_Missing_Removed.cape_1 0.012 0.0108 0.51
## Dataset_Missing_Removed.cape_9 0.011 0.0083 0.52
## Dataset_Missing_Removed.cape_12 0.012 0.0088 0.52
## Dataset_Missing_Removed.cape_14 0.012 0.0096 0.52
## Dataset_Missing_Removed.cape_19 0.011 0.0083 0.59
## Dataset_Missing_Removed.cape_38 0.012 0.0104 0.55
## Dataset_Missing_Removed.cape_39 0.013 0.0085 0.52
## Dataset_Missing_Removed.cape_40 0.011 0.0105 0.53
##
## Item statistics
## n raw.r std.r r.cor r.drop mean sd
## Dataset_Missing_Removed.cape_1 190 0.81 0.82 0.79 0.75 2.7 0.79
## Dataset_Missing_Removed.cape_9 188 0.73 0.73 0.68 0.64 2.5 0.93
## Dataset_Missing_Removed.cape_12 187 0.81 0.81 0.78 0.73 2.4 1.01
## Dataset_Missing_Removed.cape_14 189 0.81 0.82 0.80 0.75 2.1 0.98
## Dataset_Missing_Removed.cape_19 189 0.68 0.68 0.61 0.58 2.1 0.98
## Dataset_Missing_Removed.cape_38 189 0.77 0.76 0.72 0.68 2.7 1.03
## Dataset_Missing_Removed.cape_39 188 0.87 0.86 0.86 0.81 2.8 1.01
## Dataset_Missing_Removed.cape_40 188 0.72 0.73 0.67 0.63 2.8 0.92
##
## Non missing response frequency for each item
## 1 2 3 4 miss
## Dataset_Missing_Removed.cape_1 0.03 0.43 0.37 0.17 0.00
## Dataset_Missing_Removed.cape_9 0.12 0.40 0.29 0.19 0.01
## Dataset_Missing_Removed.cape_12 0.18 0.39 0.23 0.20 0.02
## Dataset_Missing_Removed.cape_14 0.29 0.41 0.17 0.13 0.01
## Dataset_Missing_Removed.cape_19 0.35 0.33 0.22 0.10 0.01
## Dataset_Missing_Removed.cape_38 0.13 0.34 0.23 0.30 0.01
## Dataset_Missing_Removed.cape_39 0.10 0.31 0.25 0.34 0.01
## Dataset_Missing_Removed.cape_40 0.06 0.36 0.29 0.29 0.01

Identify NAs and create new column with this figure for each observation

apply(CAPE_depressive.missing, 1, function(X) sum(is.na(X)))

## [1] 0 0 0 0 0 0 0 0 0 0 0 0 0 0 0 0 0 0 0 0 0 0 0 0 0 0 0 0 0 0 0 0 0 0 0 0 0
## [38] 0 0 0 0 0 0 0 0 0 0 0 0 0 0 0 0 0 0 0 0 0 0 0 0 0 0 0 0 0 0 0 0 0 0 0 0 0
## [75] 0 0 0 0 0 0 0 0 0 0 0 0 0 0 0 0 0 0 0 0 0 0 0 0 0 0 0 0 0 0 0 0 0 0 0 0 0
## [112] 0 0 0 0 0 0 0 0 1 0 0 0 0 0 0 0 0 0 0 0 0 0 0 0 0 0 0 0 0 0 0 0 0 0 0 0 0
## [149] 0 0 0 0 0 5 0 0 0 0 0 0 0 0 0 0 0 0 1 0 0 0 0 0 0 0 0 0 0 0 0 0 0 0 0 0 0
## [186] 0 0 0 5 0

Dataset_Missing_Removed$CAPE_depressive.missing <- apply(CAPE_depressive.missing, 1, function(X) sum(is.na(X)))

CAPE positive

CAPE_positive.missing <- data.frame( Dataset_Missing_Removed$cape_2, Dataset_Missing_Removed$cape_5, Dataset_Missing_Removed$cape_6, Dataset_Missing_Removed$cape_7, Dataset_Missing_Removed$cape_10, Dataset_Missing_Removed$cape_11, Dataset_Missing_Removed$cape_13, Dataset_Missing_Removed$cape_15, Dataset_Missing_Removed$cape_17, Dataset_Missing_Removed$cape_20, Dataset_Missing_Removed$cape_22, Dataset_Missing_Removed$cape_24, Dataset_Missing_Removed$cape_26, Dataset_Missing_Removed$cape_28, Dataset_Missing_Removed$cape_30, Dataset_Missing_Removed$cape_31, Dataset_Missing_Removed$cape_33, Dataset_Missing_Removed$cape_34, Dataset_Missing_Removed$cape_41, Dataset_Missing_Removed$cape_42)

Cronbach’s alpha

alpha(CAPE_positive.missing)

##
## Reliability analysis
## Call: alpha(x = CAPE_positive.missing)
##
## raw_alpha std.alpha G6(smc) average_r S/N ase mean sd median_r
## 0.93 0.93 0.95 0.41 14 0.0072 2 0.64 0.41
##
## lower alpha upper 95% confidence boundaries
## 0.92 0.93 0.95
##
## Reliability if an item is dropped:
## raw_alpha std.alpha G6(smc) average_r S/N
## Dataset_Missing_Removed.cape_2 0.93 0.93 0.95 0.40 13
## Dataset_Missing_Removed.cape_5 0.93 0.93 0.95 0.41 13
## Dataset_Missing_Removed.cape_6 0.93 0.93 0.95 0.41 13
## Dataset_Missing_Removed.cape_7 0.93 0.93 0.94 0.40 13
## Dataset_Missing_Removed.cape_10 0.92 0.93 0.94 0.40 12
## Dataset_Missing_Removed.cape_11 0.93 0.93 0.95 0.42 14
## Dataset_Missing_Removed.cape_13 0.93 0.93 0.95 0.41 13
## Dataset_Missing_Removed.cape_15 0.93 0.93 0.95 0.41 13
## Dataset_Missing_Removed.cape_17 0.93 0.93 0.95 0.41 13
## Dataset_Missing_Removed.cape_20 0.93 0.93 0.95 0.43 14
## Dataset_Missing_Removed.cape_22 0.93 0.93 0.95 0.41 13
## Dataset_Missing_Removed.cape_24 0.93 0.93 0.94 0.41 13
## Dataset_Missing_Removed.cape_26 0.93 0.93 0.94 0.40 13
## Dataset_Missing_Removed.cape_28 0.93 0.93 0.94 0.40 13
## Dataset_Missing_Removed.cape_30 0.93 0.93 0.95 0.41 13
## Dataset_Missing_Removed.cape_31 0.93 0.93 0.95 0.40 13
## Dataset_Missing_Removed.cape_33 0.93 0.93 0.94 0.41 13
## Dataset_Missing_Removed.cape_34 0.93 0.93 0.95 0.41 13
## Dataset_Missing_Removed.cape_41 0.93 0.93 0.95 0.41 13
## Dataset_Missing_Removed.cape_42 0.93 0.93 0.95 0.40 13
## alpha se var.r med.r
## Dataset_Missing_Removed.cape_2 0.0077 0.015 0.41
## Dataset_Missing_Removed.cape_5 0.0075 0.015 0.41
## Dataset_Missing_Removed.cape_6 0.0076 0.014 0.41
## Dataset_Missing_Removed.cape_7 0.0079 0.014 0.40
## Dataset_Missing_Removed.cape_10 0.0079 0.014 0.40
## Dataset_Missing_Removed.cape_11 0.0073 0.014 0.42
## Dataset_Missing_Removed.cape_13 0.0074 0.014 0.41
## Dataset_Missing_Removed.cape_15 0.0075 0.015 0.41
## Dataset_Missing_Removed.cape_17 0.0075 0.015 0.41
## Dataset_Missing_Removed.cape_20 0.0070 0.011 0.42
## Dataset_Missing_Removed.cape_22 0.0076 0.015 0.41
## Dataset_Missing_Removed.cape_24 0.0076 0.014 0.41
## Dataset_Missing_Removed.cape_26 0.0078 0.014 0.40
## Dataset_Missing_Removed.cape_28 0.0078 0.014 0.40
## Dataset_Missing_Removed.cape_30 0.0076 0.014 0.41
## Dataset_Missing_Removed.cape_31 0.0077 0.015 0.41
## Dataset_Missing_Removed.cape_33 0.0075 0.013 0.41
## Dataset_Missing_Removed.cape_34 0.0075 0.014 0.41
## Dataset_Missing_Removed.cape_41 0.0075 0.015 0.41
## Dataset_Missing_Removed.cape_42 0.0077 0.015 0.40
##
## Item statistics
## n raw.r std.r r.cor r.drop mean sd
## Dataset_Missing_Removed.cape_2 190 0.71 0.71 0.69 0.66 2.4 0.99
## Dataset_Missing_Removed.cape_5 190 0.63 0.64 0.62 0.58 1.9 0.80
## Dataset_Missing_Removed.cape_6 189 0.65 0.65 0.64 0.61 2.4 0.89
## Dataset_Missing_Removed.cape_7 187 0.77 0.77 0.77 0.74 2.3 0.99
## Dataset_Missing_Removed.cape_10 188 0.80 0.80 0.80 0.77 2.1 0.99
## Dataset_Missing_Removed.cape_11 189 0.53 0.54 0.52 0.48 1.9 0.89
## Dataset_Missing_Removed.cape_13 188 0.58 0.58 0.56 0.53 2.2 0.99
## Dataset_Missing_Removed.cape_15 189 0.65 0.65 0.64 0.61 2.0 0.99
## Dataset_Missing_Removed.cape_17 190 0.64 0.64 0.62 0.59 1.8 0.94
## Dataset_Missing_Removed.cape_20 189 0.42 0.41 0.36 0.34 1.9 1.08
## Dataset_Missing_Removed.cape_22 189 0.66 0.66 0.64 0.61 2.2 1.04
## Dataset_Missing_Removed.cape_24 188 0.67 0.66 0.65 0.62 1.8 0.91
## Dataset_Missing_Removed.cape_26 188 0.78 0.77 0.76 0.74 2.0 0.94
## Dataset_Missing_Removed.cape_28 190 0.73 0.74 0.73 0.70 2.1 1.03
## Dataset_Missing_Removed.cape_30 190 0.66 0.66 0.64 0.61 1.9 0.98
## Dataset_Missing_Removed.cape_31 190 0.72 0.71 0.70 0.68 1.9 0.95
## Dataset_Missing_Removed.cape_33 189 0.67 0.65 0.64 0.61 2.2 1.16
## Dataset_Missing_Removed.cape_34 188 0.62 0.61 0.60 0.57 1.8 1.06
## Dataset_Missing_Removed.cape_41 187 0.64 0.64 0.61 0.59 1.4 0.73
## Dataset_Missing_Removed.cape_42 189 0.72 0.72 0.70 0.68 1.9 1.05
##
## Non missing response frequency for each item
## 1 2 3 4 miss
## Dataset_Missing_Removed.cape_2 0.20 0.37 0.27 0.16 0.00
## Dataset_Missing_Removed.cape_5 0.36 0.46 0.13 0.04 0.00
## Dataset_Missing_Removed.cape_6 0.14 0.41 0.32 0.13 0.01
## Dataset_Missing_Removed.cape_7 0.26 0.35 0.26 0.13 0.02
## Dataset_Missing_Removed.cape_10 0.30 0.38 0.19 0.13 0.01
## Dataset_Missing_Removed.cape_11 0.41 0.38 0.15 0.06 0.01
## Dataset_Missing_Removed.cape_13 0.27 0.39 0.20 0.14 0.01
## Dataset_Missing_Removed.cape_15 0.39 0.34 0.16 0.11 0.01
## Dataset_Missing_Removed.cape_17 0.52 0.27 0.14 0.07 0.00
## Dataset_Missing_Removed.cape_20 0.52 0.25 0.08 0.14 0.01
## Dataset_Missing_Removed.cape_22 0.29 0.35 0.20 0.16 0.01
## Dataset_Missing_Removed.cape_24 0.49 0.30 0.15 0.06 0.01
## Dataset_Missing_Removed.cape_26 0.34 0.37 0.20 0.09 0.01
## Dataset_Missing_Removed.cape_28 0.36 0.33 0.19 0.13 0.00
## Dataset_Missing_Removed.cape_30 0.42 0.33 0.15 0.10 0.00
## Dataset_Missing_Removed.cape_31 0.45 0.33 0.15 0.08 0.00
## Dataset_Missing_Removed.cape_33 0.35 0.29 0.13 0.23 0.01
## Dataset_Missing_Removed.cape_34 0.51 0.27 0.09 0.13 0.01
## Dataset_Missing_Removed.cape_41 0.75 0.15 0.07 0.03 0.02
## Dataset_Missing_Removed.cape_42 0.47 0.28 0.13 0.13 0.01

Identify NAs and create new column with this figure for each observation

apply(CAPE_positive.missing, 1, function(X) sum(is.na(X)))

## [1] 0 0 0 0 0 0 0 0 0 0 0 0 0 0 0 0 1 0 0 0 0 0 0 0 0 0 0 0 0 0 0 0 0 0 0 0 0
## [38] 0 0 0 0 0 0 0 0 0 0 0 0 0 0 0 0 0 0 0 0 0 0 0 0 0 0 1 0 0 0 0 0 0 0 0 0 0
## [75] 0 0 1 0 0 0 0 0 0 0 0 0 0 1 1 0 0 0 0 0 0 1 0 0 0 0 0 0 0 0 0 0 0 0 0 0 0
## [112] 0 0 0 0 1 0 0 0 0 0 0 1 0 0 0 0 0 0 0 0 0 0 0 0 0 0 0 0 0 0 0 0 1 0 0 0 0
## [149] 0 1 0 0 1 4 0 0 0 0 0 0 0 0 0 0 0 0 0 0 0 0 0 0 0 0 0 0 0 0 0 0 0 0 0 0 0
## [186] 0 0 0 8 0

Dataset_Missing_Removed$CAPE_positive.missing <- apply(CAPE_positive.missing, 1, function(X) sum(is.na(X)))

change NAs in data frame to 0

Dataset_Missing_Removed[c("cape_1", "cape_2", "cape_3", "cape_4", "cape_5", "cape_6", "cape_7", "cape_8", "cape_9", "cape_10", "cape_11", "cape_12", "cape_13","cape_14", "cape_15", "cape_16", "cape_17", "cape_18", "cape_19", "cape_20", "cape_21", "cape_22", "cape_23", "cape_24", "cape_25","cape_26", "cape_27", "cape_28", "cape_29", "cape_30", "cape_31", "cape_32","cape_33", "cape_34", "cape_35", "cape_36", "cape_37", "cape_38", "cape_39", "cape_40", "cape_41", "cape_42")][is.na(Dataset_Missing_Removed[c("cape_1", "cape_2", "cape_3", "cape_4", "cape_5", "cape_6", "cape_7", "cape_8", "cape_9", "cape_10", "cape_11", "cape_12", "cape_13","cape_14", "cape_15", "cape_16", "cape_17", "cape_18", "cape_19", "cape_20", "cape_21", "cape_22", "cape_23", "cape_24", "cape_25","cape_26", "cape_27", "cape_28", "cape_29", "cape_30", "cape_31", "cape_32", "cape_33", "cape_34", "cape_35", "cape_36", "cape_37", "cape_38", "cape_39", "cape_40", "cape_41", "cape_42")])] <-0

Calculate CAPE subscale scores by adding all completed subscles items and dividing by (the total number of subscale items MINUS the number of subscale items missed)

CAPE negative

Dataset_Missing_Removed %>%
 rowwise() %>%
 mutate(CAPE_negative= sum(c(cape_3, cape_4, cape_8, cape_16, cape_18, cape_21, cape_23, cape_25, cape_27, cape_29, cape_32, cape_35, cape_36, cape_37))/(14- CAPE_negative.missing)) -> Dataset_Missing_Removed

CAPE depressive score

Dataset_Missing_Removed %>%
 rowwise() %>%
 mutate(CAPE_depressive = sum(c(cape_1, cape_9, cape_12, cape_14, cape_19, cape_38, cape_39, cape_40))/(8- CAPE_depressive.missing)) -> Dataset_Missing_Removed

CAPE positive

Dataset_Missing_Removed %>%
 rowwise() %>%
 mutate(CAPE_positive = sum(c(cape_2, cape_5, cape_6, cape_7, cape_10, cape_11, cape_13, cape_15, cape_17, cape_20, cape_22, cape_24, cape_26, cape_28, cape_30, cape_31, cape_33, cape_34, cape_41, cape_42))/(20 - CAPE_positive.missing)) -> Dataset_Missing_Removed

**SWEMWBS**

Create a dataframe to calculate the number of items missing for each participant

SWEMWBS_missing <- data.frame(Dataset_Missing_Removed$swemwbs_1, Dataset_Missing_Removed$swemwbs_2,Dataset_Missing_Removed$swemwbs_3, Dataset_Missing_Removed$swemwbs_4, Dataset_Missing_Removed$swemwbs_5, Dataset_Missing_Removed$swemwbs_6,Dataset_Missing_Removed$swemwbs_7)

Cronbach’s alpha

alpha(SWEMWBS_missing)

##
## Reliability analysis
## Call: alpha(x = SWEMWBS_missing)
##
## raw_alpha std.alpha G6(smc) average_r S/N ase mean sd median_r
## 0.9 0.9 0.89 0.56 8.8 0.011 2.8 0.88 0.55
##
## lower alpha upper 95% confidence boundaries
## 0.88 0.9 0.92
##
## Reliability if an item is dropped:
## raw_alpha std.alpha G6(smc) average_r S/N
## Dataset_Missing_Removed.swemwbs_1 0.88 0.88 0.87 0.55 7.3
## Dataset_Missing_Removed.swemwbs_2 0.89 0.89 0.88 0.57 8.1
## Dataset_Missing_Removed.swemwbs_3 0.88 0.88 0.87 0.55 7.4
## Dataset_Missing_Removed.swemwbs_4 0.87 0.87 0.86 0.54 7.0
## Dataset_Missing_Removed.swemwbs_5 0.88 0.88 0.86 0.54 7.1
## Dataset_Missing_Removed.swemwbs_6 0.90 0.90 0.89 0.59 8.6
## Dataset_Missing_Removed.swemwbs_7 0.88 0.88 0.87 0.56 7.7
## alpha se var.r med.r
## Dataset_Missing_Removed.swemwbs_1 0.014 0.0071 0.55
## Dataset_Missing_Removed.swemwbs_2 0.013 0.0064 0.59
## Dataset_Missing_Removed.swemwbs_3 0.013 0.0064 0.54
## Dataset_Missing_Removed.swemwbs_4 0.014 0.0064 0.54
## Dataset_Missing_Removed.swemwbs_5 0.014 0.0058 0.55
## Dataset_Missing_Removed.swemwbs_6 0.012 0.0046 0.60
## Dataset_Missing_Removed.swemwbs_7 0.013 0.0067 0.55
##
## Item statistics
## n raw.r std.r r.cor r.drop mean sd
## Dataset_Missing_Removed.swemwbs_1 188 0.82 0.81 0.77 0.73 2.7 1.1
## Dataset_Missing_Removed.swemwbs_2 186 0.75 0.75 0.69 0.65 2.7 1.1
## Dataset_Missing_Removed.swemwbs_3 185 0.80 0.80 0.76 0.72 2.6 1.0
## Dataset_Missing_Removed.swemwbs_4 186 0.85 0.85 0.82 0.78 2.9 1.1
## Dataset_Missing_Removed.swemwbs_5 185 0.84 0.83 0.81 0.76 3.0 1.0
## Dataset_Missing_Removed.swemwbs_6 187 0.71 0.70 0.62 0.59 2.8 1.2
## Dataset_Missing_Removed.swemwbs_7 189 0.78 0.78 0.73 0.69 3.2 1.1
##
## Non missing response frequency for each item
## 1 2 3 4 5 miss
## Dataset_Missing_Removed.swemwbs_1 0.16 0.30 0.30 0.16 0.07 0.01
## Dataset_Missing_Removed.swemwbs_2 0.16 0.26 0.34 0.18 0.06 0.02
## Dataset_Missing_Removed.swemwbs_3 0.15 0.31 0.37 0.13 0.04 0.03
## Dataset_Missing_Removed.swemwbs_4 0.13 0.18 0.36 0.26 0.06 0.02
## Dataset_Missing_Removed.swemwbs_5 0.11 0.18 0.42 0.22 0.06 0.03
## Dataset_Missing_Removed.swemwbs_6 0.16 0.26 0.28 0.25 0.05 0.02
## Dataset_Missing_Removed.swemwbs_7 0.07 0.20 0.35 0.27 0.11 0.01

Identify how many NAs are in each observation

apply(SWEMWBS_missing, 1, function(X) sum(is.na(X)))

## [1] 0 0 0 0 0 0 0 0 0 0 0 0 0 0 0 0 0 0 0 0 0 1 0 0 0 0 0 0 0 0 0 0 0 0 0 0 0
## [38] 0 0 0 0 0 0 0 0 0 0 1 0 0 0 0 0 0 1 0 0 0 0 0 0 0 0 0 0 0 0 0 0 0 1 0 0 0
## [75] 0 0 7 0 0 0 0 0 0 0 0 0 0 0 0 0 0 0 0 0 0 0 0 0 0 0 0 0 0 0 0 0 0 0 0 0 0
## [112] 0 0 0 1 0 0 0 0 0 0 0 1 0 0 0 0 3 0 0 0 0 0 0 0 0 0 0 0 0 1 0 0 0 0 0 0 0
## [149] 0 0 0 0 0 0 0 0 0 0 0 0 0 0 0 0 0 0 0 0 0 0 0 1 0 0 1 0 0 0 0 0 0 0 0 0 0
## [186] 0 0 0 5 0

Create a new column that says how many NAs are in each observation, ,i.e. how many items from SWEMWBS each participant is missing

Dataset_Missing_Removed$SWEMWBS_missing <- apply(SWEMWBS_missing, 1, function(X) sum(is.na(X)))

Replace NAs with mean values of other scores

SWEMWBS_missing2 <- SWEMWBS_missing
means_SWEMWBS <- which(is.na(SWEMWBS_missing2), arr.ind = TRUE)
SWEMWBS_missing2[means_SWEMWBS] <- rowMeans(SWEMWBS_missing2, na.rm = TRUE)[means_SWEMWBS[,1]]

Input SWEMWBS_missing2 into main data again

Dataset_Missing_Removed$swemwbs_1 <- SWEMWBS_missing2$Dataset_Missing_Removed.swemwbs_1
Dataset_Missing_Removed$swemwbs_2 <- SWEMWBS_missing2$Dataset_Missing_Removed.swemwbs_2
Dataset_Missing_Removed$swemwbs_3 <- SWEMWBS_missing2$Dataset_Missing_Removed.swemwbs_3
Dataset_Missing_Removed$swemwbs_4 <- SWEMWBS_missing2$Dataset_Missing_Removed.swemwbs_4
Dataset_Missing_Removed$swemwbs_5 <- SWEMWBS_missing2$Dataset_Missing_Removed.swemwbs_5
Dataset_Missing_Removed$swemwbs_6 <- SWEMWBS_missing2$Dataset_Missing_Removed.swemwbs_6
Dataset_Missing_Removed$swemwbs_7 <- SWEMWBS_missing2$Dataset_Missing_Removed.swemwbs_7

Round numbers to nearest whole number

Dataset_Missing_Removed$swemwbs_1 <- floor(0.5 + Dataset_Missing_Removed$swemwbs_1)
Dataset_Missing_Removed$swemwbs_2 <- floor(0.5 + Dataset_Missing_Removed$swemwbs_2)
Dataset_Missing_Removed$swemwbs_3 <- floor(0.5 + Dataset_Missing_Removed$swemwbs_3)
Dataset_Missing_Removed$swemwbs_4 <- floor(0.5 + Dataset_Missing_Removed$swemwbs_4)
Dataset_Missing_Removed$swemwbs_5 <- floor(0.5 + Dataset_Missing_Removed$swemwbs_5)
Dataset_Missing_Removed$swemwbs_6 <- floor(0.5 + Dataset_Missing_Removed$swemwbs_6)
Dataset_Missing_Removed$swemwbs_7 <- floor(0.5 + Dataset_Missing_Removed$swemwbs_7)

Create variable for total of SWEMWBS

Dataset_Missing_Removed %>%
 rowwise() %>%
 mutate(SWEMWBS_total = sum(c(swemwbs_1, swemwbs_2, swemwbs_3, swemwbs_4, swemwbs_5, swemwbs_6, swemwbs_7))) -> Dataset_Missing_Removed

Change raw scores to metric as instructed on [website](https://warwick.ac.uk/fac/sci/med/research/platform/wemwbs/using/howto/) Create copy dataset incase anything goes wrong

SWEMWBS_total_copy <- Dataset_Missing_Removed

Change SWEMWBS total score to a factor so mutate funtion can be used

SWEMWBS_total_copy$SWEMWBS_total <- as.factor(SWEMWBS_total_copy$SWEMWBS_total)

Use mutate to create a new column with converted scored and feed it back into to the data set

SWEMWBS_total_copy %>%
 mutate(SWEMWBS_metric = case_when(SWEMWBS_total == 7 ~ 7.00,
 SWEMWBS_total == 8 ~ 9.51,
 SWEMWBS_total == 9 ~ 11.25,
 SWEMWBS_total == 10 ~ 12.40,
 SWEMWBS_total == 11 ~ 13.33,
 SWEMWBS_total == 12 ~ 14.08,
 SWEMWBS_total == 13 ~ 14.75,
 SWEMWBS_total == 14 ~ 15.32,
 SWEMWBS_total == 15 ~ 15.84,
 SWEMWBS_total == 16 ~ 16.36,
 SWEMWBS_total == 17 ~ 16.88,
 SWEMWBS_total == 18 ~ 17.43,
 SWEMWBS_total == 19 ~ 17.98,
 SWEMWBS_total == 20 ~ 18.59,
 SWEMWBS_total == 21 ~ 19.25,
 SWEMWBS_total == 22 ~ 19.98,
 SWEMWBS_total == 23 ~ 20.73,
 SWEMWBS_total == 24 ~ 21.54,
 SWEMWBS_total == 25 ~ 22.35,
 SWEMWBS_total == 26 ~ 23.21,
 SWEMWBS_total == 27 ~ 24.11,
 SWEMWBS_total == 28 ~ 25.03,
 SWEMWBS_total == 29 ~ 26.02,
 SWEMWBS_total == 30 ~ 27.03,
 SWEMWBS_total == 31 ~ 28.13,
 SWEMWBS_total == 32 ~ 29.31,
 SWEMWBS_total == 33 ~ 30.70,
 SWEMWBS_total == 34 ~ 32.55,
 SWEMWBS_total == 35 ~ 35.00) ) -> SWEMWBS_total_copy

Dataset_Missing_Removed$SWEMWBS_metric <- SWEMWBS_total_copy$SWEMWBS_metric

**ReSta** Two scales used, ReSta A was to be completed by participants who were single, ReSta B was for participants who had a partner.

Cronbach’s alpha - create dataframe to calculate Cronbach’s alpha for Resta A (single subscale)and Resta B (partner subscale)

resta.single <- data.frame(Dataset_Missing_Removed$resta_1a, Dataset_Missing_Removed$resta_2a,Dataset_Missing_Removed$resta_3a, Dataset_Missing_Removed$resta_4a,Dataset_Missing_Removed$resta_5a)

Reverse score item 2 and calculate Cronbach’s alpha

resta.single %>%
 mutate(Dataset_Missing_Removed.resta_2a = c(Dataset_Missing_Removed.resta_2a)* -1) -> resta.single

alpha(resta.single)

##
## Reliability analysis
## Call: alpha(x = resta.single)
##
## raw_alpha std.alpha G6(smc) average_r S/N ase mean sd median_r
## 0.91 0.91 0.9 0.68 11 0.0097 1.5 0.89 0.69
##
## lower alpha upper 95% confidence boundaries
## 0.9 0.91 0.93
##
## Reliability if an item is dropped:
## raw_alpha std.alpha G6(smc) average_r S/N
## Dataset_Missing_Removed.resta_1a 0.89 0.89 0.87 0.67 8.0
## Dataset_Missing_Removed.resta_2a 0.92 0.92 0.90 0.74 11.3
## Dataset_Missing_Removed.resta_3a 0.88 0.88 0.85 0.64 7.1
## Dataset_Missing_Removed.resta_4a 0.91 0.91 0.89 0.70 9.5
## Dataset_Missing_Removed.resta_5a 0.88 0.88 0.86 0.65 7.5
## alpha se var.r med.r
## Dataset_Missing_Removed.resta_1a 0.0131 0.0134 0.68
## Dataset_Missing_Removed.resta_2a 0.0096 0.0030 0.74
## Dataset_Missing_Removed.resta_3a 0.0143 0.0089 0.65
## Dataset_Missing_Removed.resta_4a 0.0112 0.0078 0.71
## Dataset_Missing_Removed.resta_5a 0.0137 0.0088 0.66
##
## Item statistics
## n raw.r std.r r.cor r.drop mean sd
## Dataset_Missing_Removed.resta_1a 110 0.89 0.88 0.85 0.81 2.4 1.10
## Dataset_Missing_Removed.resta_2a 107 0.78 0.78 0.70 0.66 -2.5 1.00
## Dataset_Missing_Removed.resta_3a 107 0.92 0.92 0.91 0.87 2.5 1.06
## Dataset_Missing_Removed.resta_4a 107 0.83 0.83 0.77 0.73 2.7 0.99
## Dataset_Missing_Removed.resta_5a 107 0.90 0.90 0.88 0.84 2.5 1.07
##
## Non missing response frequency for each item
## -4 -3 -2 -1 1 2 3 4 miss
## Dataset_Missing_Removed.resta_1a 0.00 0.00 0.00 0.00 0.25 0.29 0.23 0.23 0.42
## Dataset_Missing_Removed.resta_2a 0.19 0.28 0.35 0.19 0.00 0.00 0.00 0.00 0.44
## Dataset_Missing_Removed.resta_3a 0.00 0.00 0.00 0.00 0.21 0.33 0.24 0.21 0.44
## Dataset_Missing_Removed.resta_4a 0.00 0.00 0.00 0.00 0.11 0.37 0.25 0.26 0.44
## Dataset_Missing_Removed.resta_5a 0.00 0.00 0.00 0.00 0.21 0.30 0.26 0.22 0.44

Create dataframe for Resta B (partner subscale), reverse score item 2 and then calculate Cronbach’s alpha

resta.partner <- data.frame(Dataset_Missing_Removed$resta_1b, Dataset_Missing_Removed$resta_2b, Dataset_Missing_Removed$resta_3b, Dataset_Missing_Removed$resta_4b, Dataset_Missing_Removed$resta_5b)

resta.partner %>%
 mutate(Dataset_Missing_Removed.resta_2b = c(Dataset_Missing_Removed.resta_2b)* -1) -> resta.partner

alpha(resta.partner)

##
## Reliability analysis
## Call: alpha(x = resta.partner)
##
## raw_alpha std.alpha G6(smc) average_r S/N ase mean sd median_r
## 0.92 0.92 0.92 0.7 11 0.0085 2.3 0.76 0.76
##
## lower alpha upper 95% confidence boundaries
## 0.91 0.92 0.94
##
## Reliability if an item is dropped:
## raw_alpha std.alpha G6(smc) average_r S/N
## Dataset_Missing_Removed.resta_1b 0.88 0.88 0.87 0.65 7.3
## Dataset_Missing_Removed.resta_2b 0.95 0.95 0.94 0.82 18.5
## Dataset_Missing_Removed.resta_3b 0.89 0.89 0.89 0.67 8.0
## Dataset_Missing_Removed.resta_4b 0.90 0.90 0.90 0.68 8.7
## Dataset_Missing_Removed.resta_5b 0.89 0.89 0.88 0.66 7.9
## alpha se var.r med.r
## Dataset_Missing_Removed.resta_1b 0.0134 0.0264 0.65
## Dataset_Missing_Removed.resta_2b 0.0063 0.0036 0.83
## Dataset_Missing_Removed.resta_3b 0.0123 0.0300 0.66
## Dataset_Missing_Removed.resta_4b 0.0114 0.0433 0.69
## Dataset_Missing_Removed.resta_5b 0.0125 0.0266 0.65
##
## Item statistics
## n raw.r std.r r.cor r.drop mean sd
## Dataset_Missing_Removed.resta_1b 101 0.94 0.94 0.95 0.91 3.3 0.92
## Dataset_Missing_Removed.resta_2b 101 0.68 0.70 0.57 0.55 -1.6 0.73
## Dataset_Missing_Removed.resta_3b 101 0.92 0.91 0.90 0.86 3.4 0.91
## Dataset_Missing_Removed.resta_4b 101 0.89 0.89 0.85 0.82 3.0 0.92
## Dataset_Missing_Removed.resta_5b 101 0.92 0.92 0.91 0.87 3.4 0.86
##
## Non missing response frequency for each item
## -4 -3 -2 -1 1 2 3 4 miss
## Dataset_Missing_Removed.resta_1b 0.00 0.00 0.00 0.00 0.07 0.10 0.27 0.56 0.47
## Dataset_Missing_Removed.resta_2b 0.01 0.12 0.35 0.52 0.00 0.00 0.00 0.00 0.47
## Dataset_Missing_Removed.resta_3b 0.00 0.00 0.00 0.00 0.07 0.08 0.24 0.61 0.47
## Dataset_Missing_Removed.resta_4b 0.00 0.00 0.00 0.00 0.09 0.13 0.43 0.36 0.47
## Dataset_Missing_Removed.resta_5b 0.00 0.00 0.00 0.00 0.05 0.10 0.27 0.58 0.47

Create another dataframe for Resta A (single subscale) to calculate the number of items missing for each participant

Resta_A_missing <- data.frame(Dataset_Missing_Removed$resta_1a, Dataset_Missing_Removed$resta_2a,
 Dataset_Missing_Removed$resta_3a, Dataset_Missing_Removed$resta_4a,
 Dataset_Missing_Removed$resta_5a)

Identify how many NAs are in each observation

apply(Resta_A_missing, 1, function(X) sum(is.na(X)))

## [1] 5 5 5 5 5 5 5 5 5 5 0 5 0 5 0 5 5 0 0 0 5 5 0 0 0 5 0 0 5 0 0 0 0 0 5 5 0
## [38] 0 0 0 0 0 0 0 0 0 0 0 0 5 5 5 0 5 0 5 0 5 5 5 0 0 5 0 5 0 5 5 0 0 5 4 5 0
## [75] 5 5 0 0 0 0 5 5 5 0 5 5 0 0 0 0 0 0 5 0 0 5 5 5 0 0 0 0 0 5 0 0 0 0 5 5 5
## [112] 0 0 5 0 5 0 5 0 5 0 0 0 0 0 5 0 0 0 5 0 4 5 5 0 5 0 4 0 0 0 0 5 0 5 0 0 5
## [149] 0 0 5 5 0 0 5 5 5 0 0 5 5 0 5 5 0 5 0 5 0 0 0 0 0 5 0 0 0 5 5 5 0 5 0 0 5
## [186] 5 0 0 0 5

Create a new column in original dataframe that says how many NAs are in each observation, ,i.e. how many items from Resta A each participant is missing

Dataset_Missing_Removed$RestaA_missing <- apply(Resta_A_missing, 1, function(X) sum(is.na(X)))

Replace NAs with mean values of other scores

means_RestaA <- which(is.na(Resta_A_missing), arr.ind = TRUE)
Resta_A_missing[means_RestaA] <- rowMeans(Resta_A_missing, na.rm = TRUE)[means_RestaA[,1]]

Add column that says how many items were missing from each participant into this data frame

Resta_A_missing$RestaA_missing <- Dataset_Missing_Removed$RestaA_missing

Replace missing values (NAs) with means in the original data frame for participants who are missing two or fewer items

if(Dataset_Missing_Removed$RestaA_missing < 3) {Dataset_Missing_Removed$resta_1a <- Resta_A_missing$Dataset_Missing_Removed.resta_1a}

## Warning in if (Dataset_Missing_Removed$RestaA_missing < 3) {: the condition has
## length > 1 and only the first element will be used

if(Dataset_Missing_Removed$RestaA_missing < 3) {Dataset_Missing_Removed$resta_2a <- Resta_A_missing$Dataset_Missing_Removed.resta_2a}

## Warning in if (Dataset_Missing_Removed$RestaA_missing < 3) {: the condition has
## length > 1 and only the first element will be used

if(Dataset_Missing_Removed$RestaA_missing < 3) {Dataset_Missing_Removed$resta_3a <- Resta_A_missing$Dataset_Missing_Removed.resta_3a}

## Warning in if (Dataset_Missing_Removed$RestaA_missing < 3) {: the condition has
## length > 1 and only the first element will be used

if(Dataset_Missing_Removed$RestaA_missing < 3) {Dataset_Missing_Removed$resta_4a <- Resta_A_missing$Dataset_Missing_Removed.resta_4a}

## Warning in if (Dataset_Missing_Removed$RestaA_missing < 3) {: the condition has
## length > 1 and only the first element will be used

if(Dataset_Missing_Removed$RestaA_missing < 3) {Dataset_Missing_Removed$resta_5a <- Resta_A_missing$Dataset_Missing_Removed.resta_5a}

## Warning in if (Dataset_Missing_Removed$RestaA_missing < 3) {: the condition has
## length > 1 and only the first element will be used

Create column that calculates the total score for ReSta. This code adjusts the scoring of items so the scale is from 0-3 and item 2 is reversed scored as described by the authors [here](https://link.springer.com/content/pdf/10.1007/s10902-014-9503-x.pdf)

Dataset_Missing_Removed %>%
 rowwise() %>%
 mutate(ReSta_single_total = sum(c(resta_1a -1 , 4 - resta_2a, resta_3a -1, resta_4a -1, resta_5a -1 ))) -> Dataset_Missing_Removed

Run the same code for ReSta B

Create a dataframe to calculate the number of items missing for each participant

Resta_B_missing <- data.frame(Dataset_Missing_Removed$resta_1b, Dataset_Missing_Removed$resta_2b, Dataset_Missing_Removed$resta_3b, Dataset_Missing_Removed$resta_4b, Dataset_Missing_Removed$resta_5b)

Identify how many NAs are in each observation

apply(Resta_B_missing, 1, function(X) sum(is.na(X)))

## [1] 0 0 0 0 0 0 0 0 0 0 5 0 5 0 5 0 0 5 5 5 0 0 5 5 5 0 5 5 0 5 5 5 5 5 0 0 5
## [38] 5 5 5 5 5 0 5 5 5 5 5 5 0 0 0 0 0 5 0 5 0 0 0 0 5 0 5 0 5 0 0 5 5 0 0 0 0
## [75] 0 0 5 5 5 0 0 0 0 5 0 0 0 5 5 0 5 5 0 0 5 0 0 0 5 0 5 5 5 0 0 5 5 5 0 0 0
## [112] 5 5 0 5 0 0 0 5 0 0 5 5 5 5 0 5 5 5 0 5 0 0 0 5 5 0 0 5 5 0 5 0 5 0 5 5 0
## [149] 5 0 0 0 5 0 0 0 0 5 5 0 0 5 0 0 5 0 5 0 0 5 5 0 5 0 5 5 0 0 0 0 5 0 5 5 0
## [186] 0 5 5 5 0

Create a new column in original dataframe that says how many NAs are in each observation

Dataset_Missing_Removed$RestaB_missing <- apply(Resta_B_missing, 1, function(X) sum(is.na(X)))

Replace NAs with mean values of other scores and add in column that says how many items were missing from each participant

means_RestaB <- which(is.na(Resta_B_missing), arr.ind = TRUE)
Resta_B_missing[means_RestaB] <- rowMeans(Resta_B_missing, na.rm = TRUE)[means_RestaB[,1]]

Resta_B_missing$RestaB_missing <- Dataset_Missing_Removed$RestaB_missing

Replace missing values (NAs) in the original data frame with mean scores where participants are missing 2 or fewer items

if(Dataset_Missing_Removed$RestaB_missing < 3) {Dataset_Missing_Removed$resta_1b <- Resta_B_missing$Dataset_Missing_Removed.resta_1b}

## Warning in if (Dataset_Missing_Removed$RestaB_missing < 3) {: the condition has
## length > 1 and only the first element will be used

if(Dataset_Missing_Removed$RestaB_missing < 3) {Dataset_Missing_Removed$resta_2b <- Resta_B_missing$Dataset_Missing_Removed.resta_2b}

## Warning in if (Dataset_Missing_Removed$RestaB_missing < 3) {: the condition has
## length > 1 and only the first element will be used

if(Dataset_Missing_Removed$RestaB_missing < 3) {Dataset_Missing_Removed$resta_3b <- Resta_B_missing$Dataset_Missing_Removed.resta_3b}

## Warning in if (Dataset_Missing_Removed$RestaB_missing < 3) {: the condition has
## length > 1 and only the first element will be used

if(Dataset_Missing_Removed$RestaB_missing < 3) {Dataset_Missing_Removed$resta_4b <- Resta_B_missing$Dataset_Missing_Removed.resta_4b}

## Warning in if (Dataset_Missing_Removed$RestaB_missing < 3) {: the condition has
## length > 1 and only the first element will be used

if(Dataset_Missing_Removed$RestaB_missing < 3) {Dataset_Missing_Removed$resta_5b <- Resta_B_missing$Dataset_Missing_Removed.resta_5b}

## Warning in if (Dataset_Missing_Removed$RestaB_missing < 3) {: the condition has
## length > 1 and only the first element will be used

Create total score for Resta B

Dataset_Missing_Removed %>%
 rowwise() %>%
 mutate(ReSta_partner_total = sum(c(resta_1b -1 , 4 - resta_2b, resta_3b -1, resta_4b -1, resta_5b -1 ))) -> Dataset_Missing_Removed

Create a column that shows ReSta score for both single and partnered participants…

Replace NAs in Resta total columns with zero

DataSet1 <- Dataset_Missing_Removed
DataSet2 <- Dataset_Missing_Removed
test <- DataSet1$ReSta_single_total
test

## [1] NA NA NA NA NA NA NA NA NA NA 7 NA 14 NA 4 NA NA 5 6 14 NA NA 8 5 4
## [26] NA 12 0 NA 14 11 10 5 10 NA NA 4 5 1 4 5 5 7 9 12 11 10 6 10 NA
## [51] NA NA 5 NA 13 NA 11 NA NA NA 5 3 NA 14 NA 0 NA NA 5 6 NA NA NA 9 NA
## [76] NA 13 15 8 2 NA NA NA 3 NA NA 1 15 1 2 0 1 NA 0 14 NA NA NA 7 8
## [101] 9 13 9 NA 8 5 3 5 NA NA NA 15 11 NA 4 NA 14 NA 6 NA 12 11 8 2 5
## [126] NA 9 15 10 NA 0 NA NA NA 13 NA 10 NA 9 8 12 14 NA 7 NA 12 3 NA 2 15
## [151] NA NA 0 15 NA NA NA 11 4 NA NA 1 NA NA 11 NA 12 NA 12 9 4 10 15 NA 8
## [176] 1 1 NA NA NA 6 NA 8 8 NA NA 7 12 2 NA

is.na(test)

## [1] TRUE TRUE TRUE TRUE TRUE TRUE TRUE TRUE TRUE TRUE FALSE TRUE
## [13] FALSE TRUE FALSE TRUE TRUE FALSE FALSE FALSE TRUE TRUE FALSE FALSE
## [25] FALSE TRUE FALSE FALSE TRUE FALSE FALSE FALSE FALSE FALSE TRUE TRUE
## [37] FALSE FALSE FALSE FALSE FALSE FALSE FALSE FALSE FALSE FALSE FALSE FALSE
## [49] FALSE TRUE TRUE TRUE FALSE TRUE FALSE TRUE FALSE TRUE TRUE TRUE
## [61] FALSE FALSE TRUE FALSE TRUE FALSE TRUE TRUE FALSE FALSE TRUE TRUE
## [73] TRUE FALSE TRUE TRUE FALSE FALSE FALSE FALSE TRUE TRUE TRUE FALSE
## [85] TRUE TRUE FALSE FALSE FALSE FALSE FALSE FALSE TRUE FALSE FALSE TRUE
## [97] TRUE TRUE FALSE FALSE FALSE FALSE FALSE TRUE FALSE FALSE FALSE FALSE
## [109] TRUE TRUE TRUE FALSE FALSE TRUE FALSE TRUE FALSE TRUE FALSE TRUE
## [121] FALSE FALSE FALSE FALSE FALSE TRUE FALSE FALSE FALSE TRUE FALSE TRUE
## [133] TRUE TRUE FALSE TRUE FALSE TRUE FALSE FALSE FALSE FALSE TRUE FALSE
## [145] TRUE FALSE FALSE TRUE FALSE FALSE TRUE TRUE FALSE FALSE TRUE TRUE
## [157] TRUE FALSE FALSE TRUE TRUE FALSE TRUE TRUE FALSE TRUE FALSE TRUE
## [169] FALSE FALSE FALSE FALSE FALSE TRUE FALSE FALSE FALSE TRUE TRUE TRUE
## [181] FALSE TRUE FALSE FALSE TRUE TRUE FALSE FALSE FALSE TRUE

which(is.na(test))

## [1] 1 2 3 4 5 6 7 8 9 10 12 14 16 17 21 22 26 29 35
## [20] 36 50 51 52 54 56 58 59 60 63 65 67 68 71 72 73 75 76 81
## [39] 82 83 85 86 93 96 97 98 104 109 110 111 114 116 118 120 126 130 132
## [58] 133 134 136 138 143 145 148 151 152 155 156 157 160 161 163 164 166 168 174
## [77] 178 179 180 182 185 186 190

test[which(is.na(test))]<-0
test

## [1] 0 0 0 0 0 0 0 0 0 0 7 0 14 0 4 0 0 5 6 14 0 0 8 5 4
## [26] 0 12 0 0 14 11 10 5 10 0 0 4 5 1 4 5 5 7 9 12 11 10 6 10 0
## [51] 0 0 5 0 13 0 11 0 0 0 5 3 0 14 0 0 0 0 5 6 0 0 0 9 0
## [76] 0 13 15 8 2 0 0 0 3 0 0 1 15 1 2 0 1 0 0 14 0 0 0 7 8
## [101] 9 13 9 0 8 5 3 5 0 0 0 15 11 0 4 0 14 0 6 0 12 11 8 2 5
## [126] 0 9 15 10 0 0 0 0 0 13 0 10 0 9 8 12 14 0 7 0 12 3 0 2 15
## [151] 0 0 0 15 0 0 0 11 4 0 0 1 0 0 11 0 12 0 12 9 4 10 15 0 8
## [176] 1 1 0 0 0 6 0 8 8 0 0 7 12 2 0

Dataset_Missing_Removed$ReSta_single_total[which(is.na(DataSet1$ReSta_single_total))] <- 0

test2 <- DataSet2$ReSta_partner_total
test2

## [1] 15 14 15 13 10 10 14 13 15 14 NaN 14 NaN 14 NaN 5 13 NaN
## [19] NaN NaN 1 15 NaN NaN NaN 12 NaN NaN 15 NaN NaN NaN NaN NaN 15 10
## [37] NaN NaN NaN NaN NaN NaN 13 NaN NaN NaN NaN NaN NaN 15 11 15 8 15
## [55] NaN 15 NaN 14 14 9 13 NaN 14 NaN 10 NaN 15 15 NaN NaN 14 9
## [73] 15 0 13 15 NaN NaN NaN 7 12 10 10 NaN 15 15 5 NaN NaN 9
## [91] NaN NaN 15 10 NaN 12 15 12 NaN 7 NaN NaN NaN 10 10 NaN NaN NaN
## [109] 10 14 15 NaN NaN 12 NaN 7 6 13 NaN 13 12 NaN NaN NaN NaN 15
## [127] NaN NaN NaN 15 NaN 13 15 11 NaN NaN 7 10 NaN NaN 5 NaN 14 NaN
## [145] 4 NaN NaN 14 NaN 3 8 15 NaN 3 10 6 13 NaN NaN 14 15 NaN
## [163] 15 14 NaN 13 NaN 15 8 NaN NaN 6 NaN 14 NaN NaN 3 10 3 15
## [181] NaN 13 NaN NaN 15 15 NaN NaN NaN 12

is.na(test2)

## [1] FALSE FALSE FALSE FALSE FALSE FALSE FALSE FALSE FALSE FALSE TRUE FALSE
## [13] TRUE FALSE TRUE FALSE FALSE TRUE TRUE TRUE FALSE FALSE TRUE TRUE
## [25] TRUE FALSE TRUE TRUE FALSE TRUE TRUE TRUE TRUE TRUE FALSE FALSE
## [37] TRUE TRUE TRUE TRUE TRUE TRUE FALSE TRUE TRUE TRUE TRUE TRUE
## [49] TRUE FALSE FALSE FALSE FALSE FALSE TRUE FALSE TRUE FALSE FALSE FALSE
## [61] FALSE TRUE FALSE TRUE FALSE TRUE FALSE FALSE TRUE TRUE FALSE FALSE
## [73] FALSE FALSE FALSE FALSE TRUE TRUE TRUE FALSE FALSE FALSE FALSE TRUE
## [85] FALSE FALSE FALSE TRUE TRUE FALSE TRUE TRUE FALSE FALSE TRUE FALSE
## [97] FALSE FALSE TRUE FALSE TRUE TRUE TRUE FALSE FALSE TRUE TRUE TRUE
## [109] FALSE FALSE FALSE TRUE TRUE FALSE TRUE FALSE FALSE FALSE TRUE FALSE
## [121] FALSE TRUE TRUE TRUE TRUE FALSE TRUE TRUE TRUE FALSE TRUE FALSE
## [133] FALSE FALSE TRUE TRUE FALSE FALSE TRUE TRUE FALSE TRUE FALSE TRUE
## [145] FALSE TRUE TRUE FALSE TRUE FALSE FALSE FALSE TRUE FALSE FALSE FALSE
## [157] FALSE TRUE TRUE FALSE FALSE TRUE FALSE FALSE TRUE FALSE TRUE FALSE
## [169] FALSE TRUE TRUE FALSE TRUE FALSE TRUE TRUE FALSE FALSE FALSE FALSE
## [181] TRUE FALSE TRUE TRUE FALSE FALSE TRUE TRUE TRUE FALSE

which(is.na(test2))

## [1] 11 13 15 18 19 20 23 24 25 27 28 30 31 32 33 34 37 38 39
## [20] 40 41 42 44 45 46 47 48 49 55 57 62 64 66 69 70 77 78 79
## [39] 84 88 89 91 92 95 99 101 102 103 106 107 108 112 113 115 119 122 123
## [58] 124 125 127 128 129 131 135 136 139 140 142 144 146 147 149 153 158 159 162
## [77] 165 167 170 171 173 175 176 181 183 184 187 188 189

test[which(is.na(test2))]<-0
test2

## [1] 15 14 15 13 10 10 14 13 15 14 NaN 14 NaN 14 NaN 5 13 NaN
## [19] NaN NaN 1 15 NaN NaN NaN 12 NaN NaN 15 NaN NaN NaN NaN NaN 15 10
## [37] NaN NaN NaN NaN NaN NaN 13 NaN NaN NaN NaN NaN NaN 15 11 15 8 15
## [55] NaN 15 NaN 14 14 9 13 NaN 14 NaN 10 NaN 15 15 NaN NaN 14 9
## [73] 15 0 13 15 NaN NaN NaN 7 12 10 10 NaN 15 15 5 NaN NaN 9
## [91] NaN NaN 15 10 NaN 12 15 12 NaN 7 NaN NaN NaN 10 10 NaN NaN NaN
## [109] 10 14 15 NaN NaN 12 NaN 7 6 13 NaN 13 12 NaN NaN NaN NaN 15
## [127] NaN NaN NaN 15 NaN 13 15 11 NaN NaN 7 10 NaN NaN 5 NaN 14 NaN
## [145] 4 NaN NaN 14 NaN 3 8 15 NaN 3 10 6 13 NaN NaN 14 15 NaN
## [163] 15 14 NaN 13 NaN 15 8 NaN NaN 6 NaN 14 NaN NaN 3 10 3 15
## [181] NaN 13 NaN NaN 15 15 NaN NaN NaN 12

Dataset_Missing_Removed$ReSta_partner_total[which(is.na(Dataset_Missing_Removed$ReSta_partner_total))] <- 0

Create a new dataframe to work in so if something goes wrong it doesn’t matter

Resta_dataframe <- data.frame(Dataset_Missing_Removed$R_Status_simplified, Dataset_Missing_Removed$ReSta_partner_total,
 Dataset_Missing_Removed$ReSta_single_total, Dataset_Missing_Removed$RestaA_missing,
 Dataset_Missing_Removed$RestaB_missing)

Change names of columns in new data frame so they are easily understandable

names(Resta_dataframe)[names(Resta_dataframe) == "Dataset_Missing_Removed.RestaA_missing"]<- "single.missing"
names(Resta_dataframe)[names(Resta_dataframe) == "Dataset_Missing_Removed.RestaB_missing"]<- "partner.missing"
names(Resta_dataframe)[names(Resta_dataframe) == "Dataset_Missing_Removed.R_Status_simplified"]<- "relationship.status"
names(Resta_dataframe)[names(Resta_dataframe) == "Dataset_Missing_Removed.ReSta_partner_total"]<- "ReSta_partner_total"
names(Resta_dataframe)[names(Resta_dataframe) == "Dataset_Missing_Removed.ReSta_single_total"]<- "ReSta_single_total"

Code to populates the column ‘resta.total’ depending on participants’ relationship status, single or patner.

Resta_dataframe <- mutate(Resta_dataframe, Resta.total = ifelse(grepl("partner", relationship.status), ReSta_partner_total,
 ifelse(grepl("single", relationship.status), ReSta_single_total, "NA")))

Code below is to populate ‘resta.total’ column where relationship status is not classified as single or partner, e.g. those who are dating, widowed, or did not state their relationship status. ‘Dating’ is ambiguous - participant classified based on which ReSta scale they had completed. If they completed both the ReSta A (single) scale will be used as this aligns with how the item was worded to participants (Dating or seeing someone but not ‘officially’ in a relationship). The same code for widowed , separated and participants who self-described their relationship status

Resta_dataframe <- mutate(Resta_dataframe, Resta.total.NA = case_when(
 relationship.status == "dating" & single.missing < 3 & partner.missing == 5 ~ ReSta_single_total,
 relationship.status == "dating" & partner.missing < 3 & single.missing == 5 ~ ReSta_partner_total,
 relationship.status == "dating" & partner.missing < 3 & single.missing < 3 ~ ReSta_single_total,
 relationship.status == "widowed" & single.missing < 3 & partner.missing == 5 ~ ReSta_single_total,
 relationship.status == "widowed" & partner.missing < 3 & single.missing == 5 ~ ReSta_partner_total,
 relationship.status == "widowed" & partner.missing < 3 & single.missing < 3 ~ ReSta_single_total,
 relationship.status == "separated" & single.missing < 3 & partner.missing == 5 ~ ReSta_single_total,
 relationship.status == "separated" & partner.missing < 3 & single.missing == 5 ~ ReSta_partner_total,
 relationship.status == "separated" & partner.missing < 3 & single.missing < 3 ~ ReSta_single_total,
 relationship.status == "self describe" & single.missing < 3 & partner.missing == 5 ~ ReSta_single_total,
 relationship.status == "self describe" & partner.missing < 3 & single.missing == 5 ~ ReSta_partner_total,
 relationship.status == "self describe" & partner.missing < 3 & single.missing < 3 ~ ReSta_single_total,
 is.na(relationship.status) & partner.missing < 3 & single.missing == 5 ~ ReSta_partner_total,
 is.na(relationship.status) & single.missing < 3 & partner.missing == 5 ~ ReSta_single_total,
 is.na(relationship.status) & partner.missing < 3 & single.missing < 3 ~ ReSta_single_total))

Add the above code into the Resta.total column

Resta_dataframe <- mutate(Resta_dataframe, Resta.total2 = ifelse(grepl("partner", relationship.status), ReSta_partner_total, ifelse(grepl("single", relationship.status), ReSta_single_total, Resta.total.NA)))

Finally, add this column back into the main data set

Dataset_Missing_Removed$Resta.total <- Resta_dataframe$Resta.total2

**Loneliness** Cronbach’s alpha

loneliness <- data.frame(Dataset_Missing_Removed$loneliness_1,
 Dataset_Missing_Removed$loneliness_2,
 Dataset_Missing_Removed$loneliness_3)

alpha(loneliness)

##
## Reliability analysis
## Call: alpha(x = loneliness)
##
## raw_alpha std.alpha G6(smc) average_r S/N ase mean sd median_r
## 0.81 0.81 0.77 0.59 4.3 0.025 2.2 0.64 0.51
##
## lower alpha upper 95% confidence boundaries
## 0.76 0.81 0.86
##
## Reliability if an item is dropped:
## raw_alpha std.alpha G6(smc) average_r S/N
## Dataset_Missing_Removed.loneliness_1 0.86 0.86 0.75 0.75 6.1
## Dataset_Missing_Removed.loneliness_2 0.66 0.66 0.50 0.50 2.0
## Dataset_Missing_Removed.loneliness_3 0.68 0.68 0.51 0.51 2.1
## alpha se var.r med.r
## Dataset_Missing_Removed.loneliness_1 0.020 NA 0.75
## Dataset_Missing_Removed.loneliness_2 0.049 NA 0.50
## Dataset_Missing_Removed.loneliness_3 0.047 NA 0.51
##
## Item statistics
## n raw.r std.r r.cor r.drop mean sd
## Dataset_Missing_Removed.loneliness_1 184 0.79 0.79 0.58 0.54 2.1 0.76
## Dataset_Missing_Removed.loneliness_2 186 0.89 0.89 0.83 0.73 2.2 0.77
## Dataset_Missing_Removed.loneliness_3 186 0.88 0.88 0.82 0.72 2.3 0.72
##
## Non missing response frequency for each item
## 1 2 3 miss
## Dataset_Missing_Removed.loneliness_1 0.24 0.42 0.34 0.03
## Dataset_Missing_Removed.loneliness_2 0.22 0.39 0.39 0.02
## Dataset_Missing_Removed.loneliness_3 0.16 0.41 0.43 0.02

To create composite variable items are summed to give total score.

Dataset_Missing_Removed %>%
 rowwise() %>%
 mutate(LonelinessTotal = sum(c(loneliness_1, loneliness_2, loneliness_3))) -> Dataset_Missing_Removed

**Internalised stigma** The ISMI-10 contains 10 items which produce a total score. Reverse-code items 2 and 9 before calculating the total score. Add the item scores together and then divide by the total number of answered items. The resulting score should range from 1-4.

Calculate Cronbach’s alpha

ismi <- data.frame(Dataset_Missing_Removed$ismi_1, Dataset_Missing_Removed$ismi_2,
 Dataset_Missing_Removed$ismi_3, Dataset_Missing_Removed$ismi_4,
 Dataset_Missing_Removed$ismi_5, Dataset_Missing_Removed$ismi_6,
 Dataset_Missing_Removed$ismi_7, Dataset_Missing_Removed$ismi_8,
 Dataset_Missing_Removed$ismi_9, Dataset_Missing_Removed$ismi_10)

ismi %>%
 mutate(Dataset_Missing_Removed.ismi_2 = c(Dataset_Missing_Removed.ismi_2)* -1,
 Dataset_Missing_Removed.ismi_9 = c(Dataset_Missing_Removed.ismi_9)* -1,) -> ismi

alpha(ismi)

##
## Reliability analysis
## Call: alpha(x = ismi)
##
## raw_alpha std.alpha G6(smc) average_r S/N ase mean sd median_r
## 0.83 0.82 0.85 0.32 4.6 0.018 1.4 0.57 0.34
##
## lower alpha upper 95% confidence boundaries
## 0.79 0.83 0.86
##
## Reliability if an item is dropped:
## raw_alpha std.alpha G6(smc) average_r S/N
## Dataset_Missing_Removed.ismi_1 0.84 0.83 0.85 0.35 4.9
## Dataset_Missing_Removed.ismi_2 0.83 0.83 0.85 0.35 4.8
## Dataset_Missing_Removed.ismi_3 0.81 0.80 0.83 0.31 4.1
## Dataset_Missing_Removed.ismi_4 0.81 0.80 0.83 0.31 4.0
## Dataset_Missing_Removed.ismi_5 0.79 0.78 0.81 0.29 3.6
## Dataset_Missing_Removed.ismi_6 0.82 0.81 0.83 0.32 4.2
## Dataset_Missing_Removed.ismi_7 0.80 0.80 0.81 0.30 3.9
## Dataset_Missing_Removed.ismi_8 0.80 0.79 0.82 0.29 3.7
## Dataset_Missing_Removed.ismi_9 0.81 0.81 0.83 0.32 4.1
## Dataset_Missing_Removed.ismi_10 0.81 0.80 0.83 0.31 4.1
## alpha se var.r med.r
## Dataset_Missing_Removed.ismi_1 0.017 0.022 0.37
## Dataset_Missing_Removed.ismi_2 0.018 0.025 0.37
## Dataset_Missing_Removed.ismi_3 0.020 0.026 0.33
## Dataset_Missing_Removed.ismi_4 0.021 0.026 0.33
## Dataset_Missing_Removed.ismi_5 0.022 0.026 0.32
## Dataset_Missing_Removed.ismi_6 0.019 0.030 0.36
## Dataset_Missing_Removed.ismi_7 0.021 0.025 0.33
## Dataset_Missing_Removed.ismi_8 0.022 0.028 0.30
## Dataset_Missing_Removed.ismi_9 0.020 0.029 0.35
## Dataset_Missing_Removed.ismi_10 0.020 0.028 0.33
##
## Item statistics
## n raw.r std.r r.cor r.drop mean sd
## Dataset_Missing_Removed.ismi_1 187 0.37 0.39 0.30 0.23 1.7 0.77
## Dataset_Missing_Removed.ismi_2 187 0.41 0.44 0.35 0.29 -3.2 0.75
## Dataset_Missing_Removed.ismi_3 187 0.63 0.63 0.58 0.53 2.8 0.97
## Dataset_Missing_Removed.ismi_4 187 0.67 0.67 0.62 0.57 3.0 0.92
## Dataset_Missing_Removed.ismi_5 185 0.79 0.77 0.76 0.70 2.4 0.97
## Dataset_Missing_Removed.ismi_6 187 0.59 0.59 0.53 0.47 2.6 0.92
## Dataset_Missing_Removed.ismi_7 187 0.70 0.70 0.68 0.61 2.7 0.91
## Dataset_Missing_Removed.ismi_8 187 0.74 0.75 0.73 0.67 1.9 0.94
## Dataset_Missing_Removed.ismi_9 185 0.62 0.62 0.56 0.50 -2.9 0.95
## Dataset_Missing_Removed.ismi_10 187 0.63 0.63 0.59 0.52 2.6 0.92
##
## Non missing response frequency for each item
## -4 -3 -2 -1 1 2 3 4 miss
## Dataset_Missing_Removed.ismi_1 0.00 0.00 0.00 0.00 0.45 0.41 0.11 0.03 0.02
## Dataset_Missing_Removed.ismi_2 0.37 0.48 0.12 0.03 0.00 0.00 0.00 0.00 0.02
## Dataset_Missing_Removed.ismi_3 0.00 0.00 0.00 0.00 0.11 0.24 0.36 0.29 0.02
## Dataset_Missing_Removed.ismi_4 0.00 0.00 0.00 0.00 0.07 0.20 0.39 0.34 0.02
## Dataset_Missing_Removed.ismi_5 0.00 0.00 0.00 0.00 0.21 0.34 0.31 0.14 0.03
## Dataset_Missing_Removed.ismi_6 0.00 0.00 0.00 0.00 0.10 0.37 0.32 0.20 0.02
## Dataset_Missing_Removed.ismi_7 0.00 0.00 0.00 0.00 0.10 0.28 0.41 0.21 0.02
## Dataset_Missing_Removed.ismi_8 0.00 0.00 0.00 0.00 0.40 0.35 0.17 0.08 0.02
## Dataset_Missing_Removed.ismi_9 0.29 0.39 0.22 0.10 0.00 0.00 0.00 0.00 0.03
## Dataset_Missing_Removed.ismi_10 0.00 0.00 0.00 0.00 0.13 0.29 0.41 0.17 0.02

Calculate total ISMI score for each participant

First, create another dataframe to calculate the number of ISMI missing for each participant

ISMI.missing <- data.frame(Dataset_Missing_Removed$ismi_1, Dataset_Missing_Removed$ismi_2, Dataset_Missing_Removed$ismi_3, Dataset_Missing_Removed$ismi_4, Dataset_Missing_Removed$ismi_5, Dataset_Missing_Removed$ismi_6, Dataset_Missing_Removed$ismi_7, Dataset_Missing_Removed$ismi_8, Dataset_Missing_Removed$ismi_9, Dataset_Missing_Removed$ismi_10)

Identify how many NAs are in each observation and create a new column to show this

apply(ISMI.missing, 1, function(X) sum(is.na(X)))

## [1] 0 0 0 0 0 0 0 0 0 0 0 0 0 0 0 0 0 0 0 0 0 0 0 0 0
## [26] 0 0 0 0 1 0 0 0 0 0 0 0 0 0 0 0 0 0 0 0 0 0 0 0 0
## [51] 0 0 0 0 0 0 0 0 0 0 0 0 0 0 0 0 0 0 0 0 0 0 0 0 0
## [76] 0 0 0 0 0 0 0 0 0 0 0 0 0 0 0 0 0 0 0 0 0 0 0 0 0
## [101] 0 0 0 0 0 1 0 0 0 0 0 0 0 0 0 0 0 0 0 0 0 0 0 0 0
## [126] 0 0 0 0 0 0 0 10 0 0 10 0 0 0 0 0 0 0 0 0 10 0 0 0 0
## [151] 0 0 0 1 0 0 0 0 0 0 0 0 0 0 0 0 0 0 0 0 0 0 0 0 0
## [176] 0 0 0 1 0 0 0 0 0 0 0 0 0 0 0

Dataset_Missing_Removed$ISMI.missing <- apply(ISMI.missing, 1, function(X) sum(is.na(X)))

Change NAs in ISMI items to 0

Dataset_Missing_Removed[c("ismi_1", "ismi_2", "ismi_3", "ismi_4", "ismi_5", "ismi_6", "ismi_7", "ismi_8", "ismi_9", "ismi_10")][is.na(Dataset_Missing_Removed[c("ismi_1", "ismi_2", "ismi_3", "ismi_4", "ismi_5", "ismi_6", "ismi_7", "ismi_8", "ismi_9", "ismi_10")])] <-0

Calculate total scores

Dataset_Missing_Removed %>%
 rowwise() %>%
 mutate(ISMI_total = sum(c(ismi_1, 5 -ismi_2, ismi_3, ismi_4, ismi_5, ismi_6, ismi_7, ismi_8, 5 - ismi_9, ismi_10))/(10 - ISMI.missing)) -> Dataset_Missing_Removed

**Perceived social support** MSPSS has three subscales - family, friends and significant other. For the analysis only the family and friends subscales were used. Info about scoring can be found [here](https://gzimet.wixsite.com/mspss)

Create dataframe for family subscale, calculate Cronbach’s alpha and identify how many missing items (NAs) each participant has

mspss.fam <- data.frame(Dataset_Missing_Removed$mspss_3, Dataset_Missing_Removed$mspss_4,Dataset_Missing_Removed$mspss_8, Dataset_Missing_Removed$mspss_11)

alpha(mspss.fam)

##
## Reliability analysis
## Call: alpha(x = mspss.fam)
##
## raw_alpha std.alpha G6(smc) average_r S/N ase mean sd median_r
## 0.92 0.92 0.91 0.75 12 0.009 4.3 1.9 0.78
##
## lower alpha upper 95% confidence boundaries
## 0.91 0.92 0.94
##
## Reliability if an item is dropped:
## raw_alpha std.alpha G6(smc) average_r S/N
## Dataset_Missing_Removed.mspss_3 0.93 0.93 0.89 0.81 12.6
## Dataset_Missing_Removed.mspss_4 0.88 0.88 0.84 0.71 7.2
## Dataset_Missing_Removed.mspss_8 0.90 0.90 0.87 0.75 9.0
## Dataset_Missing_Removed.mspss_11 0.90 0.90 0.87 0.75 8.9
## alpha se var.r med.r
## Dataset_Missing_Removed.mspss_3 0.0092 0.00022 0.80
## Dataset_Missing_Removed.mspss_4 0.0153 0.00650 0.68
## Dataset_Missing_Removed.mspss_8 0.0126 0.00436 0.77
## Dataset_Missing_Removed.mspss_11 0.0128 0.00852 0.77
##
## Item statistics
## n raw.r std.r r.cor r.drop mean sd
## Dataset_Missing_Removed.mspss_3 181 0.85 0.86 0.78 0.75 4.9 2.0
## Dataset_Missing_Removed.mspss_4 181 0.94 0.94 0.93 0.89 4.1 2.1
## Dataset_Missing_Removed.mspss_8 181 0.91 0.91 0.87 0.83 4.0 2.0
## Dataset_Missing_Removed.mspss_11 180 0.91 0.91 0.87 0.83 4.4 2.1
##
## Non missing response frequency for each item
## 1 2 3 4 5 6 7 miss
## Dataset_Missing_Removed.mspss_3 0.09 0.08 0.09 0.09 0.15 0.28 0.23 0.05
## Dataset_Missing_Removed.mspss_4 0.19 0.08 0.13 0.10 0.15 0.20 0.15 0.05
## Dataset_Missing_Removed.mspss_8 0.20 0.10 0.11 0.09 0.22 0.17 0.10 0.05
## Dataset_Missing_Removed.mspss_11 0.17 0.08 0.07 0.09 0.19 0.22 0.18 0.05

apply(mspss.fam, 1, function(X) sum(is.na(X)))

## [1] 0 0 0 0 0 0 0 0 0 0 0 0 0 0 0 0 0 0 0 0 0 0 0 0 0 0 0 0 0 0 0 0 0 0 0 0 0
## [38] 0 0 0 0 0 0 0 0 0 0 4 0 0 0 0 0 0 0 0 0 0 0 0 0 0 0 0 0 4 0 0 0 0 0 0 0 0
## [75] 0 0 0 0 0 0 0 0 0 0 0 0 0 0 0 0 0 0 0 0 0 0 0 0 0 0 0 0 0 0 0 0 0 0 0 0 0
## [112] 0 0 0 0 0 4 0 0 0 0 0 4 0 4 0 0 0 0 0 0 0 4 0 0 4 0 0 0 0 1 0 0 0 0 4 0 0
## [149] 0 0 0 0 0 0 0 0 0 0 0 0 0 0 0 0 0 0 0 0 0 0 0 0 0 0 0 0 0 0 0 0 0 0 0 0 0
## [186] 0 0 0 4 0

Create a new column in original dataframe that says how many NAs are in each observation

Dataset_Missing_Removed$mspss.fam_missing <- apply(mspss.fam, 1, function(X) sum(is.na(X)))

Replace NAs with mean values of other scores

means_mspss.fam <- which(is.na(mspss.fam), arr.ind = TRUE)
mspss.fam[means_mspss.fam] <- rowMeans(mspss.fam, na.rm = TRUE)[means_mspss.fam[,1]]

Calculate total score for family and add into main data frame

mspss.fam %>%
 rowwise() %>%
 mutate(MSPSS_fam = sum(c(Dataset_Missing_Removed.mspss_3, Dataset_Missing_Removed.mspss_4, Dataset_Missing_Removed.mspss_8, Dataset_Missing_Removed.mspss_11))) -> mspss.fam

Dataset_Missing_Removed$mspss.fam <- mspss.fam$MSPSS_fam

Repeat for friends subscale

mspss.friends <- data.frame(Dataset_Missing_Removed$mspss_6, Dataset_Missing_Removed$mspss_7,Dataset_Missing_Removed$mspss_9, Dataset_Missing_Removed$mspss_12)

alpha(mspss.friends)

##
## Reliability analysis
## Call: alpha(x = mspss.friends)
##
## raw_alpha std.alpha G6(smc) average_r S/N ase mean sd median_r
## 0.94 0.95 0.93 0.81 17 0.0065 4.4 1.7 0.82
##
## lower alpha upper 95% confidence boundaries
## 0.93 0.94 0.96
##
## Reliability if an item is dropped:
## raw_alpha std.alpha G6(smc) average_r S/N
## Dataset_Missing_Removed.mspss_6 0.93 0.93 0.90 0.82 14
## Dataset_Missing_Removed.mspss_7 0.92 0.92 0.89 0.80 12
## Dataset_Missing_Removed.mspss_9 0.92 0.93 0.89 0.81 12
## Dataset_Missing_Removed.mspss_12 0.93 0.93 0.90 0.82 14
## alpha se var.r med.r
## Dataset_Missing_Removed.mspss_6 0.0084 0.00011 0.83
## Dataset_Missing_Removed.mspss_7 0.0098 0.00062 0.81
## Dataset_Missing_Removed.mspss_9 0.0095 0.00096 0.82
## Dataset_Missing_Removed.mspss_12 0.0085 0.00013 0.82
##
## Item statistics
## n raw.r std.r r.cor r.drop mean sd
## Dataset_Missing_Removed.mspss_6 181 0.91 0.92 0.88 0.85 4.6 1.7
## Dataset_Missing_Removed.mspss_7 180 0.94 0.94 0.91 0.89 4.3 1.8
## Dataset_Missing_Removed.mspss_9 181 0.93 0.93 0.90 0.88 4.6 1.9
## Dataset_Missing_Removed.mspss_12 179 0.93 0.92 0.88 0.86 4.3 1.9
##
## Non missing response frequency for each item
## 1 2 3 4 5 6 7 miss
## Dataset_Missing_Removed.mspss_6 0.08 0.06 0.09 0.19 0.25 0.20 0.12 0.05
## Dataset_Missing_Removed.mspss_7 0.11 0.09 0.12 0.20 0.21 0.16 0.12 0.05
## Dataset_Missing_Removed.mspss_9 0.10 0.07 0.09 0.13 0.23 0.22 0.16 0.05
## Dataset_Missing_Removed.mspss_12 0.13 0.10 0.07 0.16 0.22 0.18 0.14 0.06

apply(mspss.friends, 1, function(X) sum(is.na(X)))

## [1] 0 0 0 0 0 0 0 0 0 0 0 0 0 0 0 0 0 0 0 0 0 0 0 0 0 0 0 0 0 0 0 0 0 0 0 0 0
## [38] 0 0 1 0 0 0 0 0 0 0 4 0 0 0 0 0 0 0 0 0 0 0 0 0 0 0 0 0 4 0 0 0 0 0 0 0 0
## [75] 0 0 0 0 0 0 0 0 0 0 0 0 0 0 0 0 0 0 0 0 1 0 0 0 0 0 0 0 0 0 0 0 0 0 0 0 0
## [112] 0 0 0 0 0 4 0 0 0 0 0 4 0 4 0 0 0 0 0 0 0 4 0 0 4 0 0 0 0 1 0 0 0 0 4 0 0
## [149] 0 0 0 0 0 0 0 0 0 0 0 0 0 0 0 0 0 0 0 0 0 0 0 0 0 0 0 0 0 0 0 0 0 0 0 0 0
## [186] 0 0 0 4 0

Dataset_Missing_Removed$mspss.fri_missing <- apply(mspss.friends, 1, function(X) sum(is.na(X)))

means_mspss.fri <- which(is.na(mspss.friends), arr.ind = TRUE)
mspss.friends[means_mspss.fri] <- rowMeans(mspss.friends, na.rm = TRUE)[means_mspss.fri[,1]]

mspss.friends %>%
 rowwise() %>%
 mutate(MSPSS_fri = sum(c(Dataset_Missing_Removed.mspss_6, Dataset_Missing_Removed.mspss_7, Dataset_Missing_Removed.mspss_9, Dataset_Missing_Removed.mspss_12))) -> mspss.friends

Dataset_Missing_Removed$mspss.fri <- mspss.friends$MSPSS_fri

Calculate total perceieved social support score (friends and family only)

Dataset_Missing_Removed %>%
 rowwise()%>%
 mutate(mspss_ff = sum(c(mspss.fam, mspss.fri)/8)) -> Dataset_Missing_Removed

**Self-esteem**

Items 1,3,9,10,13,15,16,17,18 and 20 of the SERS-SF are scored as minus numbers, scores in these columns to negative numbers

Calculate Cronbach’s alpha. Create a dataframe with all SERS items and reverse score approrpriate items before calculating Cronbach’s alpha

sers <- data.frame(Dataset_Missing_Removed$sers_2,Dataset_Missing_Removed$sers_4, Dataset_Missing_Removed$sers_5, Dataset_Missing_Removed$sers_6,
Dataset_Missing_Removed$sers_7, Dataset_Missing_Removed$sers_8,
Dataset_Missing_Removed$sers_11, Dataset_Missing_Removed$sers_12,
Dataset_Missing_Removed$sers_14, Dataset_Missing_Removed$sers_19, Dataset_Missing_Removed$sers_1, Dataset_Missing_Removed$sers_3,
Dataset_Missing_Removed$sers_9, Dataset_Missing_Removed$sers_10,
Dataset_Missing_Removed$sers_13, Dataset_Missing_Removed$sers_15, Dataset_Missing_Removed$sers_16, Dataset_Missing_Removed$sers_17, Dataset_Missing_Removed$sers_18, Dataset_Missing_Removed$sers_20)

sers %>%
 mutate(Dataset_Missing_Removed.sers_1 = c(Dataset_Missing_Removed.sers_1)* -1,
 Dataset_Missing_Removed.sers_3 = c(Dataset_Missing_Removed.sers_3)* -1,
 Dataset_Missing_Removed.sers_9 = c(Dataset_Missing_Removed.sers_9)* -1,
 Dataset_Missing_Removed.sers_10 = c(Dataset_Missing_Removed.sers_10)* -1,
 Dataset_Missing_Removed.sers_13 = c(Dataset_Missing_Removed.sers_13)* -1,
 Dataset_Missing_Removed.sers_15 = c(Dataset_Missing_Removed.sers_15)* -1,
 Dataset_Missing_Removed.sers_16 = c(Dataset_Missing_Removed.sers_16)* -1,
 Dataset_Missing_Removed.sers_17 = c(Dataset_Missing_Removed.sers_17)* -1,
 Dataset_Missing_Removed.sers_18 = c(Dataset_Missing_Removed.sers_18)* -1,
 Dataset_Missing_Removed.sers_20 = c(Dataset_Missing_Removed.sers_20)* -1) -> sers


alpha(sers)

## Number of categories should be increased in order to count frequencies.

##
## Reliability analysis
## Call: alpha(x = sers)
##
## raw_alpha std.alpha G6(smc) average_r S/N ase mean sd median_r
## 0.95 0.95 0.96 0.48 18 0.0056 -0.24 1.4 0.48
##
## lower alpha upper 95% confidence boundaries
## 0.94 0.95 0.96
##
## Reliability if an item is dropped:
## raw_alpha std.alpha G6(smc) average_r S/N
## Dataset_Missing_Removed.sers_2 0.94 0.94 0.96 0.47 17
## Dataset_Missing_Removed.sers_4 0.94 0.94 0.96 0.47 17
## Dataset_Missing_Removed.sers_5 0.94 0.94 0.96 0.47 17
## Dataset_Missing_Removed.sers_6 0.94 0.94 0.96 0.47 17
## Dataset_Missing_Removed.sers_7 0.94 0.94 0.96 0.47 17
## Dataset_Missing_Removed.sers_8 0.94 0.95 0.96 0.48 17
## Dataset_Missing_Removed.sers_11 0.94 0.94 0.96 0.47 17
## Dataset_Missing_Removed.sers_12 0.95 0.95 0.96 0.49 18
## Dataset_Missing_Removed.sers_14 0.95 0.95 0.96 0.48 18
## Dataset_Missing_Removed.sers_19 0.95 0.95 0.96 0.48 17
## Dataset_Missing_Removed.sers_1 0.94 0.95 0.96 0.48 17
## Dataset_Missing_Removed.sers_3 0.94 0.94 0.96 0.47 17
## Dataset_Missing_Removed.sers_9 0.94 0.95 0.96 0.48 17
## Dataset_Missing_Removed.sers_10 0.94 0.95 0.96 0.47 17
## Dataset_Missing_Removed.sers_13 0.94 0.95 0.96 0.48 17
## Dataset_Missing_Removed.sers_15 0.94 0.95 0.96 0.47 17
## Dataset_Missing_Removed.sers_16 0.94 0.94 0.96 0.47 17
## Dataset_Missing_Removed.sers_17 0.94 0.95 0.96 0.48 17
## Dataset_Missing_Removed.sers_18 0.95 0.95 0.96 0.48 18
## Dataset_Missing_Removed.sers_20 0.94 0.94 0.96 0.47 17
## alpha se var.r med.r
## Dataset_Missing_Removed.sers_2 0.0059 0.018 0.48
## Dataset_Missing_Removed.sers_4 0.0059 0.017 0.48
## Dataset_Missing_Removed.sers_5 0.0059 0.018 0.48
## Dataset_Missing_Removed.sers_6 0.0058 0.017 0.47
## Dataset_Missing_Removed.sers_7 0.0059 0.017 0.47
## Dataset_Missing_Removed.sers_8 0.0058 0.018 0.48
## Dataset_Missing_Removed.sers_11 0.0058 0.017 0.48
## Dataset_Missing_Removed.sers_12 0.0056 0.015 0.49
## Dataset_Missing_Removed.sers_14 0.0056 0.015 0.48
## Dataset_Missing_Removed.sers_19 0.0058 0.016 0.48
## Dataset_Missing_Removed.sers_1 0.0059 0.017 0.48
## Dataset_Missing_Removed.sers_3 0.0060 0.017 0.47
## Dataset_Missing_Removed.sers_9 0.0058 0.016 0.48
## Dataset_Missing_Removed.sers_10 0.0059 0.017 0.48
## Dataset_Missing_Removed.sers_13 0.0058 0.016 0.48
## Dataset_Missing_Removed.sers_15 0.0059 0.016 0.48
## Dataset_Missing_Removed.sers_16 0.0060 0.017 0.47
## Dataset_Missing_Removed.sers_17 0.0058 0.016 0.48
## Dataset_Missing_Removed.sers_18 0.0057 0.017 0.48
## Dataset_Missing_Removed.sers_20 0.0061 0.016 0.47
##
## Item statistics
## n raw.r std.r r.cor r.drop mean sd
## Dataset_Missing_Removed.sers_2 178 0.73 0.74 0.73 0.70 3.8 1.8
## Dataset_Missing_Removed.sers_4 178 0.72 0.74 0.72 0.69 3.7 1.7
## Dataset_Missing_Removed.sers_5 180 0.72 0.73 0.71 0.69 3.8 1.6
## Dataset_Missing_Removed.sers_6 180 0.72 0.73 0.72 0.69 3.7 1.6
## Dataset_Missing_Removed.sers_7 178 0.76 0.78 0.77 0.73 3.7 1.6
## Dataset_Missing_Removed.sers_8 179 0.71 0.72 0.71 0.68 3.1 1.7
## Dataset_Missing_Removed.sers_11 179 0.72 0.73 0.73 0.69 3.7 1.7
## Dataset_Missing_Removed.sers_12 179 0.53 0.55 0.52 0.48 4.8 1.7
## Dataset_Missing_Removed.sers_14 179 0.59 0.61 0.59 0.54 3.9 1.7
## Dataset_Missing_Removed.sers_19 179 0.68 0.70 0.69 0.65 3.9 1.5
## Dataset_Missing_Removed.sers_1 181 0.70 0.71 0.69 0.68 -4.8 1.7
## Dataset_Missing_Removed.sers_3 177 0.77 0.76 0.76 0.74 -4.4 1.7
## Dataset_Missing_Removed.sers_9 179 0.69 0.68 0.66 0.65 -4.3 1.9
## Dataset_Missing_Removed.sers_10 180 0.74 0.72 0.71 0.70 -4.2 2.0
## Dataset_Missing_Removed.sers_13 179 0.71 0.69 0.67 0.66 -4.4 2.0
## Dataset_Missing_Removed.sers_15 180 0.74 0.73 0.71 0.70 -4.3 1.9
## Dataset_Missing_Removed.sers_16 178 0.78 0.77 0.76 0.74 -3.9 1.9
## Dataset_Missing_Removed.sers_17 180 0.70 0.68 0.67 0.66 -4.1 2.0
## Dataset_Missing_Removed.sers_18 178 0.64 0.63 0.60 0.59 -3.8 1.8
## Dataset_Missing_Removed.sers_20 179 0.80 0.78 0.78 0.77 -3.8 2.3

Calculate composite variable. First, create data frame to calculate the number of SERS positive items missing for each participant and identify how many missing items (NAs) each participant has

sers.positive <- data.frame(Dataset_Missing_Removed$sers_2, Dataset_Missing_Removed$sers_4, Dataset_Missing_Removed$sers_5, Dataset_Missing_Removed$sers_6, Dataset_Missing_Removed$sers_7, Dataset_Missing_Removed$sers_8,Dataset_Missing_Removed$sers_11, Dataset_Missing_Removed$sers_12,Dataset_Missing_Removed$sers_14,
 Dataset_Missing_Removed$sers_19)

apply(sers.positive, 1, function(X) sum(is.na(X)))

## [1] 0 0 0 0 0 0 0 0 0 0 0 0 0 0 0 0 0 10 0 0 0 0 0 0 0
## [26] 0 0 0 0 0 0 0 0 0 0 0 0 0 0 0 0 0 0 0 0 0 0 10 0 0
## [51] 0 0 0 0 0 0 0 0 0 0 0 0 0 0 0 10 0 0 0 0 0 0 0 0 0
## [76] 0 0 0 0 0 0 0 0 0 0 0 0 0 0 0 0 0 0 0 0 0 0 0 0 0
## [101] 0 0 0 0 0 0 0 0 0 0 0 0 0 0 0 0 10 0 0 0 10 0 10 0 10
## [126] 0 0 1 0 0 0 0 10 0 0 10 0 0 0 0 0 0 0 1 0 10 0 0 0 0
## [151] 0 0 0 7 0 0 0 0 0 0 0 0 0 0 0 1 0 0 0 0 0 0 0 0 1
## [176] 0 0 0 0 0 0 0 0 0 0 0 0 0 0 0

Create a new column in original data frame that says how many NAs are in each observation. Can use this to see

Dataset_Missing_Removed$sers.pos_missing <- apply(sers.positive, 1, function(X) sum(is.na(X)))

Replace NAs with mean values of other scores

means_sers.pos <- which(is.na(sers.positive), arr.ind = TRUE)
sers.positive[means_sers.pos] <- rowMeans(sers.positive, na.rm = TRUE)[means_sers.pos[,1]]

Calculate total SERS positive score

sers.positive %>%
 rowwise() %>%
 mutate(sers.pos.total= sum(c(Dataset_Missing_Removed.sers_2, Dataset_Missing_Removed.sers_4,Dataset_Missing_Removed.sers_5, Dataset_Missing_Removed.sers_6,Dataset_Missing_Removed.sers_7, Dataset_Missing_Removed.sers_8, Dataset_Missing_Removed.sers_11, Dataset_Missing_Removed.sers_12,Dataset_Missing_Removed.sers_14,
 Dataset_Missing_Removed.sers_19))) -> sers.positive

Round numbers to nearest whole number and add to the main data frame

sers.positive$sers.pos.total <- floor(0.5 + sers.positive$sers.pos.total)

Dataset_Missing_Removed$sers.pos.total <- sers.positive$sers.pos.total

Repeate for the negative subscale of SERS

sers.negative <- data.frame(Dataset_Missing_Removed$sers_1, Dataset_Missing_Removed$sers_3, Dataset_Missing_Removed$sers_9, Dataset_Missing_Removed$sers_10, Dataset_Missing_Removed$sers_13, Dataset_Missing_Removed$sers_15,Dataset_Missing_Removed$sers_16, Dataset_Missing_Removed$sers_17, Dataset_Missing_Removed$sers_18, Dataset_Missing_Removed$sers_20)

apply(sers.negative, 1, function(X) sum(is.na(X)))

## [1] 0 0 0 0 0 0 0 0 0 0 0 0 0 0 0 0 0 10 0 0 0 0 0 0 0
## [26] 0 0 0 0 0 0 0 0 0 0 0 0 0 0 0 0 0 0 0 0 0 0 10 0 0
## [51] 0 0 0 0 0 0 0 0 0 0 0 0 0 0 0 10 0 0 0 0 0 0 0 1 0
## [76] 0 0 0 0 0 0 0 1 0 0 0 0 0 0 0 0 0 0 0 0 0 0 0 0 0
## [101] 0 0 0 0 0 1 0 0 0 0 0 0 0 1 0 0 10 0 0 0 9 0 10 0 10
## [126] 0 0 1 0 0 0 0 10 0 0 10 0 0 0 0 0 0 0 0 0 10 0 0 0 0
## [151] 0 0 0 4 0 0 0 0 0 0 0 0 0 0 0 0 0 0 0 0 0 1 0 0 0
## [176] 0 0 0 0 0 0 0 0 0 0 0 0 0 0 0

Dataset_Missing_Removed$sers.neg_missing <- apply(sers.negative, 1, function(X) sum(is.na(X)))

means_sers.neg <- which(is.na(sers.negative), arr.ind = TRUE)
sers.negative[means_sers.neg] <- rowMeans(sers.negative, na.rm = TRUE)[means_sers.neg[,1]]

sers.negative %>%
 rowwise() %>%
 mutate(sers.neg.total= sum(c(Dataset_Missing_Removed.sers_1, Dataset_Missing_Removed.sers_3, Dataset_Missing_Removed.sers_9, Dataset_Missing_Removed.sers_10, Dataset_Missing_Removed.sers_13, Dataset_Missing_Removed.sers_15, Dataset_Missing_Removed.sers_16, Dataset_Missing_Removed.sers_17,Dataset_Missing_Removed.sers_18,
 Dataset_Missing_Removed.sers_20))) -> sers.negative

sers.negative$sers.neg.total <- floor(0.5 + sers.negative$sers.neg.total)

Tranform negative subscale total to negative number

sers.negative %>%
 mutate(sers.neg.total = c(sers.neg.total)* -1) -> sers.negative

Add into main data frame

Dataset_Missing_Removed$sers.neg.total <- sers.negative$sers.neg.total

Calculate total number of items missing. Can see that all total scores have been calculated from at least nine items, those with 19 or 20 missing show as NaN

Dataset_Missing_Removed %>%
 rowwise () %>%
 mutate (total.sers.missing = sum(c(sers.neg_missing, sers.pos_missing))) -> Dataset_Missing_Removed

Calculate total SERS score by adding positive and negative subscales together

Dataset_Missing_Removed %>%
 rowwise () %>%
 mutate (SERS_total = sum(c(sers.pos.total, sers.neg.total))) -> Dataset_Missing_Removed

**Attachment**

Label relationships questionnaire

Dataset_Missing_Removed$attachment_style <- as.factor(Dataset_Missing_Removed$attachment_style)

Dataset_Missing_Removed %>%
 mutate(Attachment_style = case_when(attachment_style == 1 ~ "secure",
 attachment_style == 2 ~ "fearful",
 attachment_style == 3 ~ "preoccupied",
 attachment_style == 4 ~ "dismissing")) -> Dataset_Missing_Removed

table(Dataset_Missing_Removed$Attachment_style)

##
## dismissing fearful preoccupied secure
## 24 73 27 28

Rename attachment style rating columns

names(Dataset_Missing_Removed)[names(Dataset_Missing_Removed)== "attach_a"] <- "secure_rating"
names(Dataset_Missing_Removed)[names(Dataset_Missing_Removed)== "attach_b"] <- "fearful_rating"
names(Dataset_Missing_Removed)[names(Dataset_Missing_Removed)== "attach_c"] <- "preocc_rating"
names(Dataset_Missing_Removed)[names(Dataset_Missing_Removed)== "attach_d"] <- "dismiss_rating"

Save progress on data set

write.csv(Dataset_Missing_Removed, file = "Z:\\Online study IRAS ID 271957\\Online analysis\\Dataset_190_obs_2.9.21.csv")
