# Supplementary material for: Being more satisfied with romantic relationship status is associated with increased mental wellbeing in people with experience of psychosis
Source: Front Psychiatry. 2023 Sep 28;14:1232973. doi: 10.3389/fpsyt.2023.1232973 (PMC10569177; doi:10.3389/fpsyt.2023.1232973)
Supplement: Supplementary file 7 [file Data_Sheet_7.DOCX]

Mediation analysis (Self esteem)

Rebecca White

10/08/2022

Install packages and dataset

library(readr)
library(mediation)

## Loading required package: MASS

## Loading required package: Matrix

## Loading required package: mvtnorm

## Loading required package: sandwich

## mediation: Causal Mediation Analysis
## Version: 4.5.0

library(tidyverse)

## -- Attaching packages --------------------------------------- tidyverse 1.3.0 --

## v ggplot2 3.3.2 v dplyr 1.0.2
## v tibble 3.0.4 v stringr 1.4.0
## v tidyr 1.1.2 v forcats 0.5.0
## v purrr 0.3.4

## -- Conflicts ------------------------------------------ tidyverse_conflicts() --
## x tidyr::expand() masks Matrix::expand()
## x dplyr::filter() masks stats::filter()
## x dplyr::lag() masks stats::lag()
## x tidyr::pack() masks Matrix::pack()
## x dplyr::select() masks MASS::select()
## x tidyr::unpack() masks Matrix::unpack()

df <- read_csv("Z:/Online study IRAS ID 271957/Online analysis/Dataset_190_obs_2.9.21.csv")

## Warning: Missing column names filled in: 'X1' [1]

##
## -- Column specification --------------------------------------------------------
## cols(
## .default = col_double(),
## redcap_survey_identifier = col_logical(),
## pis_timestamp = col_datetime(format = ""),
## screening_questions_timestamp = col_datetime(format = ""),
## demographic_information_timestamp = col_datetime(format = ""),
## nationality = col_character(),
## ethnicity_other = col_character(),
## gender_self_describe = col_character(),
## sexual_orientation_selfdescribe = col_character(),
## rr_selfdescribe = col_character(),
## last_rr_end = col_character(),
## current_rr_length = col_character(),
## the_community_assessment_of_psychic_experiences_ca_timestamp = col_datetime(format = ""),
## the_short_warwick_mental_health_wellbeing_scale_timestamp = col_datetime(format = ""),
## adapted_satisfaction_with_relationships_scale_rest_timestamp = col_datetime(format = ""),
## three_item_loneliness_scale_timestamp = col_datetime(format = ""),
## internalised_stigma_of_mental_illness_inventory_10_timestamp = col_datetime(format = ""),
## multidimensional_scale_of_perceived_social_support_timestamp = col_datetime(format = ""),
## self_esteem_rating_scale_short_form_serssf_timestamp = col_datetime(format = ""),
## relationships_questionnaire_timestamp = col_datetime(format = ""),
## Screening_Qs_result = col_character()
## # ... with 7 more columns
## )
## i Use `spec()` for the full column specifications.

## SWEMWBS

testing mediation model : resta -> self esteem -> SWEMWBS

Create dataframe to work from

new.df <- data.frame(df$X1, df$Resta.total, df$SERS_total, df$SWEMWBS_metric)

#remove any rows with NAs
new.df %>%
 filter(! is.na(df.SERS_total) & ! is.na(df.SWEMWBS_metric)) -> new.df

Create model without covariates first

model.m <- lm(df.SERS_total ~ df.Resta.total, data = new.df)
model.y <- lm(df.SWEMWBS_metric ~ df.Resta.total + df.SERS_total, data = new.df)

Check parametric assumptions and for outliers

new.df$standardized.residuals <- rstandard(model.y)
plot(model.y)


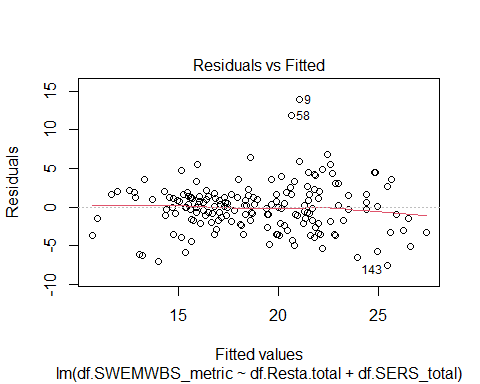

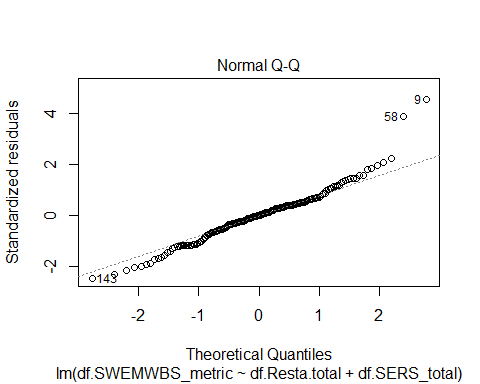

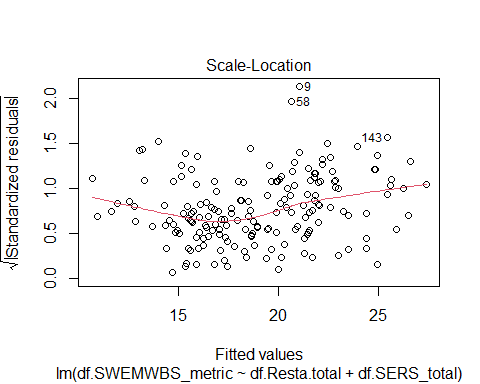

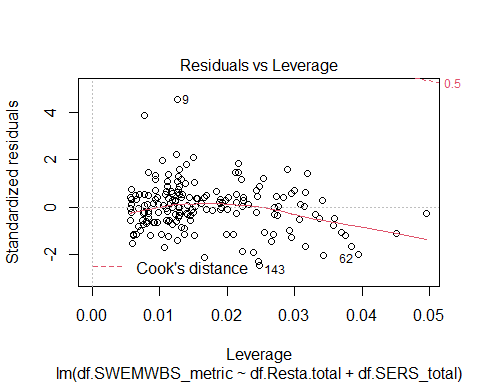


hist(new.df$standardized.residuals)


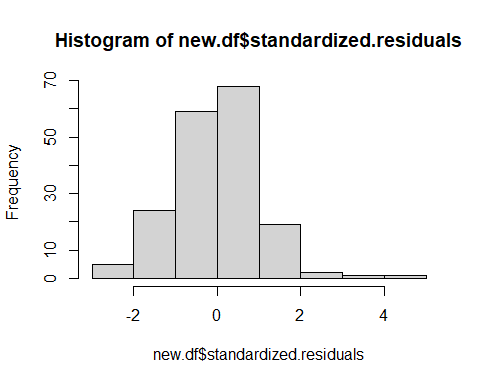


shapiro.test(new.df$standardized.residuals)

##
## Shapiro-Wilk normality test
##
## data: new.df$standardized.residuals
## W = 0.96062, p-value = 6.379e-05

new.df$standardized.residuals > 2 | new.df$standardized.residuals < -2

## [1] FALSE FALSE FALSE FALSE FALSE FALSE FALSE FALSE TRUE FALSE FALSE FALSE
## [13] FALSE FALSE FALSE FALSE FALSE FALSE FALSE FALSE FALSE FALSE FALSE FALSE
## [25] FALSE FALSE FALSE FALSE FALSE FALSE FALSE FALSE FALSE FALSE FALSE FALSE
## [37] FALSE FALSE FALSE FALSE FALSE FALSE FALSE FALSE FALSE FALSE FALSE FALSE
## [49] FALSE FALSE FALSE FALSE FALSE FALSE FALSE FALSE FALSE TRUE FALSE FALSE
## [61] FALSE TRUE FALSE FALSE FALSE FALSE FALSE FALSE FALSE FALSE FALSE FALSE
## [73] FALSE FALSE FALSE FALSE FALSE FALSE FALSE FALSE FALSE FALSE FALSE FALSE
## [85] FALSE FALSE FALSE FALSE FALSE FALSE FALSE FALSE FALSE FALSE FALSE FALSE
## [97] FALSE FALSE FALSE FALSE FALSE FALSE FALSE FALSE FALSE FALSE FALSE FALSE
## [109] FALSE FALSE FALSE FALSE FALSE FALSE FALSE FALSE FALSE FALSE FALSE TRUE
## [121] FALSE FALSE TRUE FALSE FALSE FALSE FALSE FALSE FALSE FALSE TRUE TRUE
## [133] FALSE FALSE FALSE FALSE FALSE FALSE FALSE FALSE FALSE FALSE TRUE FALSE
## [145] FALSE FALSE FALSE FALSE FALSE FALSE FALSE FALSE FALSE FALSE FALSE FALSE
## [157] FALSE FALSE FALSE FALSE FALSE FALSE FALSE FALSE FALSE FALSE FALSE FALSE
## [169] FALSE FALSE FALSE FALSE FALSE FALSE FALSE FALSE FALSE TRUE FALSE

new.df$large.residual <- new.df$standardized.residuals > 2 | new.df$standardized.residuals < -2
sum(new.df$large.residual)

## [1] 9

new.df[new.df$large.residual, c( "df.X1", "standardized.residuals")]

## df.X1 standardized.residuals
## 9 9 4.546497
## 58 60 3.873817
## 62 64 -2.006879
## 120 128 2.092475
## 123 131 -2.046992
## 131 141 -2.140600
## 132 142 2.232729
## 143 154 -2.461852
## 178 189 -2.308985

new.df$cooks.distance <- cooks.distance(model.y)
new.df$leverage <- hatvalues(model.y)
new.df$covariance <- covratio(model.y)

new.df[new.df$large.residual, c("cooks.distance", "leverage", "covariance" )]

## cooks.distance leverage covariance
## 9 0.08833862 0.012658594 0.7082380
## 58 0.03939692 0.007814445 0.7847265
## 62 0.05517479 0.039475544 0.9879932
## 120 0.02219157 0.014977345 0.9575359
## 123 0.04953096 0.034247718 0.9798602
## 131 0.02586751 0.016653722 0.9557567
## 132 0.02105759 0.012513799 0.9450545
## 143 0.05151301 0.024864470 0.9390792
## 178 0.04483234 0.024606531 0.9509700

Run analysis with outliers then remove outliers and re-run analysis

med.with <- mediate(model.m, model.y, sims = 1000, boot = TRUE, treat = "df.Resta.total", mediator = "df.SERS_total")

## Running nonparametric bootstrap

summary(med.with)

##
## Causal Mediation Analysis
##
## Nonparametric Bootstrap Confidence Intervals with the Percentile Method
##
## Estimate 95% CI Lower 95% CI Upper p-value
## ACME 0.1775 0.0740 0.28 <2e-16 ***
## ADE 0.1560 0.0587 0.26 <2e-16 ***
## Total Effect 0.3335 0.2007 0.47 <2e-16 ***
## Prop. Mediated 0.5323 0.3021 0.78 <2e-16 ***
## ---
## Signif. codes: 0 '***' 0.001 '**' 0.01 '*' 0.05 '.' 0.1 ' ' 1
##
## Sample Size Used: 179
##
##
## Simulations: 1000

new.df.OR <- new.df

new.df.OR <- new.df.OR[-c(9, 58),]

model.m.OR <- lm(df.SERS_total ~ df.Resta.total, data = new.df.OR)
model.y.OR <- lm(df.SWEMWBS_metric ~ df.Resta.total + df.SERS_total, data = new.df.OR )

Re-check parametric assumptions

new.df.OR$standardized.residuals <- rstandard(model.y.OR)
plot(model.y.OR)


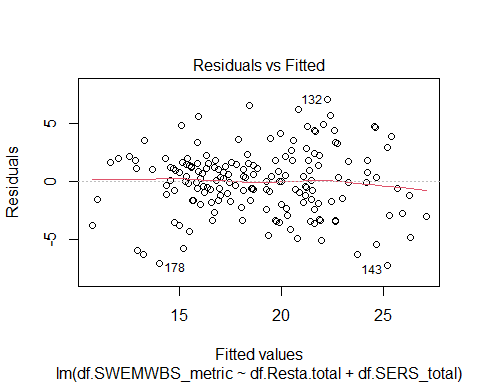

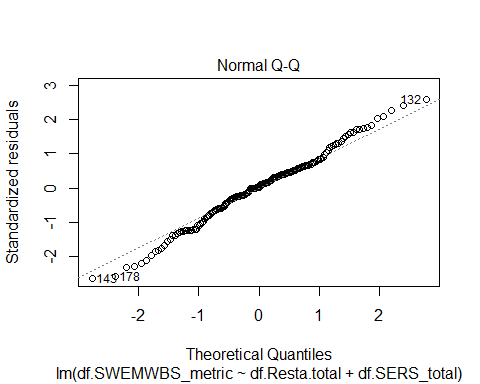

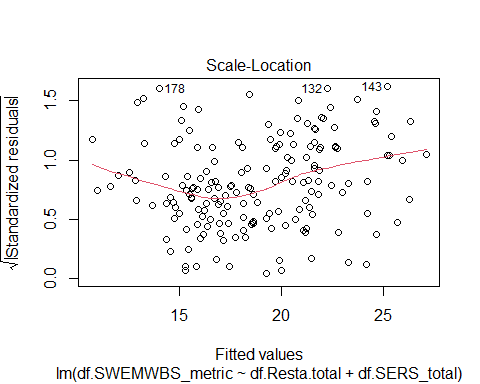

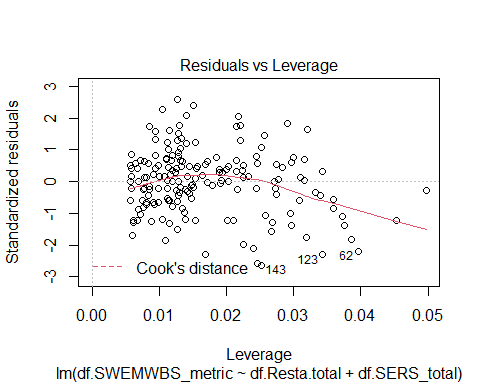


hist(new.df.OR$standardized.residuals)


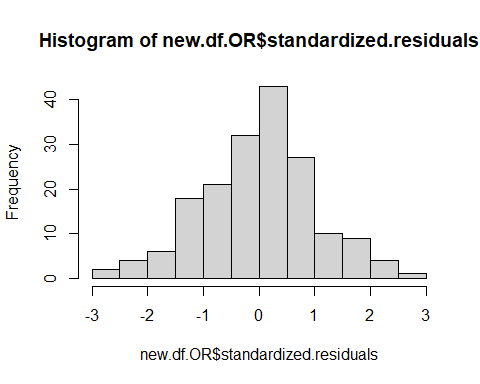


shapiro.test(new.df.OR$standardized.residuals)

##
## Shapiro-Wilk normality test
##
## data: new.df.OR$standardized.residuals
## W = 0.99061, p-value = 0.2986

Re-run analysis

med.without<- mediate(model.m.OR , model.y.OR, sims = 1000, treat = "df.Resta.total",
 mediator = "df.SERS_total")
summary(med.without)

##
## Causal Mediation Analysis
##
## Quasi-Bayesian Confidence Intervals
##
## Estimate 95% CI Lower 95% CI Upper p-value
## ACME 0.1724 0.0770 0.28 0.002 **
## ADE 0.1421 0.0562 0.23 0.004 **
## Total Effect 0.3145 0.1788 0.45 <2e-16 ***
## Prop. Mediated 0.5465 0.3198 0.78 0.002 **
## ---
## Signif. codes: 0 '***' 0.001 '**' 0.01 '*' 0.05 '.' 0.1 ' ' 1
##
## Sample Size Used: 177
##
##
## Simulations: 1000

Add in covariates : gender, age, ethnicity, sexuality, relationship status, employment

Create dataframe to work from using file previously saved

new.df.cov <- read_csv("Z:/Online study IRAS ID 271957/Online analysis/new.df.cov_2022.csv")

## Warning: Missing column names filled in: 'X1' [1]

##
## -- Column specification --------------------------------------------------------
## cols(
## X1 = col_double(),
## Resta = col_double(),
## Loneliness = col_double(),
## gender = col_character(),
## age = col_double(),
## ethnicity = col_character(),
## sexuality = col_character(),
## relationship.status = col_character(),
## SWEMWBS = col_double(),
## employment = col_character(),
## ethnicity.dicotomised = col_character(),
## sexuality.dicotomised = col_character(),
## relationship.dicotomised = col_character(),
## employment.dicotomised = col_character()
## )

#remove column loneliness
new.df.cov <- new.df.cov[, -c(3)]

# add in self esteem (SERS-SF)
new.df.cov$SERS <- df$SERS_total

#remove rows with missing data to allow for mediation package to work
new.df.cov <- na.omit(new.df.cov)

Build model with covariates

model.m.cov <- lm(SERS ~ Resta + gender + age + ethnicity.dicotomised +
 sexuality.dicotomised + relationship.dicotomised +
 employment.dicotomised, data = new.df.cov)
model.y.cov <- lm(SWEMWBS ~ Resta + SERS + gender + age + ethnicity.dicotomised + sexuality.dicotomised + relationship.dicotomised + employment.dicotomised, data = new.df.cov)

Check assumptions

new.df.cov$standardized.residuals <- rstandard(model.y.cov)
plot(model.y.cov)


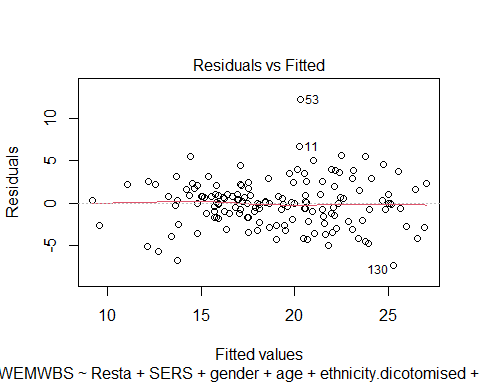

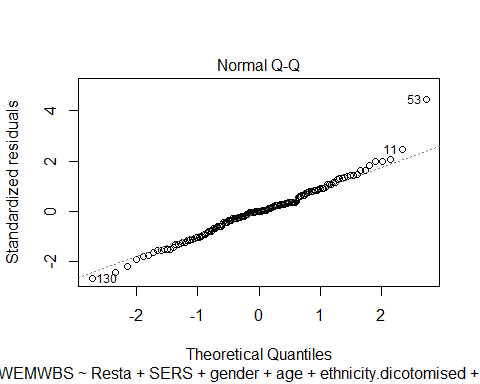

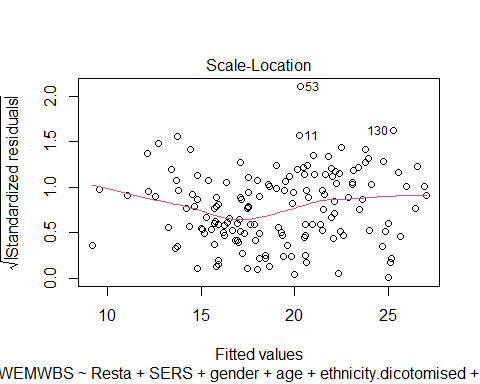

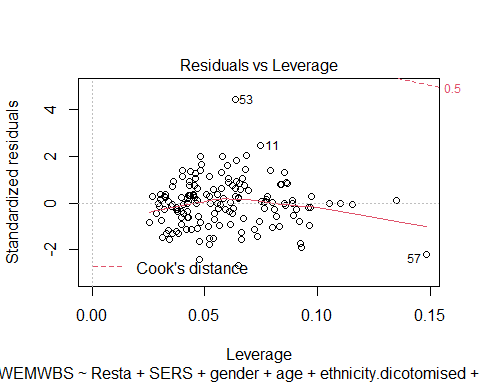


hist(new.df.cov$standardized.residuals)


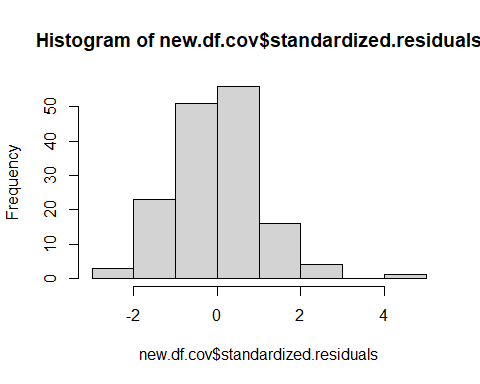


shapiro.test(new.df.cov$standardized.residuals)

##
## Shapiro-Wilk normality test
##
## data: new.df.cov$standardized.residuals
## W = 0.97678, p-value = 0.01051

Check for outliers

new.df.cov$standardized.residuals > 2 | new.df.cov$standardized.residuals < -2

## 1 2 3 4 5 6 7 8 9 10 11 12 13
## FALSE FALSE FALSE FALSE FALSE FALSE FALSE FALSE FALSE FALSE TRUE FALSE FALSE
## 14 15 16 17 18 19 20 21 22 23 24 25 26
## FALSE FALSE FALSE FALSE FALSE FALSE FALSE FALSE FALSE FALSE FALSE FALSE FALSE
## 27 28 29 30 31 32 33 34 35 36 37 38 39
## FALSE FALSE FALSE FALSE FALSE FALSE FALSE FALSE FALSE FALSE FALSE FALSE FALSE
## 40 41 42 43 44 45 46 47 48 49 50 51 52
## TRUE FALSE FALSE FALSE FALSE FALSE FALSE FALSE FALSE FALSE FALSE FALSE FALSE
## 53 54 55 56 57 58 59 60 61 62 63 64 65
## TRUE FALSE FALSE FALSE TRUE FALSE FALSE FALSE FALSE FALSE FALSE FALSE FALSE
## 66 67 68 69 70 71 72 73 74 75 76 77 78
## FALSE FALSE FALSE FALSE FALSE FALSE FALSE FALSE FALSE FALSE FALSE FALSE FALSE
## 79 80 81 82 83 84 85 86 87 88 89 90 91
## FALSE FALSE FALSE FALSE FALSE FALSE FALSE FALSE FALSE FALSE FALSE FALSE FALSE
## 92 93 94 95 96 97 98 99 100 101 102 103 104
## FALSE FALSE FALSE FALSE FALSE FALSE FALSE FALSE FALSE FALSE FALSE FALSE FALSE
## 105 106 107 108 109 110 111 112 113 114 115 116 117
## FALSE FALSE FALSE FALSE FALSE FALSE FALSE FALSE FALSE FALSE FALSE FALSE FALSE
## 118 119 120 121 122 123 124 125 126 127 128 129 130
## FALSE TRUE FALSE FALSE FALSE FALSE FALSE FALSE FALSE FALSE FALSE FALSE TRUE
## 131 132 133 134 135 136 137 138 139 140 141 142 143
## FALSE FALSE FALSE FALSE FALSE FALSE FALSE FALSE FALSE FALSE FALSE FALSE FALSE
## 144 145 146 147 148 149 150 151 152 153 154
## FALSE FALSE FALSE FALSE FALSE FALSE FALSE FALSE TRUE FALSE TRUE

new.df.cov$large.residual <- new.df.cov$standardized.residuals > 2 | new.df.cov$standardized.residuals < -2
sum(new.df.cov$large.residual)

## [1] 8

new.df.cov[new.df.cov$large.residual, c("X1", "standardized.residuals")]

## # A tibble: 8 x 2
## X1 standardized.residuals
## <dbl> <dbl>
## 1 13 2.46
## 2 44 2.06
## 3 60 4.44
## 4 64 -2.18
## 5 142 2.01
## 6 154 -2.65
## 7 181 2.01
## 8 189 -2.42

new.df.cov$cooks.distance <- cooks.distance(model.y.cov)
new.df.cov$leverage <- hatvalues(model.y.cov)
new.df.cov$covariance <- covratio(model.y.cov)

new.df.cov[new.df.cov$large.residual, c("X1", "cooks.distance", "leverage", "covariance" )]

## # A tibble: 8 x 4
## X1 cooks.distance leverage covariance
## <dbl> <dbl> <dbl> <dbl>
## 1 13 0.0545 0.0748 0.783
## 2 44 0.0344 0.0683 0.875
## 3 60 0.148 0.0634 0.304
## 4 64 0.0920 0.148 0.925
## 5 142 0.0276 0.0580 0.877
## 6 154 0.0542 0.0648 0.727
## 7 181 0.0227 0.0483 0.868
## 8 189 0.0323 0.0475 0.772

Remove outlier

new.df.cov %>%
 filter (! (X1 == 60)) -> new.df.cov

Build models again

model.m.cov <- lm(SERS ~ Resta + gender + age + ethnicity.dicotomised +
 sexuality.dicotomised + relationship.dicotomised +
 employment.dicotomised, data = new.df.cov)
model.y.cov <- lm(SWEMWBS ~ Resta + SERS + gender + age + ethnicity.dicotomised + sexuality.dicotomised + relationship.dicotomised + employment.dicotomised, data = new.df.cov)

Re-check assumptions

new.df.cov$standardized.residuals <- rstandard(model.y.cov)
plot(model.y.cov)


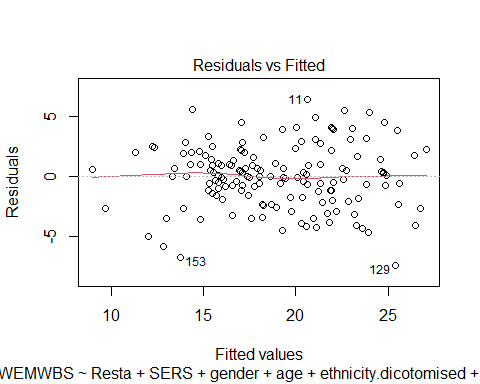

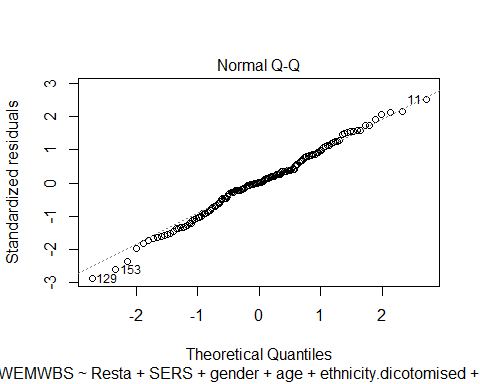

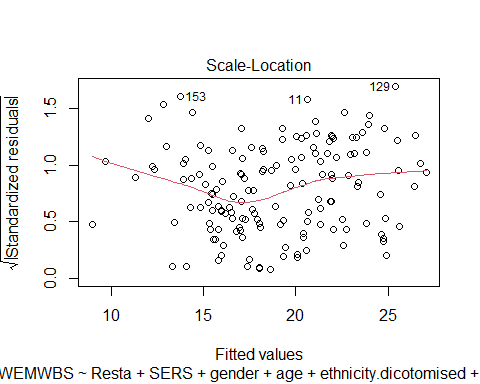

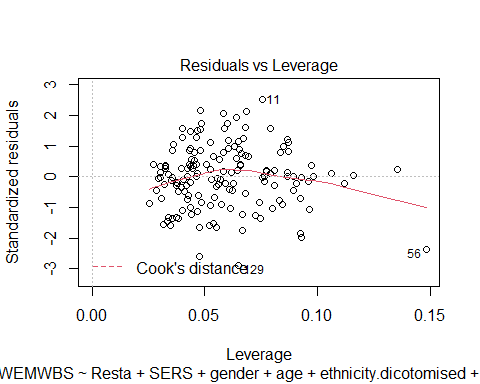


hist(new.df.cov$standardized.residuals)


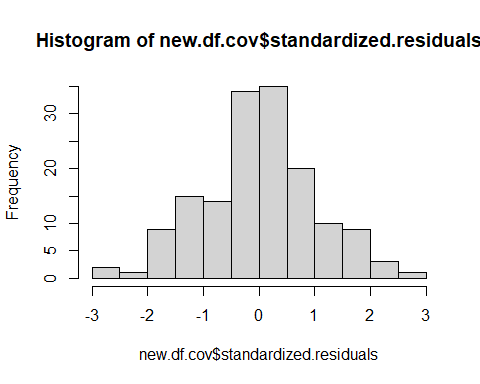


shapiro.test(new.df.cov$standardized.residuals)

##
## Shapiro-Wilk normality test
##
## data: new.df.cov$standardized.residuals
## W = 0.99308, p-value = 0.6746

Run analysis

med.cov<- mediate(model.m.cov , model.y.cov, sims = 5000,
 treat = "Resta", mediator = "SERS")
summary(med.cov)

##
## Causal Mediation Analysis
##
## Quasi-Bayesian Confidence Intervals
##
## Estimate 95% CI Lower 95% CI Upper p-value
## ACME 0.3298 0.2090 0.46 <2e-16 ***
## ADE 0.1852 0.0638 0.30 0.0016 **
## Total Effect 0.5150 0.3535 0.67 <2e-16 ***
## Prop. Mediated 0.6391 0.4618 0.85 <2e-16 ***
## ---
## Signif. codes: 0 '***' 0.001 '**' 0.01 '*' 0.05 '.' 0.1 ' ' 1
##
## Sample Size Used: 153
##
##
## Simulations: 5000

## CAPE POSITIVE

testing mediation model : resta -> self esteem -> CAPE pos

Create data frame to work from

new.df2 <- data.frame(df$Resta.total, df$SERS_total, df$CAPE_positive)

#remove any rows with NAs
new.df2 %>%
 filter(! is.na(df.SERS_total)) -> new.df2

Build models

model.m2 <- lm(df.SERS_total ~ df.Resta.total, data = new.df2)
model.y2 <- lm(df.CAPE_positive ~ df.Resta.total + df.SERS_total, data = new.df2)

Check parametric assumptions and for outliers

new.df2$standardized.residuals <- rstandard(model.y2)
plot(model.y2)


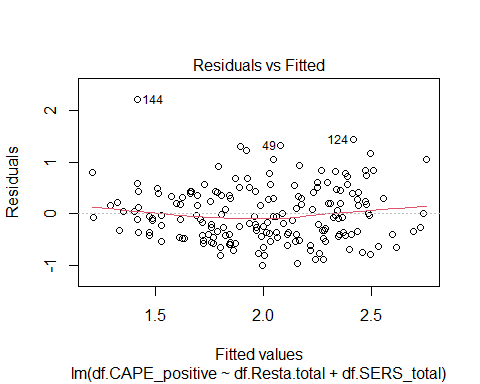

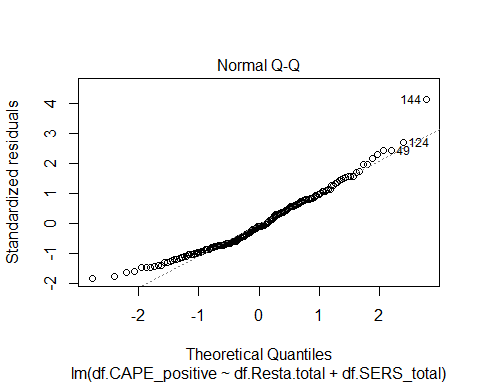

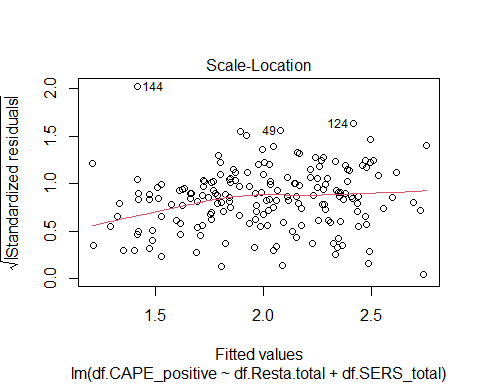

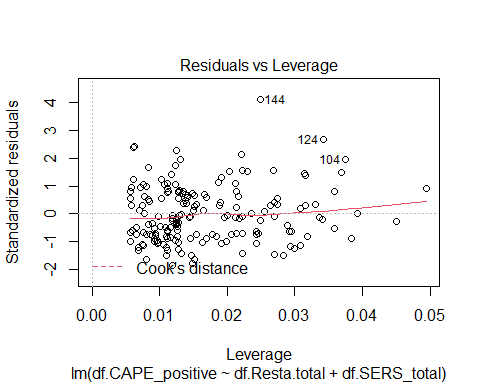


hist(new.df2$standardized.residuals)


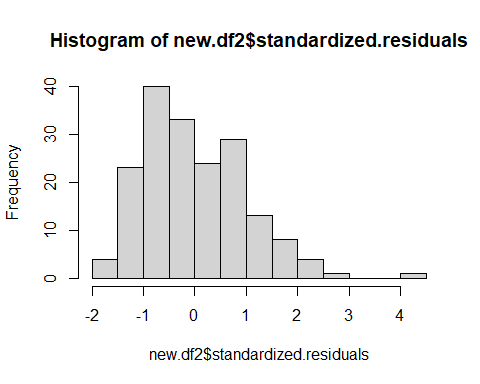


shapiro.test(new.df2$standardized.residuals)

##
## Shapiro-Wilk normality test
##
## data: new.df2$standardized.residuals
## W = 0.96382, p-value = 0.0001316

new.df2$standardized.residuals > 2 | new.df2$standardized.residuals < -2

## [1] FALSE FALSE FALSE FALSE FALSE FALSE FALSE FALSE FALSE FALSE FALSE FALSE
## [13] FALSE FALSE FALSE FALSE FALSE FALSE FALSE FALSE FALSE FALSE FALSE FALSE
## [25] FALSE FALSE FALSE FALSE FALSE FALSE FALSE FALSE FALSE FALSE FALSE FALSE
## [37] FALSE FALSE FALSE FALSE FALSE FALSE FALSE FALSE FALSE FALSE FALSE FALSE
## [49] TRUE FALSE FALSE FALSE FALSE FALSE FALSE FALSE FALSE FALSE FALSE FALSE
## [61] FALSE FALSE FALSE FALSE FALSE FALSE FALSE FALSE FALSE FALSE FALSE FALSE
## [73] FALSE FALSE TRUE FALSE FALSE FALSE FALSE FALSE FALSE FALSE FALSE FALSE
## [85] FALSE FALSE FALSE FALSE FALSE FALSE FALSE FALSE FALSE FALSE FALSE FALSE
## [97] FALSE FALSE FALSE FALSE FALSE TRUE FALSE FALSE FALSE FALSE FALSE FALSE
## [109] FALSE FALSE FALSE FALSE FALSE FALSE FALSE FALSE FALSE FALSE FALSE FALSE
## [121] FALSE FALSE FALSE TRUE FALSE FALSE FALSE FALSE FALSE FALSE FALSE FALSE
## [133] FALSE FALSE FALSE FALSE FALSE FALSE FALSE FALSE FALSE FALSE FALSE TRUE
## [145] FALSE FALSE FALSE FALSE TRUE FALSE FALSE FALSE FALSE FALSE FALSE FALSE
## [157] FALSE FALSE FALSE FALSE FALSE FALSE FALSE FALSE FALSE FALSE FALSE FALSE
## [169] FALSE FALSE FALSE FALSE FALSE FALSE FALSE FALSE FALSE FALSE FALSE FALSE

new.df2$large.residual <- new.df2$standardized.residuals > 2 | new.df2$standardized.residuals < -2
sum(new.df2$large.residual)

## [1] 6

new.df2[new.df2$large.residual, c( "standardized.residuals")]

## [1] 2.432291 2.276189 2.406834 2.680437 4.110127 2.151187

new.df2$cooks.distance <- cooks.distance(model.y2)
new.df2$leverage <- hatvalues(model.y2)
new.df2$covariance <- covratio(model.y2)

new.df2[new.df2$large.residual, c("cooks.distance", "leverage", "covariance" )]

## cooks.distance leverage covariance
## 49 0.01250868 0.006303122 0.9243490
## 75 0.02186005 0.012499509 0.9421888
## 102 0.01186253 0.006105853 0.9261633
## 124 0.08492887 0.034247685 0.9300930
## 144 0.14345252 0.024842415 0.7719995
## 149 0.03483954 0.022087044 0.9606495

Run with outlier first then remove outlier and build models again

med.with2 <- mediate(model.m2 , model.y2, sims = 1000, boot = TRUE,
 treat = "df.Resta.total",
 mediator = "df.SERS_total")

## Running nonparametric bootstrap

summary(med.with2)

##
## Causal Mediation Analysis
##
## Nonparametric Bootstrap Confidence Intervals with the Percentile Method
##
## Estimate 95% CI Lower 95% CI Upper p-value
## ACME -0.01902 -0.03115 -0.01 <2e-16 ***
## ADE 0.00496 -0.01291 0.02 0.54
## Total Effect -0.01406 -0.03467 0.01 0.23
## Prop. Mediated 1.35289 -8.41604 10.63 0.23
## ---
## Signif. codes: 0 '***' 0.001 '**' 0.01 '*' 0.05 '.' 0.1 ' ' 1
##
## Sample Size Used: 180
##
##
## Simulations: 1000

new.df2.OR <- new.df2[-c(144),]

model.m2.OR <- lm(df.SERS_total ~ df.Resta.total, data = new.df2.OR)
model.y2.OR <- lm(df.CAPE_positive ~ df.Resta.total + df.SERS_total, data = new.df2.OR )

Check parametric assumptions

new.df2.OR$standardized.residuals <- rstandard(model.y2.OR)
plot(model.y2.OR)


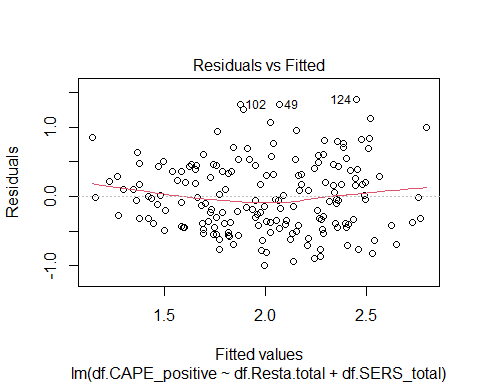

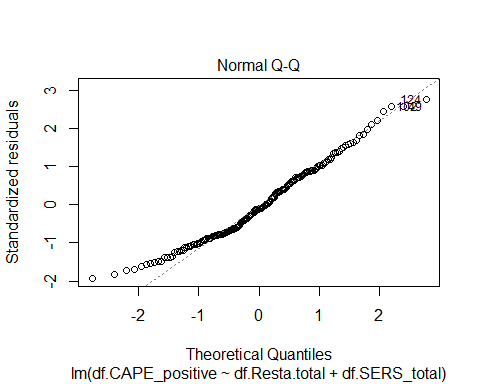

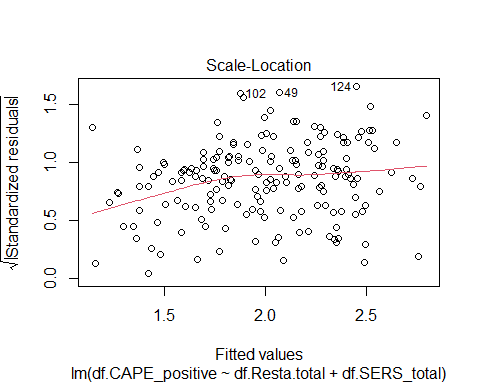

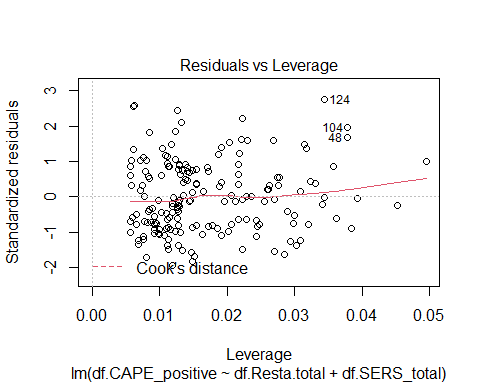


hist(new.df2.OR$standardized.residuals)


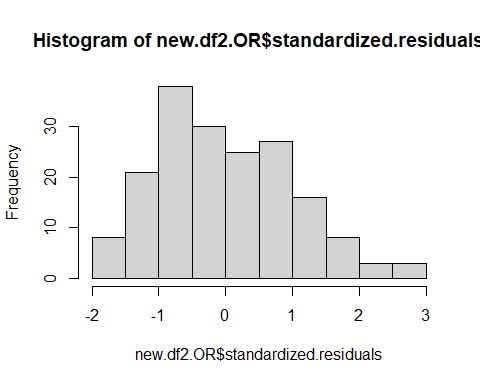


shapiro.test(new.df2.OR$standardized.residuals)

##
## Shapiro-Wilk normality test
##
## data: new.df2.OR$standardized.residuals
## W = 0.97582, p-value = 0.00331

Re run analysis

med.without2<- mediate(model.m2.OR , model.y2.OR, sims = 1000, boot = TRUE,
 treat = "df.Resta.total",
 mediator = "df.SERS_total")

## Running nonparametric bootstrap

summary(med.without2)

##
## Causal Mediation Analysis
##
## Nonparametric Bootstrap Confidence Intervals with the Percentile Method
##
## Estimate 95% CI Lower 95% CI Upper p-value
## ACME -0.01932 -0.03245 -0.01 0.002 **
## ADE 0.00295 -0.01452 0.02 0.714
## Total Effect -0.01637 -0.03730 0.01 0.152
## Prop. Mediated 1.18043 -3.70832 7.07 0.150
## ---
## Signif. codes: 0 '***' 0.001 '**' 0.01 '*' 0.05 '.' 0.1 ' ' 1
##
## Sample Size Used: 179
##
##
## Simulations: 1000

Add in co-variates

#Upload previous dataframe to work from
new.df.cov2 <- read_csv("Z:/Online study IRAS ID 271957/Online analysis/new.df.cov_2022.csv")

## Warning: Missing column names filled in: 'X1' [1]

##
## -- Column specification --------------------------------------------------------
## cols(
## X1 = col_double(),
## Resta = col_double(),
## Loneliness = col_double(),
## gender = col_character(),
## age = col_double(),
## ethnicity = col_character(),
## sexuality = col_character(),
## relationship.status = col_character(),
## SWEMWBS = col_double(),
## employment = col_character(),
## ethnicity.dicotomised = col_character(),
## sexuality.dicotomised = col_character(),
## relationship.dicotomised = col_character(),
## employment.dicotomised = col_character()
## )

#Add CAPE positive subscale scores and self esteem score to the dataframe. Remove loneliness column
new.df.cov2$CAPE_pos <- df$CAPE_positive
new.df.cov2$SERS <- df$SERS_total
new.df.cov2 <- new.df.cov2[, -c(3)]

#remove rows with missing data to allow for mediation package to work
new.df.cov2 <- na.omit(new.df.cov2)

Create model with co-variates

model.m2.cov <- lm(SERS ~ Resta + gender + age + ethnicity.dicotomised +
 sexuality.dicotomised + relationship.dicotomised +
 employment.dicotomised, data = new.df.cov2)
model.y2.cov <- lm(CAPE_pos ~ Resta + SERS + gender + age + ethnicity.dicotomised + sexuality.dicotomised + relationship.dicotomised +
 employment.dicotomised, data = new.df.cov2)

Check parametric assumptions and for outliers

new.df.cov2$standardized.residuals <- rstandard(model.y2.cov)
plot(model.y2.cov)


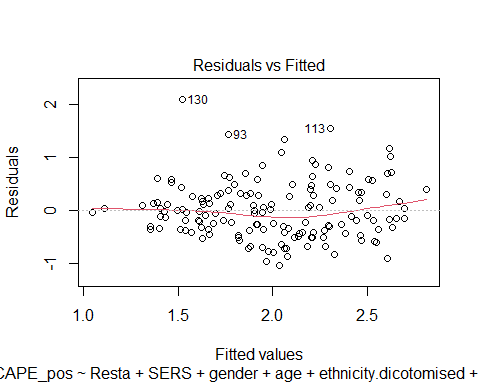

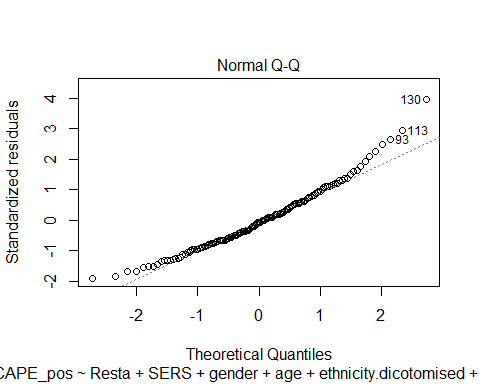

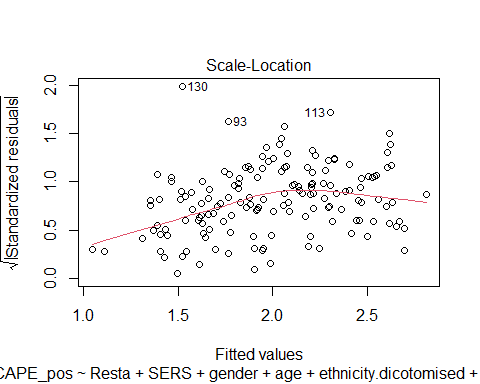

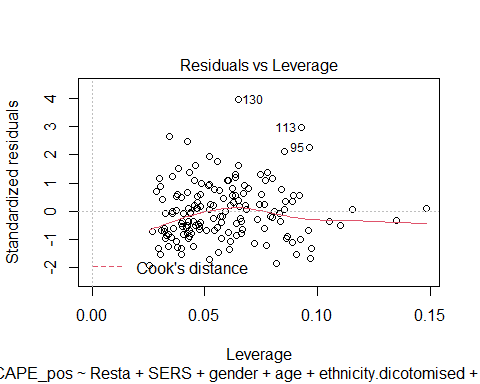


hist(new.df.cov2$standardized.residuals)


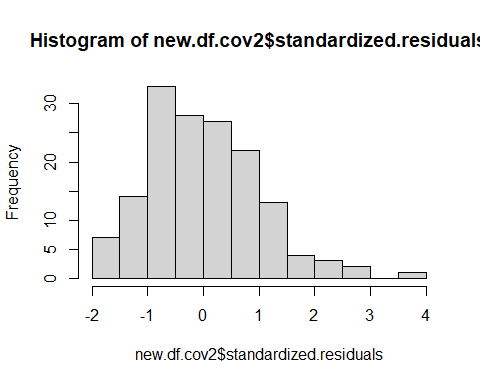


shapiro.test(new.df.cov2$standardized.residuals)

##
## Shapiro-Wilk normality test
##
## data: new.df.cov2$standardized.residuals
## W = 0.96634, p-value = 0.0008145

new.df.cov2$standardized.residuals > 2 |new.df.cov2$standardized.residuals < -2

## 1 2 3 4 5 6 7 8 9 10 11 12 13
## FALSE FALSE FALSE FALSE FALSE FALSE FALSE FALSE FALSE FALSE FALSE FALSE FALSE
## 14 15 16 17 18 19 20 21 22 23 24 25 26
## FALSE FALSE FALSE FALSE FALSE FALSE FALSE FALSE FALSE FALSE FALSE FALSE FALSE
## 27 28 29 30 31 32 33 34 35 36 37 38 39
## FALSE FALSE FALSE FALSE FALSE FALSE FALSE FALSE FALSE FALSE FALSE FALSE FALSE
## 40 41 42 43 44 45 46 47 48 49 50 51 52
## FALSE FALSE FALSE FALSE FALSE TRUE FALSE FALSE FALSE FALSE FALSE FALSE FALSE
## 53 54 55 56 57 58 59 60 61 62 63 64 65
## FALSE FALSE FALSE FALSE FALSE FALSE FALSE FALSE FALSE FALSE FALSE FALSE FALSE
## 66 67 68 69 70 71 72 73 74 75 76 77 78
## FALSE TRUE FALSE FALSE FALSE FALSE FALSE FALSE FALSE FALSE FALSE FALSE FALSE
## 79 80 81 82 83 84 85 86 87 88 89 90 91
## FALSE FALSE FALSE FALSE FALSE FALSE FALSE FALSE FALSE FALSE FALSE FALSE FALSE
## 92 93 94 95 96 97 98 99 100 101 102 103 104
## FALSE TRUE FALSE TRUE FALSE FALSE FALSE FALSE FALSE FALSE FALSE FALSE FALSE
## 105 106 107 108 109 110 111 112 113 114 115 116 117
## FALSE FALSE FALSE FALSE FALSE FALSE FALSE FALSE TRUE FALSE FALSE FALSE FALSE
## 118 119 120 121 122 123 124 125 126 127 128 129 130
## FALSE FALSE FALSE FALSE FALSE FALSE FALSE FALSE FALSE FALSE FALSE FALSE TRUE
## 131 132 133 134 135 136 137 138 139 140 141 142 143
## FALSE FALSE FALSE FALSE FALSE FALSE FALSE FALSE FALSE FALSE FALSE FALSE FALSE
## 144 145 146 147 148 149 150 151 152 153 154
## FALSE FALSE FALSE FALSE FALSE FALSE FALSE FALSE FALSE FALSE FALSE

new.df.cov2$large.residual <- new.df.cov2$standardized.residuals > 2 | new.df.cov2$standardized.residuals < -2
sum(new.df.cov2$large.residual)

## [1] 6

new.df.cov2[new.df.cov2$large.residual, c("X1", "standardized.residuals")]

## # A tibble: 6 x 2
## X1 standardized.residuals
## <dbl> <dbl>
## 1 51 2.48
## 2 78 2.10
## 3 105 2.65
## 4 107 2.26
## 5 131 2.95
## 6 154 3.95

new.df.cov2$cooks.distance <- cooks.distance(model.y2.cov)
new.df.cov2$leverage <- hatvalues(model.y2.cov)
new.df.cov2$covariance <- covratio(model.y2.cov)

new.df.cov2[new.df.cov2$large.residual, c("X1", "cooks.distance", "leverage", "covariance" )]

## # A tibble: 6 x 4
## X1 cooks.distance leverage covariance
## <dbl> <dbl> <dbl> <dbl>
## 1 51 0.0302 0.0422 0.751
## 2 78 0.0455 0.0851 0.881
## 3 105 0.0279 0.0345 0.705
## 4 107 0.0606 0.0963 0.852
## 5 131 0.0992 0.0929 0.672
## 6 154 0.120 0.0648 0.408

Remove outlier

new.df.cov2 %>%
 filter (! (X1 == 154)) -> new.df.cov2

Build models again

model.m2.cov <- lm(SERS ~ Resta + gender + age + ethnicity.dicotomised +
 sexuality.dicotomised + relationship.dicotomised +
 employment.dicotomised, data = new.df.cov2)
model.y2.cov <- lm(CAPE_pos ~ Resta + SERS + gender + age + ethnicity.dicotomised + sexuality.dicotomised + relationship.dicotomised +
 employment.dicotomised, data = new.df.cov2)

Re-check assumptions

new.df.cov2$standardized.residuals <- rstandard(model.y2.cov)
plot(model.y2.cov)


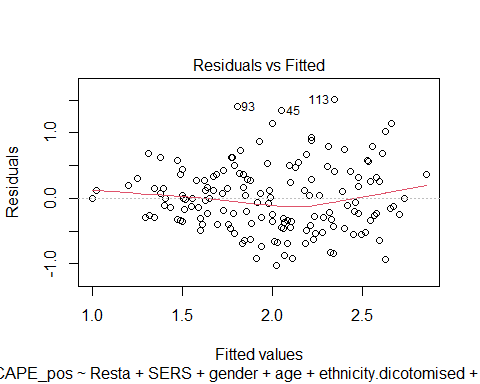

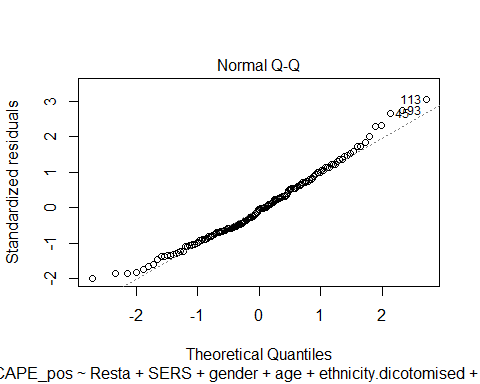

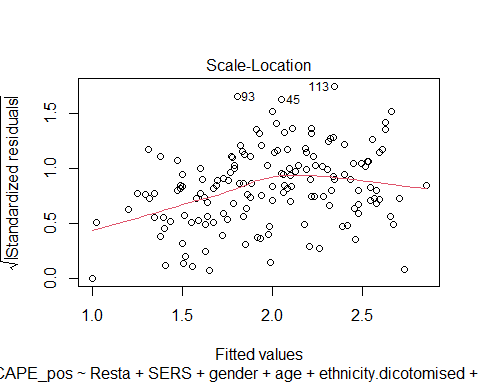

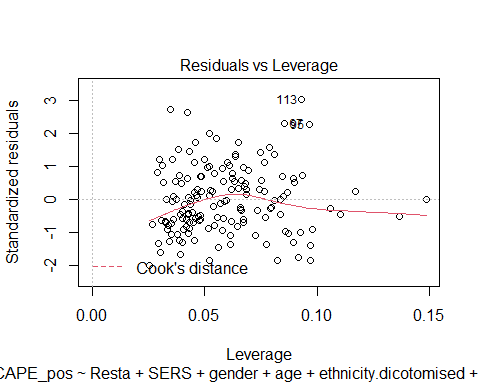


hist(new.df.cov2$standardized.residuals)


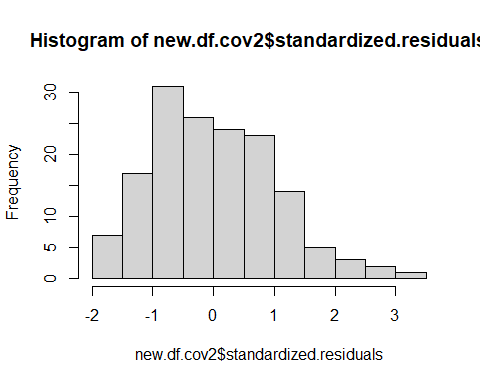


shapiro.test(new.df.cov2$standardized.residuals)

##
## Shapiro-Wilk normality test
##
## data: new.df.cov2$standardized.residuals
## W = 0.98113, p-value = 0.03413

Re-run mediation analysis

med.cov2<- mediate(model.m2.cov , model.y2.cov, sims = 5000, boot = TRUE,
 treat = "Resta", mediator = "SERS")

## Running nonparametric bootstrap

summary(med.cov2)

##
## Causal Mediation Analysis
##
## Nonparametric Bootstrap Confidence Intervals with the Percentile Method
##
## Estimate 95% CI Lower 95% CI Upper p-value
## ACME -0.034328 -0.050180 -0.02 <2e-16 ***
## ADE 0.000984 -0.024197 0.03 0.937
## Total Effect -0.033344 -0.061832 0.00 0.023 *
## Prop. Mediated 1.029496 0.525072 4.05 0.023 *
## ---
## Signif. codes: 0 '***' 0.001 '**' 0.01 '*' 0.05 '.' 0.1 ' ' 1
##
## Sample Size Used: 153
##
##
## Simulations: 5000

## CAPE NEGATIVE

Testing mediation model : Resta -> self esteem -> CAPE negative

Create data frame to work from

new.df3 <- data.frame(df$Resta.total, df$SERS_total, df$CAPE_negative)

#remove any rows with NAs
new.df3 %>%
 filter(! is.na(df.SERS_total)) -> new.df3

Build models

model.m3 <- lm(df.SERS_total ~ df.Resta.total, data = new.df3)
model.y3 <- lm(df.CAPE_negative ~ df.Resta.total + df.SERS_total, data = new.df3)

Check parametric assumptions and for outliers

new.df3$standardized.residuals <- rstandard(model.y3)
plot(model.y3)


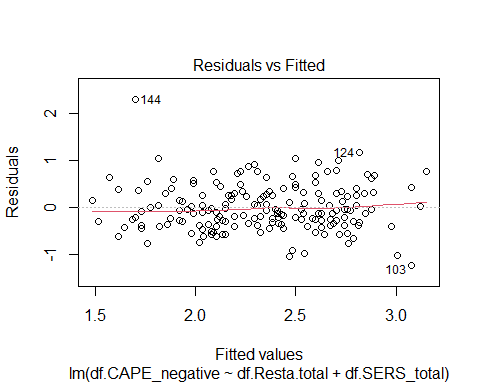

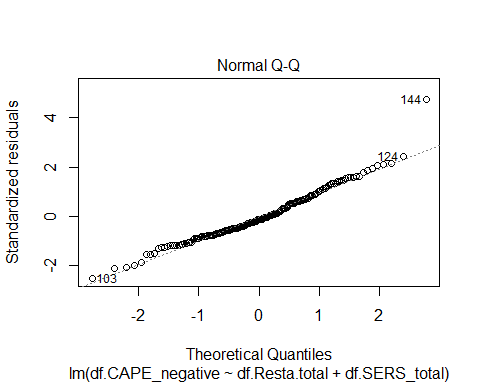

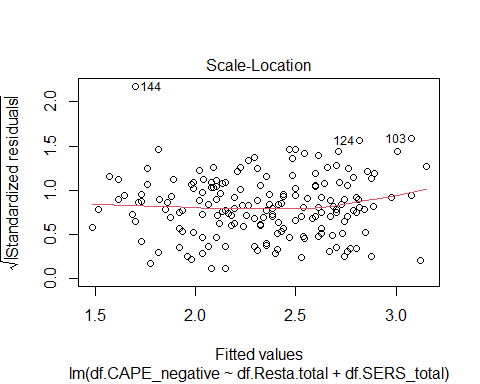

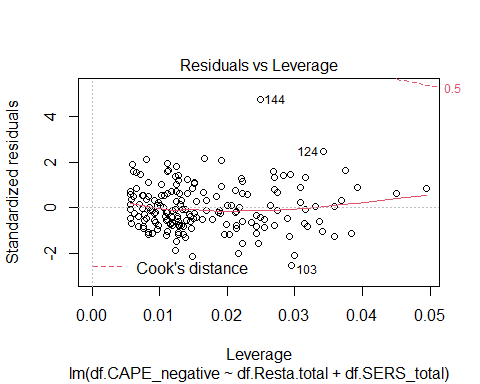


hist(new.df3$standardized.residuals)


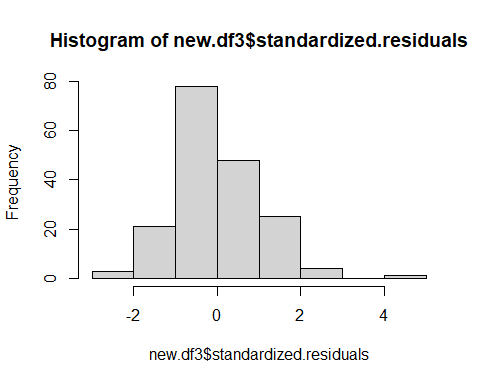


shapiro.test(new.df3$standardized.residuals)

##
## Shapiro-Wilk normality test
##
## data: new.df3$standardized.residuals
## W = 0.96663, p-value = 0.0002662

new.df3$standardized.residuals > 2 | new.df3$standardized.residuals < -2

## [1] FALSE TRUE FALSE FALSE FALSE FALSE FALSE FALSE FALSE FALSE FALSE FALSE
## [13] FALSE FALSE FALSE FALSE FALSE FALSE FALSE FALSE FALSE FALSE FALSE FALSE
## [25] FALSE FALSE FALSE FALSE FALSE FALSE FALSE FALSE FALSE FALSE FALSE FALSE
## [37] FALSE FALSE FALSE FALSE FALSE FALSE FALSE FALSE FALSE FALSE FALSE FALSE
## [49] FALSE FALSE FALSE FALSE FALSE FALSE FALSE FALSE FALSE FALSE FALSE TRUE
## [61] FALSE FALSE FALSE FALSE FALSE FALSE FALSE FALSE FALSE FALSE FALSE FALSE
## [73] FALSE FALSE FALSE FALSE FALSE FALSE FALSE FALSE FALSE FALSE FALSE FALSE
## [85] FALSE FALSE FALSE FALSE FALSE FALSE FALSE FALSE FALSE FALSE FALSE FALSE
## [97] FALSE FALSE FALSE FALSE FALSE FALSE TRUE FALSE FALSE FALSE FALSE FALSE
## [109] FALSE FALSE FALSE FALSE FALSE FALSE FALSE FALSE FALSE FALSE FALSE FALSE
## [121] TRUE FALSE FALSE TRUE FALSE FALSE FALSE FALSE FALSE FALSE FALSE TRUE
## [133] FALSE FALSE TRUE FALSE FALSE FALSE FALSE FALSE FALSE FALSE FALSE TRUE
## [145] FALSE FALSE FALSE FALSE FALSE FALSE FALSE FALSE FALSE FALSE FALSE FALSE
## [157] FALSE FALSE FALSE FALSE FALSE FALSE FALSE FALSE FALSE FALSE FALSE FALSE
## [169] FALSE FALSE FALSE FALSE FALSE FALSE FALSE FALSE FALSE FALSE FALSE FALSE

new.df3$large.residual <- new.df3$standardized.residuals > 2 | new.df3$standardized.residuals < -2
sum(new.df3$large.residual)

## [1] 8

new.df3[new.df3$large.residual, c("standardized.residuals")]

## [1] 2.052741 -2.075833 -2.518365 -2.125535 2.442056 2.133716 2.122281
## [8] 4.734051

new.df3$cooks.distance <- cooks.distance(model.y3)
new.df3$leverage <- hatvalues(model.y3)
new.df3$covariance <- covratio(model.y3)

new.df3[new.df3$large.residual, c("cooks.distance", "leverage", "covariance" )]

## cooks.distance leverage covariance
## 2 0.02745152 0.019169616 0.9647075
## 60 0.04432804 0.029937453 0.9738022
## 103 0.06433755 0.029534421 0.9394217
## 121 0.02267114 0.014830944 0.9553958
## 124 0.07049456 0.034247685 0.9503019
## 132 0.02568945 0.016646104 0.9565794
## 135 0.01228886 0.008118704 0.9491586
## 144 0.19031084 0.024842415 0.6948979

Run analysis with outlier then remove outlier and build models again

med.with3 <- mediate(model.m3 , model.y3, sims = 1000, boot = TRUE,
 treat = "df.Resta.total",
 mediator = "df.SERS_total")

## Running nonparametric bootstrap

summary(med.with3)

##
## Causal Mediation Analysis
##
## Nonparametric Bootstrap Confidence Intervals with the Percentile Method
##
## Estimate 95% CI Lower 95% CI Upper p-value
## ACME -0.019762 -0.031581 -0.01 0.002 **
## ADE -0.000263 -0.017443 0.02 0.946
## Total Effect -0.020024 -0.041134 0.00 0.086 .
## Prop. Mediated 0.986884 -3.179081 4.52 0.084 .
## ---
## Signif. codes: 0 '***' 0.001 '**' 0.01 '*' 0.05 '.' 0.1 ' ' 1
##
## Sample Size Used: 180
##
##
## Simulations: 1000

new.df3.OR <- new.df3[-c(144),]

model.m3.OR <- lm(df.SERS_total ~ df.Resta.total, data = new.df3.OR)
model.y3.OR <- lm(df.CAPE_negative ~ df.Resta.total + df.SERS_total, data = new.df3.OR)

Re-check parametric assumptions

new.df3.OR$standardized.residuals <- rstandard(model.y3.OR)
plot(model.y3.OR)


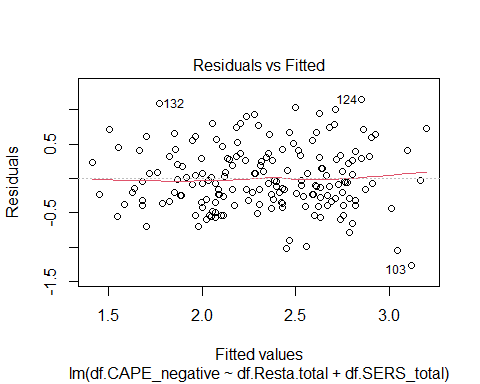

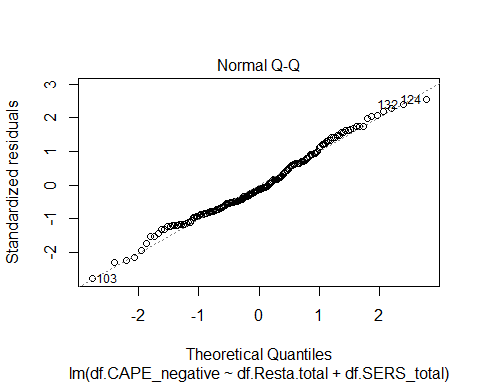

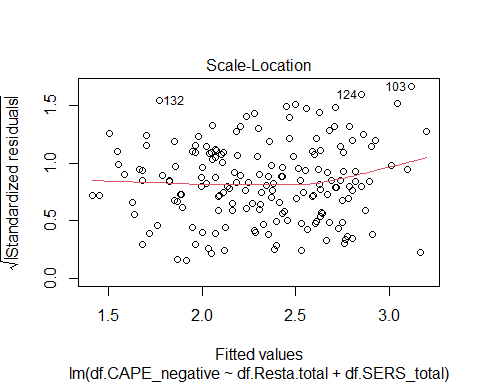

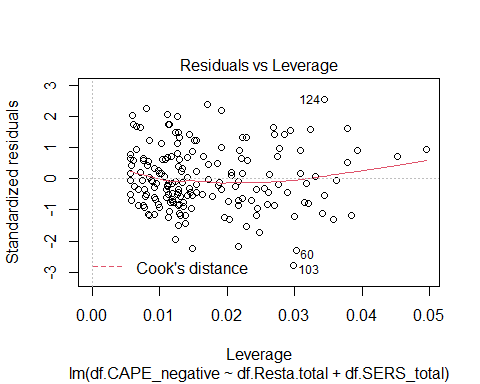


hist(new.df3.OR$standardized.residuals)


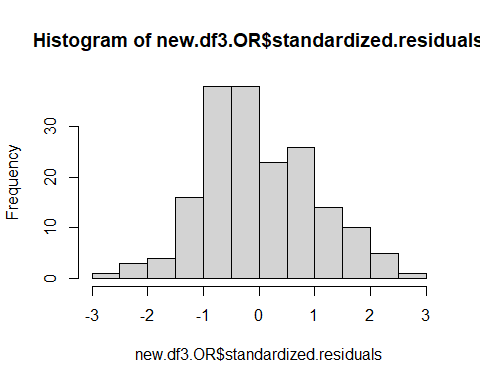


shapiro.test(new.df3.OR$standardized.residuals)

##
## Shapiro-Wilk normality test
##
## data: new.df3.OR$standardized.residuals
## W = 0.98584, p-value = 0.06797

Run mediation analysis

med.without3<- mediate(model.m3.OR , model.y3.OR, sims = 1000,
 treat = "df.Resta.total",
 mediator = "df.SERS_total")
summary(med.without3)

##
## Causal Mediation Analysis
##
## Quasi-Bayesian Confidence Intervals
##
## Estimate 95% CI Lower 95% CI Upper p-value
## ACME -0.0205 -0.0331 -0.01 <2e-16 ***
## ADE -0.0023 -0.0173 0.01 0.762
## Total Effect -0.0228 -0.0415 0.00 0.024 *
## Prop. Mediated 0.8866 0.4480 2.65 0.024 *
## ---
## Signif. codes: 0 '***' 0.001 '**' 0.01 '*' 0.05 '.' 0.1 ' ' 1
##
## Sample Size Used: 179
##
##
## Simulations: 1000

Add in co-variates

#upload previous data frame to work from
new.df.cov3 <- read_csv("Z:/Online study IRAS ID 271957/Online analysis/new.df.cov_2022.csv")

## Warning: Missing column names filled in: 'X1' [1]

##
## -- Column specification --------------------------------------------------------
## cols(
## X1 = col_double(),
## Resta = col_double(),
## Loneliness = col_double(),
## gender = col_character(),
## age = col_double(),
## ethnicity = col_character(),
## sexuality = col_character(),
## relationship.status = col_character(),
## SWEMWBS = col_double(),
## employment = col_character(),
## ethnicity.dicotomised = col_character(),
## sexuality.dicotomised = col_character(),
## relationship.dicotomised = col_character(),
## employment.dicotomised = col_character()
## )

#add in all cape negative subscale scores and self esteem scores
new.df.cov3$CAPE_neg <- df$CAPE_negative
new.df.cov3$SERS <- df$SERS_total

#remove column loneliness
new.df.cov3 <- new.df.cov3[, -c(3)]

#remove rows with missing data to allow for mediation package to work
new.df.cov3 <- na.omit(new.df.cov3)

Create models including co-variates

model.m3.cov <- lm(SERS ~ Resta + gender + age + ethnicity.dicotomised +
 sexuality.dicotomised + relationship.dicotomised +
 employment.dicotomised, data = new.df.cov3)
model.y3.cov <- lm(CAPE_neg ~ Resta + SERS + gender + age + ethnicity.dicotomised + sexuality.dicotomised + relationship.dicotomised +
 employment.dicotomised, data = new.df.cov3)

Check assumptions and for outliers

new.df.cov3$standardized.residuals <- rstandard(model.y3.cov)
plot(model.y3.cov)


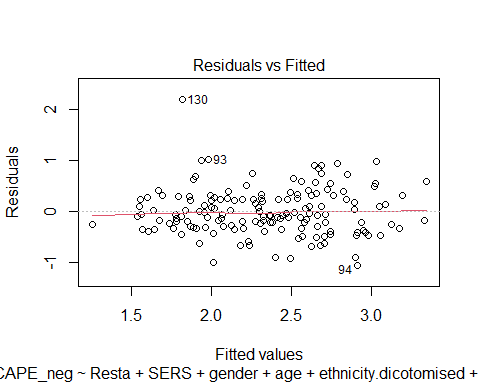

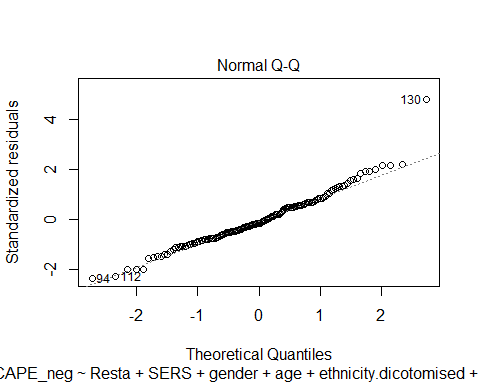

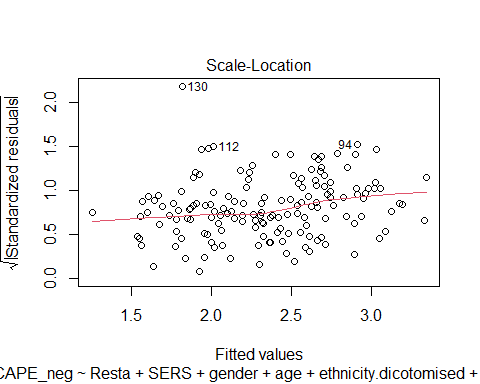

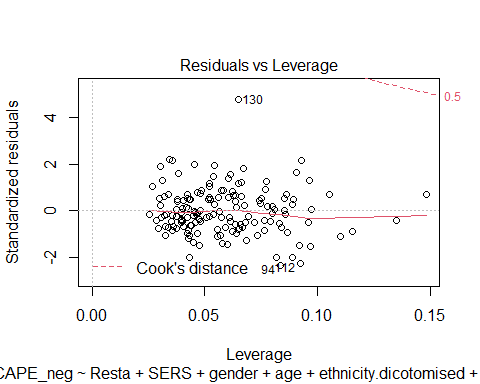


hist(new.df.cov3$standardized.residuals)


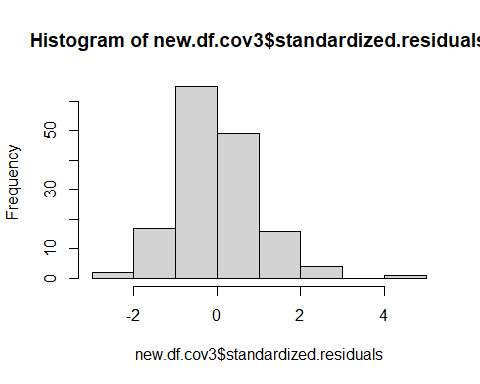


shapiro.test(new.df.cov3$standardized.residuals)

##
## Shapiro-Wilk normality test
##
## data: new.df.cov3$standardized.residuals
## W = 0.96355, p-value = 0.0004322

new.df.cov3$standardized.residuals > 2 |new.df.cov3$standardized.residuals < -2

## 1 2 3 4 5 6 7 8 9 10 11 12 13
## FALSE TRUE FALSE FALSE FALSE FALSE FALSE FALSE FALSE FALSE FALSE FALSE FALSE
## 14 15 16 17 18 19 20 21 22 23 24 25 26
## FALSE FALSE FALSE FALSE FALSE FALSE FALSE FALSE FALSE TRUE FALSE FALSE FALSE
## 27 28 29 30 31 32 33 34 35 36 37 38 39
## FALSE FALSE FALSE FALSE FALSE FALSE FALSE FALSE FALSE FALSE FALSE FALSE FALSE
## 40 41 42 43 44 45 46 47 48 49 50 51 52
## FALSE FALSE FALSE FALSE FALSE FALSE FALSE FALSE FALSE FALSE FALSE FALSE FALSE
## 53 54 55 56 57 58 59 60 61 62 63 64 65
## FALSE FALSE FALSE FALSE FALSE FALSE FALSE FALSE FALSE FALSE FALSE FALSE FALSE
## 66 67 68 69 70 71 72 73 74 75 76 77 78
## FALSE FALSE FALSE FALSE FALSE FALSE FALSE FALSE FALSE FALSE FALSE FALSE FALSE
## 79 80 81 82 83 84 85 86 87 88 89 90 91
## FALSE FALSE FALSE FALSE FALSE FALSE FALSE FALSE FALSE FALSE FALSE FALSE FALSE
## 92 93 94 95 96 97 98 99 100 101 102 103 104
## FALSE TRUE TRUE FALSE FALSE FALSE FALSE FALSE FALSE FALSE FALSE FALSE FALSE
## 105 106 107 108 109 110 111 112 113 114 115 116 117
## FALSE FALSE FALSE FALSE FALSE FALSE FALSE TRUE TRUE FALSE FALSE FALSE FALSE
## 118 119 120 121 122 123 124 125 126 127 128 129 130
## FALSE FALSE FALSE FALSE FALSE FALSE FALSE FALSE FALSE FALSE FALSE FALSE TRUE
## 131 132 133 134 135 136 137 138 139 140 141 142 143
## FALSE FALSE FALSE FALSE FALSE FALSE FALSE FALSE FALSE FALSE FALSE FALSE FALSE
## 144 145 146 147 148 149 150 151 152 153 154
## FALSE FALSE FALSE FALSE FALSE FALSE FALSE FALSE FALSE FALSE FALSE

new.df.cov3$large.residual <- new.df.cov3$standardized.residuals > 2 | new.df.cov3$standardized.residuals < -2
sum(new.df.cov3$large.residual)

## [1] 7

new.df.cov3[new.df.cov3$large.residual, c("X1", "standardized.residuals")]

## # A tibble: 7 x 2
## X1 standardized.residuals
## <dbl> <dbl>
## 1 2 2.02
## 2 26 2.15
## 3 105 2.20
## 4 106 -2.34
## 5 130 -2.25
## 6 131 2.16
## 7 154 4.79

new.df.cov3$cooks.distance <- cooks.distance(model.y3.cov)
new.df.cov3$leverage <- hatvalues(model.y3.cov)
new.df.cov3$covariance <- covratio(model.y3.cov)

new.df.cov3[new.df.cov3$large.residual, c("X1", "cooks.distance", "leverage", "covariance" )]

## # A tibble: 7 x 4
## X1 cooks.distance leverage covariance
## <dbl> <dbl> <dbl> <dbl>
## 1 2 0.0215 0.0454 0.863
## 2 26 0.0189 0.0355 0.825
## 3 105 0.0192 0.0345 0.812
## 4 106 0.0552 0.0834 0.822
## 5 130 0.0569 0.0922 0.852
## 6 131 0.0533 0.0929 0.873
## 7 154 0.176 0.0648 0.242

Remove outlier

new.df.cov3 %>%
 filter (! (X1 == 154)) -> new.df.cov3

Build models again

model.m3.cov <- lm(SERS ~ Resta + gender + age + ethnicity.dicotomised +
 sexuality.dicotomised + relationship.dicotomised +
 employment.dicotomised, data = new.df.cov3)
model.y3.cov <- lm(CAPE_neg ~ Resta + SERS + gender + age + ethnicity.dicotomised + sexuality.dicotomised + relationship.dicotomised +
 employment.dicotomised, data = new.df.cov3)

Re-check assumptions

new.df.cov3$standardized.residuals <- rstandard(model.y3.cov)
plot(model.y3.cov)


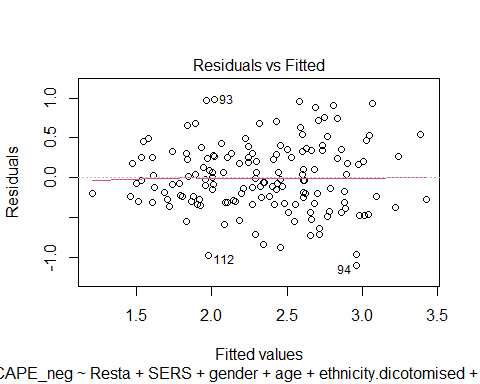

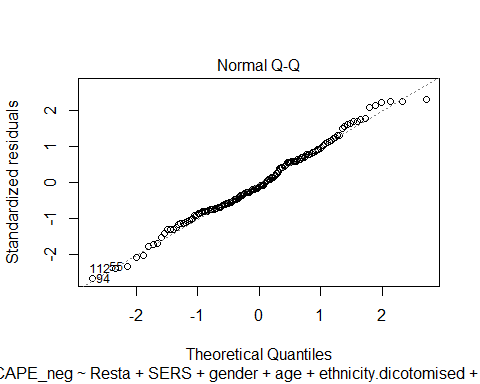

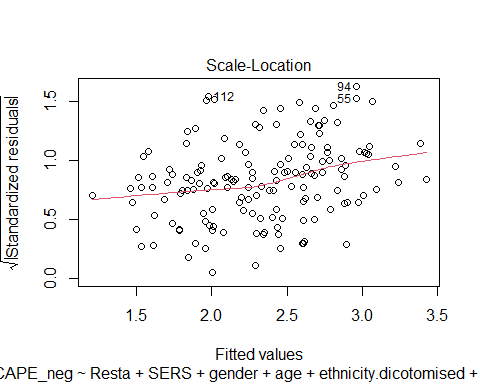

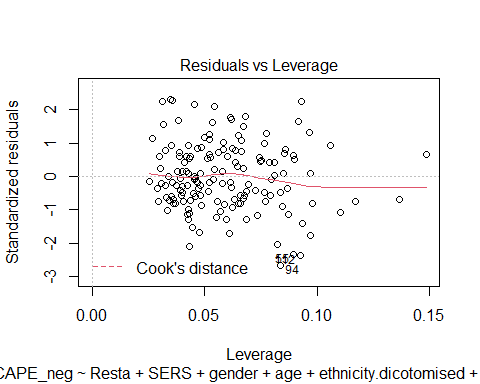


hist(new.df.cov3$standardized.residuals)


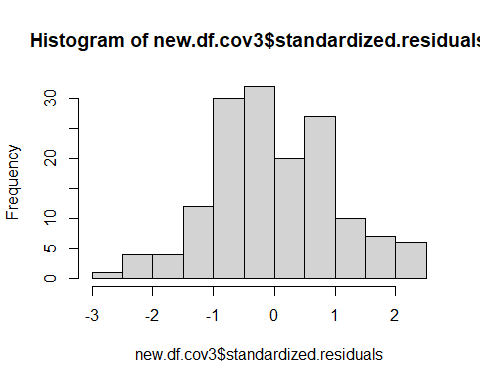


shapiro.test(new.df.cov3$standardized.residuals)

##
## Shapiro-Wilk normality test
##
## data: new.df.cov3$standardized.residuals
## W = 0.98966, p-value = 0.3236

Run mediation analysis

med.cov3 <- mediate(model.m3.cov, model.y3.cov, sims = 5000, treat = "Resta", mediator = "SERS")

summary(med.cov3)

##
## Causal Mediation Analysis
##
## Quasi-Bayesian Confidence Intervals
##
## Estimate 95% CI Lower 95% CI Upper p-value
## ACME -0.03757 -0.05458 -0.02 <2e-16 ***
## ADE -0.00888 -0.02823 0.01 0.3856
## Total Effect -0.04645 -0.06865 -0.02 0.0004 ***
## Prop. Mediated 0.80956 0.51505 1.36 0.0004 ***
## ---
## Signif. codes: 0 '***' 0.001 '**' 0.01 '*' 0.05 '.' 0.1 ' ' 1
##
## Sample Size Used: 153
##
##
## Simulations: 5000

## CAPE DEPRESSIVE

Testing mediation model : Resta -> self esteem -> CAPE depressive

Create data frame

new.df4 <- data.frame(df$Resta.total, df$SERS_total, df$CAPE_depressive)

#remove any rows with NAs
new.df4%>%
 filter(! is.na(df.SERS_total)) -> new.df4

Build models

model.m4 <- lm(df.SERS_total ~ df.Resta.total, data = new.df4)
model.y4 <- lm(df.CAPE_depressive ~ df.Resta.total + df.SERS_total, data = new.df4)

Check parametric assumptions and for outliers

new.df4$standardized.residuals <- rstandard(model.y4)
plot(model.y4)


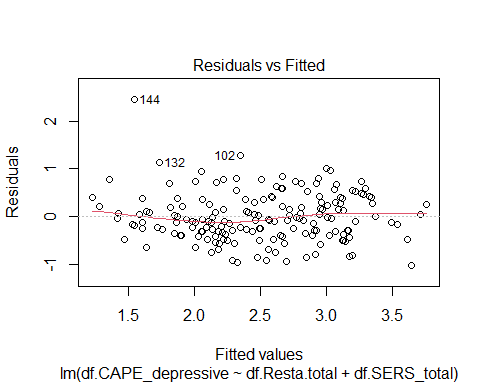

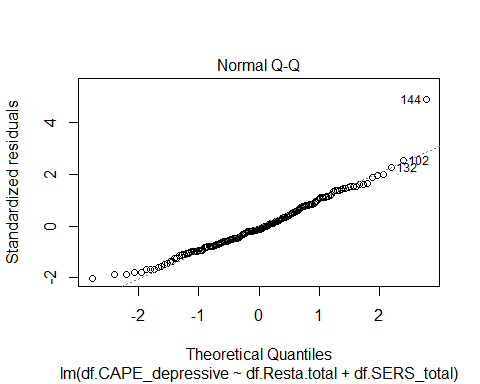

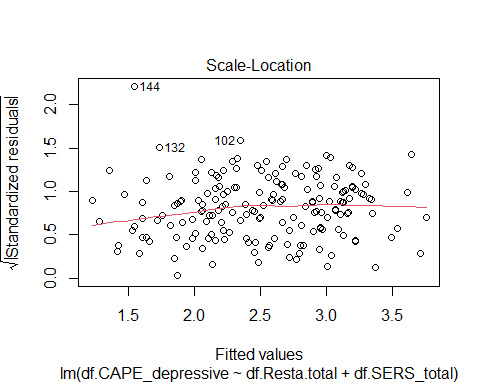

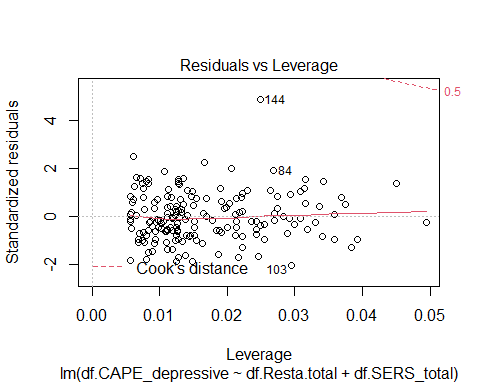


hist(new.df4$standardized.residuals)


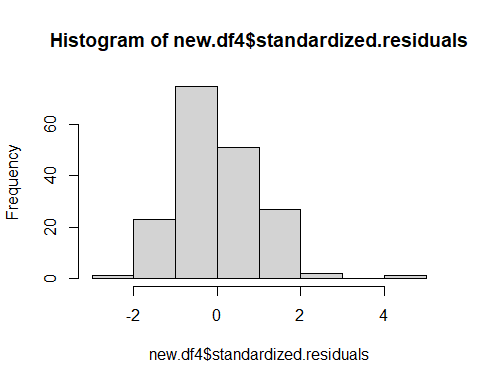


shapiro.test(new.df4$standardized.residuals)

##
## Shapiro-Wilk normality test
##
## data: new.df4$standardized.residuals
## W = 0.96561, p-value = 0.0002055

new.df4$standardized.residuals > 2 | new.df4$standardized.residuals < -2

## [1] FALSE FALSE FALSE FALSE FALSE FALSE FALSE FALSE FALSE FALSE FALSE FALSE
## [13] FALSE FALSE FALSE FALSE FALSE FALSE FALSE FALSE FALSE FALSE FALSE FALSE
## [25] FALSE FALSE FALSE FALSE FALSE FALSE FALSE FALSE FALSE FALSE FALSE FALSE
## [37] FALSE FALSE FALSE FALSE FALSE FALSE FALSE FALSE FALSE FALSE FALSE FALSE
## [49] FALSE FALSE FALSE FALSE FALSE FALSE FALSE FALSE FALSE FALSE FALSE FALSE
## [61] FALSE FALSE FALSE FALSE FALSE FALSE FALSE FALSE FALSE FALSE FALSE FALSE
## [73] FALSE FALSE FALSE FALSE FALSE FALSE FALSE FALSE FALSE FALSE FALSE FALSE
## [85] FALSE FALSE FALSE FALSE FALSE FALSE FALSE FALSE FALSE FALSE FALSE FALSE
## [97] FALSE FALSE FALSE FALSE FALSE TRUE TRUE FALSE FALSE FALSE FALSE FALSE
## [109] FALSE FALSE FALSE FALSE FALSE FALSE FALSE FALSE FALSE FALSE FALSE FALSE
## [121] FALSE FALSE FALSE FALSE FALSE FALSE FALSE FALSE FALSE FALSE FALSE TRUE
## [133] FALSE FALSE FALSE FALSE FALSE FALSE FALSE FALSE FALSE FALSE FALSE TRUE
## [145] FALSE FALSE FALSE FALSE FALSE FALSE FALSE FALSE FALSE FALSE FALSE FALSE
## [157] FALSE FALSE FALSE FALSE FALSE FALSE FALSE FALSE FALSE FALSE FALSE FALSE
## [169] FALSE FALSE FALSE FALSE FALSE FALSE FALSE FALSE FALSE FALSE FALSE FALSE

new.df4$large.residual <- new.df4$standardized.residuals > 2 | new.df4$standardized.residuals < -2
sum(new.df4$large.residual)

## [1] 4

new.df4[new.df4$large.residual, c( "standardized.residuals")]

## [1] 2.523924 -2.024010 2.263257 4.879454

new.df4$cooks.distance <- cooks.distance(model.y4)
new.df4$leverage <- hatvalues(model.y4)
new.df4$covariance <- covratio(model.y4)

new.df4[new.df4$large.residual, c("cooks.distance", "leverage", "covariance" )]

## cooks.distance leverage covariance
## 102 0.01304480 0.006105853 0.9168253
## 103 0.04155779 0.029534421 0.9769950
## 132 0.02890343 0.016646104 0.9471320
## 144 0.20218094 0.024842415 0.6762174

Run with outlier then remove outlier and rebuild models

med.with4 <- mediate(model.m4 , model.y4, sims = 1000, boot = TRUE,
 treat = "df.Resta.total",
 mediator = "df.SERS_total")

## Running nonparametric bootstrap

summary(med.with4)

##
## Causal Mediation Analysis
##
## Nonparametric Bootstrap Confidence Intervals with the Percentile Method
##
## Estimate 95% CI Lower 95% CI Upper p-value
## ACME -0.02947 -0.04799 -0.01 <2e-16 ***
## ADE -0.00342 -0.01854 0.01 0.67
## Total Effect -0.03288 -0.05835 -0.01 0.01 **
## Prop. Mediated 0.89612 0.54252 1.81 0.01 **
## ---
## Signif. codes: 0 '***' 0.001 '**' 0.01 '*' 0.05 '.' 0.1 ' ' 1
##
## Sample Size Used: 180
##
##
## Simulations: 1000

new.df4.OR <- new.df4[-c(144),]

model.m4.OR <- lm(df.SERS_total ~ df.Resta.total, data = new.df4.OR)
model.y4.OR <- lm(df.CAPE_depressive ~ df.Resta.total + df.SERS_total, data = new.df4.OR )

Re-check parametric assumptions for new models

new.df4.OR$standardized.residuals <- rstandard(model.y4.OR)
plot(model.y4.OR)


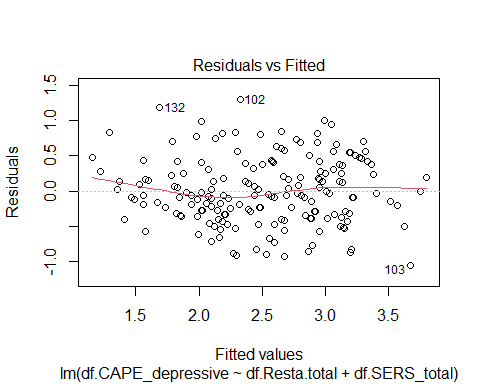

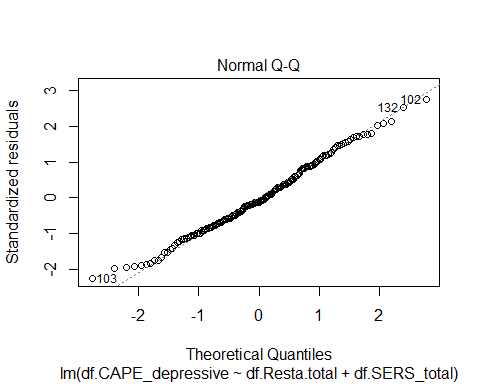

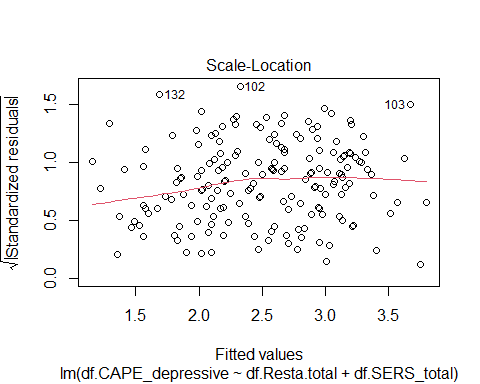

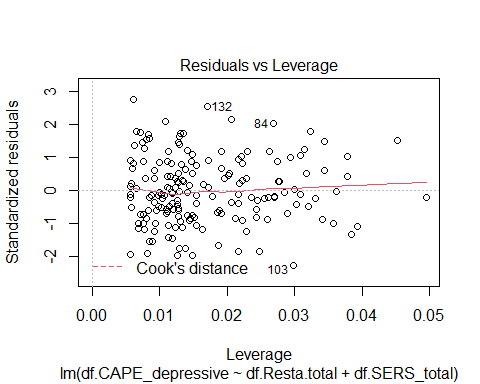


hist(new.df4.OR$standardized.residuals)


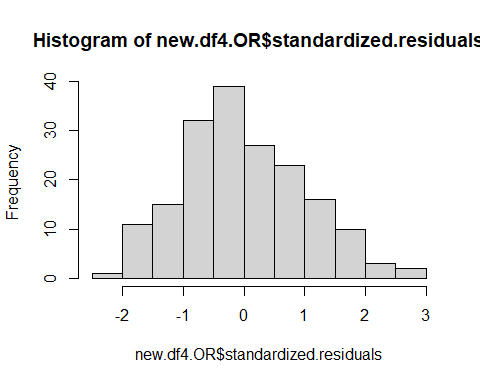


shapiro.test(new.df4.OR$standardized.residuals)

##
## Shapiro-Wilk normality test
##
## data: new.df4.OR$standardized.residuals
## W = 0.99095, p-value = 0.3201

Re-run mediation analysis

med.without4<- mediate(model.m4.OR , model.y4.OR, sims = 1000,
 treat = "df.Resta.total",
 mediator = "df.SERS_total")
summary(med.without4)

##
## Causal Mediation Analysis
##
## Quasi-Bayesian Confidence Intervals
##
## Estimate 95% CI Lower 95% CI Upper p-value
## ACME -0.0292 -0.0481 -0.01 <2e-16 ***
## ADE -0.0056 -0.0210 0.01 0.526
## Total Effect -0.0348 -0.0561 -0.01 0.002 **
## Prop. Mediated 0.8390 0.4960 1.58 0.002 **
## ---
## Signif. codes: 0 '***' 0.001 '**' 0.01 '*' 0.05 '.' 0.1 ' ' 1
##
## Sample Size Used: 179
##
##
## Simulations: 1000

Add in co-variates

#Upload previous dataframe to work from

new.df.cov4 <- read_csv("Z:/Online study IRAS ID 271957/Online analysis/new.df.cov_2022.csv")

## Warning: Missing column names filled in: 'X1' [1]

##
## -- Column specification --------------------------------------------------------
## cols(
## X1 = col_double(),
## Resta = col_double(),
## Loneliness = col_double(),
## gender = col_character(),
## age = col_double(),
## ethnicity = col_character(),
## sexuality = col_character(),
## relationship.status = col_character(),
## SWEMWBS = col_double(),
## employment = col_character(),
## ethnicity.dicotomised = col_character(),
## sexuality.dicotomised = col_character(),
## relationship.dicotomised = col_character(),
## employment.dicotomised = col_character()
## )

#add in cape depressive subscale scores and self-esteem scores
new.df.cov4$CAPE_dep <- df$CAPE_depressive
new.df.cov4$SERS <- df$SERS_total

#remove column loneliness
new.df.cov4 <- new.df.cov4[, -c(3)]

#remove rows with missing data to allow for mediation package to work
new.df.cov4 <- na.omit(new.df.cov4)

Add covariates into models

model.m4.cov <- lm(SERS~ Resta + gender + age + ethnicity.dicotomised +
 sexuality.dicotomised + relationship.dicotomised +
 employment.dicotomised, data = new.df.cov4)
model.y4.cov <- lm(CAPE_dep ~ Resta + SERS + gender + age + ethnicity.dicotomised + sexuality.dicotomised + relationship.dicotomised +
 employment.dicotomised, data = new.df.cov4)

Check assumptions and for outliers

new.df.cov4$standardized.residuals <- rstandard(model.y4.cov)
plot(model.y4.cov)


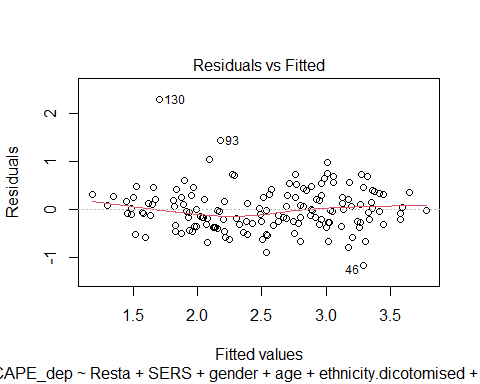

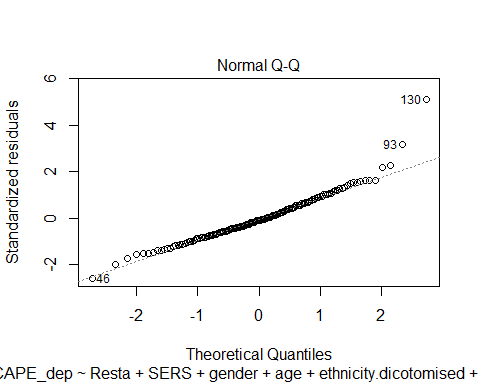

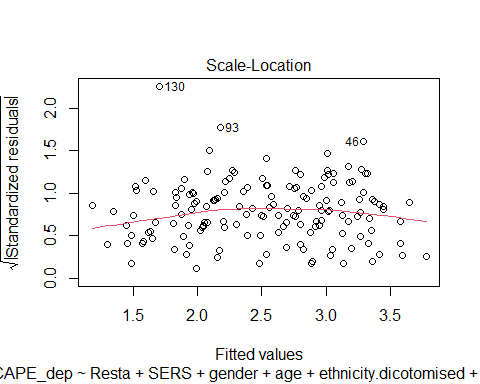

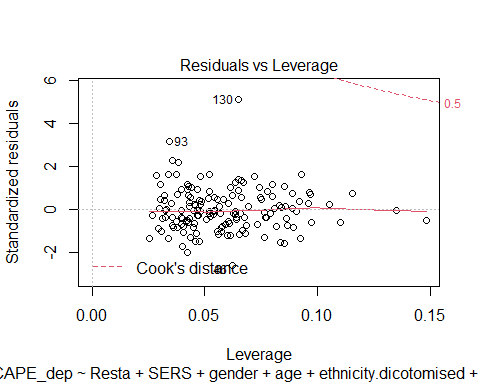


hist(new.df.cov4$standardized.residuals)


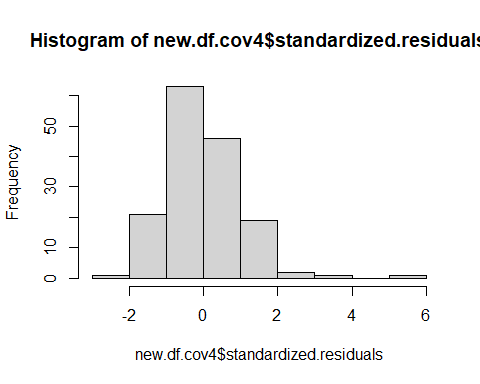


shapiro.test(new.df.cov4$standardized.residuals)

##
## Shapiro-Wilk normality test
##
## data: new.df.cov4$standardized.residuals
## W = 0.94934, p-value = 2.266e-05

new.df.cov4$standardized.residuals > 2 |new.df.cov4$standardized.residuals < -2

## 1 2 3 4 5 6 7 8 9 10 11 12 13
## FALSE FALSE FALSE FALSE FALSE FALSE FALSE FALSE FALSE FALSE FALSE FALSE FALSE
## 14 15 16 17 18 19 20 21 22 23 24 25 26
## FALSE FALSE FALSE FALSE FALSE FALSE FALSE FALSE FALSE TRUE FALSE FALSE FALSE
## 27 28 29 30 31 32 33 34 35 36 37 38 39
## FALSE FALSE FALSE FALSE FALSE FALSE FALSE FALSE FALSE FALSE FALSE FALSE FALSE
## 40 41 42 43 44 45 46 47 48 49 50 51 52
## FALSE FALSE FALSE FALSE FALSE FALSE TRUE FALSE FALSE FALSE FALSE FALSE FALSE
## 53 54 55 56 57 58 59 60 61 62 63 64 65
## FALSE FALSE FALSE FALSE FALSE FALSE FALSE FALSE FALSE FALSE FALSE FALSE FALSE
## 66 67 68 69 70 71 72 73 74 75 76 77 78
## FALSE FALSE FALSE FALSE FALSE FALSE FALSE FALSE FALSE TRUE FALSE FALSE FALSE
## 79 80 81 82 83 84 85 86 87 88 89 90 91
## FALSE FALSE FALSE FALSE FALSE FALSE FALSE FALSE FALSE FALSE FALSE FALSE FALSE
## 92 93 94 95 96 97 98 99 100 101 102 103 104
## FALSE TRUE FALSE FALSE FALSE FALSE FALSE FALSE FALSE FALSE FALSE FALSE FALSE
## 105 106 107 108 109 110 111 112 113 114 115 116 117
## FALSE FALSE FALSE FALSE FALSE FALSE FALSE FALSE FALSE FALSE FALSE FALSE FALSE
## 118 119 120 121 122 123 124 125 126 127 128 129 130
## FALSE FALSE FALSE FALSE FALSE FALSE FALSE FALSE FALSE FALSE FALSE FALSE TRUE
## 131 132 133 134 135 136 137 138 139 140 141 142 143
## FALSE FALSE FALSE FALSE FALSE FALSE FALSE FALSE FALSE FALSE FALSE FALSE FALSE
## 144 145 146 147 148 149 150 151 152 153 154
## FALSE FALSE FALSE FALSE FALSE FALSE FALSE FALSE FALSE FALSE FALSE

new.df.cov4$large.residual <- new.df.cov4$standardized.residuals > 2 | new.df.cov4$standardized.residuals < -2
sum(new.df.cov4$large.residual)

## [1] 5

new.df.cov4[new.df.cov4$large.residual, c("X1", "standardized.residuals")]

## # A tibble: 5 x 2
## X1 standardized.residuals
## <dbl> <dbl>
## 1 26 2.26
## 2 52 -2.59
## 3 87 2.17
## 4 105 3.16
## 5 154 5.10

new.df.cov4$cooks.distance <- cooks.distance(model.y4.cov)
new.df.cov4$leverage <- hatvalues(model.y4.cov)
new.df.cov4$covariance <- covratio(model.y4.cov)

new.df.cov4[new.df.cov4$large.residual, c("X1", "cooks.distance", "leverage", "covariance" )]

## # A tibble: 5 x 4
## X1 cooks.distance leverage covariance
## <dbl> <dbl> <dbl> <dbl>
## 1 26 0.0209 0.0355 0.799
## 2 52 0.0495 0.0622 0.740
## 3 87 0.0209 0.0384 0.823
## 4 105 0.0396 0.0345 0.580
## 5 154 0.200 0.0648 0.192

Remove outliers

new.df.cov4 %>%
 filter (! (X1 == 154)) -> new.df.cov4

new.df.cov4 %>%
 filter (! (X1 == 105)) -> new.df.cov4

Build models again

model.m4.cov <- lm(SERS ~ Resta + gender + age + ethnicity.dicotomised +
 sexuality.dicotomised + relationship.dicotomised +
 employment.dicotomised, data = new.df.cov4)
model.y4.cov <- lm(CAPE_dep ~ Resta + SERS + gender + age + ethnicity.dicotomised + sexuality.dicotomised + relationship.dicotomised +
 employment.dicotomised, data = new.df.cov4)

Check parametric assumptions

new.df.cov4$standardized.residuals <- rstandard(model.y4.cov)
plot(model.y4.cov)


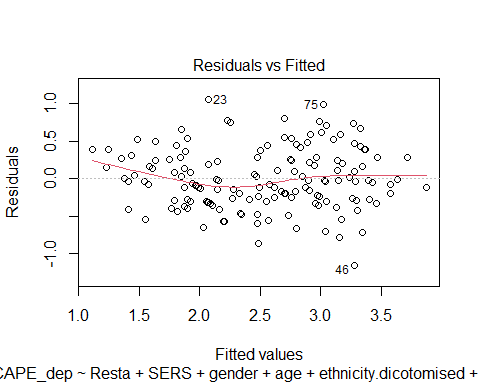

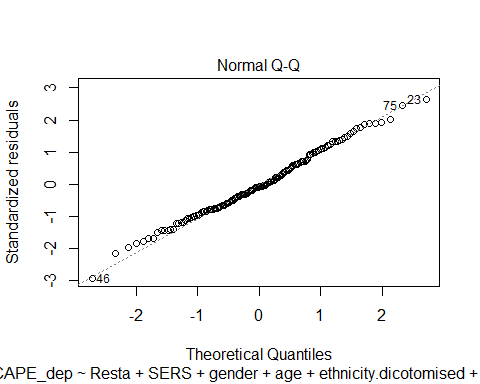

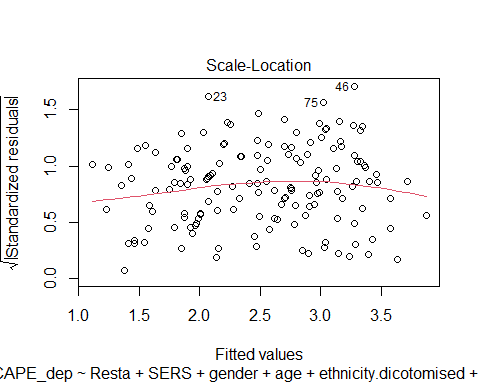

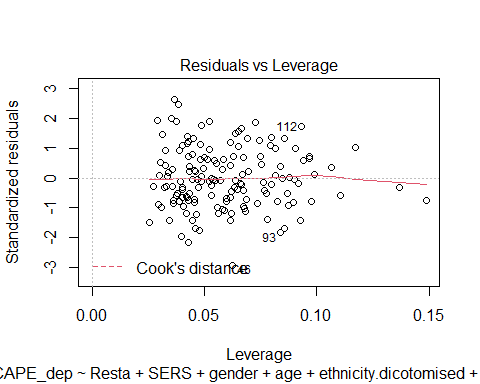


hist(new.df.cov4$standardized.residuals)


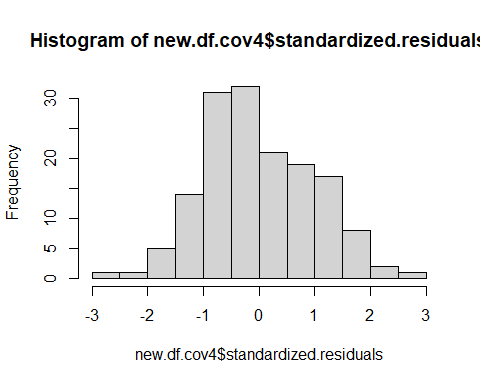


shapiro.test(new.df.cov4$standardized.residuals)

##
## Shapiro-Wilk normality test
##
## data: new.df.cov4$standardized.residuals
## W = 0.99341, p-value = 0.7185

Run mediation analysis with covariates and without outliers

med.cov4 <- mediate(model.m4.cov, model.y4.cov, sims = 5000, treat = "Resta", mediator = "SERS")

summary(med.cov4)

##
## Causal Mediation Analysis
##
## Quasi-Bayesian Confidence Intervals
##
## Estimate 95% CI Lower 95% CI Upper p-value
## ACME -0.0504 -0.0714 -0.03 <2e-16 ***
## ADE -0.0265 -0.0445 -0.01 0.0036 **
## Total Effect -0.0769 -0.1028 -0.05 <2e-16 ***
## Prop. Mediated 0.6562 0.4669 0.87 <2e-16 ***
## ---
## Signif. codes: 0 '***' 0.001 '**' 0.01 '*' 0.05 '.' 0.1 ' ' 1
##
## Sample Size Used: 152
##
##
## Simulations: 5000
