# Supplementary material for: Being more satisfied with romantic relationship status is associated with increased mental wellbeing in people with experience of psychosis
Source: Front Psychiatry. 2023 Sep 28;14:1232973. doi: 10.3389/fpsyt.2023.1232973 (PMC10569177; doi:10.3389/fpsyt.2023.1232973)
Supplement: Supplementary file 8 [file Data_Sheet_8.DOCX]

Mediation analysis (Internalised stigma)

Rebecca White

09/08/2022

Install packages and dataset

library(readr)
library(mediation)

## Loading required package: MASS

## Loading required package: Matrix

## Loading required package: mvtnorm

## Loading required package: sandwich

## mediation: Causal Mediation Analysis
## Version: 4.5.0

library(tidyverse)

## -- Attaching packages --------------------------------------- tidyverse 1.3.0 --

## v ggplot2 3.3.2 v dplyr 1.0.2
## v tibble 3.0.4 v stringr 1.4.0
## v tidyr 1.1.2 v forcats 0.5.0
## v purrr 0.3.4

## -- Conflicts ------------------------------------------ tidyverse_conflicts() --
## x tidyr::expand() masks Matrix::expand()
## x dplyr::filter() masks stats::filter()
## x dplyr::lag() masks stats::lag()
## x tidyr::pack() masks Matrix::pack()
## x dplyr::select() masks MASS::select()
## x tidyr::unpack() masks Matrix::unpack()

df <- read_csv("Z:/Online study IRAS ID 271957/Online analysis/Dataset_190_obs_2.9.21.csv")

## Warning: Missing column names filled in: 'X1' [1]

##
## -- Column specification --------------------------------------------------------
## cols(
## .default = col_double(),
## redcap_survey_identifier = col_logical(),
## pis_timestamp = col_datetime(format = ""),
## screening_questions_timestamp = col_datetime(format = ""),
## demographic_information_timestamp = col_datetime(format = ""),
## nationality = col_character(),
## ethnicity_other = col_character(),
## gender_self_describe = col_character(),
## sexual_orientation_selfdescribe = col_character(),
## rr_selfdescribe = col_character(),
## last_rr_end = col_character(),
## current_rr_length = col_character(),
## the_community_assessment_of_psychic_experiences_ca_timestamp = col_datetime(format = ""),
## the_short_warwick_mental_health_wellbeing_scale_timestamp = col_datetime(format = ""),
## adapted_satisfaction_with_relationships_scale_rest_timestamp = col_datetime(format = ""),
## three_item_loneliness_scale_timestamp = col_datetime(format = ""),
## internalised_stigma_of_mental_illness_inventory_10_timestamp = col_datetime(format = ""),
## multidimensional_scale_of_perceived_social_support_timestamp = col_datetime(format = ""),
## self_esteem_rating_scale_short_form_serssf_timestamp = col_datetime(format = ""),
## relationships_questionnaire_timestamp = col_datetime(format = ""),
## Screening_Qs_result = col_character()
## # ... with 7 more columns
## )
## i Use `spec()` for the full column specifications.

## SWEMWBS

testing mediation model : resta -> internalised stigma -> SWEMWBS

Create dataframe to work from

new.df <- data.frame(df$X1, df$Resta.total, df$ISMI_total, df$SWEMWBS_metric)

#remove any rows with NAs
new.df %>%
 filter(! is.na(df.ISMI_total) & ! is.na(df.SWEMWBS_metric)) -> new.df

# remove rows with value of 'Inf' from ISMI total
new.df %>%
 filter( ! (df.ISMI_total == Inf)) -> new.df

Create model without covariates first

model.m <- lm(df.ISMI_total ~ df.Resta.total, data = new.df)
model.y <- lm(df.SWEMWBS_metric ~ df.Resta.total + df.ISMI_total, data = new.df )

Check parametric assumptions

new.df$standardized.residuals <- rstandard(model.y)
plot(model.y)


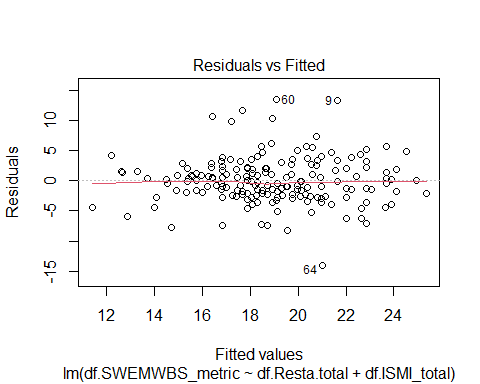

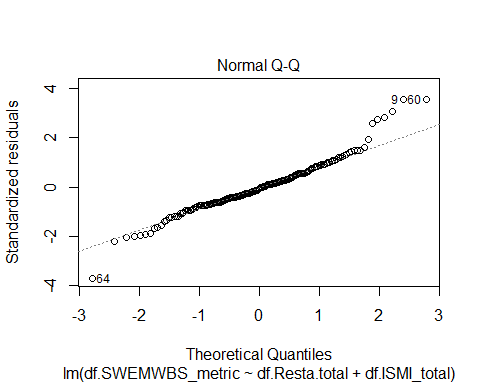

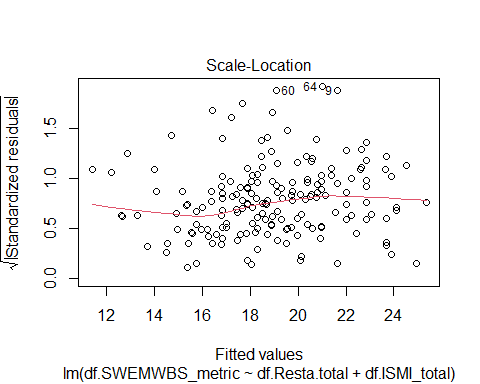

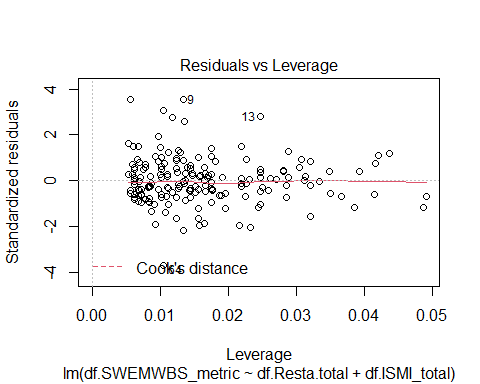


hist(new.df$standardized.residuals)


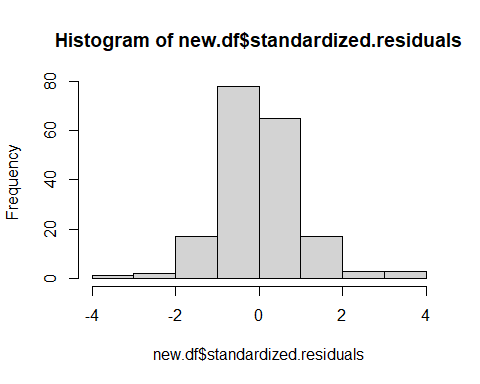


shapiro.test(new.df$standardized.residuals)

##
## Shapiro-Wilk normality test
##
## data: new.df$standardized.residuals
## W = 0.95854, p-value = 2.756e-05

new.df$standardized.residuals > 2 | new.df$standardized.residuals < -2

## [1] FALSE FALSE FALSE FALSE FALSE FALSE FALSE FALSE TRUE FALSE FALSE FALSE
## [13] TRUE FALSE FALSE FALSE FALSE FALSE FALSE FALSE FALSE FALSE FALSE FALSE
## [25] FALSE FALSE FALSE FALSE FALSE FALSE FALSE FALSE FALSE FALSE FALSE FALSE
## [37] FALSE FALSE FALSE TRUE FALSE FALSE FALSE FALSE FALSE FALSE FALSE FALSE
## [49] FALSE FALSE FALSE FALSE FALSE FALSE FALSE FALSE FALSE FALSE FALSE TRUE
## [61] FALSE FALSE FALSE TRUE FALSE FALSE FALSE FALSE FALSE FALSE FALSE FALSE
## [73] FALSE FALSE FALSE FALSE FALSE FALSE FALSE FALSE FALSE FALSE FALSE FALSE
## [85] FALSE FALSE FALSE FALSE FALSE FALSE FALSE FALSE FALSE FALSE FALSE FALSE
## [97] FALSE FALSE FALSE FALSE FALSE FALSE FALSE FALSE FALSE FALSE FALSE FALSE
## [109] FALSE FALSE FALSE FALSE FALSE FALSE FALSE FALSE FALSE FALSE FALSE FALSE
## [121] FALSE FALSE FALSE FALSE FALSE FALSE FALSE FALSE FALSE FALSE FALSE FALSE
## [133] FALSE FALSE FALSE FALSE FALSE FALSE TRUE FALSE FALSE FALSE FALSE FALSE
## [145] FALSE FALSE FALSE FALSE FALSE FALSE FALSE FALSE FALSE FALSE FALSE FALSE
## [157] FALSE FALSE FALSE FALSE FALSE FALSE FALSE FALSE TRUE FALSE FALSE FALSE
## [169] FALSE FALSE FALSE FALSE FALSE FALSE FALSE FALSE FALSE FALSE FALSE FALSE
## [181] TRUE FALSE FALSE FALSE TRUE FALSE

new.df$large.residual <- new.df$standardized.residuals > 2 | new.df$standardized.residuals < -2
sum(new.df$large.residual)

## [1] 9

new.df[new.df$large.residual, c("df.X1", "standardized.residuals")]

## df.X1 standardized.residuals
## 9 9 3.535619
## 13 13 2.817141
## 40 40 2.596769
## 60 60 3.540963
## 64 64 -3.693667
## 139 142 2.743539
## 165 169 3.073266
## 181 185 -2.193625
## 185 189 -2.047156

new.df$cooks.distance <- cooks.distance(model.y)
new.df$leverage <- hatvalues(model.y)
new.df$covariance <- covratio(model.y)

new.df[new.df$large.residual, c("cooks.distance", "leverage", "covariance" )]

## cooks.distance leverage covariance
## 9 0.05654521 0.013388510 0.8333131
## 13 0.06699373 0.024698865 0.9125056
## 40 0.03086315 0.013544787 0.9207591
## 60 0.02385882 0.005676168 0.8262996
## 64 0.04807936 0.010461578 0.8142566
## 139 0.02988874 0.011772358 0.9069006
## 165 0.03362619 0.010567802 0.8764183
## 181 0.02180467 0.013411644 0.9512295
## 185 0.03337503 0.023333888 0.9709770

Run analysis with outliers then remove outliers and re-run analysis

med.with <- mediate(model.m, model.y, sims = 1000, boot = TRUE, treat = "df.Resta.total",
 mediator = "df.ISMI_total")

## Running nonparametric bootstrap

summary(med.with)

##
## Causal Mediation Analysis
##
## Nonparametric Bootstrap Confidence Intervals with the Percentile Method
##
## Estimate 95% CI Lower 95% CI Upper p-value
## ACME 0.1043 0.0213 0.19 0.014 *
## ADE 0.2130 0.0964 0.33 <2e-16 ***
## Total Effect 0.3173 0.1802 0.45 <2e-16 ***
## Prop. Mediated 0.3287 0.0881 0.57 0.014 *
## ---
## Signif. codes: 0 '***' 0.001 '**' 0.01 '*' 0.05 '.' 0.1 ' ' 1
##
## Sample Size Used: 186
##
##
## Simulations: 1000

new.df.OR <- new.df
new.df.OR <- new.df.OR[-c(9, 60, 64, 165),]

model.m.OR <- lm(df.ISMI_total ~ df.Resta.total, data = new.df.OR)
model.y.OR <- lm(df.SWEMWBS_metric ~ df.Resta.total + df.ISMI_total, data = new.df.OR )

Re-check parametric assumptions

new.df.OR$standardized.residuals <- rstandard(model.y.OR)
plot(model.y.OR)


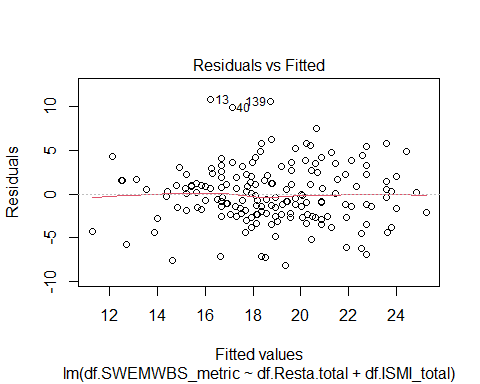

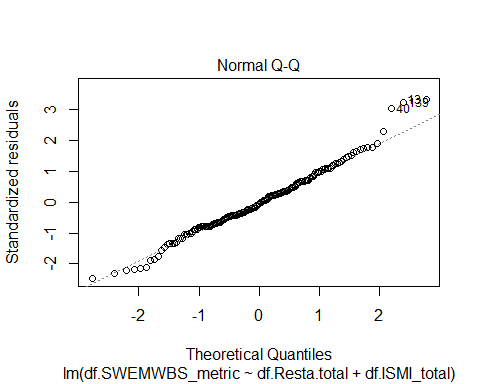

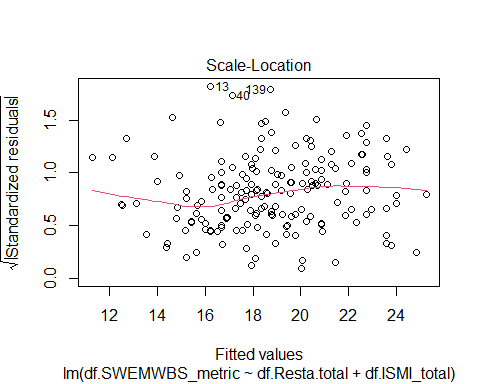

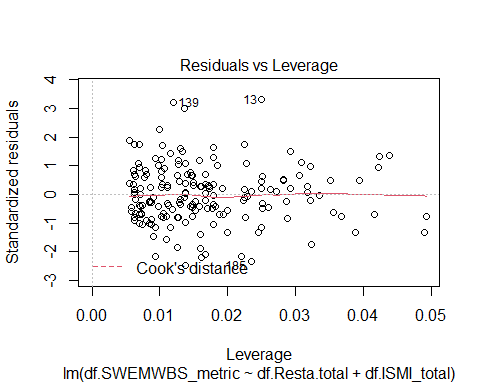


hist(new.df.OR$standardized.residuals)


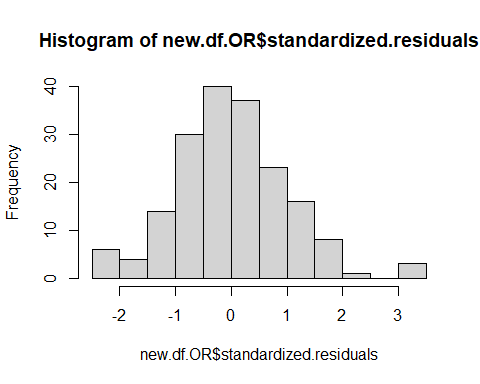


shapiro.test(new.df.OR$standardized.residuals)

##
## Shapiro-Wilk normality test
##
## data: new.df.OR$standardized.residuals
## W = 0.98451, p-value = 0.04174

Re-run analysis

med.without<- mediate(model.m.OR , model.y.OR, sims = 1000, boot = TRUE, treat = "df.Resta.total", mediator = "df.ISMI_total")

## Running nonparametric bootstrap

summary(med.without)

##
## Causal Mediation Analysis
##
## Nonparametric Bootstrap Confidence Intervals with the Percentile Method
##
## Estimate 95% CI Lower 95% CI Upper p-value
## ACME 0.1048 0.0220 0.19 0.006 **
## ADE 0.2043 0.0925 0.31 <2e-16 ***
## Total Effect 0.3090 0.1740 0.45 <2e-16 ***
## Prop. Mediated 0.3391 0.1100 0.62 0.006 **
## ---
## Signif. codes: 0 '***' 0.001 '**' 0.01 '*' 0.05 '.' 0.1 ' ' 1
##
## Sample Size Used: 182
##
##
## Simulations: 1000

Add in covariates : gender, age, ethnicity, sexuality, relationship status, employment

Create dataframe to work from using file previously saved

new.df.cov <- read_csv("Z:/Online study IRAS ID 271957/Online analysis/new.df.cov_2022.csv")

## Warning: Missing column names filled in: 'X1' [1]

##
## -- Column specification --------------------------------------------------------
## cols(
## X1 = col_double(),
## Resta = col_double(),
## Loneliness = col_double(),
## gender = col_character(),
## age = col_double(),
## ethnicity = col_character(),
## sexuality = col_character(),
## relationship.status = col_character(),
## SWEMWBS = col_double(),
## employment = col_character(),
## ethnicity.dicotomised = col_character(),
## sexuality.dicotomised = col_character(),
## relationship.dicotomised = col_character(),
## employment.dicotomised = col_character()
## )

#remove column loneliness
new.df.cov <- new.df.cov[, -c(3)]

# add in internalised stigma
new.df.cov$ISMI <- df$ISMI_total

#remove rows with missing data to allow for mediation package to work
new.df.cov <- na.omit(new.df.cov)

#remove 'Inf' rows from ismi column
new.df.cov %>%
 filter( ! (ISMI == Inf)) -> new.df.cov

Add covariates into models

model.m.cov <- lm(ISMI ~ Resta + gender + age + ethnicity.dicotomised +
 sexuality.dicotomised + relationship.dicotomised +
 employment.dicotomised, data = new.df.cov)
model.y.cov <- lm(SWEMWBS ~ Resta + ISMI + gender + age + ethnicity.dicotomised +
 sexuality.dicotomised + relationship.dicotomised +
 employment.dicotomised, data = new.df.cov)

Check assumptions

new.df.cov$standardized.residuals <- rstandard(model.y.cov)
plot(model.y.cov)


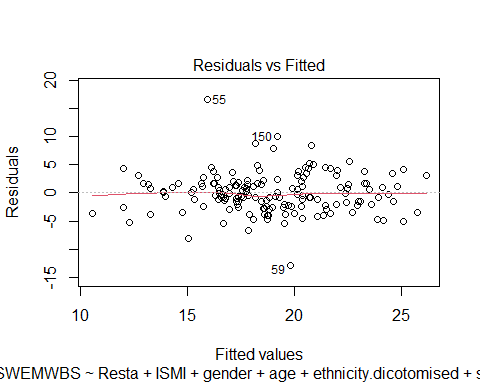

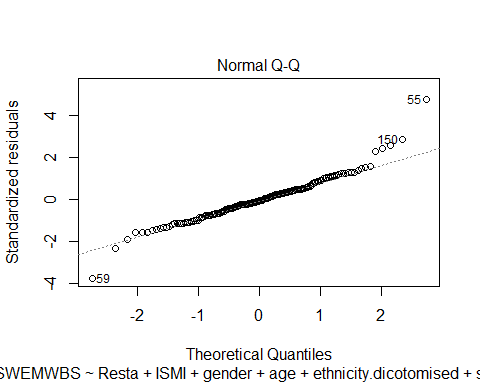

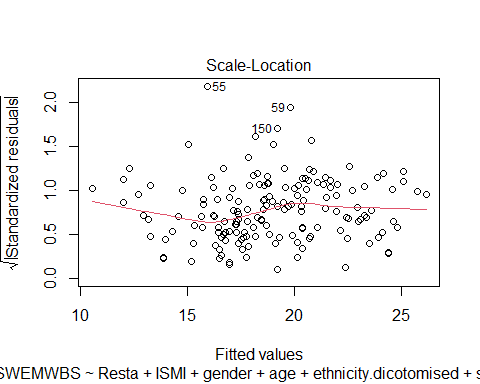

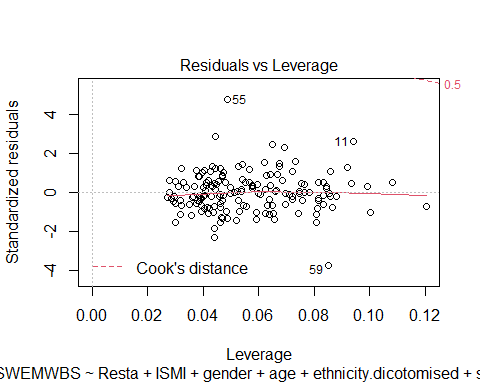


hist(new.df.cov$standardized.residuals)


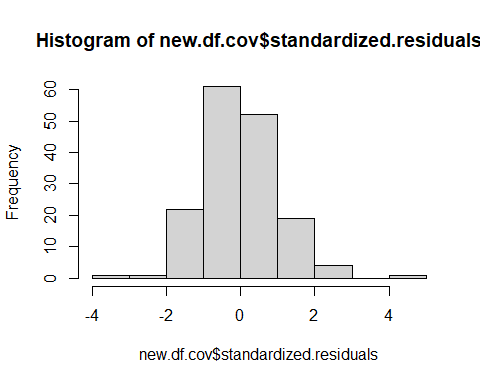


shapiro.test(new.df.cov$standardized.residuals)

##
## Shapiro-Wilk normality test
##
## data: new.df.cov$standardized.residuals
## W = 0.95463, p-value = 4.352e-05

Check for outliers

new.df.cov$standardized.residuals > 2 | new.df.cov$standardized.residuals < -2

## 1 2 3 4 5 6 7 8 9 10 11 12 13
## FALSE FALSE FALSE FALSE FALSE FALSE FALSE FALSE FALSE FALSE TRUE FALSE FALSE
## 14 15 16 17 18 19 20 21 22 23 24 25 26
## FALSE FALSE FALSE FALSE FALSE FALSE FALSE FALSE FALSE FALSE FALSE FALSE FALSE
## 27 28 29 30 31 32 33 34 35 36 37 38 39
## FALSE FALSE FALSE FALSE FALSE FALSE FALSE FALSE FALSE FALSE TRUE FALSE FALSE
## 40 41 42 43 44 45 46 47 48 49 50 51 52
## FALSE FALSE FALSE FALSE FALSE FALSE FALSE FALSE FALSE FALSE FALSE FALSE FALSE
## 53 54 55 56 57 58 59 60 61 62 63 64 65
## FALSE FALSE TRUE FALSE FALSE FALSE TRUE FALSE FALSE FALSE FALSE FALSE FALSE
## 66 67 68 69 70 71 72 73 74 75 76 77 78
## FALSE FALSE FALSE FALSE FALSE FALSE FALSE FALSE FALSE FALSE FALSE FALSE FALSE
## 79 80 81 82 83 84 85 86 87 88 89 90 91
## FALSE FALSE FALSE FALSE FALSE FALSE FALSE FALSE FALSE FALSE FALSE FALSE FALSE
## 92 93 94 95 96 97 98 99 100 101 102 103 104
## FALSE FALSE FALSE FALSE FALSE FALSE FALSE FALSE FALSE FALSE FALSE FALSE FALSE
## 105 106 107 108 109 110 111 112 113 114 115 116 117
## FALSE FALSE FALSE FALSE FALSE FALSE FALSE FALSE FALSE FALSE FALSE FALSE FALSE
## 118 119 120 121 122 123 124 125 126 127 128 129 130
## FALSE FALSE FALSE FALSE FALSE FALSE FALSE FALSE TRUE FALSE FALSE FALSE FALSE
## 131 132 133 134 135 136 137 138 139 140 141 142 143
## FALSE FALSE FALSE FALSE FALSE FALSE FALSE FALSE FALSE FALSE FALSE FALSE FALSE
## 144 145 146 147 148 149 150 151 152 153 154 155 156
## FALSE FALSE FALSE FALSE FALSE FALSE TRUE FALSE FALSE FALSE FALSE FALSE FALSE
## 157 158 159 160 161
## FALSE FALSE FALSE FALSE TRUE

new.df.cov$large.residual <- new.df.cov$standardized.residuals > 2 | new.df.cov$standardized.residuals < -2
sum(new.df.cov$large.residual)

## [1] 7

new.df.cov[new.df.cov$large.residual, c("X1", "standardized.residuals")]

## # A tibble: 7 x 2
## X1 standardized.residuals
## <dbl> <dbl>
## 1 13 2.59
## 2 40 2.32
## 3 60 4.75
## 4 64 -3.75
## 5 142 2.46
## 6 169 2.88
## 7 189 -2.31

new.df.cov$cooks.distance <- cooks.distance(model.y.cov)
new.df.cov$leverage <- hatvalues(model.y.cov)
new.df.cov$covariance <- covratio(model.y.cov)

new.df.cov[new.df.cov$large.residual, c("X1", "cooks.distance", "leverage", "covariance" )]

## # A tibble: 7 x 4
## X1 cooks.distance leverage covariance
## <dbl> <dbl> <dbl> <dbl>
## 1 13 0.0771 0.0940 0.781
## 2 40 0.0444 0.0694 0.825
## 3 60 0.129 0.0487 0.262
## 4 64 0.145 0.0851 0.485
## 5 142 0.0467 0.0651 0.788
## 6 169 0.0428 0.0443 0.670
## 7 189 0.0274 0.0441 0.805

Remove cases 60 and 64

new.df.cov %>%
 filter ( ! (X1 == 60)) %>%
 filter (!( X1 == 64)) -> new.df.cov

Build models again

model.m.cov <- lm(ISMI ~ Resta + gender + age + ethnicity.dicotomised +
 sexuality.dicotomised + relationship.dicotomised +
 employment.dicotomised, data = new.df.cov)
model.y.cov <- lm(SWEMWBS ~ Resta + ISMI + gender + age + ethnicity.dicotomised +
 sexuality.dicotomised + relationship.dicotomised +
 employment.dicotomised, data = new.df.cov)

Re-check assumptions

new.df.cov$standardized.residuals <- rstandard(model.y.cov)
plot(model.y.cov)


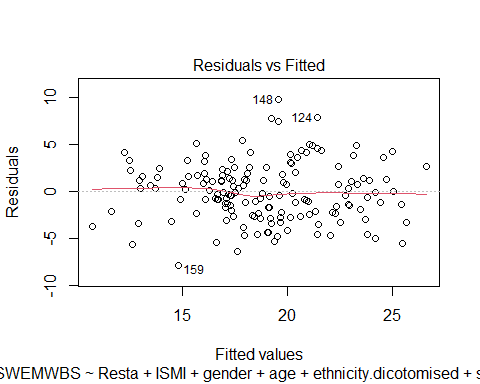

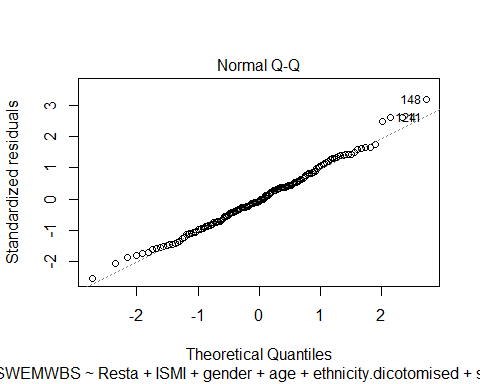

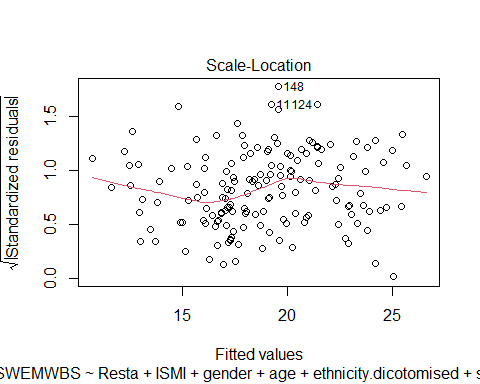

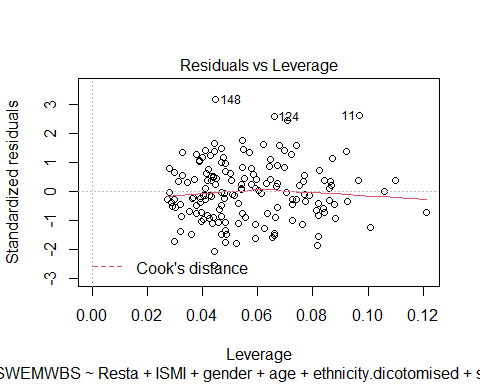


hist(new.df.cov$standardized.residuals)


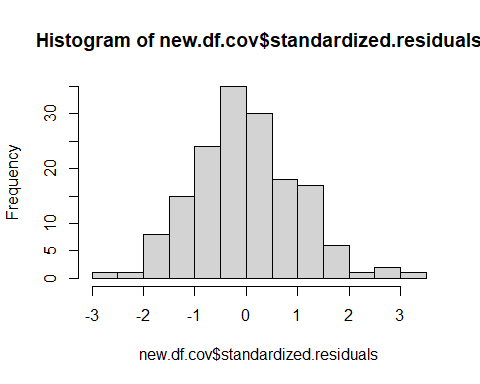


shapiro.test(new.df.cov$standardized.residuals)

##
## Shapiro-Wilk normality test
##
## data: new.df.cov$standardized.residuals
## W = 0.99195, p-value = 0.5141

Run analysis

med.cov<- mediate(model.m.cov , model.y.cov, sims = 5000,
 treat = "Resta", mediator = "ISMI")
summary(med.cov)

##
## Causal Mediation Analysis
##
## Quasi-Bayesian Confidence Intervals
##
## Estimate 95% CI Lower 95% CI Upper p-value
## ACME 0.1528 0.0698 0.25 4e-04 ***
## ADE 0.3770 0.2486 0.51 <2e-16 ***
## Total Effect 0.5299 0.3844 0.68 <2e-16 ***
## Prop. Mediated 0.2866 0.1461 0.45 4e-04 ***
## ---
## Signif. codes: 0 '***' 0.001 '**' 0.01 '*' 0.05 '.' 0.1 ' ' 1
##
## Sample Size Used: 159
##
##
## Simulations: 5000

## CAPE POSITIVE

test mediation model : resta -> internalised stigma -> CAPE pos

Create data frame to work from

new.df2 <- data.frame(df$Resta.total, df$ISMI_total, df$CAPE_positive)

#remove any rows with NAs
new.df2 %>%
 filter(! is.na(df.ISMI_total) & !(df.ISMI_total == "Inf")) -> new.df2

Build models

model.m2 <- lm(df.ISMI_total ~ df.Resta.total, data = new.df2)
model.y2 <- lm(df.CAPE_positive ~ df.Resta.total + df.ISMI_total, data = new.df2)

Check parametric assumptions and for outliers

new.df2$standardized.residuals <- rstandard(model.y2)
plot(model.y2)


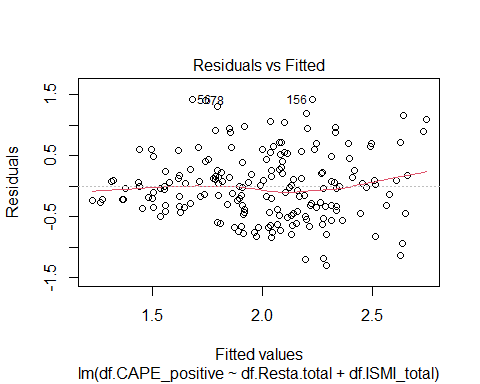

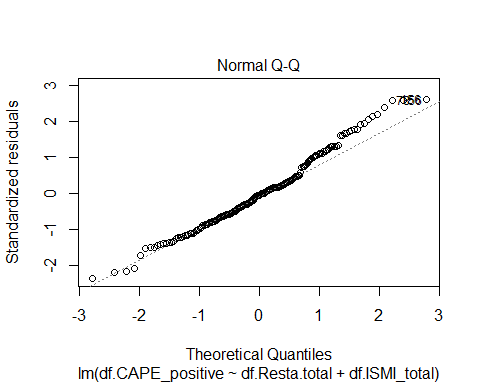

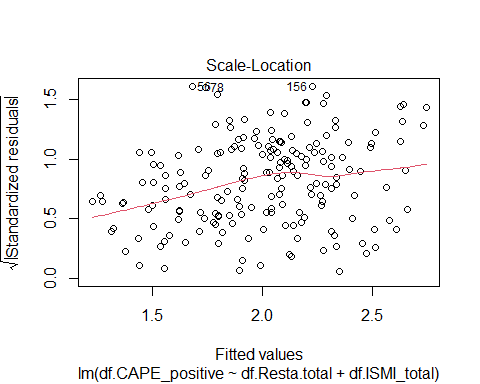

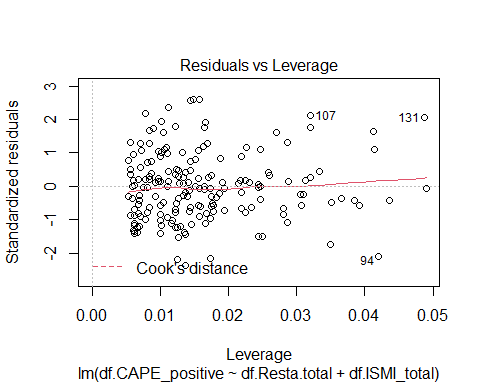


hist(new.df2$standardized.residuals)


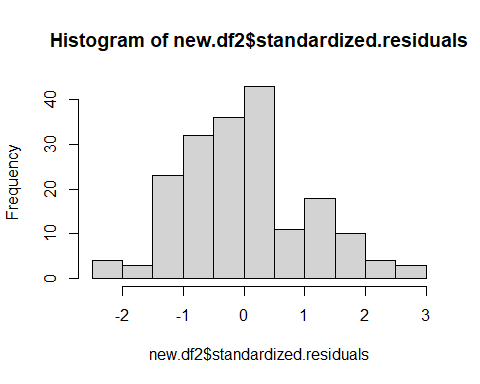


shapiro.test(new.df2$standardized.residuals)

##
## Shapiro-Wilk normality test
##
## data: new.df2$standardized.residuals
## W = 0.9831, p-value = 0.02349

new.df2$standardized.residuals > 2 | new.df2$standardized.residuals < -2

## [1] FALSE FALSE FALSE FALSE FALSE FALSE FALSE FALSE FALSE FALSE FALSE FALSE
## [13] FALSE FALSE FALSE FALSE FALSE FALSE FALSE FALSE FALSE FALSE FALSE TRUE
## [25] FALSE FALSE FALSE FALSE FALSE TRUE FALSE FALSE FALSE FALSE FALSE FALSE
## [37] FALSE FALSE FALSE FALSE FALSE FALSE FALSE FALSE FALSE FALSE FALSE FALSE
## [49] FALSE FALSE TRUE FALSE FALSE FALSE FALSE TRUE FALSE FALSE FALSE FALSE
## [61] FALSE FALSE FALSE FALSE FALSE FALSE FALSE FALSE FALSE FALSE FALSE FALSE
## [73] FALSE FALSE FALSE FALSE FALSE TRUE FALSE FALSE FALSE FALSE FALSE FALSE
## [85] FALSE FALSE FALSE FALSE FALSE FALSE FALSE FALSE FALSE TRUE FALSE FALSE
## [97] FALSE FALSE FALSE FALSE FALSE FALSE FALSE FALSE FALSE FALSE TRUE FALSE
## [109] FALSE TRUE FALSE FALSE FALSE FALSE FALSE FALSE FALSE FALSE FALSE FALSE
## [121] FALSE FALSE FALSE FALSE FALSE FALSE FALSE FALSE FALSE TRUE TRUE FALSE
## [133] FALSE FALSE FALSE FALSE FALSE FALSE FALSE FALSE FALSE FALSE FALSE FALSE
## [145] FALSE FALSE FALSE FALSE FALSE FALSE FALSE FALSE FALSE FALSE FALSE TRUE
## [157] FALSE FALSE FALSE FALSE FALSE FALSE FALSE FALSE FALSE FALSE FALSE FALSE
## [169] FALSE FALSE FALSE FALSE FALSE FALSE FALSE FALSE FALSE FALSE FALSE FALSE
## [181] FALSE FALSE FALSE FALSE FALSE FALSE FALSE

new.df2$large.residual <- new.df2$standardized.residuals > 2 | new.df2$standardized.residuals < -2
sum(new.df2$large.residual)

## [1] 11

new.df2[new.df2$large.residual, c( "standardized.residuals")]

## [1] -2.354860 -2.177637 2.179104 2.586273 2.566759 -2.083294 2.133408
## [8] 2.371561 -2.154509 2.049655 2.594668

new.df2$cooks.distance <- cooks.distance(model.y2)
new.df2$leverage <- hatvalues(model.y2)
new.df2$covariance <- covratio(model.y2)

new.df2[new.df2$large.residual, c("cooks.distance", "leverage", "covariance" )]

## cooks.distance leverage covariance
## 24 0.02559452 0.013657330 0.9401613
## 30 0.01992539 0.012448494 0.9517478
## 51 0.01246368 0.007812772 0.9471997
## 56 0.03568753 0.015754067 0.9241693
## 78 0.03202200 0.014371850 0.9244443
## 94 0.06350555 0.042050785 0.9877740
## 107 0.05028663 0.032082210 0.9741554
## 110 0.02121536 0.011189636 0.9365711
## 130 0.02731649 0.017347993 0.9580979
## 131 0.07174137 0.048733931 0.9970247
## 156 0.03376441 0.014822836 0.9226165

No outliers to remove, run analysis

med.with2 <- mediate(model.m2 , model.y2, sims = 1000, boot = TRUE,
 treat = "df.Resta.total",
 mediator = "df.ISMI_total")

## Running nonparametric bootstrap

summary(med.with2)

##
## Causal Mediation Analysis
##
## Nonparametric Bootstrap Confidence Intervals with the Percentile Method
##
## Estimate 95% CI Lower 95% CI Upper p-value
## ACME -0.01477 -0.02757 0.00 0.01 **
## ADE 0.00476 -0.01268 0.02 0.66
## Total Effect -0.01001 -0.03123 0.01 0.37
## Prop. Mediated 1.47515 -8.46834 11.34 0.38
## ---
## Signif. codes: 0 '***' 0.001 '**' 0.01 '*' 0.05 '.' 0.1 ' ' 1
##
## Sample Size Used: 187
##
##
## Simulations: 1000

Add in co-variates

#upload previous dataframe to work from and add in CAPE positive variable
new.df.cov2 <- read_csv("Z:/Online study IRAS ID 271957/Online analysis/new.df.cov_2022.csv")

## Warning: Missing column names filled in: 'X1' [1]

##
## -- Column specification --------------------------------------------------------
## cols(
## X1 = col_double(),
## Resta = col_double(),
## Loneliness = col_double(),
## gender = col_character(),
## age = col_double(),
## ethnicity = col_character(),
## sexuality = col_character(),
## relationship.status = col_character(),
## SWEMWBS = col_double(),
## employment = col_character(),
## ethnicity.dicotomised = col_character(),
## sexuality.dicotomised = col_character(),
## relationship.dicotomised = col_character(),
## employment.dicotomised = col_character()
## )

new.df.cov2$CAPE_pos <- df$CAPE_positive

#remove column loneliness
new.df.cov2 <- new.df.cov2[, -c(3)]

# add in ISMI
new.df.cov2$ISMI <- df$ISMI_total

# remove ISMI rows with missing or 'Inf'
new.df.cov2 %>%
 filter(! is.na(ISMI) & !(ISMI == "Inf")) -> new.df.cov2

#remove rows with missing data to allow for mediation package to work
new.df.cov2 <- na.omit(new.df.cov2)

Create model with co-variates

model.m2.cov <- lm(ISMI ~ Resta + gender + age + ethnicity.dicotomised +
 sexuality.dicotomised + relationship.dicotomised +
 employment.dicotomised, data = new.df.cov2)
model.y2.cov <- lm(CAPE_pos ~ Resta + ISMI+ gender + age + ethnicity.dicotomised +
 sexuality.dicotomised + relationship.dicotomised +
 employment.dicotomised, data = new.df.cov2)

Check parametric assumptions and for outliers

new.df.cov2$standardized.residuals <- rstandard(model.y2.cov)
plot(model.y2.cov)


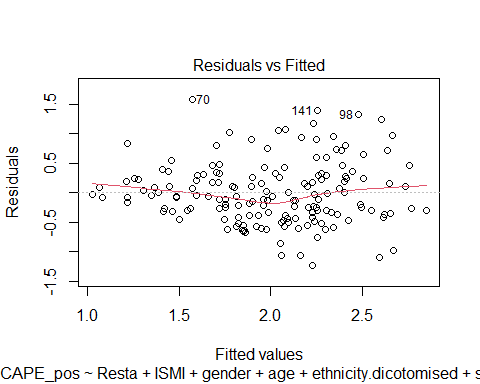

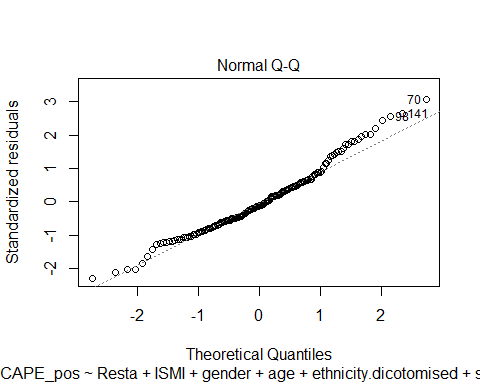

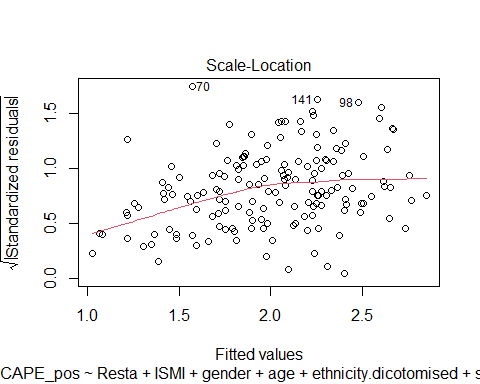

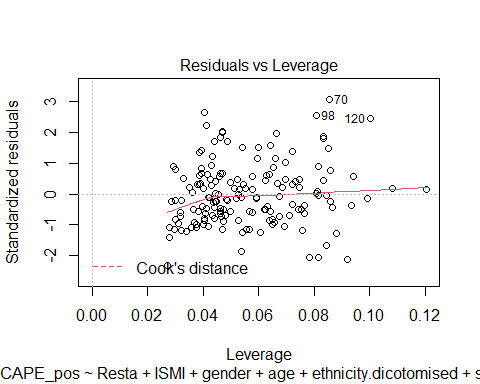


hist(new.df.cov2$standardized.residuals)


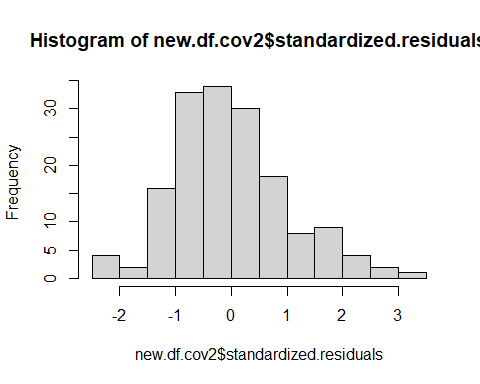


shapiro.test(new.df.cov2$standardized.residuals)

##
## Shapiro-Wilk normality test
##
## data: new.df.cov2$standardized.residuals
## W = 0.97559, p-value = 0.005975

new.df.cov2$standardized.residuals > 2 |new.df.cov2$standardized.residuals < -2

## 1 2 3 4 5 6 7 8 9 10 11 12 13
## FALSE FALSE FALSE FALSE FALSE FALSE FALSE FALSE FALSE FALSE FALSE FALSE FALSE
## 14 15 16 17 18 19 20 21 22 23 24 25 26
## FALSE FALSE FALSE FALSE FALSE FALSE FALSE FALSE TRUE FALSE FALSE FALSE FALSE
## 27 28 29 30 31 32 33 34 35 36 37 38 39
## FALSE TRUE FALSE FALSE FALSE FALSE FALSE FALSE FALSE FALSE FALSE FALSE FALSE
## 40 41 42 43 44 45 46 47 48 49 50 51 52
## FALSE FALSE FALSE FALSE FALSE FALSE FALSE TRUE FALSE FALSE FALSE FALSE TRUE
## 53 54 55 56 57 58 59 60 61 62 63 64 65
## FALSE FALSE FALSE FALSE FALSE FALSE FALSE FALSE FALSE FALSE FALSE FALSE FALSE
## 66 67 68 69 70 71 72 73 74 75 76 77 78
## FALSE FALSE FALSE FALSE TRUE FALSE FALSE FALSE FALSE TRUE FALSE FALSE FALSE
## 79 80 81 82 83 84 85 86 87 88 89 90 91
## FALSE FALSE FALSE FALSE FALSE FALSE TRUE FALSE FALSE FALSE FALSE FALSE FALSE
## 92 93 94 95 96 97 98 99 100 101 102 103 104
## FALSE FALSE FALSE FALSE FALSE FALSE TRUE FALSE FALSE FALSE FALSE FALSE FALSE
## 105 106 107 108 109 110 111 112 113 114 115 116 117
## FALSE FALSE FALSE FALSE FALSE FALSE FALSE FALSE FALSE FALSE FALSE FALSE FALSE
## 118 119 120 121 122 123 124 125 126 127 128 129 130
## FALSE TRUE TRUE FALSE FALSE FALSE FALSE FALSE FALSE FALSE FALSE FALSE FALSE
## 131 132 133 134 135 136 137 138 139 140 141 142 143
## FALSE FALSE FALSE FALSE FALSE FALSE FALSE FALSE FALSE FALSE TRUE FALSE FALSE
## 144 145 146 147 148 149 150 151 152 153 154 155 156
## FALSE FALSE FALSE FALSE FALSE FALSE FALSE FALSE FALSE FALSE FALSE FALSE FALSE
## 157 158 159 160 161
## FALSE FALSE FALSE FALSE FALSE

new.df.cov2$large.residual <- new.df.cov2$standardized.residuals > 2 | new.df.cov2$standardized.residuals < -2
sum(new.df.cov2$large.residual)

## [1] 11

new.df.cov2[new.df.cov2$large.residual, c("X1", "standardized.residuals")]

## # A tibble: 11 x 2
## X1 standardized.residuals
## <dbl> <dbl>
## 1 24 -2.30
## 2 30 -2.05
## 3 51 2.21
## 4 56 2.01
## 5 78 3.06
## 6 84 2.03
## 7 94 -2.13
## 8 107 2.56
## 9 130 -2.05
## 10 131 2.44
## 11 159 2.65

new.df.cov2$cooks.distance <- cooks.distance(model.y2.cov)
new.df.cov2$leverage <- hatvalues(model.y2.cov)
new.df.cov2$covariance <- covratio(model.y2.cov)

new.df.cov2[new.df.cov2$large.residual, c("X1", "cooks.distance", "leverage", "covariance" )]

## # A tibble: 11 x 4
## X1 cooks.distance leverage covariance
## <dbl> <dbl> <dbl> <dbl>
## 1 24 0.0164 0.0270 0.792
## 2 30 0.0414 0.0816 0.899
## 3 51 0.0235 0.0413 0.824
## 4 56 0.0220 0.0469 0.874
## 5 78 0.0973 0.0854 0.654
## 6 84 0.0226 0.0470 0.869
## 7 94 0.0509 0.0919 0.890
## 8 107 0.0639 0.0808 0.777
## 9 130 0.0395 0.0783 0.895
## 10 131 0.0734 0.100 0.824
## 11 159 0.0327 0.0404 0.723

Remove one outlier

new.df.cov2 %>%
 filter (! (X1 == 78)) -> new.df.cov2

Build models again

model.m2.cov <- lm(ISMI~ Resta + gender + age + ethnicity.dicotomised +
 sexuality.dicotomised + relationship.dicotomised +
 employment.dicotomised, data = new.df.cov2)
model.y2.cov <- lm(CAPE_pos ~ Resta + ISMI + gender + age + ethnicity.dicotomised +
 sexuality.dicotomised + relationship.dicotomised +
 employment.dicotomised, data = new.df.cov2)

Re-check assumptions

new.df.cov2$standardized.residuals <- rstandard(model.y2.cov)
plot(model.y2.cov)


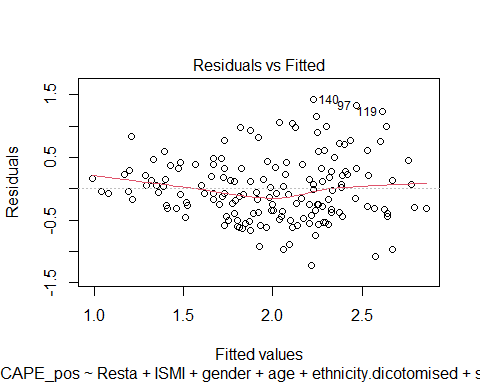

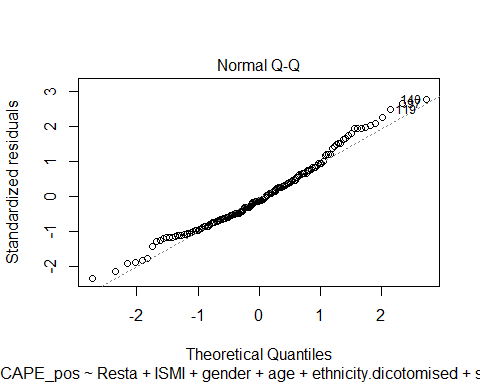

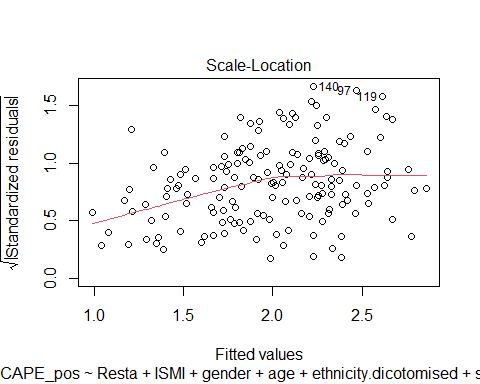

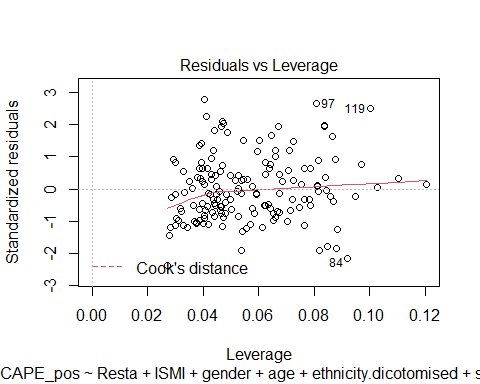


hist(new.df.cov2$standardized.residuals)


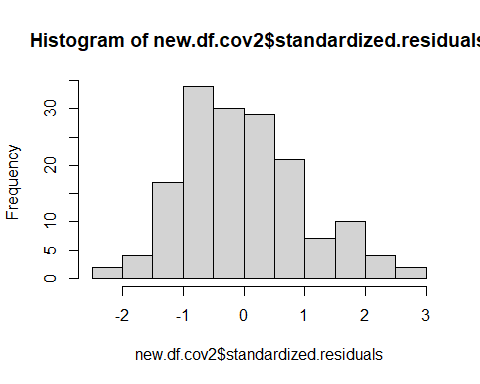


shapiro.test(new.df.cov2$standardized.residuals)

##
## Shapiro-Wilk normality test
##
## data: new.df.cov2$standardized.residuals
## W = 0.97788, p-value = 0.0114

Re-run mediation analysis

med.cov2<- mediate(model.m2.cov , model.y2.cov, sims = 5000, boot = TRUE,
 treat = "Resta", mediator = "ISMI")

## Running nonparametric bootstrap

summary(med.cov2)

##
## Causal Mediation Analysis
##
## Nonparametric Bootstrap Confidence Intervals with the Percentile Method
##
## Estimate 95% CI Lower 95% CI Upper p-value
## ACME -0.02277 -0.03850 -0.01 0.0012 **
## ADE -0.00633 -0.02943 0.02 0.5824
## Total Effect -0.02911 -0.05618 0.00 0.0396 *
## Prop. Mediated 0.78242 0.27263 3.25 0.0400 *
## ---
## Signif. codes: 0 '***' 0.001 '**' 0.01 '*' 0.05 '.' 0.1 ' ' 1
##
## Sample Size Used: 160
##
##
## Simulations: 5000

## CAPE NEGATIVE

Test mediation model : Resta -> internalised stigma -> CAPE negative

Create data frame to work from

new.df3 <- data.frame(df$Resta.total, df$ISMI_total, df$CAPE_negative)

#remove any rows with NAs
new.df3 %>%
 filter(! is.na(df.ISMI_total) & ! (df.ISMI_total == "Inf")) -> new.df3

Build models and run mediation analysis

model.m3 <- lm(df.ISMI_total ~ df.Resta.total, data = new.df3)
model.y3 <- lm(df.CAPE_negative ~ df.Resta.total + df.ISMI_total, data = new.df3)

Check parametric assumptions and for outliers

new.df3$standardized.residuals <- rstandard(model.y3)
plot(model.y3)


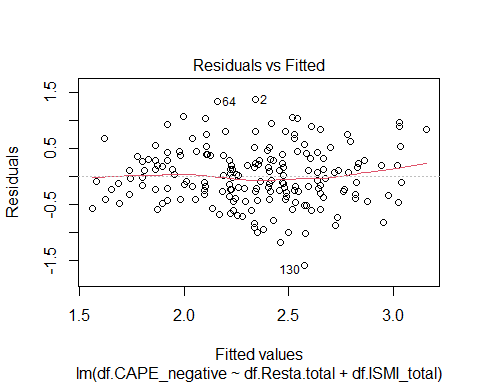

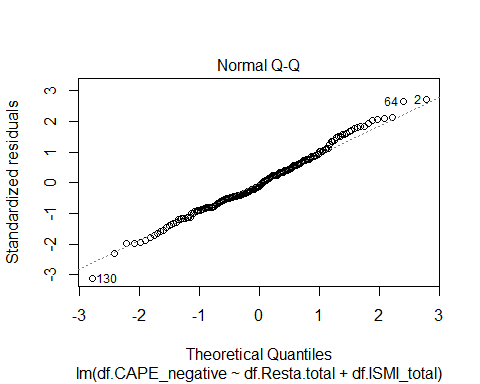

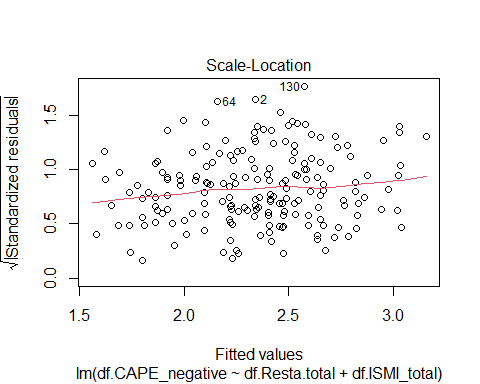

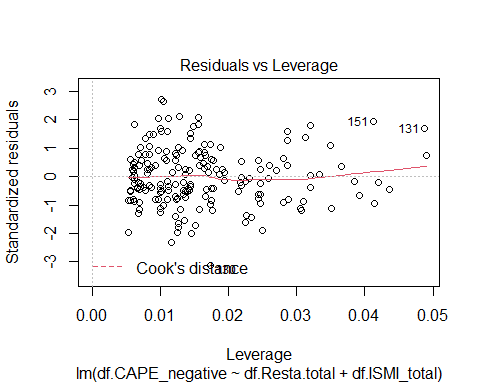


hist(new.df3$standardized.residuals)


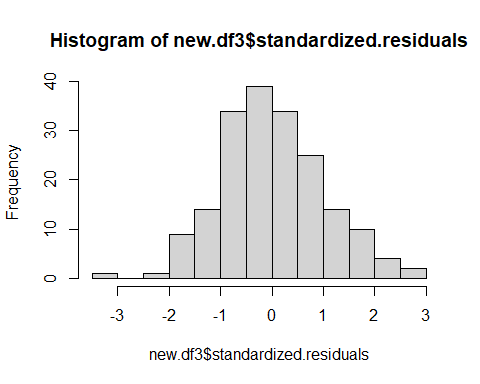


shapiro.test(new.df3$standardized.residuals)

##
## Shapiro-Wilk normality test
##
## data: new.df3$standardized.residuals
## W = 0.99295, p-value = 0.5072

new.df3$standardized.residuals > 2 | new.df3$standardized.residuals < -2

## [1] FALSE TRUE FALSE FALSE FALSE FALSE FALSE FALSE FALSE FALSE FALSE FALSE
## [13] FALSE FALSE FALSE FALSE FALSE FALSE FALSE FALSE FALSE FALSE FALSE FALSE
## [25] FALSE FALSE FALSE FALSE FALSE FALSE FALSE FALSE FALSE FALSE FALSE FALSE
## [37] FALSE FALSE FALSE FALSE FALSE FALSE FALSE FALSE FALSE FALSE FALSE FALSE
## [49] FALSE FALSE FALSE FALSE FALSE FALSE FALSE FALSE FALSE FALSE FALSE FALSE
## [61] FALSE FALSE FALSE TRUE FALSE FALSE FALSE FALSE FALSE TRUE FALSE FALSE
## [73] FALSE FALSE FALSE FALSE FALSE FALSE TRUE FALSE FALSE FALSE FALSE FALSE
## [85] FALSE FALSE FALSE FALSE FALSE FALSE FALSE FALSE FALSE FALSE FALSE FALSE
## [97] FALSE FALSE FALSE FALSE FALSE FALSE FALSE FALSE FALSE FALSE FALSE FALSE
## [109] FALSE TRUE FALSE FALSE FALSE FALSE FALSE FALSE FALSE FALSE FALSE FALSE
## [121] FALSE FALSE FALSE FALSE FALSE FALSE FALSE FALSE FALSE TRUE FALSE FALSE
## [133] FALSE FALSE FALSE FALSE FALSE FALSE FALSE TRUE FALSE FALSE FALSE FALSE
## [145] FALSE FALSE FALSE FALSE FALSE FALSE FALSE FALSE FALSE FALSE FALSE FALSE
## [157] FALSE FALSE FALSE TRUE FALSE FALSE FALSE FALSE FALSE FALSE FALSE FALSE
## [169] FALSE FALSE FALSE FALSE FALSE FALSE FALSE FALSE FALSE FALSE FALSE FALSE
## [181] FALSE FALSE FALSE FALSE FALSE FALSE FALSE

new.df3$large.residual <- new.df3$standardized.residuals > 2 | new.df3$standardized.residuals < -2
sum(new.df3$large.residual)

## [1] 8

new.df3[new.df3$large.residual, c("standardized.residuals")]

## [1] 2.704077 2.635555 2.025725 2.114475 2.051027 -3.113922 -2.312312
## [8] 2.081446

new.df3$cooks.distance <- cooks.distance(model.y3)
new.df3$leverage <- hatvalues(model.y3)
new.df3$covariance <- covratio(model.y3)

new.df3[new.df3$large.residual, c("cooks.distance", "leverage", "covariance" )]

## cooks.distance leverage covariance
## 2 0.02491276 0.010117858 0.9092518
## 64 0.02435155 0.010407822 0.9151801
## 70 0.01337002 0.009679834 0.9592655
## 79 0.01936209 0.012825149 0.9564372
## 110 0.01586809 0.011189636 0.9590785
## 130 0.05706157 0.017347993 0.8793570
## 140 0.02098520 0.011637449 0.9413756
## 160 0.02295561 0.015646991 0.9614020

Run analysis with outlier then remove outlier and build models again

med.with3 <- mediate(model.m3 , model.y3, sims = 1000,
 treat = "df.Resta.total",
 mediator = "df.ISMI_total")
summary(med.with3)

##
## Causal Mediation Analysis
##
## Quasi-Bayesian Confidence Intervals
##
## Estimate 95% CI Lower 95% CI Upper p-value
## ACME -0.01491 -0.02691 0.00 0.004 **
## ADE -0.00275 -0.01830 0.01 0.710
## Total Effect -0.01766 -0.03647 0.00 0.092 .
## Prop. Mediated 0.78599 -1.89093 3.87 0.092 .
## ---
## Signif. codes: 0 '***' 0.001 '**' 0.01 '*' 0.05 '.' 0.1 ' ' 1
##
## Sample Size Used: 187
##
##
## Simulations: 1000

new.df3.OR <- new.df3[-c(130),]

model.m3.OR <- lm(df.ISMI_total ~ df.Resta.total, data = new.df3.OR)
model.y3.OR <- lm(df.CAPE_negative ~ df.Resta.total + df.ISMI_total, data = new.df3.OR )

Re-check parametric assumptions

new.df3.OR$standardized.residuals <- rstandard(model.y3.OR)
plot(model.y3.OR)


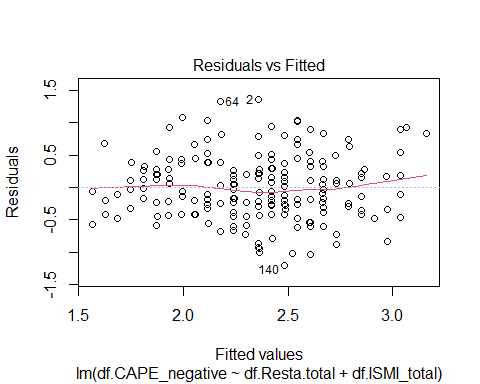

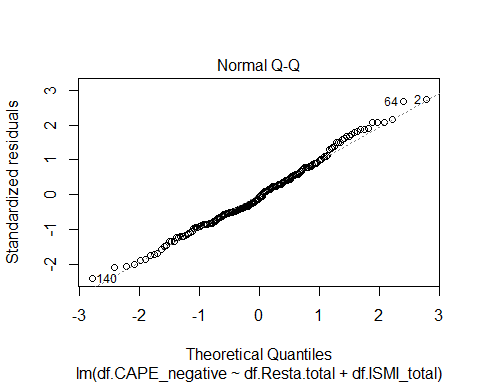

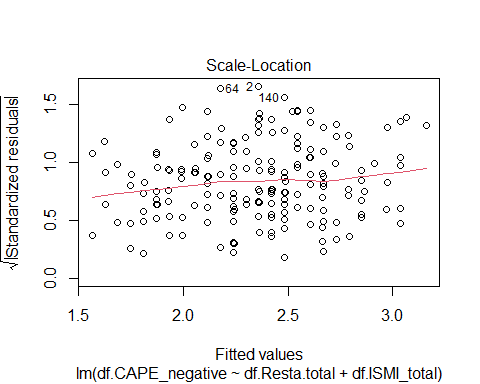

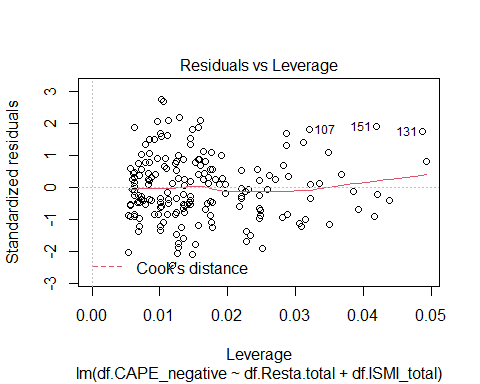


hist(new.df3.OR$standardized.residuals)


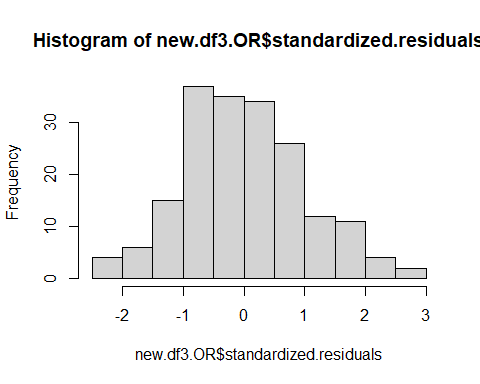


shapiro.test(new.df3.OR$standardized.residuals)

##
## Shapiro-Wilk normality test
##
## data: new.df3.OR$standardized.residuals
## W = 0.99026, p-value = 0.2381

Run mediation analysis

med.without3<- mediate(model.m3.OR , model.y3.OR, sims = 1000,
 treat = "df.Resta.total",
 mediator = "df.ISMI_total")
summary(med.without3)

##
## Causal Mediation Analysis
##
## Quasi-Bayesian Confidence Intervals
##
## Estimate 95% CI Lower 95% CI Upper p-value
## ACME -0.015613 -0.027418 0.00 0.002 **
## ADE -0.000834 -0.016203 0.01 0.914
## Total Effect -0.016447 -0.035644 0.00 0.092 .
## Prop. Mediated 0.889596 -3.299614 5.03 0.094 .
## ---
## Signif. codes: 0 '***' 0.001 '**' 0.01 '*' 0.05 '.' 0.1 ' ' 1
##
## Sample Size Used: 186
##
##
## Simulations: 1000

Add in co-variates

#upload previous data frame to work from and add in CAPE negative variable
new.df.cov3 <- read_csv("Z:/Online study IRAS ID 271957/Online analysis/new.df.cov_2022.csv")

## Warning: Missing column names filled in: 'X1' [1]

##
## -- Column specification --------------------------------------------------------
## cols(
## X1 = col_double(),
## Resta = col_double(),
## Loneliness = col_double(),
## gender = col_character(),
## age = col_double(),
## ethnicity = col_character(),
## sexuality = col_character(),
## relationship.status = col_character(),
## SWEMWBS = col_double(),
## employment = col_character(),
## ethnicity.dicotomised = col_character(),
## sexuality.dicotomised = col_character(),
## relationship.dicotomised = col_character(),
## employment.dicotomised = col_character()
## )

#add in cape negative subscale scores and internalised stigma
new.df.cov3$CAPE_neg <- df$CAPE_negative
new.df.cov3$ISMI <- df$ISMI_total

#remove column loneliness
new.df.cov3 <- new.df.cov3[, -c(3)]

#remove "Inf" rows from ISMI
new.df.cov3 %>%
 filter(! is.na(ISMI) & ! (ISMI == "Inf")) -> new.df.cov3

#remove rows with missing data to allow for mediation package to work
new.df.cov3 <- na.omit(new.df.cov3)

Create models including co-variates

model.m3.cov <- lm(ISMI~ Resta + gender + age + ethnicity.dicotomised +
 sexuality.dicotomised + relationship.dicotomised +
 employment.dicotomised, data = new.df.cov3)
model.y3.cov <- lm(CAPE_neg ~ Resta + ISMI + gender + age + ethnicity.dicotomised +
 sexuality.dicotomised + relationship.dicotomised +
 employment.dicotomised, data = new.df.cov3)

Check assumptions and for outliers

new.df.cov3$standardized.residuals <- rstandard(model.y3.cov)
plot(model.y3.cov)


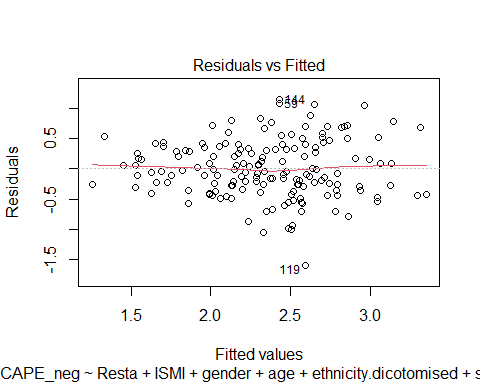

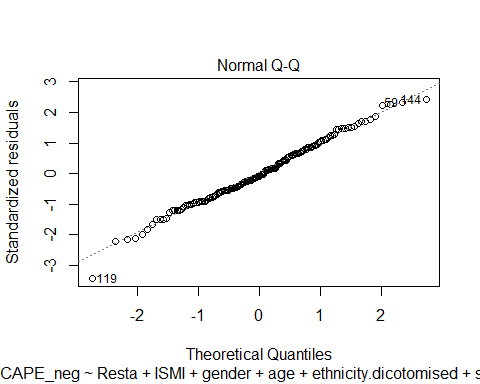

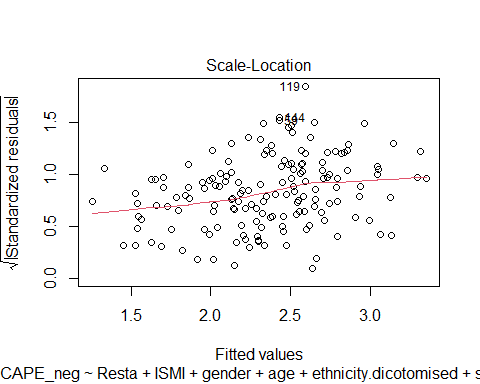

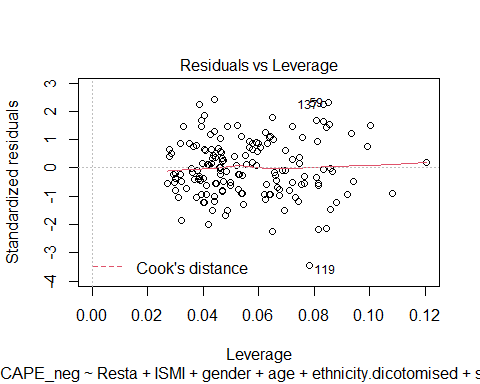


hist(new.df.cov3$standardized.residuals)


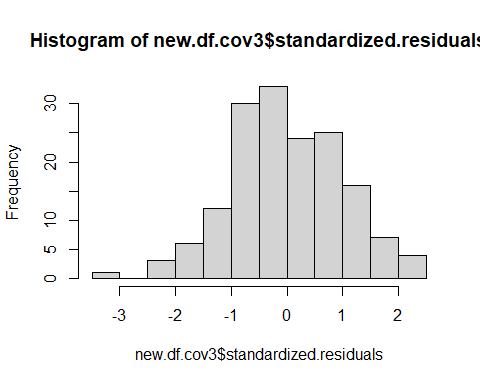


shapiro.test(new.df.cov3$standardized.residuals)

##
## Shapiro-Wilk normality test
##
## data: new.df.cov3$standardized.residuals
## W = 0.99199, p-value = 0.5088

new.df.cov3$standardized.residuals > 2 |new.df.cov3$standardized.residuals < -2

## 1 2 3 4 5 6 7 8 9 10 11 12 13
## FALSE TRUE FALSE FALSE FALSE FALSE FALSE FALSE FALSE FALSE FALSE FALSE FALSE
## 14 15 16 17 18 19 20 21 22 23 24 25 26
## FALSE FALSE FALSE FALSE FALSE FALSE FALSE FALSE FALSE FALSE FALSE FALSE FALSE
## 27 28 29 30 31 32 33 34 35 36 37 38 39
## FALSE TRUE FALSE FALSE FALSE FALSE FALSE FALSE FALSE FALSE FALSE FALSE FALSE
## 40 41 42 43 44 45 46 47 48 49 50 51 52
## FALSE FALSE FALSE FALSE FALSE FALSE FALSE FALSE FALSE FALSE FALSE FALSE FALSE
## 53 54 55 56 57 58 59 60 61 62 63 64 65
## FALSE FALSE FALSE FALSE FALSE FALSE TRUE FALSE FALSE FALSE FALSE FALSE FALSE
## 66 67 68 69 70 71 72 73 74 75 76 77 78
## FALSE FALSE FALSE FALSE FALSE FALSE FALSE FALSE FALSE FALSE FALSE FALSE FALSE
## 79 80 81 82 83 84 85 86 87 88 89 90 91
## FALSE FALSE FALSE FALSE FALSE FALSE FALSE FALSE FALSE FALSE FALSE FALSE FALSE
## 92 93 94 95 96 97 98 99 100 101 102 103 104
## FALSE FALSE FALSE FALSE FALSE FALSE FALSE FALSE FALSE FALSE FALSE FALSE FALSE
## 105 106 107 108 109 110 111 112 113 114 115 116 117
## FALSE FALSE FALSE FALSE FALSE FALSE FALSE FALSE FALSE FALSE FALSE FALSE FALSE
## 118 119 120 121 122 123 124 125 126 127 128 129 130
## FALSE TRUE FALSE FALSE FALSE FALSE FALSE FALSE TRUE FALSE FALSE FALSE FALSE
## 131 132 133 134 135 136 137 138 139 140 141 142 143
## FALSE FALSE TRUE FALSE FALSE FALSE TRUE FALSE FALSE FALSE FALSE FALSE FALSE
## 144 145 146 147 148 149 150 151 152 153 154 155 156
## TRUE FALSE FALSE FALSE FALSE FALSE FALSE FALSE FALSE FALSE FALSE FALSE FALSE
## 157 158 159 160 161
## FALSE FALSE FALSE FALSE FALSE

new.df.cov3$large.residual <- new.df.cov3$standardized.residuals > 2 | new.df.cov3$standardized.residuals < -2
sum(new.df.cov3$large.residual)

## [1] 8

new.df.cov3[new.df.cov3$large.residual, c("X1", "standardized.residuals")]

## # A tibble: 8 x 2
## X1 standardized.residuals
## <dbl> <dbl>
## 1 2 2.25
## 2 30 -2.17
## 3 64 2.32
## 4 130 -3.43
## 5 142 -2.24
## 6 150 -2.13
## 7 154 2.24
## 8 163 2.42

new.df.cov3$cooks.distance <- cooks.distance(model.y3.cov)
new.df.cov3$leverage <- hatvalues(model.y3.cov)
new.df.cov3$covariance <- covratio(model.y3.cov)

new.df.cov3[new.df.cov3$large.residual, c("X1", "cooks.distance", "leverage", "covariance" )]

## # A tibble: 8 x 4
## X1 cooks.distance leverage covariance
## <dbl> <dbl> <dbl> <dbl>
## 1 2 0.0227 0.0387 0.814
## 2 30 0.0467 0.0816 0.870
## 3 64 0.0556 0.0851 0.839
## 4 130 0.111 0.0783 0.557
## 5 142 0.0388 0.0651 0.839
## 6 150 0.0466 0.0843 0.881
## 7 154 0.0509 0.0834 0.855
## 8 163 0.0300 0.0442 0.781

Remove outlier and build models again

new.df.cov3 %>%
 filter (! (X1 == 130)) -> new.df.cov3


model.m3.cov <- lm(ISMI~ Resta + gender + age + ethnicity.dicotomised +
 sexuality.dicotomised + relationship.dicotomised +
 employment.dicotomised, data = new.df.cov3)
model.y3.cov <- lm(CAPE_neg ~ Resta + ISMI + gender + age + ethnicity.dicotomised +
 sexuality.dicotomised + relationship.dicotomised +
 employment.dicotomised, data = new.df.cov3)

Re-check assumptions

new.df.cov3$standardized.residuals <- rstandard(model.y3.cov)
plot(model.y3.cov)


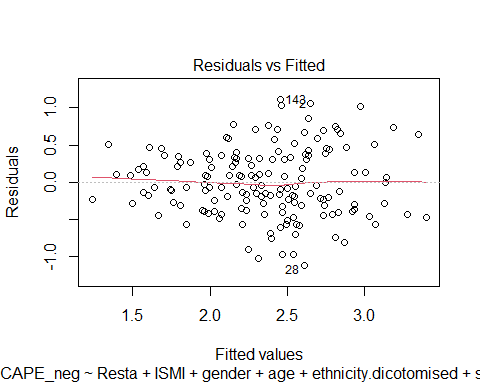

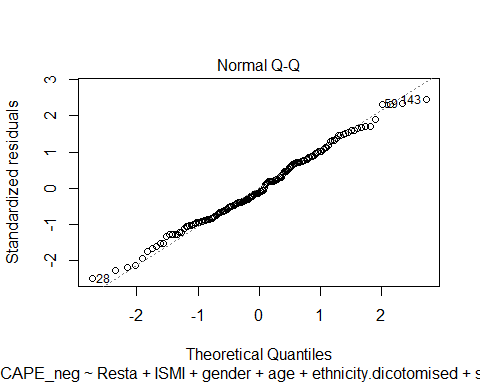

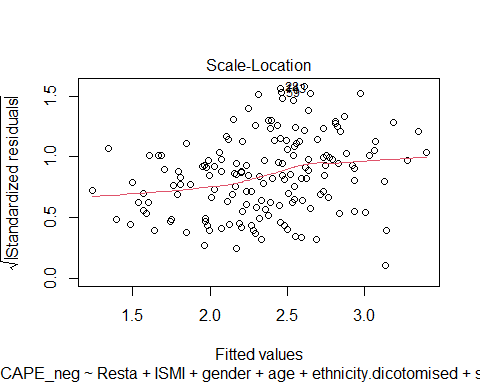

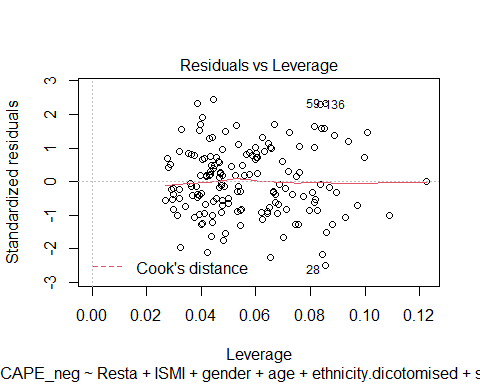


hist(new.df.cov3$standardized.residuals)


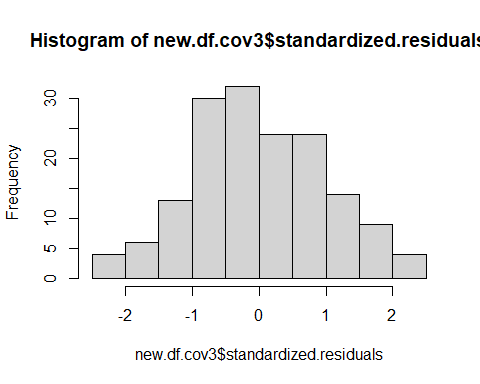


shapiro.test(new.df.cov3$standardized.residuals)

##
## Shapiro-Wilk normality test
##
## data: new.df.cov3$standardized.residuals
## W = 0.99166, p-value = 0.4775

Run mediation analysis

med.cov3 <- mediate(model.m3.cov, model.y3.cov, sims = 5000, treat = "Resta", mediator = "ISMI")

summary(med.cov3)

##
## Causal Mediation Analysis
##
## Quasi-Bayesian Confidence Intervals
##
## Estimate 95% CI Lower 95% CI Upper p-value
## ACME -0.0230 -0.0374 -0.01 <2e-16 ***
## ADE -0.0138 -0.0330 0.01 0.1524
## Total Effect -0.0367 -0.0584 -0.02 0.0004 ***
## Prop. Mediated 0.6233 0.3152 1.28 0.0004 ***
## ---
## Signif. codes: 0 '***' 0.001 '**' 0.01 '*' 0.05 '.' 0.1 ' ' 1
##
## Sample Size Used: 160
##
##
## Simulations: 5000

## CAPE DEPRESSIVE

Test mediation model : Resta -> internalised stigma -> CAPE depressive

Create data frame

new.df4 <- data.frame(df$Resta.total, df$ISMI_total, df$CAPE_depressive)

#remove any rows with NAs
new.df4%>%
 filter(! is.na(df.ISMI_total) & ! (df.ISMI_total == "Inf")) -> new.df4

Build models

model.m4 <- lm(df.ISMI_total ~ df.Resta.total, data = new.df4)
model.y4 <- lm(df.CAPE_depressive ~ df.Resta.total + df.ISMI_total, data = new.df4)

Check parametric assumptions and for outliers

new.df4$standardized.residuals <- rstandard(model.y4)
plot(model.y4)


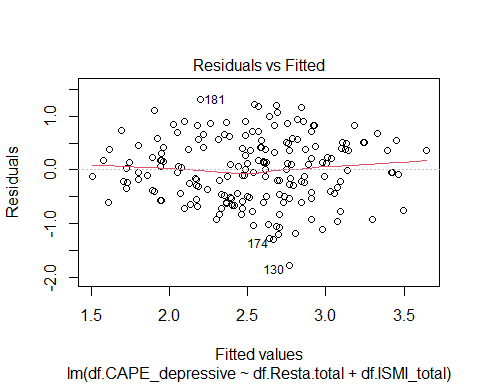

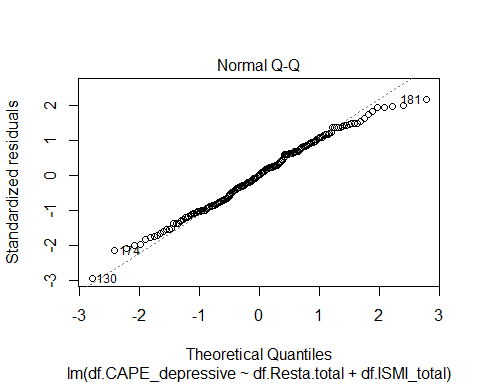

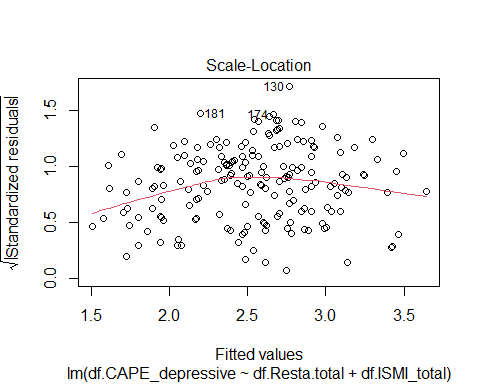

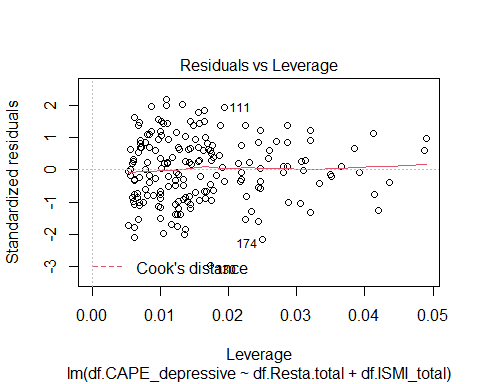


hist(new.df4$standardized.residuals)


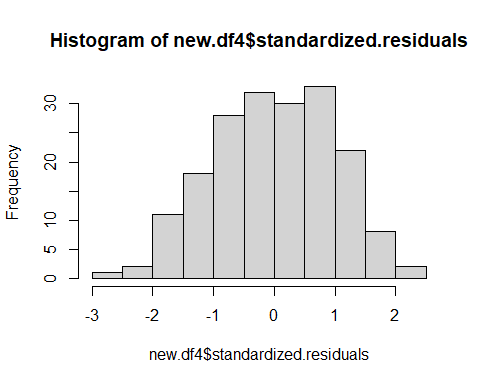


shapiro.test(new.df4$standardized.residuals)

##
## Shapiro-Wilk normality test
##
## data: new.df4$standardized.residuals
## W = 0.99116, p-value = 0.3083

new.df4$standardized.residuals > 2 | new.df4$standardized.residuals < -2

## [1] FALSE FALSE FALSE FALSE FALSE FALSE FALSE FALSE FALSE FALSE FALSE FALSE
## [13] FALSE FALSE FALSE FALSE FALSE FALSE FALSE FALSE FALSE FALSE FALSE FALSE
## [25] FALSE FALSE FALSE FALSE FALSE FALSE FALSE FALSE FALSE FALSE FALSE FALSE
## [37] FALSE FALSE FALSE FALSE FALSE FALSE FALSE FALSE FALSE FALSE FALSE FALSE
## [49] FALSE FALSE FALSE FALSE FALSE FALSE FALSE FALSE FALSE FALSE FALSE FALSE
## [61] FALSE FALSE FALSE FALSE FALSE FALSE FALSE FALSE FALSE FALSE FALSE FALSE
## [73] FALSE FALSE FALSE FALSE FALSE FALSE FALSE FALSE FALSE FALSE FALSE FALSE
## [85] FALSE FALSE FALSE FALSE FALSE FALSE FALSE FALSE FALSE FALSE FALSE FALSE
## [97] FALSE FALSE FALSE FALSE FALSE FALSE FALSE FALSE FALSE FALSE FALSE FALSE
## [109] FALSE FALSE FALSE FALSE FALSE FALSE FALSE FALSE FALSE FALSE FALSE FALSE
## [121] FALSE FALSE FALSE FALSE FALSE FALSE FALSE FALSE FALSE TRUE FALSE FALSE
## [133] FALSE FALSE FALSE FALSE FALSE TRUE FALSE FALSE FALSE FALSE FALSE FALSE
## [145] FALSE FALSE FALSE FALSE FALSE FALSE FALSE FALSE FALSE FALSE FALSE FALSE
## [157] FALSE FALSE FALSE FALSE FALSE FALSE FALSE FALSE FALSE FALSE FALSE FALSE
## [169] FALSE FALSE FALSE FALSE FALSE TRUE FALSE FALSE FALSE FALSE FALSE FALSE
## [181] TRUE TRUE FALSE FALSE FALSE FALSE FALSE

new.df4$large.residual <- new.df4$standardized.residuals > 2 | new.df4$standardized.residuals < -2
sum(new.df4$large.residual)

## [1] 5

new.df4[new.df4$large.residual, c( "standardized.residuals")]

## [1] -2.944949 -2.097786 -2.155862 2.169864 2.008155

new.df4$cooks.distance <- cooks.distance(model.y4)
new.df4$leverage <- hatvalues(model.y4)
new.df4$covariance <- covratio(model.y4)

new.df4[new.df4$large.residual, c("cooks.distance", "leverage", "covariance" )]

## cooks.distance leverage covariance
## 130 0.051036828 0.017347993 0.8949432
## 138 0.009137721 0.006190701 0.9511687
## 174 0.039747976 0.025014546 0.9655375
## 181 0.017214577 0.010849631 0.9507469
## 182 0.018081573 0.013272726 0.9638967

No outliers to remove, run analysis

med.with4 <- mediate(model.m4 , model.y4, sims = 1000,
 treat = "df.Resta.total",
 mediator = "df.ISMI_total")
summary(med.with4)

##
## Causal Mediation Analysis
##
## Quasi-Bayesian Confidence Intervals
##
## Estimate 95% CI Lower 95% CI Upper p-value
## ACME -0.0189 -0.0332 -0.01 0.002 **
## ADE -0.0141 -0.0319 0.01 0.158
## Total Effect -0.0329 -0.0550 -0.01 0.006 **
## Prop. Mediated 0.5705 0.2192 1.33 0.008 **
## ---
## Signif. codes: 0 '***' 0.001 '**' 0.01 '*' 0.05 '.' 0.1 ' ' 1
##
## Sample Size Used: 187
##
##
## Simulations: 1000

Add in co-variates

#Create data frame
new.df.cov4 <- read_csv("Z:/Online study IRAS ID 271957/Online analysis/new.df.cov_2022.csv")

## Warning: Missing column names filled in: 'X1' [1]

##
## -- Column specification --------------------------------------------------------
## cols(
## X1 = col_double(),
## Resta = col_double(),
## Loneliness = col_double(),
## gender = col_character(),
## age = col_double(),
## ethnicity = col_character(),
## sexuality = col_character(),
## relationship.status = col_character(),
## SWEMWBS = col_double(),
## employment = col_character(),
## ethnicity.dicotomised = col_character(),
## sexuality.dicotomised = col_character(),
## relationship.dicotomised = col_character(),
## employment.dicotomised = col_character()
## )

#add in cape depressive subscale scores and internalised stigma scores
new.df.cov4$CAPE_dep <- df$CAPE_depressive
new.df.cov4$ISMI <- df$ISMI_total

#remove column loneliness
new.df.cov4 <- new.df.cov4[, -c(3)]

# remove NAs and 'Inf' rows from ISMI
new.df.cov4%>%
 filter(! is.na(ISMI) & ! (ISMI == "Inf")) -> new.df.cov4

#remove rows with missing data to allow for mediation package to work
new.df.cov4 <- na.omit(new.df.cov4)

Add covariates into models

model.m4.cov <- lm(ISMI ~ Resta + gender + age + ethnicity.dicotomised +
 sexuality.dicotomised + relationship.dicotomised +
 employment.dicotomised, data = new.df.cov4)
model.y4.cov <- lm(CAPE_dep ~ Resta + ISMI + gender + age + ethnicity.dicotomised +
 sexuality.dicotomised + relationship.dicotomised +
 employment.dicotomised, data = new.df.cov4)

Check assumptions and for outliers

new.df.cov4$standardized.residuals <- rstandard(model.y4.cov)
plot(model.y4.cov)


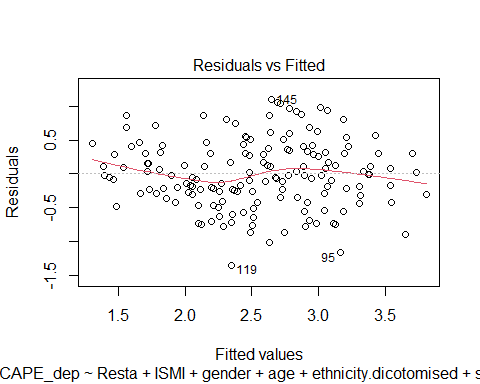

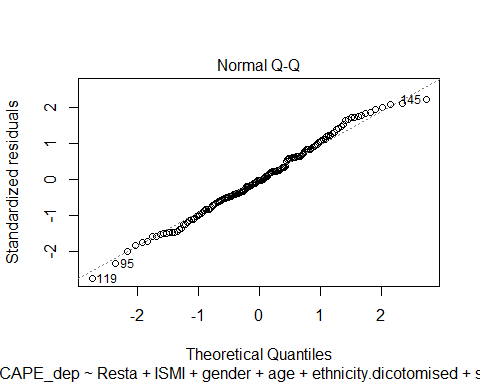

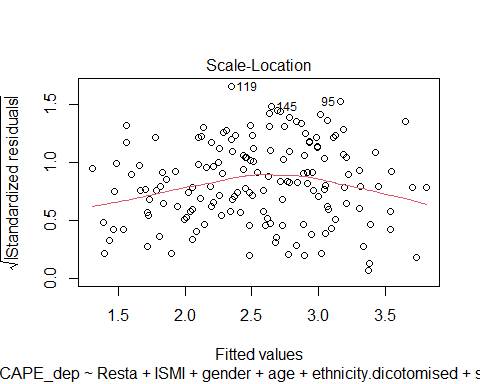

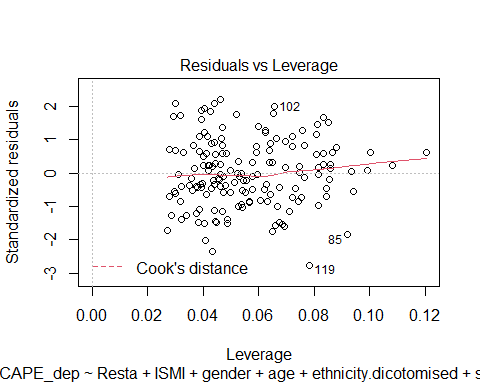


hist(new.df.cov4$standardized.residuals)


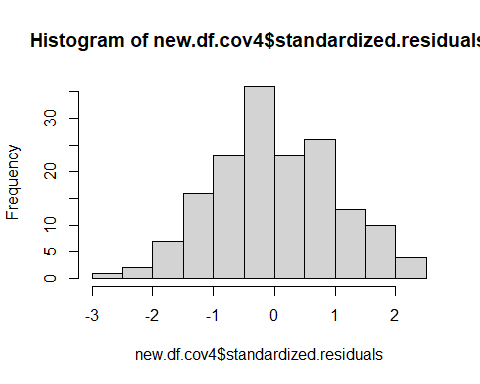


shapiro.test(new.df.cov4$standardized.residuals)

##
## Shapiro-Wilk normality test
##
## data: new.df.cov4$standardized.residuals
## W = 0.99252, p-value = 0.5705

new.df.cov4$standardized.residuals > 2 |new.df.cov4$standardized.residuals < -2

## 1 2 3 4 5 6 7 8 9 10 11 12 13
## FALSE FALSE FALSE TRUE FALSE FALSE FALSE FALSE FALSE FALSE FALSE FALSE FALSE
## 14 15 16 17 18 19 20 21 22 23 24 25 26
## FALSE FALSE FALSE FALSE FALSE FALSE FALSE FALSE FALSE FALSE FALSE FALSE FALSE
## 27 28 29 30 31 32 33 34 35 36 37 38 39
## FALSE FALSE FALSE FALSE FALSE FALSE FALSE FALSE FALSE FALSE FALSE FALSE FALSE
## 40 41 42 43 44 45 46 47 48 49 50 51 52
## FALSE FALSE FALSE FALSE FALSE FALSE FALSE FALSE FALSE FALSE FALSE FALSE FALSE
## 53 54 55 56 57 58 59 60 61 62 63 64 65
## FALSE FALSE FALSE FALSE FALSE FALSE FALSE FALSE FALSE FALSE FALSE FALSE FALSE
## 66 67 68 69 70 71 72 73 74 75 76 77 78
## FALSE FALSE FALSE FALSE FALSE FALSE FALSE FALSE FALSE FALSE FALSE FALSE FALSE
## 79 80 81 82 83 84 85 86 87 88 89 90 91
## FALSE FALSE FALSE FALSE FALSE FALSE FALSE FALSE FALSE FALSE FALSE FALSE FALSE
## 92 93 94 95 96 97 98 99 100 101 102 103 104
## TRUE FALSE FALSE TRUE FALSE FALSE FALSE FALSE FALSE FALSE TRUE FALSE FALSE
## 105 106 107 108 109 110 111 112 113 114 115 116 117
## FALSE FALSE FALSE FALSE FALSE FALSE FALSE FALSE FALSE FALSE FALSE FALSE FALSE
## 118 119 120 121 122 123 124 125 126 127 128 129 130
## FALSE TRUE FALSE FALSE FALSE FALSE FALSE FALSE FALSE FALSE FALSE FALSE FALSE
## 131 132 133 134 135 136 137 138 139 140 141 142 143
## FALSE FALSE FALSE FALSE FALSE FALSE FALSE FALSE FALSE FALSE FALSE FALSE FALSE
## 144 145 146 147 148 149 150 151 152 153 154 155 156
## TRUE TRUE FALSE FALSE FALSE FALSE FALSE FALSE FALSE FALSE FALSE FALSE FALSE
## 157 158 159 160 161
## FALSE FALSE FALSE FALSE FALSE

new.df.cov4$large.residual <- new.df.cov4$standardized.residuals > 2 | new.df.cov4$standardized.residuals < -2
sum(new.df.cov4$large.residual)

## [1] 7

new.df.cov4[new.df.cov4$large.residual, c("X1", "standardized.residuals")]

## # A tibble: 7 x 2
## X1 standardized.residuals
## <dbl> <dbl>
## 1 4 2.10
## 2 101 -2.02
## 3 104 -2.34
## 4 111 2.01
## 5 130 -2.76
## 6 163 2.08
## 7 164 2.21

new.df.cov4$cooks.distance <- cooks.distance(model.y4.cov)
new.df.cov4$leverage <- hatvalues(model.y4.cov)
new.df.cov4$covariance <- covratio(model.y4.cov)

new.df.cov4[new.df.cov4$large.residual, c("X1", "cooks.distance", "leverage", "covariance" )]

## # A tibble: 7 x 4
## X1 cooks.distance leverage covariance
## <dbl> <dbl> <dbl> <dbl>
## 1 4 0.0151 0.0299 0.839
## 2 101 0.0194 0.0410 0.866
## 3 104 0.0276 0.0435 0.798
## 4 111 0.0314 0.0656 0.892
## 5 130 0.0717 0.0783 0.726
## 6 163 0.0223 0.0442 0.856
## 7 164 0.0263 0.0462 0.829

No outliers, run mediation analysis

med.cov4 <- mediate(model.m4.cov, model.y4.cov, sims = 5000, treat = "Resta", mediator = "ISMI")

summary(med.cov4)

##
## Causal Mediation Analysis
##
## Quasi-Bayesian Confidence Intervals
##
## Estimate 95% CI Lower 95% CI Upper p-value
## ACME -0.0285 -0.0460 -0.01 4e-04 ***
## ADE -0.0421 -0.0627 -0.02 <2e-16 ***
## Total Effect -0.0706 -0.0955 -0.05 <2e-16 ***
## Prop. Mediated 0.4008 0.2212 0.62 4e-04 ***
## ---
## Signif. codes: 0 '***' 0.001 '**' 0.01 '*' 0.05 '.' 0.1 ' ' 1
##
## Sample Size Used: 161
##
##
## Simulations: 5000
