# Supplementary material for: Being more satisfied with romantic relationship status is associated with increased mental wellbeing in people with experience of psychosis
Source: Front Psychiatry. 2023 Sep 28;14:1232973. doi: 10.3389/fpsyt.2023.1232973 (PMC10569177; doi:10.3389/fpsyt.2023.1232973)
Supplement: Supplementary file 9 [file Data_Sheet_9.DOCX]

Mediation analysis (Loneliness)

Rebecca White

11/07/2022

Install packages and dataset

library(readr)
library(mediation)

## Loading required package: MASS

## Loading required package: Matrix

## Loading required package: mvtnorm

## Loading required package: sandwich

## mediation: Causal Mediation Analysis
## Version: 4.5.0

library(tidyverse)

## -- Attaching packages --------------------------------------- tidyverse 1.3.0 --

## v ggplot2 3.3.2 v dplyr 1.0.2
## v tibble 3.0.4 v stringr 1.4.0
## v tidyr 1.1.2 v forcats 0.5.0
## v purrr 0.3.4

## -- Conflicts ------------------------------------------ tidyverse_conflicts() --
## x tidyr::expand() masks Matrix::expand()
## x dplyr::filter() masks stats::filter()
## x dplyr::lag() masks stats::lag()
## x tidyr::pack() masks Matrix::pack()
## x dplyr::select() masks MASS::select()
## x tidyr::unpack() masks Matrix::unpack()

df <- read_csv("Dataset_190_obs_2.9.21.csv")

## Warning: Missing column names filled in: 'X1' [1]

##
## -- Column specification --------------------------------------------------------
## cols(
## .default = col_double(),
## redcap_survey_identifier = col_logical(),
## pis_timestamp = col_datetime(format = ""),
## screening_questions_timestamp = col_datetime(format = ""),
## demographic_information_timestamp = col_datetime(format = ""),
## nationality = col_character(),
## ethnicity_other = col_character(),
## gender_self_describe = col_character(),
## sexual_orientation_selfdescribe = col_character(),
## rr_selfdescribe = col_character(),
## last_rr_end = col_character(),
## current_rr_length = col_character(),
## the_community_assessment_of_psychic_experiences_ca_timestamp = col_datetime(format = ""),
## the_short_warwick_mental_health_wellbeing_scale_timestamp = col_datetime(format = ""),
## adapted_satisfaction_with_relationships_scale_rest_timestamp = col_datetime(format = ""),
## three_item_loneliness_scale_timestamp = col_datetime(format = ""),
## internalised_stigma_of_mental_illness_inventory_10_timestamp = col_datetime(format = ""),
## multidimensional_scale_of_perceived_social_support_timestamp = col_datetime(format = ""),
## self_esteem_rating_scale_short_form_serssf_timestamp = col_datetime(format = ""),
## relationships_questionnaire_timestamp = col_datetime(format = ""),
## Screening_Qs_result = col_character()
## # ... with 7 more columns
## )
## i Use `spec()` for the full column specifications.

## SWEMWBS

test mediation model : resta -> loneliness -> SWEMWBS

Create dataframe to work from

new.df <- data.frame(df$X1, df$Resta.total, df$LonelinessTotal, df$SWEMWBS_metric)

#remove any rows with NAs
new.df %>%
 filter(! is.na(df.LonelinessTotal)) -> new.df

Create model without covariates first

model.m <- lm(df.LonelinessTotal ~ df.Resta.total, data = new.df)
model.y <- lm(df.SWEMWBS_metric ~ df.Resta.total + df.LonelinessTotal, data = new.df )

Check parametric assumptions

new.df$standardized.residuals <- rstandard(model.y)
plot(model.y)


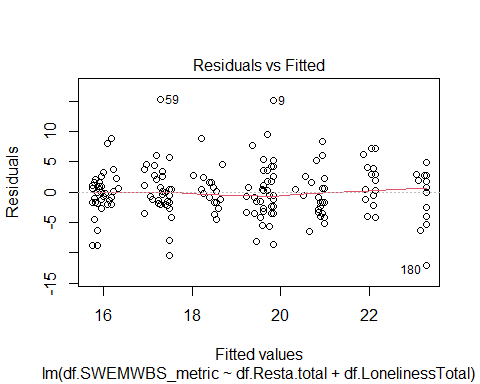

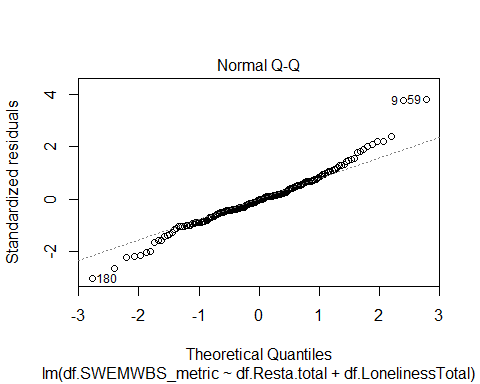

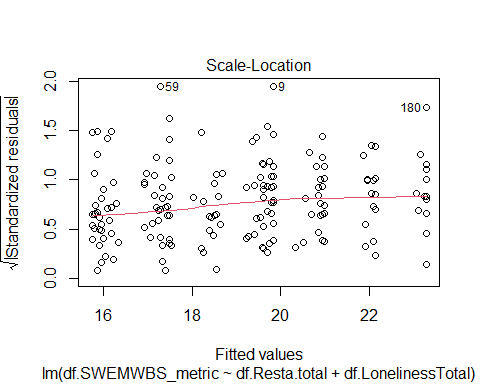

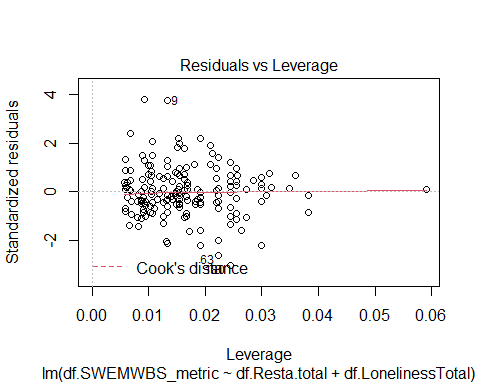


hist(new.df$standardized.residuals)


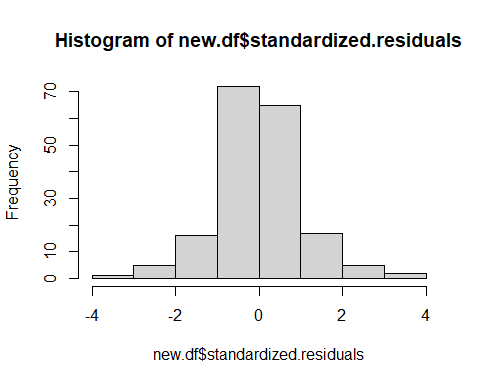


shapiro.test(new.df$standardized.residuals)

##
## Shapiro-Wilk normality test
##
## data: new.df$standardized.residuals
## W = 0.97051, p-value = 0.0006483

new.df$standardized.residuals > 2 | new.df$standardized.residuals < -2

## [1] FALSE FALSE FALSE FALSE FALSE FALSE FALSE FALSE TRUE FALSE FALSE FALSE
## [13] FALSE FALSE FALSE FALSE FALSE FALSE FALSE FALSE FALSE FALSE FALSE FALSE
## [25] TRUE FALSE FALSE FALSE FALSE FALSE FALSE FALSE FALSE FALSE FALSE FALSE
## [37] FALSE FALSE FALSE FALSE FALSE FALSE FALSE FALSE FALSE FALSE TRUE FALSE
## [49] FALSE FALSE FALSE FALSE FALSE FALSE FALSE FALSE FALSE FALSE TRUE FALSE
## [61] FALSE FALSE TRUE FALSE FALSE FALSE FALSE FALSE FALSE FALSE FALSE FALSE
## [73] FALSE FALSE FALSE FALSE FALSE FALSE FALSE FALSE FALSE FALSE FALSE FALSE
## [85] FALSE FALSE FALSE FALSE FALSE FALSE FALSE FALSE FALSE FALSE FALSE FALSE
## [97] FALSE FALSE FALSE FALSE FALSE FALSE FALSE FALSE TRUE FALSE FALSE FALSE
## [109] FALSE FALSE FALSE FALSE FALSE FALSE FALSE FALSE FALSE FALSE FALSE FALSE
## [121] FALSE FALSE FALSE FALSE FALSE FALSE FALSE FALSE TRUE FALSE FALSE FALSE
## [133] TRUE FALSE FALSE FALSE FALSE TRUE FALSE FALSE FALSE FALSE FALSE FALSE
## [145] FALSE FALSE FALSE FALSE FALSE FALSE FALSE FALSE FALSE FALSE FALSE FALSE
## [157] FALSE TRUE FALSE FALSE FALSE FALSE FALSE TRUE FALSE FALSE FALSE FALSE
## [169] FALSE TRUE FALSE FALSE FALSE FALSE FALSE FALSE FALSE FALSE FALSE TRUE
## [181] FALSE FALSE FALSE

new.df$large.residual <- new.df$standardized.residuals > 2 | new.df$standardized.residuals < -2
sum(new.df$large.residual)

## [1] 13

new.df[new.df$large.residual, c("df.X1", "standardized.residuals")]

## df.X1 standardized.residuals
## 9 9 3.772312
## 25 25 2.196830
## 47 48 -2.039049
## 59 60 3.792625
## 63 64 -2.621982
## 105 107 -2.211418
## 129 131 -2.191093
## 133 137 2.211763
## 138 142 2.078466
## 158 163 -2.136130
## 164 169 2.381237
## 170 175 2.000159
## 180 185 -3.012578

new.df$cooks.distance <- cooks.distance(model.y)
new.df$leverage <- hatvalues(model.y)
new.df$covariance <- covratio(model.y)

new.df[new.df$large.residual, c("cooks.distance", "leverage", "covariance" )]

## cooks.distance leverage covariance
## 9 0.06362751 0.013236229 0.8049018
## 25 0.02506305 0.015340811 0.9518389
## 47 0.01838788 0.013094029 0.9605833
## 59 0.04463364 0.009223158 0.7994143
## 63 0.05253901 0.022412957 0.9254787
## 105 0.03173906 0.019098499 0.9544334
## 129 0.04930187 0.029887202 0.9665278
## 133 0.03171045 0.019075773 0.9543863
## 138 0.01563077 0.010738095 0.9556447
## 158 0.02040258 0.013236229 0.9540935
## 164 0.01279016 0.006721451 0.9300024
## 170 0.02098064 0.015489279 0.9655032
## 180 0.07568694 0.024408085 0.8924516

Run analysis with outliers then remove outliers and re-run analysis

med.with <- mediate(model.m, model.y, sims = 1000, boot = TRUE, treat = "df.Resta.total",
 mediator = "df.LonelinessTotal")

## Running nonparametric bootstrap

summary(med.with)

##
## Causal Mediation Analysis
##
## Nonparametric Bootstrap Confidence Intervals with the Percentile Method
##
## Estimate 95% CI Lower 95% CI Upper p-value
## ACME 0.2558 0.1665 0.36 <2e-16 ***
## ADE 0.0433 -0.0967 0.19 0.55
## Total Effect 0.2991 0.1608 0.44 <2e-16 ***
## Prop. Mediated 0.8553 0.5232 1.57 <2e-16 ***
## ---
## Signif. codes: 0 '***' 0.001 '**' 0.01 '*' 0.05 '.' 0.1 ' ' 1
##
## Sample Size Used: 183
##
##
## Simulations: 1000

new.df.OR <- new.df

new.df.OR %>%
 filter(! (df.X1 == 9)) %>%
 filter(! (df.X1 == 59)) %>%
 filter(! (df.X1 == 180)) -> new.df.OR

model.m.OR <- lm(df.LonelinessTotal ~ df.Resta.total, data = new.df.OR)
model.y.OR <- lm(df.SWEMWBS_metric ~ df.Resta.total + df.LonelinessTotal, data = new.df.OR )

Re-check parametric assumptions

new.df.OR$standardized.residuals <- rstandard(model.y.OR)
plot(model.y.OR)


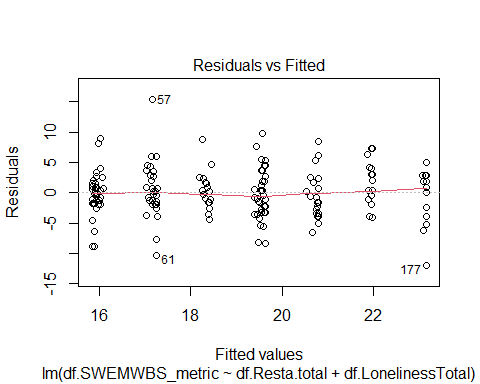

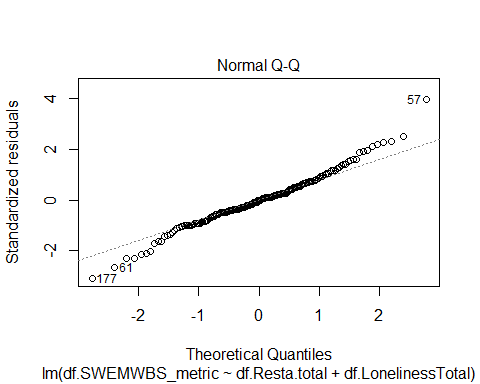

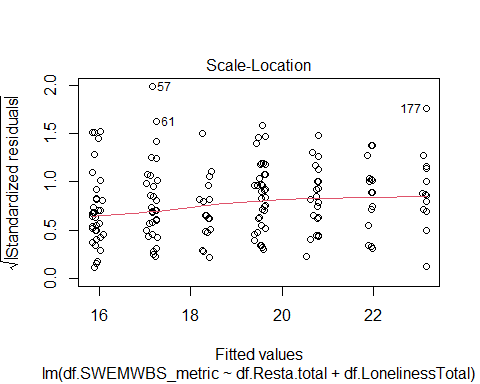

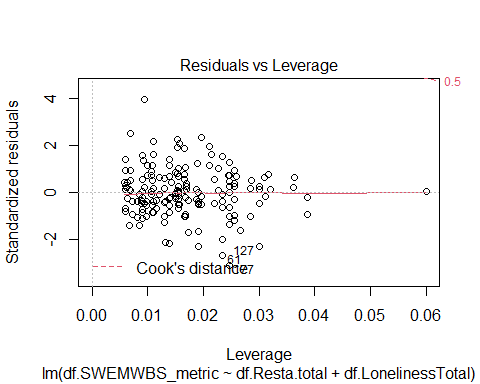


hist(new.df.OR$standardized.residuals)


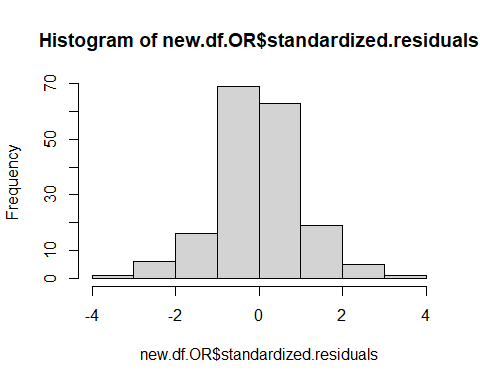


shapiro.test(new.df.OR$standardized.residuals)

##
## Shapiro-Wilk normality test
##
## data: new.df.OR$standardized.residuals
## W = 0.98135, p-value = 0.01648

Re-run analysis

med.without<- mediate(model.m.OR , model.y.OR, sims = 1000, treat = "df.Resta.total", mediator = "df.LonelinessTotal")

summary(med.without)

##
## Causal Mediation Analysis
##
## Quasi-Bayesian Confidence Intervals
##
## Estimate 95% CI Lower 95% CI Upper p-value
## ACME 0.2653 0.1716 0.37 <2e-16 ***
## ADE 0.0174 -0.1215 0.16 0.82
## Total Effect 0.2827 0.1471 0.42 <2e-16 ***
## Prop. Mediated 0.9359 0.5744 1.78 <2e-16 ***
## ---
## Signif. codes: 0 '***' 0.001 '**' 0.01 '*' 0.05 '.' 0.1 ' ' 1
##
## Sample Size Used: 180
##
##
## Simulations: 1000

Add in covariates : gender, age, ethnicity, sexuality, relationship status, employment

Create new data frame to work from

Dataset_Missing_Removed <- df

new.df.cov <- data.frame(Dataset_Missing_Removed$Resta.total,
 Dataset_Missing_Removed$LonelinessTotal,
 Dataset_Missing_Removed$GenderF,
 Dataset_Missing_Removed$age,
 Dataset_Missing_Removed$EthnicityF,
 Dataset_Missing_Removed$SexualityF,
 Dataset_Missing_Removed$R_Status_simplified,
 Dataset_Missing_Removed$rr_selfdescribe,
 Dataset_Missing_Removed$SWEMWBS_metric,
 Dataset_Missing_Removed$EmploymentF,
 Dataset_Missing_Removed$ethnicity_other)


#rename columns
names(new.df.cov)[names(new.df.cov) == "Dataset_Missing_Removed.SWEMWBS_metric"]<- "SWEMWBS"
names(new.df.cov)[names(new.df.cov) == "Dataset_Missing_Removed.LonelinessTotal"]<- "Loneliness"
names(new.df.cov)[names(new.df.cov) == "Dataset_Missing_Removed.Resta.total"]<- "Resta"
names(new.df.cov)[names(new.df.cov) == "Dataset_Missing_Removed.GenderF"]<- "gender"
names(new.df.cov)[names(new.df.cov) == "Dataset_Missing_Removed.age"]<- "age"
names(new.df.cov)[names(new.df.cov) == "Dataset_Missing_Removed.EthnicityF"]<- "ethnicity"
names(new.df.cov)[names(new.df.cov) == "Dataset_Missing_Removed.SexualityF"]<- "sexuality"
names(new.df.cov)[names(new.df.cov) == "Dataset_Missing_Removed.R_Status_simplified"]<- "relationship.status"
names(new.df.cov)[names(new.df.cov) == "Dataset_Missing_Removed.EmploymentF"]<- "employment"

#create dichotomous variables
#GENDER
#Gender is categorised as 1= female, 2 = male, 3 = prefer not to say, 4 = prefer to self describe
table(new.df.cov$gender)

##
## female male Self_describe
## 105 69 6

#remove 'prefer not to say & self describe

new.df.cov$gender <- factor(new.df.cov$gender,
 c("female","male"), c("female", "male"))
table(new.df.cov$gender)

##
## female male
## 105 69

#ETHNICITY
table(new.df.cov$ethnicity)

##
## asian black mixed other white
## 10 11 5 5 148

new.df.cov %>%
 mutate(ethnicity.dicotomised = case_when(ethnicity == "other" & Dataset_Missing_Removed.ethnicity_other == "White Scottish" ~ "white",
 ethnicity == "white" ~ "white",
 ethnicity == "mixed" ~ "PGM",
 ethnicity == "asian" ~ "PGM",
 ethnicity == "black" ~ "PGM",
 ethnicity == "chinese" ~ "PGM",
 ethnicity == "other" ~ "PGM")) -> new.df.cov

table(new.df.cov$ethnicity.dicotomised)

##
## PGM white
## 29 150

#SEXUALITY
table(new.df.cov$sexuality)

##
## bisexual gay/lesbian heterosexual prefer not to say
## 24 9 132 6
## self-describe
## 9

new.df.cov %>%
 mutate(sexuality.dicotomised = case_when(sexuality == "heterosexual" ~ "heterosexual",
 sexuality == "bisexual" ~ "LGBQ+",
 sexuality == "gay/lesbian" ~ "LGBQ+",
 sexuality == "self-describe" ~ "LGBQ+")) -> new.df.cov
table(new.df.cov$sexuality.dicotomised)

##
## heterosexual LGBQ+
## 132 42

#RELATIONSHIP STATUS
table(new.df.cov$relationship.status)

##
## dating partner self describe separated single
## 5 78 5 2 89

view(Dataset_Missing_Removed$rr_selfdescribe)

new.df.cov%>%
 mutate(relationship.dicotomised = case_when(relationship.status == "single" ~ "single",
 relationship.status == "dating" ~ "single",
 relationship.status == "separated" ~ "single",
 relationship.status == "widowed" ~ "single",
 relationship.status == "partner" ~ "partner",
 relationship.status == "self describe" & Dataset_Missing_Removed.rr_selfdescribe == "Living with a wife, queerplatonic partner, and steady, with one long-distance relationship as well" ~ "partner",
 relationship.status == "self describe" ~ "single")) -> new.df.cov

table(new.df.cov$relationship.dicotomised)

##
## partner single
## 79 100

#EMPLOYMENT
table(new.df.cov$employment)

##
## employee FT education
## 56 21
## looking after home/family rec. sickness/disability benefits
## 6 58
## retired self-employed
## 5 5
## unemployed
## 26

new.df.cov %>%
 mutate(employment.dicotomised = case_when ( employment == "employee" ~ "working/FT.education",
 employment == "self-employed" ~ "working/FT.education",
 employment == "FT education" ~ "working/FT.education",
 employment == "unemployed" ~ "unemployed",
 employment == "looking after home/family" ~ "working/FT.education",
 employment == "rec. sickness/disability benefits" ~ "unemployed",
 employment == "retired" ~ "unemployed")) -> new.df.cov

table(new.df.cov$employment.dicotomised)

##
## unemployed working/FT.education
## 89 88

## remove missing data
#remove columns that are mainly NA e.g. rr_self describe so they don't interfere with next stages
drops <- c("Dataset_Missing_Removed.rr_selfdescribe", "Dataset_Missing_Removed.ethnicity_other")
new.df.cov[ , !(names(new.df.cov) %in% drops)] -> new.df.cov

## save this table so don't have to build it all again later!
write.csv(new.df.cov, file = "Z:/Online study IRAS ID 271957/Online analysis/new.df.cov_2022.csv", row.names = TRUE)

Use new data frame to build model with covariates

new.df.cov <- read_csv("new.df.cov_2022.csv")

## Warning: Missing column names filled in: 'X1' [1]

##
## -- Column specification --------------------------------------------------------
## cols(
## X1 = col_double(),
## Resta = col_double(),
## Loneliness = col_double(),
## gender = col_character(),
## age = col_double(),
## ethnicity = col_character(),
## sexuality = col_character(),
## relationship.status = col_character(),
## SWEMWBS = col_double(),
## employment = col_character(),
## ethnicity.dicotomised = col_character(),
## sexuality.dicotomised = col_character(),
## relationship.dicotomised = col_character(),
## employment.dicotomised = col_character()
## )

#remove rows with missing data to allow for mediation package to work
new.df.cov <- na.omit(new.df.cov)

Add covariates into models

model.m.cov <- lm(Loneliness ~ Resta + gender + age + ethnicity.dicotomised +
 sexuality.dicotomised + relationship.dicotomised +
 employment.dicotomised, data = new.df.cov)

model.y.cov <- lm(SWEMWBS ~ Resta + Loneliness+ gender + age + ethnicity.dicotomised + sexuality.dicotomised + relationship.dicotomised +
 employment.dicotomised, data = new.df.cov)

Check assumptions

new.df.cov$standardized.residuals <- rstandard(model.y.cov)
plot(model.y.cov)


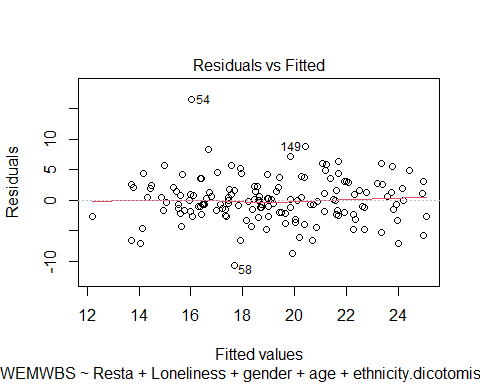

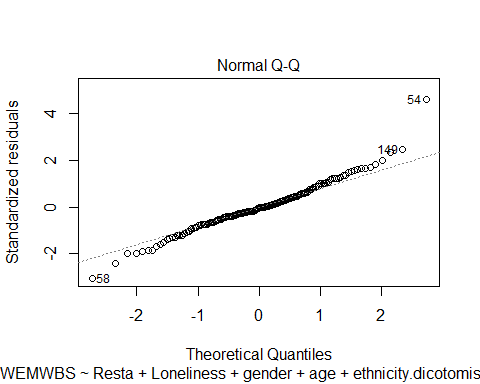

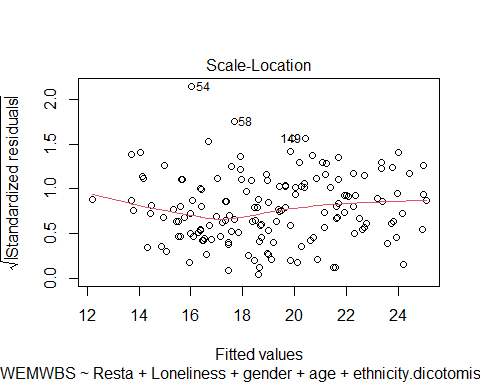

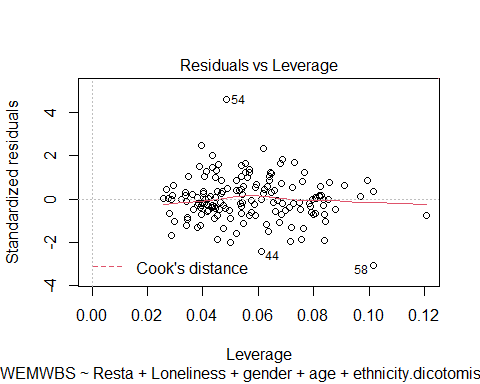


hist(new.df.cov$standardized.residuals)


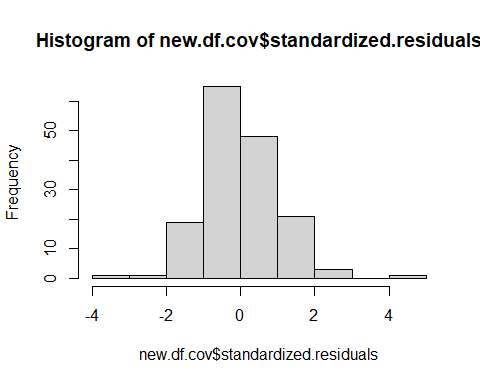


shapiro.test(new.df.cov$standardized.residuals)

##
## Shapiro-Wilk normality test
##
## data: new.df.cov$standardized.residuals
## W = 0.97131, p-value = 0.002154

Check for outliers

new.df.cov$standardized.residuals > 2 | new.df.cov$standardized.residuals < -2

## 1 2 3 4 5 6 7 8 9 10 11 12 13
## FALSE FALSE FALSE FALSE FALSE FALSE FALSE FALSE FALSE FALSE FALSE FALSE FALSE
## 14 15 16 17 18 19 20 21 22 23 24 25 26
## FALSE FALSE FALSE FALSE FALSE FALSE FALSE FALSE FALSE TRUE FALSE FALSE FALSE
## 27 28 29 30 31 32 33 34 35 36 37 38 39
## FALSE FALSE FALSE FALSE FALSE FALSE FALSE FALSE FALSE FALSE FALSE FALSE FALSE
## 40 41 42 43 44 45 46 47 48 49 50 51 52
## FALSE FALSE FALSE FALSE TRUE FALSE FALSE FALSE FALSE FALSE FALSE FALSE FALSE
## 53 54 55 56 57 58 59 60 61 62 63 64 65
## FALSE TRUE FALSE FALSE FALSE TRUE FALSE FALSE FALSE FALSE FALSE FALSE FALSE
## 66 67 68 69 70 71 72 73 74 75 76 77 78
## FALSE FALSE FALSE FALSE FALSE FALSE FALSE FALSE FALSE FALSE FALSE FALSE FALSE
## 79 80 81 82 83 84 85 86 87 88 89 90 91
## FALSE FALSE FALSE FALSE FALSE FALSE FALSE FALSE FALSE FALSE FALSE FALSE FALSE
## 92 93 94 95 96 97 98 99 100 101 102 103 104
## FALSE FALSE FALSE FALSE FALSE FALSE FALSE FALSE FALSE FALSE FALSE FALSE FALSE
## 105 106 107 108 109 110 111 112 113 114 115 116 117
## FALSE FALSE FALSE FALSE FALSE FALSE FALSE FALSE FALSE FALSE FALSE FALSE FALSE
## 118 119 120 121 122 123 124 125 126 127 128 129 130
## FALSE FALSE FALSE FALSE FALSE TRUE FALSE FALSE FALSE FALSE FALSE FALSE FALSE
## 131 132 133 134 135 136 137 138 139 140 141 142 143
## FALSE FALSE FALSE FALSE FALSE FALSE FALSE FALSE FALSE FALSE FALSE FALSE FALSE
## 144 145 146 147 148 149 150 151 152 153 154 155 156
## FALSE FALSE FALSE FALSE FALSE TRUE FALSE FALSE FALSE FALSE FALSE FALSE FALSE
## 157 158 159
## FALSE FALSE FALSE

new.df.cov$large.residual <- new.df.cov$standardized.residuals > 2 | new.df.cov$standardized.residuals < -2
sum(new.df.cov$large.residual)

## [1] 6

new.df.cov[new.df.cov$large.residual, c("X1", "standardized.residuals")]

## # A tibble: 6 x 2
## X1 standardized.residuals
## <dbl> <dbl>
## 1 25 2.00
## 2 48 -2.43
## 3 60 4.61
## 4 64 -3.06
## 5 137 2.35
## 6 169 2.46

new.df.cov$cooks.distance <- cooks.distance(model.y.cov)
new.df.cov$leverage <- hatvalues(model.y.cov)
new.df.cov$covariance <- covratio(model.y.cov)

new.df.cov[new.df.cov$large.residual, c("X1", "cooks.distance", "leverage", "covariance" )]

## # A tibble: 6 x 4
## X1 cooks.distance leverage covariance
## <dbl> <dbl> <dbl> <dbl>
## 1 25 0.0202 0.0434 0.870
## 2 48 0.0428 0.0610 0.787
## 3 60 0.120 0.0485 0.283
## 4 64 0.118 0.102 0.662
## 5 137 0.0403 0.0618 0.808
## 6 169 0.0278 0.0397 0.763

Remove cases 60 and 64

new.df.cov %>%
 filter ( ! (X1 == 60)) %>%
 filter (!( X1 == 64)) -> new.df.cov

Build models again

model.m.cov <- lm(Loneliness ~ Resta + gender + age + ethnicity.dicotomised +
 sexuality.dicotomised + relationship.dicotomised +
 employment.dicotomised, data = new.df.cov)

model.y.cov <- lm(SWEMWBS ~ Resta + Loneliness+ gender + age + ethnicity.dicotomised + sexuality.dicotomised + relationship.dicotomised +
 employment.dicotomised, data = new.df.cov)

Re-check assumptions

new.df.cov$standardized.residuals <- rstandard(model.y.cov)
plot(model.y.cov)


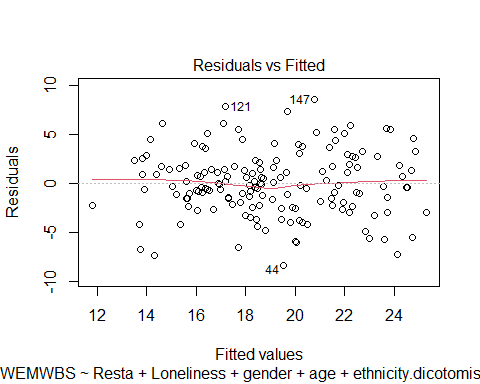

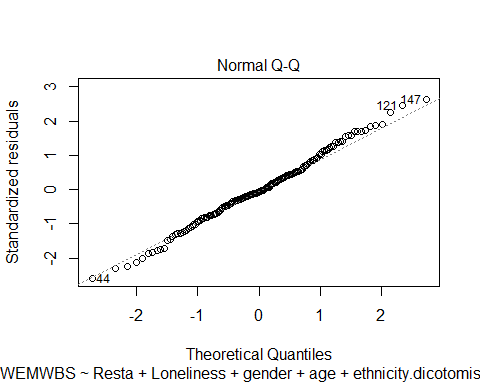

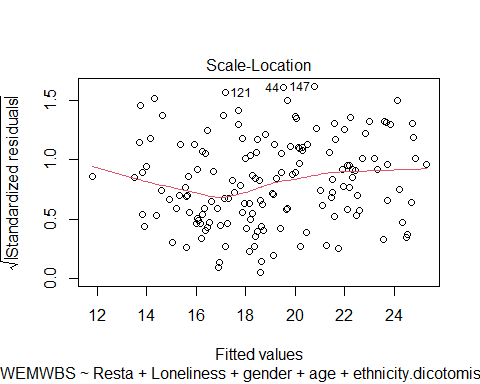

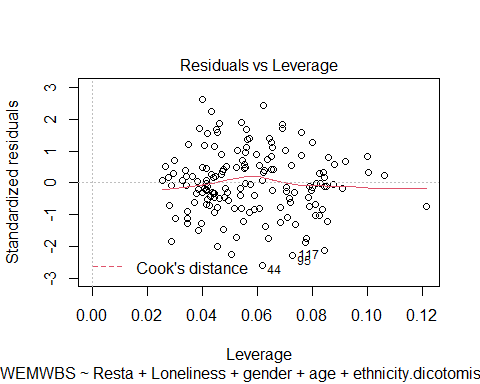


hist(new.df.cov$standardized.residuals)


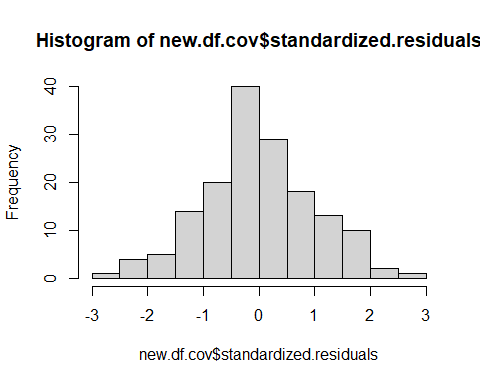


shapiro.test(new.df.cov$standardized.residuals)

##
## Shapiro-Wilk normality test
##
## data: new.df.cov$standardized.residuals
## W = 0.99495, p-value = 0.8689

Run analysis

med.cov<- mediate(model.m.cov , model.y.cov, sims = 5000,
 treat = "Resta", mediator = "Loneliness")
summary(med.cov)

##
## Causal Mediation Analysis
##
## Quasi-Bayesian Confidence Intervals
##
## Estimate 95% CI Lower 95% CI Upper p-value
## ACME 0.3165 0.2007 0.45 <2e-16 ***
## ADE 0.1959 0.0262 0.36 0.022 *
## Total Effect 0.5124 0.3616 0.66 <2e-16 ***
## Prop. Mediated 0.6189 0.3834 0.94 <2e-16 ***
## ---
## Signif. codes: 0 '***' 0.001 '**' 0.01 '*' 0.05 '.' 0.1 ' ' 1
##
## Sample Size Used: 157
##
##
## Simulations: 5000

## CAPE POSITIVE

test mediation model : resta -> loneliness -> CAPE pos

Create data frame to work from

new.df2 <- data.frame(df$Resta.total, df$LonelinessTotal, df$CAPE_positive)

#remove any rows with NAs
new.df2 %>%
 filter(! is.na(df.LonelinessTotal)) -> new.df2

Build models

model.m2 <- lm(df.LonelinessTotal ~ df.Resta.total, data = new.df2)
model.y2 <- lm(df.CAPE_positive ~ df.Resta.total + df.LonelinessTotal, data = new.df2 )

Check parametric assumptions and for outliers

new.df2$standardized.residuals <- rstandard(model.y2)
plot(model.y2)


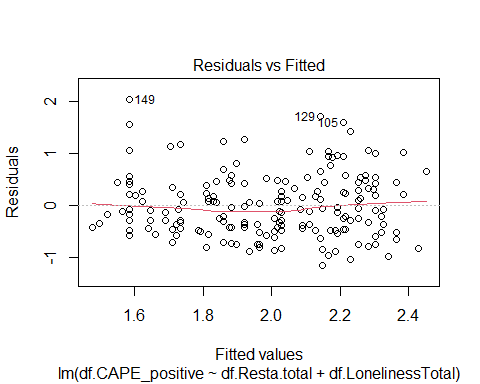

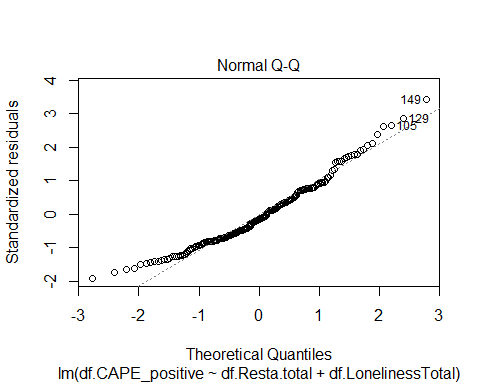

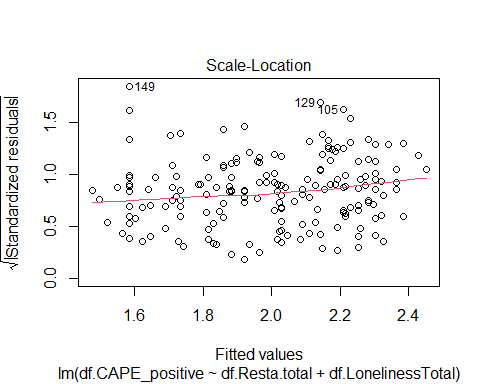

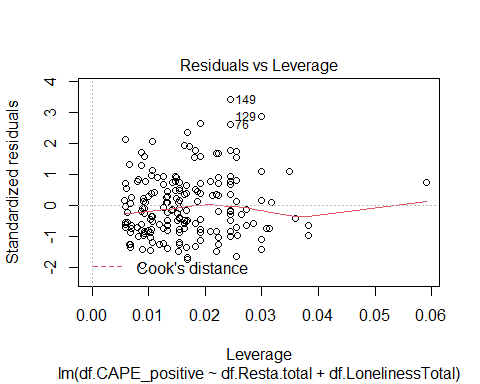


hist(new.df2$standardized.residuals)


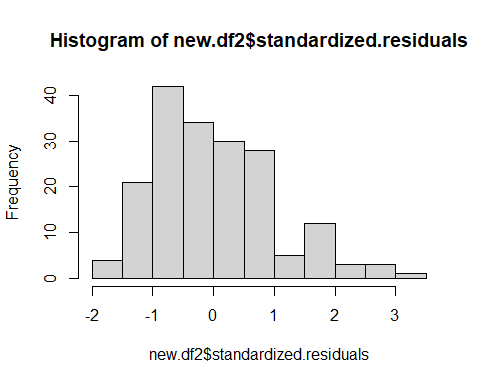


shapiro.test(new.df2$standardized.residuals)

##
## Shapiro-Wilk normality test
##
## data: new.df2$standardized.residuals
## W = 0.96364, p-value = 0.0001095

new.df2$standardized.residuals > 2 | new.df2$standardized.residuals < -2

## [1] FALSE FALSE FALSE FALSE FALSE FALSE FALSE FALSE FALSE FALSE FALSE FALSE
## [13] FALSE FALSE FALSE FALSE FALSE FALSE FALSE FALSE FALSE FALSE FALSE FALSE
## [25] FALSE FALSE FALSE FALSE FALSE FALSE FALSE FALSE FALSE FALSE FALSE FALSE
## [37] FALSE FALSE FALSE FALSE FALSE FALSE FALSE FALSE FALSE FALSE FALSE FALSE
## [49] FALSE FALSE FALSE FALSE FALSE FALSE FALSE FALSE FALSE FALSE FALSE FALSE
## [61] FALSE FALSE FALSE FALSE FALSE FALSE FALSE FALSE FALSE FALSE FALSE FALSE
## [73] FALSE FALSE FALSE TRUE FALSE FALSE FALSE FALSE FALSE FALSE FALSE FALSE
## [85] FALSE FALSE FALSE FALSE FALSE FALSE FALSE FALSE FALSE FALSE FALSE FALSE
## [97] FALSE FALSE FALSE FALSE FALSE FALSE TRUE FALSE TRUE FALSE FALSE TRUE
## [109] FALSE FALSE FALSE FALSE FALSE FALSE FALSE FALSE FALSE FALSE FALSE FALSE
## [121] FALSE FALSE FALSE FALSE FALSE FALSE FALSE FALSE TRUE FALSE FALSE FALSE
## [133] FALSE FALSE FALSE FALSE FALSE FALSE FALSE FALSE FALSE FALSE FALSE FALSE
## [145] FALSE FALSE FALSE FALSE TRUE FALSE FALSE FALSE FALSE TRUE FALSE FALSE
## [157] FALSE FALSE FALSE FALSE FALSE FALSE FALSE FALSE FALSE FALSE FALSE FALSE
## [169] FALSE FALSE FALSE FALSE FALSE FALSE FALSE FALSE FALSE FALSE FALSE FALSE
## [181] FALSE FALSE FALSE

new.df2$large.residual <- new.df2$standardized.residuals > 2 | new.df2$standardized.residuals < -2
sum(new.df2$large.residual)

## [1] 7

new.df2[new.df2$large.residual, c( "standardized.residuals")]

## [1] 2.618931 2.124046 2.656677 2.062297 2.866365 3.414336 2.366780

new.df2$cooks.distance <- cooks.distance(model.y2)
new.df2$leverage <- hatvalues(model.y2)
new.df2$covariance <- covratio(model.y2)

new.df2[new.df2$large.residual, c("cooks.distance", "leverage", "covariance" )]

## cooks.distance leverage covariance
## 76 0.057199507 0.024408085 0.9276283
## 103 0.009033852 0.005971253 0.9479544
## 105 0.045806811 0.019098499 0.9194274
## 108 0.015388523 0.010738095 0.9567377
## 129 0.084373230 0.029887202 0.9111009
## 149 0.097220283 0.024408085 0.8526141
## 154 0.032174646 0.016939460 0.9407794

Run analysis with outlier then remove outlier and re-build model

med.with2 <- mediate(model.m2 , model.y2, sims = 1000, boot = TRUE,
 treat = "df.Resta.total",
 mediator = "df.LonelinessTotal")

## Running nonparametric bootstrap

summary(med.with2)

##
## Causal Mediation Analysis
##
## Nonparametric Bootstrap Confidence Intervals with the Percentile Method
##
## Estimate 95% CI Lower 95% CI Upper p-value
## ACME -0.03287 -0.04732 -0.02 <2e-16 ***
## ADE 0.02203 0.00108 0.05 0.04 *
## Total Effect -0.01083 -0.03366 0.01 0.32
## Prop. Mediated 3.03354 -16.61439 36.08 0.32
## ---
## Signif. codes: 0 '***' 0.001 '**' 0.01 '*' 0.05 '.' 0.1 ' ' 1
##
## Sample Size Used: 183
##
##
## Simulations: 1000

new.df2.OR <- new.df2[-c(149),]

model.m2.OR <- lm(df.LonelinessTotal ~ df.Resta.total, data = new.df2.OR)
model.y2.OR <- lm(df.CAPE_positive ~ df.Resta.total + df.LonelinessTotal, data = new.df2.OR )

Re-check assumptions

new.df2.OR$standardized.residuals <- rstandard(model.y2.OR)
plot(model.y2.OR)


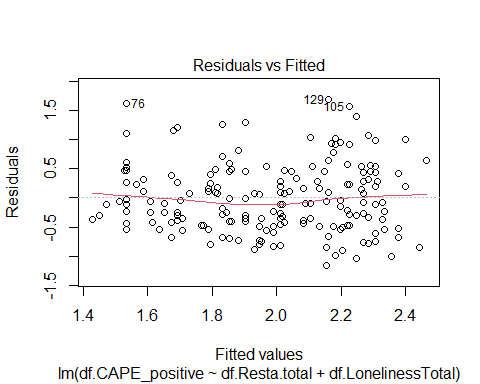

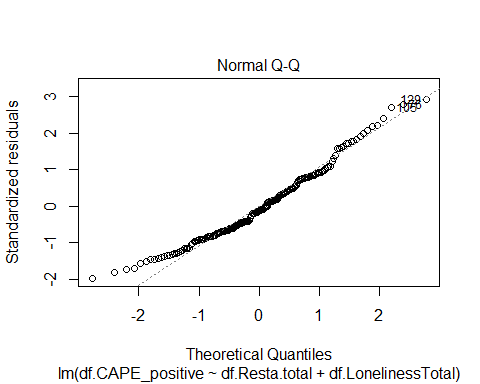

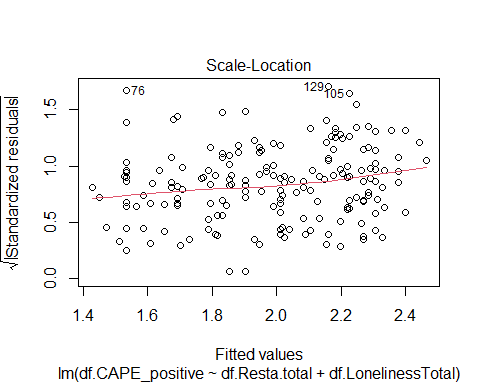

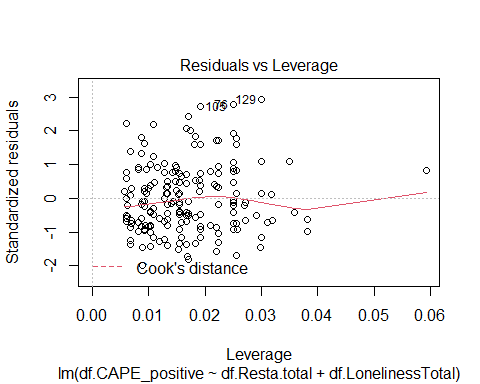


hist(new.df2.OR$standardized.residuals)


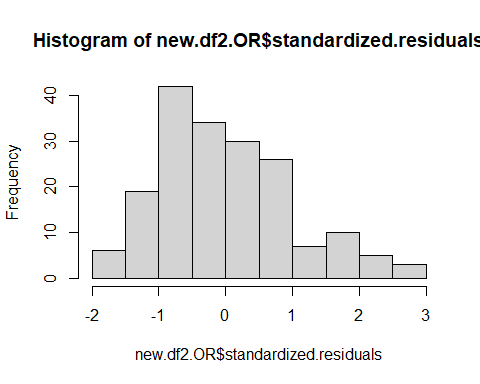


shapiro.test(new.df2.OR$standardized.residuals)

##
## Shapiro-Wilk normality test
##
## data: new.df2.OR$standardized.residuals
## W = 0.97119, p-value = 0.0008135

Re-run analysis

med.without2<- mediate(model.m2.OR , model.y2.OR, sims = 1000, boot = TRUE,
 treat = "df.Resta.total",
 mediator = "df.LonelinessTotal")

## Running nonparametric bootstrap

summary(med.without2)

##
## Causal Mediation Analysis
##
## Nonparametric Bootstrap Confidence Intervals with the Percentile Method
##
## Estimate 95% CI Lower 95% CI Upper p-value
## ACME -0.03472 -0.04861 -0.02 <2e-16 ***
## ADE 0.02162 -0.00078 0.04 0.056 .
## Total Effect -0.01310 -0.03448 0.01 0.238
## Prop. Mediated 2.65052 -20.10682 31.88 0.238
## ---
## Signif. codes: 0 '***' 0.001 '**' 0.01 '*' 0.05 '.' 0.1 ' ' 1
##
## Sample Size Used: 182
##
##
## Simulations: 1000

Add in co-variates

#upload previous dataframe to work from and add in CAPE positive variable
new.df.cov2 <- read_csv("new.df.cov_2022.csv")

## Warning: Missing column names filled in: 'X1' [1]

##
## -- Column specification --------------------------------------------------------
## cols(
## X1 = col_double(),
## Resta = col_double(),
## Loneliness = col_double(),
## gender = col_character(),
## age = col_double(),
## ethnicity = col_character(),
## sexuality = col_character(),
## relationship.status = col_character(),
## SWEMWBS = col_double(),
## employment = col_character(),
## ethnicity.dicotomised = col_character(),
## sexuality.dicotomised = col_character(),
## relationship.dicotomised = col_character(),
## employment.dicotomised = col_character()
## )

new.df.cov2$CAPE_pos <- df$CAPE_positive

#remove rows with missing data to allow for mediation package to work
new.df.cov2 <- na.omit(new.df.cov2)

Create model with co-variates

model.m2.cov <- lm(Loneliness ~ Resta + gender + age + ethnicity.dicotomised +
 sexuality.dicotomised + relationship.dicotomised +
 employment.dicotomised, data = new.df.cov2)

model.y2.cov <- lm(CAPE_pos ~ Resta + Loneliness+ gender + age + ethnicity.dicotomised + sexuality.dicotomised + relationship.dicotomised +
 employment.dicotomised, data = new.df.cov2)

Check parametric assumptions and for outliers

new.df.cov2$standardized.residuals <- rstandard(model.y2.cov)
plot(model.y2.cov)


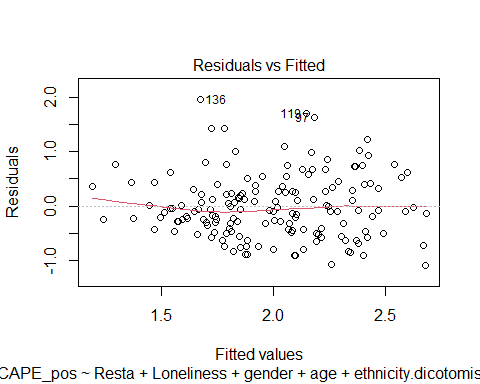

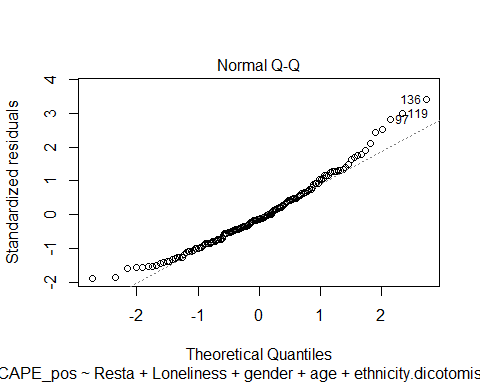

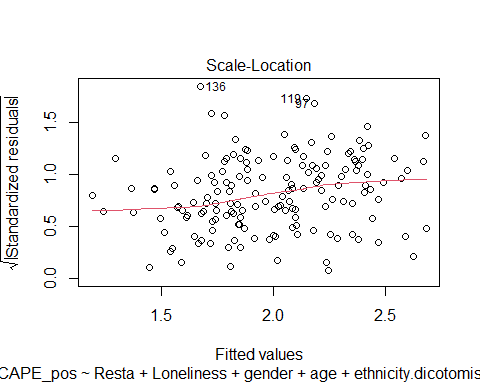

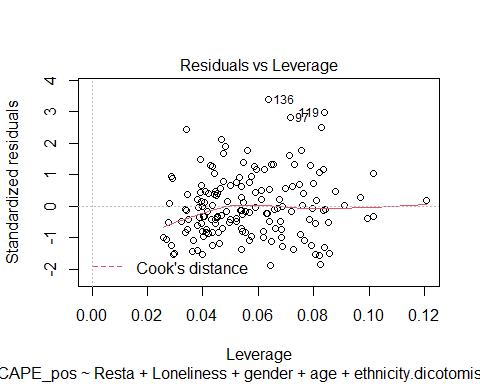


hist(new.df.cov2$standardized.residuals)


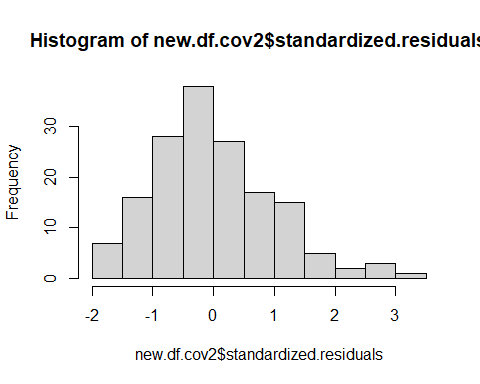


shapiro.test(new.df.cov2$standardized.residuals)

##
## Shapiro-Wilk normality test
##
## data: new.df.cov2$standardized.residuals
## W = 0.96784, p-value = 0.0009208

new.df.cov2$standardized.residuals > 2 |new.df.cov2$standardized.residuals < -2

## 1 2 3 4 5 6 7 8 9 10 11 12 13
## FALSE FALSE FALSE FALSE FALSE FALSE FALSE FALSE FALSE FALSE FALSE FALSE FALSE
## 14 15 16 17 18 19 20 21 22 23 24 25 26
## FALSE FALSE FALSE FALSE FALSE FALSE FALSE FALSE FALSE FALSE FALSE FALSE FALSE
## 27 28 29 30 31 32 33 34 35 36 37 38 39
## FALSE FALSE FALSE FALSE FALSE FALSE FALSE FALSE FALSE FALSE FALSE FALSE FALSE
## 40 41 42 43 44 45 46 47 48 49 50 51 52
## FALSE FALSE FALSE FALSE FALSE FALSE FALSE FALSE FALSE FALSE FALSE FALSE FALSE
## 53 54 55 56 57 58 59 60 61 62 63 64 65
## FALSE FALSE FALSE FALSE FALSE FALSE FALSE FALSE FALSE FALSE FALSE FALSE FALSE
## 66 67 68 69 70 71 72 73 74 75 76 77 78
## FALSE FALSE FALSE TRUE FALSE FALSE FALSE FALSE FALSE FALSE FALSE FALSE FALSE
## 79 80 81 82 83 84 85 86 87 88 89 90 91
## FALSE FALSE FALSE FALSE FALSE FALSE FALSE FALSE FALSE FALSE FALSE FALSE FALSE
## 92 93 94 95 96 97 98 99 100 101 102 103 104
## FALSE FALSE FALSE TRUE FALSE TRUE FALSE FALSE FALSE FALSE FALSE FALSE FALSE
## 105 106 107 108 109 110 111 112 113 114 115 116 117
## FALSE FALSE FALSE FALSE FALSE FALSE FALSE FALSE FALSE FALSE FALSE FALSE FALSE
## 118 119 120 121 122 123 124 125 126 127 128 129 130
## FALSE TRUE FALSE FALSE FALSE FALSE FALSE FALSE FALSE FALSE FALSE FALSE FALSE
## 131 132 133 134 135 136 137 138 139 140 141 142 143
## FALSE FALSE FALSE FALSE FALSE TRUE FALSE FALSE FALSE TRUE FALSE FALSE FALSE
## 144 145 146 147 148 149 150 151 152 153 154 155 156
## FALSE FALSE FALSE FALSE FALSE FALSE FALSE FALSE FALSE FALSE FALSE FALSE FALSE
## 157 158 159
## FALSE FALSE FALSE

new.df.cov2$large.residual <- new.df.cov2$standardized.residuals > 2 | new.df.cov2$standardized.residuals < -2
sum(new.df.cov2$large.residual)

## [1] 6

new.df.cov2[new.df.cov2$large.residual, c("X1", "standardized.residuals")]

## # A tibble: 6 x 2
## X1 standardized.residuals
## <dbl> <dbl>
## 1 78 2.51
## 2 105 2.43
## 3 107 2.83
## 4 131 2.99
## 5 154 3.39
## 6 159 2.12

new.df.cov2$cooks.distance <- cooks.distance(model.y2.cov)
new.df.cov2$leverage <- hatvalues(model.y2.cov)
new.df.cov2$covariance <- covratio(model.y2.cov)

new.df.cov2[new.df.cov2$large.residual, c("X1", "cooks.distance", "leverage", "covariance" )]

## # A tibble: 6 x 4
## X1 cooks.distance leverage covariance
## <dbl> <dbl> <dbl> <dbl>
## 1 78 0.0633 0.0829 0.787
## 2 105 0.0232 0.0342 0.767
## 3 107 0.0684 0.0716 0.699
## 4 131 0.0913 0.0839 0.666
## 5 154 0.0869 0.0636 0.553
## 6 159 0.0245 0.0468 0.848

Remove one outlier

new.df.cov2 %>%
 filter (! (X1 == 154)) -> new.df.cov2

Build models again

model.m2.cov <- lm(Loneliness ~ Resta + gender + age + ethnicity.dicotomised +
 sexuality.dicotomised + relationship.dicotomised +
 employment.dicotomised, data = new.df.cov2)

model.y2.cov <- lm(CAPE_pos ~ Resta + Loneliness+ gender + age + ethnicity.dicotomised + sexuality.dicotomised + relationship.dicotomised +
 employment.dicotomised, data = new.df.cov2)

Re-check assumptions

new.df.cov2$standardized.residuals <- rstandard(model.y2.cov)
plot(model.y2.cov)


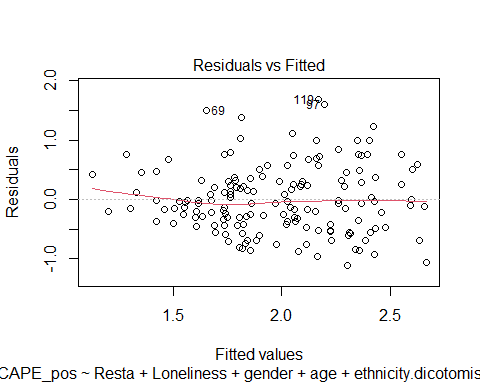

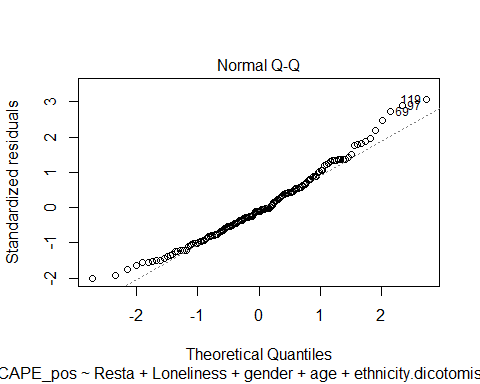

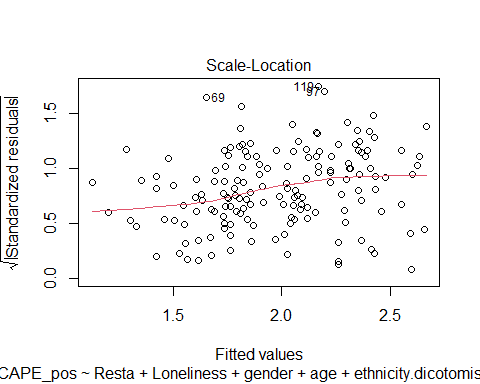

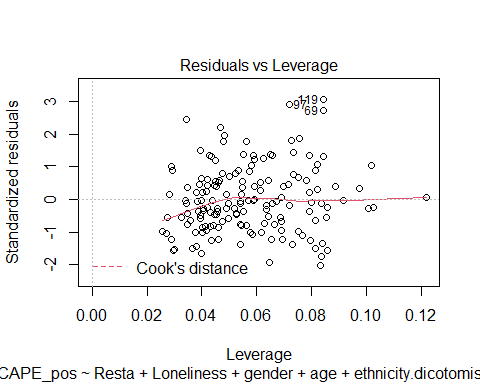


hist(new.df.cov2$standardized.residuals)


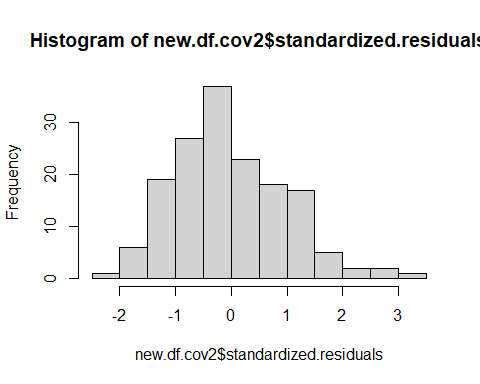


shapiro.test(new.df.cov2$standardized.residuals)

##
## Shapiro-Wilk normality test
##
## data: new.df.cov2$standardized.residuals
## W = 0.97754, p-value = 0.01115

Re-run mediation analysis

med.cov2<- mediate(model.m2.cov , model.y2.cov, sims = 5000, boot = TRUE,
 treat = "Resta", mediator = "Loneliness")

## Running nonparametric bootstrap

summary(med.cov2)

##
## Causal Mediation Analysis
##
## Nonparametric Bootstrap Confidence Intervals with the Percentile Method
##
## Estimate 95% CI Lower 95% CI Upper p-value
## ACME -0.03973 -0.05986 -0.02 <2e-16 ***
## ADE 0.00918 -0.02081 0.04 0.55
## Total Effect -0.03055 -0.05801 0.00 0.03 *
## Prop. Mediated 1.30065 0.49949 5.93 0.03 *
## ---
## Signif. codes: 0 '***' 0.001 '**' 0.01 '*' 0.05 '.' 0.1 ' ' 1
##
## Sample Size Used: 158
##
##
## Simulations: 5000

## CAPE NEGATIVE

Test mediation model : Resta -> Loneliness -> CAPE negative

Create data frame to work from

new.df3 <- data.frame(df$Resta.total, df$LonelinessTotal, df$CAPE_negative)

new.df3 %>%
 filter(! is.na(df.LonelinessTotal)) -> new.df3

Build models and run mediation analysis

model.m3 <- lm(df.LonelinessTotal ~ df.Resta.total, data = new.df3)
model.y3 <- lm(df.CAPE_negative ~ df.Resta.total + df.LonelinessTotal, data = new.df3 )

Check parametric assumptions and for outliers

new.df3$standardized.residuals <- rstandard(model.y3)
plot(model.y3)


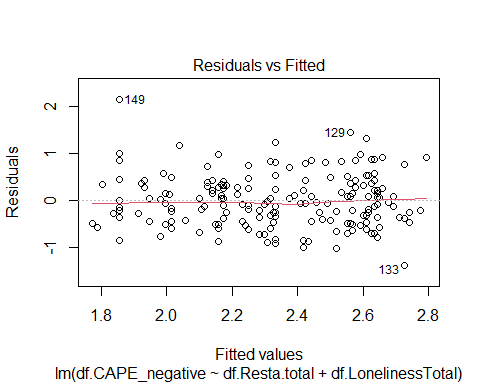

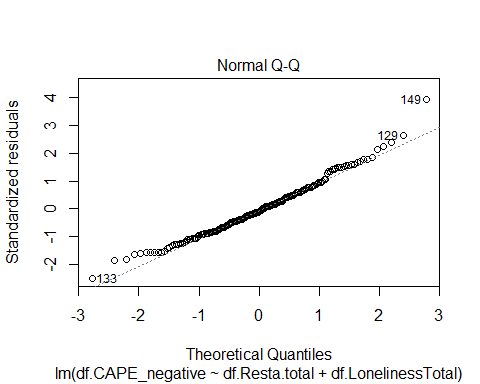

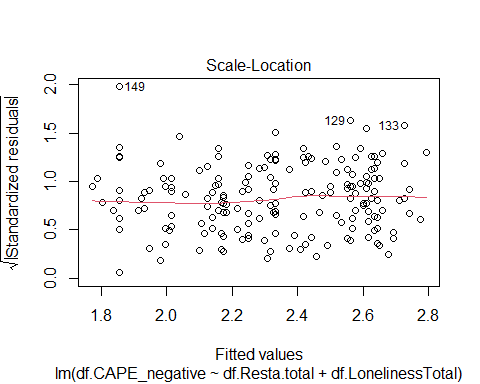

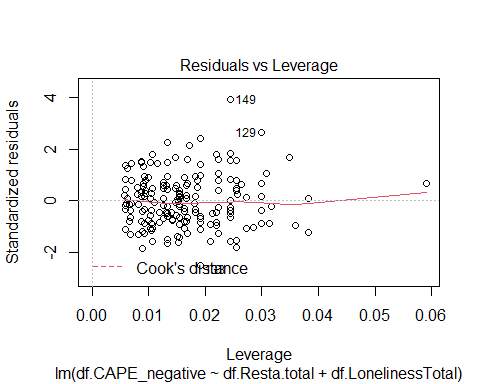


hist(new.df3$standardized.residuals)


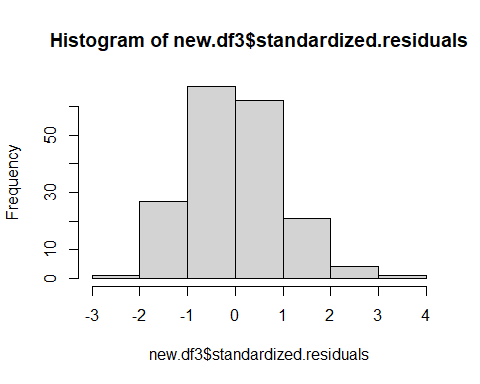


shapiro.test(new.df3$standardized.residuals)

##
## Shapiro-Wilk normality test
##
## data: new.df3$standardized.residuals
## W = 0.9823, p-value = 0.02031

new.df3$standardized.residuals > 2 | new.df3$standardized.residuals < -2

## [1] FALSE FALSE FALSE FALSE FALSE FALSE FALSE FALSE FALSE FALSE FALSE FALSE
## [13] FALSE FALSE FALSE FALSE FALSE FALSE FALSE FALSE FALSE FALSE FALSE FALSE
## [25] FALSE FALSE FALSE FALSE FALSE FALSE FALSE FALSE FALSE FALSE FALSE FALSE
## [37] FALSE FALSE FALSE FALSE FALSE FALSE FALSE FALSE FALSE FALSE FALSE FALSE
## [49] FALSE FALSE FALSE FALSE FALSE FALSE FALSE FALSE FALSE FALSE FALSE FALSE
## [61] FALSE FALSE FALSE FALSE FALSE FALSE FALSE FALSE FALSE FALSE FALSE FALSE
## [73] FALSE FALSE FALSE FALSE FALSE FALSE FALSE FALSE FALSE FALSE FALSE FALSE
## [85] FALSE FALSE FALSE FALSE FALSE FALSE FALSE FALSE FALSE FALSE FALSE FALSE
## [97] FALSE FALSE FALSE FALSE FALSE FALSE FALSE FALSE TRUE FALSE FALSE FALSE
## [109] FALSE FALSE FALSE FALSE FALSE TRUE FALSE FALSE FALSE FALSE FALSE FALSE
## [121] FALSE FALSE FALSE FALSE FALSE FALSE FALSE FALSE TRUE FALSE FALSE FALSE
## [133] TRUE FALSE FALSE FALSE FALSE FALSE FALSE FALSE FALSE FALSE FALSE FALSE
## [145] FALSE FALSE FALSE FALSE TRUE FALSE FALSE FALSE FALSE FALSE FALSE FALSE
## [157] FALSE TRUE FALSE FALSE FALSE FALSE FALSE FALSE FALSE FALSE FALSE FALSE
## [169] FALSE FALSE FALSE FALSE FALSE FALSE FALSE FALSE FALSE FALSE FALSE FALSE
## [181] FALSE FALSE FALSE

new.df3$large.residual <- new.df3$standardized.residuals > 2 | new.df3$standardized.residuals < -2
sum(new.df3$large.residual)

## [1] 6

new.df3[new.df3$large.residual, c("standardized.residuals")]

## [1] 2.410602 2.144158 2.646844 -2.501172 3.930865 2.258509

new.df3$cooks.distance <- cooks.distance(model.y3)
new.df3$leverage <- hatvalues(model.y3)
new.df3$covariance <- covratio(model.y3)

new.df3[new.df3$large.residual, c("cooks.distance", "leverage", "covariance" )]

## cooks.distance leverage covariance
## 105 0.03771410 0.01909850 0.9394586
## 114 0.02696256 0.01728996 0.9574663
## 129 0.07194467 0.02988720 0.9304936
## 133 0.04055201 0.01907577 0.9322576
## 149 0.12886072 0.02440808 0.7962563
## 158 0.02280729 0.01323623 0.9453458

Run analysis with outlier then remove outlier and build models again

med.with3 <- mediate(model.m3 , model.y3, sims = 1000, boot = TRUE,
 treat = "df.Resta.total",
 mediator = "df.LonelinessTotal")

## Running nonparametric bootstrap

summary(med.with3)

##
## Causal Mediation Analysis
##
## Nonparametric Bootstrap Confidence Intervals with the Percentile Method
##
## Estimate 95% CI Lower 95% CI Upper p-value
## ACME -0.03533 -0.04950 -0.02 <2e-16 ***
## ADE 0.01662 -0.00448 0.04 0.142
## Total Effect -0.01871 -0.04159 0.00 0.084 .
## Prop. Mediated 1.88818 -7.93507 13.87 0.084 .
## ---
## Signif. codes: 0 '***' 0.001 '**' 0.01 '*' 0.05 '.' 0.1 ' ' 1
##
## Sample Size Used: 183
##
##
## Simulations: 1000

new.df3.OR <- new.df3[-c(149),]

model.m3.OR <- lm(df.LonelinessTotal ~ df.Resta.total, data = new.df3.OR)
model.y3.OR <- lm(df.CAPE_negative ~ df.Resta.total + df.LonelinessTotal, data = new.df3.OR )

Re-check parametric assumptions

new.df3.OR$standardized.residuals <- rstandard(model.y3.OR)
plot(model.y3.OR)


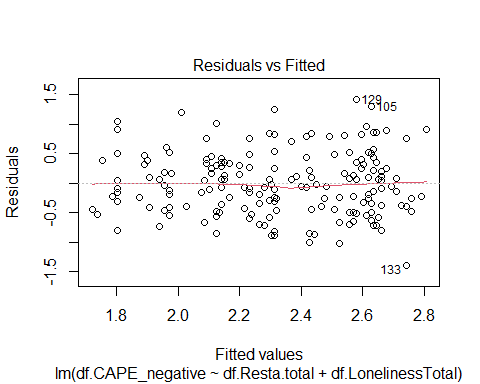

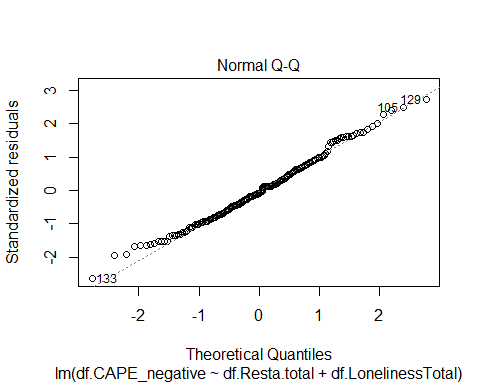

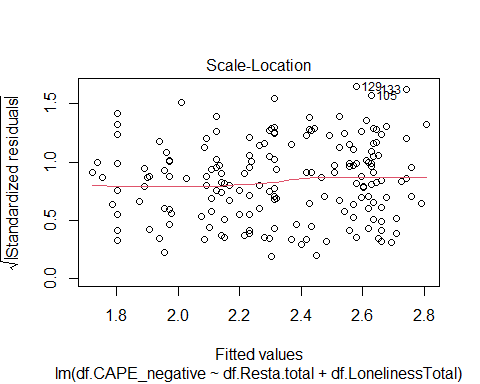

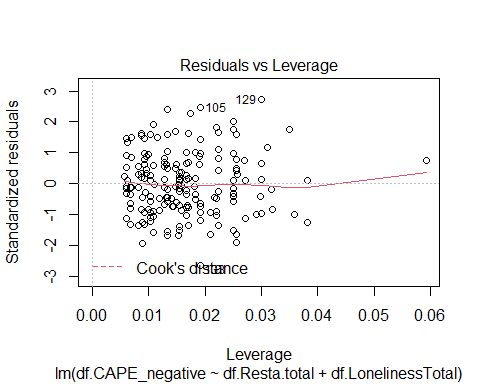


hist(new.df3.OR$standardized.residuals)


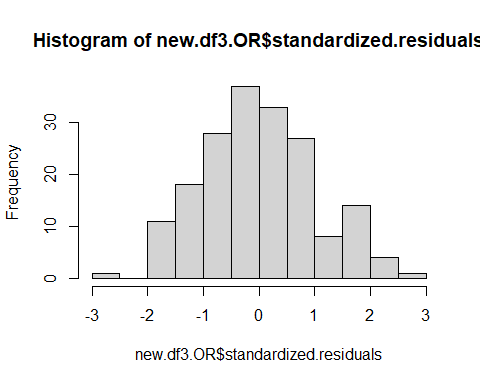


shapiro.test(new.df3.OR$standardized.residuals)

##
## Shapiro-Wilk normality test
##
## data: new.df3.OR$standardized.residuals
## W = 0.99014, p-value = 0.2434

Run mediation analysis

med.without3<- mediate(model.m3.OR , model.y3.OR, sims = 1000,
 treat = "df.Resta.total",
 mediator = "df.LonelinessTotal")
summary(med.without3)

##
## Causal Mediation Analysis
##
## Quasi-Bayesian Confidence Intervals
##
## Estimate 95% CI Lower 95% CI Upper p-value
## ACME -0.03715 -0.05142 -0.02 <2e-16 ***
## ADE 0.01602 -0.00258 0.04 0.118
## Total Effect -0.02113 -0.04049 0.00 0.024 *
## Prop. Mediated 1.75592 0.87061 9.09 0.024 *
## ---
## Signif. codes: 0 '***' 0.001 '**' 0.01 '*' 0.05 '.' 0.1 ' ' 1
##
## Sample Size Used: 182
##
##
## Simulations: 1000

Add in co-variates

Create data frame

#upload previous data frame to work from and add in CAPE negative variable
new.df.cov3 <- read_csv("new.df.cov_2022.csv")

## Warning: Missing column names filled in: 'X1' [1]

##
## -- Column specification --------------------------------------------------------
## cols(
## X1 = col_double(),
## Resta = col_double(),
## Loneliness = col_double(),
## gender = col_character(),
## age = col_double(),
## ethnicity = col_character(),
## sexuality = col_character(),
## relationship.status = col_character(),
## SWEMWBS = col_double(),
## employment = col_character(),
## ethnicity.dicotomised = col_character(),
## sexuality.dicotomised = col_character(),
## relationship.dicotomised = col_character(),
## employment.dicotomised = col_character()
## )

new.df.cov3$CAPE_neg <- df$CAPE_negative

#remove rows with missing data to allow for mediation package to work
new.df.cov3 <- na.omit(new.df.cov3)

Create models including co-variates

model.m3.cov <- lm(Loneliness ~ Resta + gender + age + ethnicity.dicotomised +
 sexuality.dicotomised + relationship.dicotomised +
 employment.dicotomised, data = new.df.cov3)

model.y3.cov <- lm(CAPE_neg ~ Resta + Loneliness+ gender + age + ethnicity.dicotomised + sexuality.dicotomised + relationship.dicotomised +
 employment.dicotomised, data = new.df.cov3)

Check assumptions and for outliers

new.df.cov3$standardized.residuals <- rstandard(model.y3.cov)
plot(model.y3.cov)


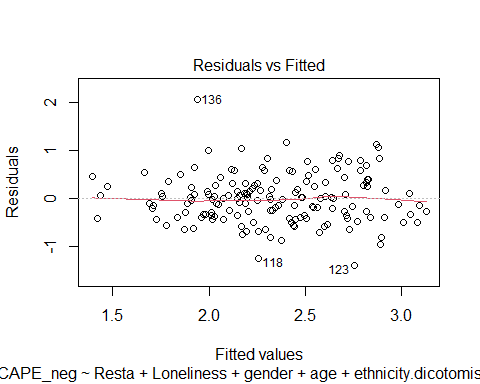

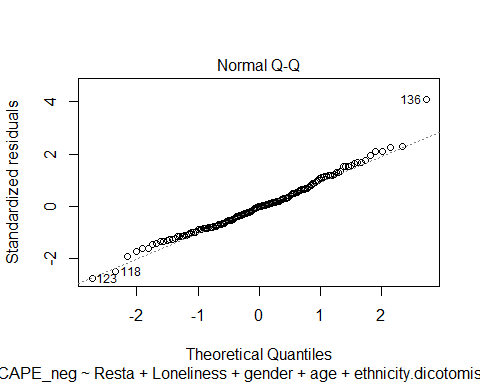

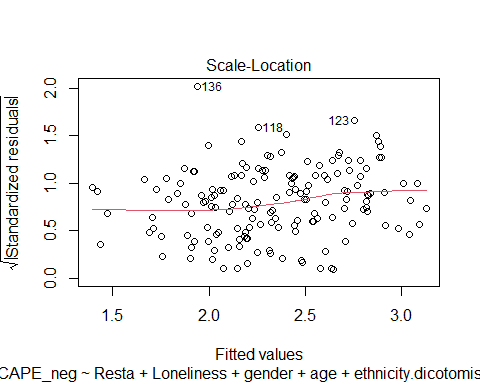

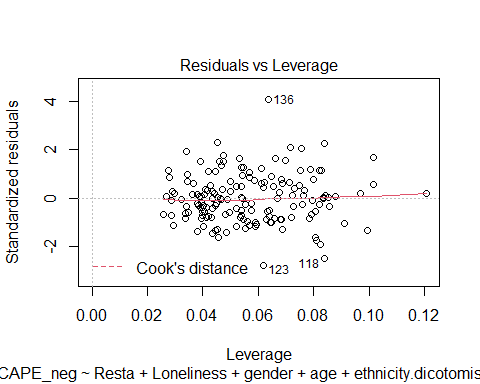


hist(new.df.cov3$standardized.residuals)


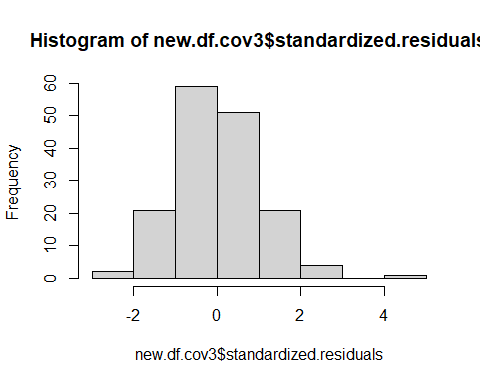


shapiro.test(new.df.cov3$standardized.residuals)

##
## Shapiro-Wilk normality test
##
## data: new.df.cov3$standardized.residuals
## W = 0.98197, p-value = 0.03607

new.df.cov3$standardized.residuals > 2 |new.df.cov3$standardized.residuals < -2

## 1 2 3 4 5 6 7 8 9 10 11 12 13
## FALSE FALSE FALSE FALSE FALSE FALSE FALSE FALSE FALSE FALSE FALSE FALSE FALSE
## 14 15 16 17 18 19 20 21 22 23 24 25 26
## FALSE FALSE FALSE FALSE FALSE FALSE FALSE FALSE FALSE FALSE FALSE FALSE FALSE
## 27 28 29 30 31 32 33 34 35 36 37 38 39
## FALSE FALSE FALSE FALSE FALSE FALSE FALSE FALSE FALSE FALSE FALSE FALSE FALSE
## 40 41 42 43 44 45 46 47 48 49 50 51 52
## FALSE FALSE FALSE FALSE FALSE FALSE FALSE FALSE FALSE FALSE FALSE FALSE FALSE
## 53 54 55 56 57 58 59 60 61 62 63 64 65
## FALSE FALSE FALSE FALSE FALSE FALSE FALSE FALSE FALSE FALSE FALSE FALSE FALSE
## 66 67 68 69 70 71 72 73 74 75 76 77 78
## FALSE FALSE FALSE FALSE FALSE FALSE FALSE FALSE FALSE FALSE FALSE FALSE FALSE
## 79 80 81 82 83 84 85 86 87 88 89 90 91
## FALSE FALSE FALSE FALSE FALSE FALSE FALSE FALSE FALSE FALSE FALSE FALSE FALSE
## 92 93 94 95 96 97 98 99 100 101 102 103 104
## FALSE FALSE FALSE FALSE FALSE TRUE FALSE FALSE FALSE FALSE FALSE FALSE FALSE
## 105 106 107 108 109 110 111 112 113 114 115 116 117
## FALSE TRUE FALSE FALSE FALSE FALSE FALSE FALSE FALSE FALSE FALSE FALSE FALSE
## 118 119 120 121 122 123 124 125 126 127 128 129 130
## TRUE TRUE FALSE FALSE FALSE TRUE FALSE FALSE FALSE FALSE FALSE FALSE FALSE
## 131 132 133 134 135 136 137 138 139 140 141 142 143
## FALSE FALSE FALSE FALSE FALSE TRUE FALSE FALSE FALSE FALSE FALSE FALSE TRUE
## 144 145 146 147 148 149 150 151 152 153 154 155 156
## FALSE FALSE FALSE FALSE FALSE FALSE FALSE FALSE FALSE FALSE FALSE FALSE FALSE
## 157 158 159
## FALSE FALSE FALSE

new.df.cov3$large.residual <- new.df.cov3$standardized.residuals > 2 | new.df.cov3$standardized.residuals < -2
sum(new.df.cov3$large.residual)

## [1] 7

new.df.cov3[new.df.cov3$large.residual, c("X1", "standardized.residuals")]

## # A tibble: 7 x 2
## X1 standardized.residuals
## <dbl> <dbl>
## 1 107 2.09
## 2 116 2.08
## 3 130 -2.51
## 4 131 2.27
## 5 137 -2.77
## 6 154 4.08
## 7 163 2.30

new.df.cov3$cooks.distance <- cooks.distance(model.y3.cov)
new.df.cov3$leverage <- hatvalues(model.y3.cov)
new.df.cov3$covariance <- covratio(model.y3.cov)

new.df.cov3[new.df.cov3$large.residual, c("X1", "cooks.distance", "leverage", "covariance" )]

## # A tibble: 7 x 4
## X1 cooks.distance leverage covariance
## <dbl> <dbl> <dbl> <dbl>
## 1 107 0.0374 0.0716 0.877
## 2 116 0.0393 0.0754 0.882
## 3 130 0.0642 0.0838 0.787
## 4 131 0.0524 0.0839 0.846
## 5 137 0.0561 0.0618 0.706
## 6 154 0.126 0.0636 0.393
## 7 163 0.0279 0.0454 0.806

Remove outlier and build models again

new.df.cov3 %>%
 filter (! (X1 == 154)) -> new.df.cov3

model.m3.cov <- lm(Loneliness ~ Resta + gender + age + ethnicity.dicotomised +
 sexuality.dicotomised + relationship.dicotomised +
 employment.dicotomised, data = new.df.cov3)

model.y3.cov <- lm(CAPE_neg ~ Resta + Loneliness+ gender + age + ethnicity.dicotomised + sexuality.dicotomised + relationship.dicotomised +
 employment.dicotomised, data = new.df.cov3)

Re-check assumptions

new.df.cov3$standardized.residuals <- rstandard(model.y3.cov)
plot(model.y3.cov)


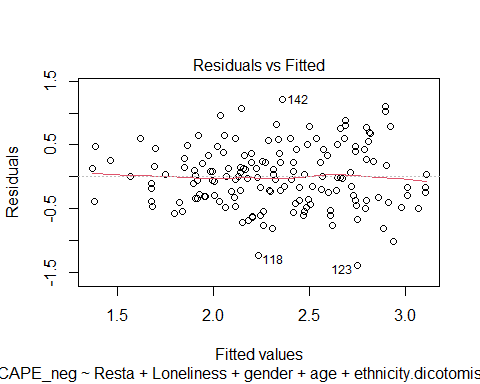

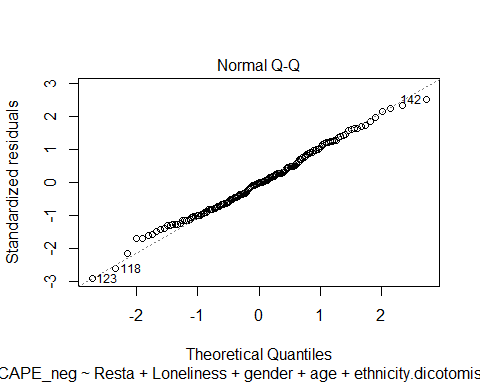

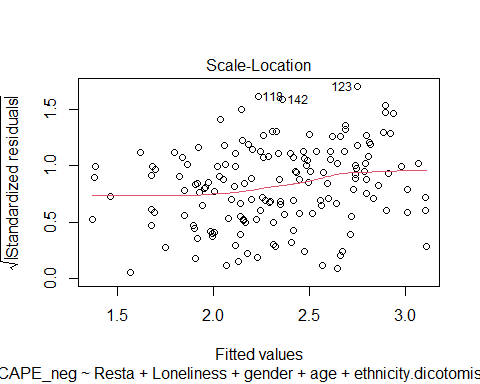

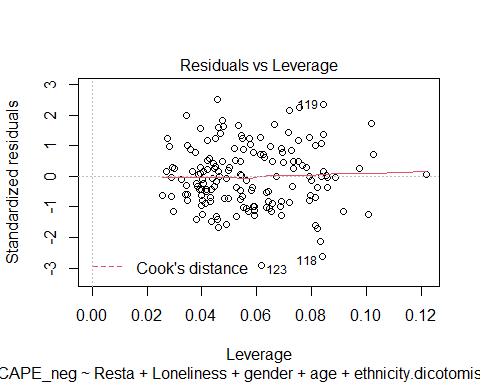


hist(new.df.cov3$standardized.residuals)


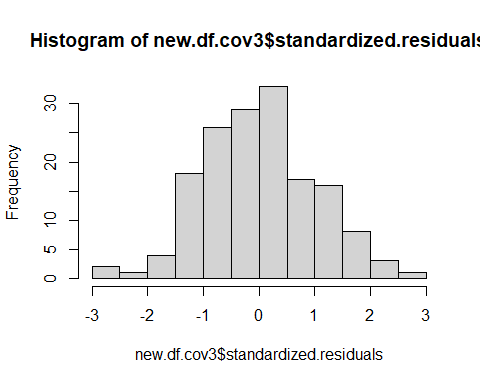


shapiro.test(new.df.cov3$standardized.residuals)

##
## Shapiro-Wilk normality test
##
## data: new.df.cov3$standardized.residuals
## W = 0.99295, p-value = 0.6369

Run mediation analysis

med.cov3 <- mediate(model.m3.cov, model.y3.cov, sims = 5000, treat = "Resta", mediator = "Loneliness")

summary(med.cov3)

##
## Causal Mediation Analysis
##
## Quasi-Bayesian Confidence Intervals
##
## Estimate 95% CI Lower 95% CI Upper p-value
## ACME -0.04542 -0.06382 -0.03 <2e-16 ***
## ADE 0.00109 -0.02316 0.03 0.94
## Total Effect -0.04433 -0.06569 -0.02 <2e-16 ***
## Prop. Mediated 1.02221 0.59924 2.05 <2e-16 ***
## ---
## Signif. codes: 0 '***' 0.001 '**' 0.01 '*' 0.05 '.' 0.1 ' ' 1
##
## Sample Size Used: 158
##
##
## Simulations: 5000

## CAPE DEPRESSIVE

Test mediation model : Resta -> Loneliness -> CAPE depressive

Create data frame

new.df4 <- data.frame(df$Resta.total, df$LonelinessTotal, df$CAPE_depressive)

new.df4%>%
 filter(! is.na(df.LonelinessTotal)) -> new.df4

Build models

model.m4 <- lm(df.LonelinessTotal ~ df.Resta.total, data = new.df4)
model.y4 <- lm(df.CAPE_depressive ~ df.Resta.total + df.LonelinessTotal, data = new.df4)

Check parametric assumptions and for outliers

new.df4$standardized.residuals <- rstandard(model.y4)
plot(model.y4)


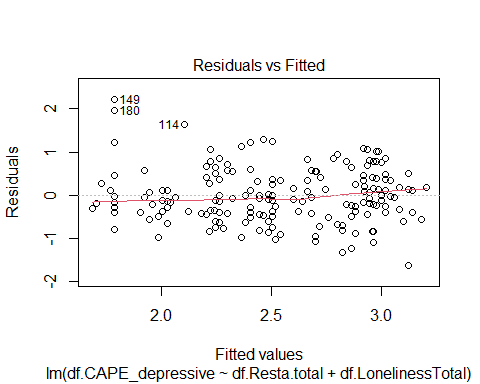

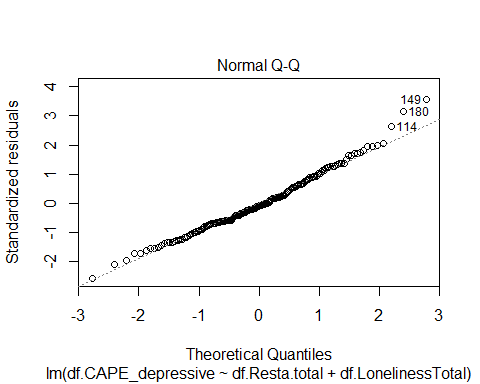

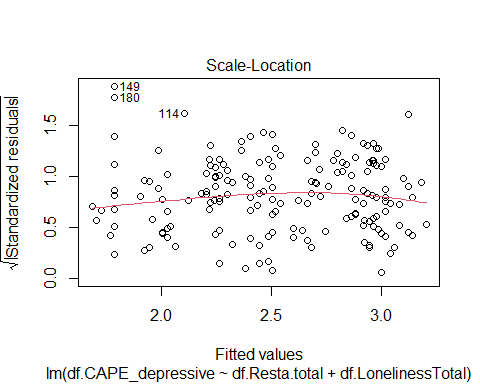

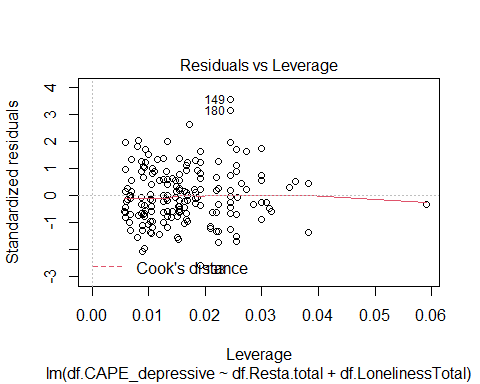


hist(new.df4$standardized.residuals)


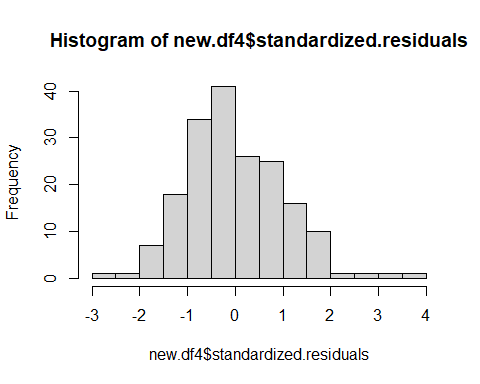


shapiro.test(new.df4$standardized.residuals)

##
## Shapiro-Wilk normality test
##
## data: new.df4$standardized.residuals
## W = 0.98415, p-value = 0.0364

new.df4$standardized.residuals > 2 | new.df4$standardized.residuals < -2

## [1] FALSE FALSE FALSE TRUE FALSE FALSE FALSE FALSE FALSE FALSE FALSE FALSE
## [13] FALSE FALSE FALSE FALSE FALSE FALSE FALSE FALSE FALSE FALSE FALSE FALSE
## [25] FALSE FALSE FALSE FALSE FALSE FALSE FALSE FALSE FALSE FALSE FALSE FALSE
## [37] FALSE FALSE FALSE FALSE FALSE FALSE FALSE FALSE FALSE FALSE FALSE FALSE
## [49] FALSE FALSE FALSE FALSE FALSE FALSE FALSE FALSE FALSE FALSE FALSE FALSE
## [61] FALSE FALSE FALSE FALSE FALSE FALSE FALSE FALSE FALSE FALSE FALSE FALSE
## [73] FALSE FALSE FALSE FALSE FALSE FALSE FALSE FALSE FALSE FALSE FALSE FALSE
## [85] FALSE FALSE FALSE FALSE FALSE FALSE FALSE FALSE FALSE FALSE FALSE FALSE
## [97] FALSE FALSE FALSE FALSE FALSE FALSE FALSE FALSE FALSE FALSE FALSE FALSE
## [109] FALSE FALSE FALSE FALSE FALSE TRUE FALSE FALSE FALSE FALSE FALSE FALSE
## [121] FALSE FALSE FALSE FALSE FALSE FALSE FALSE FALSE FALSE FALSE FALSE FALSE
## [133] TRUE FALSE FALSE FALSE FALSE FALSE FALSE FALSE FALSE FALSE FALSE FALSE
## [145] FALSE FALSE FALSE FALSE TRUE FALSE FALSE FALSE FALSE FALSE FALSE FALSE
## [157] FALSE FALSE FALSE FALSE FALSE FALSE FALSE FALSE FALSE FALSE FALSE FALSE
## [169] FALSE FALSE FALSE FALSE FALSE FALSE FALSE FALSE FALSE FALSE FALSE TRUE
## [181] FALSE TRUE FALSE

new.df4$large.residual <- new.df4$standardized.residuals > 2 | new.df4$standardized.residuals < -2
sum(new.df4$large.residual)

## [1] 6

new.df4[new.df4$large.residual, c( "standardized.residuals")]

## [1] 2.050299 2.639401 -2.594648 3.557678 3.156248 -2.101879

new.df4$cooks.distance <- cooks.distance(model.y4)
new.df4$leverage <- hatvalues(model.y4)
new.df4$covariance <- covratio(model.y4)

new.df4[new.df4$large.residual, c("cooks.distance", "leverage", "covariance" )]

## cooks.distance leverage covariance
## 4 0.01154879 0.008174446 0.9550687
## 114 0.04085622 0.017289963 0.9191927
## 133 0.04363977 0.019075773 0.9246109
## 149 0.10555469 0.024408085 0.8375192
## 180 0.08307811 0.024408085 0.8786409
## 182 0.01334020 0.008977421 0.9523533

Run analysis with outliers first then remove and rebuild models

med.with4 <- mediate(model.m4 , model.y4, sims = 1000, boot = TRUE,
 treat = "df.Resta.total",
 mediator = "df.LonelinessTotal")

## Running nonparametric bootstrap

summary(med.with4)

##
## Causal Mediation Analysis
##
## Nonparametric Bootstrap Confidence Intervals with the Percentile Method
##
## Estimate 95% CI Lower 95% CI Upper p-value
## ACME -0.053254 -0.069688 -0.04 <2e-16 ***
## ADE 0.020434 -0.000852 0.04 0.058 .
## Total Effect -0.032821 -0.056325 -0.01 0.014 *
## Prop. Mediated 1.622586 0.950899 4.64 0.014 *
## ---
## Signif. codes: 0 '***' 0.001 '**' 0.01 '*' 0.05 '.' 0.1 ' ' 1
##
## Sample Size Used: 183
##
##
## Simulations: 1000

new.df4.OR <- new.df4[-c(149, 180),]

model.m4.OR <- lm(df.LonelinessTotal ~ df.Resta.total, data = new.df4.OR)
model.y4.OR <- lm(df.CAPE_depressive ~ df.Resta.total + df.LonelinessTotal, data = new.df4.OR )

Re-check assumptions

new.df4.OR$standardized.residuals <- rstandard(model.y4.OR)
plot(model.y4.OR)


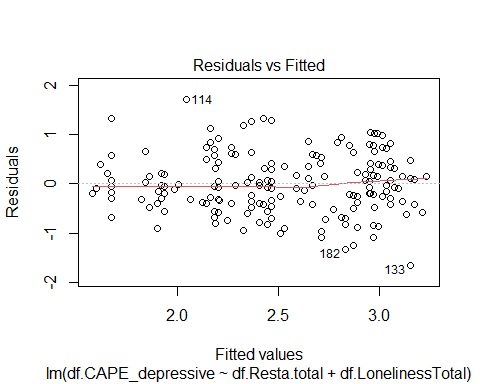

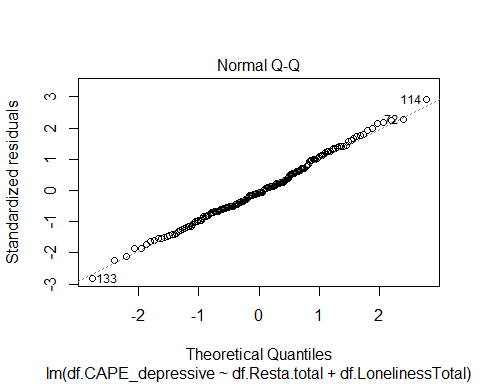

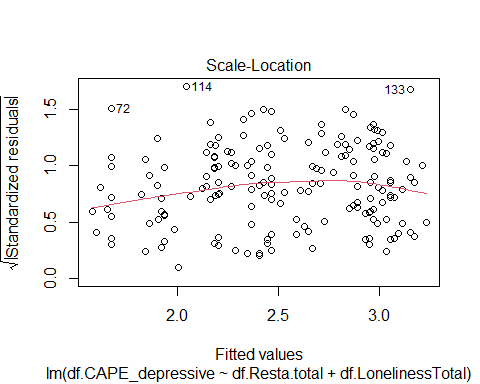

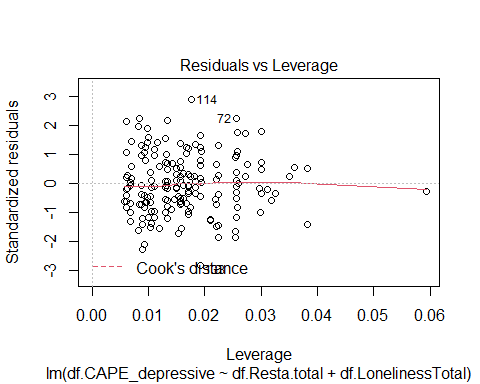


hist(new.df4.OR$standardized.residuals)


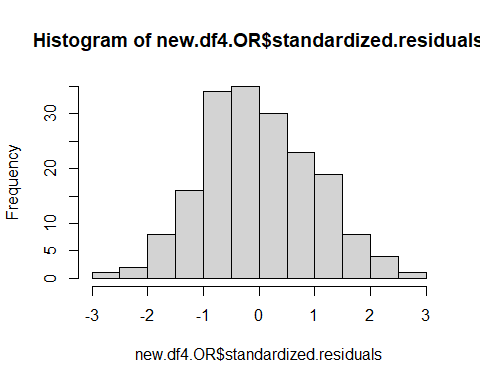


shapiro.test(new.df4.OR$standardized.residuals)

##
## Shapiro-Wilk normality test
##
## data: new.df4.OR$standardized.residuals
## W = 0.99467, p-value = 0.7657

Run mediation analysis

med.without4<- mediate(model.m4.OR , model.y4.OR, sims = 1000,
 treat = "df.Resta.total",
 mediator = "df.LonelinessTotal")
summary(med.without4)

##
## Causal Mediation Analysis
##
## Quasi-Bayesian Confidence Intervals
##
## Estimate 95% CI Lower 95% CI Upper p-value
## ACME -0.05616 -0.07469 -0.04 <2e-16 ***
## ADE 0.01944 -0.00403 0.04 0.094 .
## Total Effect -0.03672 -0.05878 -0.01 0.002 **
## Prop. Mediated 1.52903 0.91322 3.47 0.002 **
## ---
## Signif. codes: 0 '***' 0.001 '**' 0.01 '*' 0.05 '.' 0.1 ' ' 1
##
## Sample Size Used: 181
##
##
## Simulations: 1000

Add in co-variates

Create data frame

new.df.cov4 <- read_csv("new.df.cov_2022.csv")

## Warning: Missing column names filled in: 'X1' [1]

##
## -- Column specification --------------------------------------------------------
## cols(
## X1 = col_double(),
## Resta = col_double(),
## Loneliness = col_double(),
## gender = col_character(),
## age = col_double(),
## ethnicity = col_character(),
## sexuality = col_character(),
## relationship.status = col_character(),
## SWEMWBS = col_double(),
## employment = col_character(),
## ethnicity.dicotomised = col_character(),
## sexuality.dicotomised = col_character(),
## relationship.dicotomised = col_character(),
## employment.dicotomised = col_character()
## )

new.df.cov4$CAPE_dep <- df$CAPE_depressive

new.df.cov4 <- na.omit(new.df.cov4)

Add covariates into models

model.m4.cov <- lm(Loneliness ~ Resta + gender + age + ethnicity.dicotomised +
 sexuality.dicotomised + relationship.dicotomised +
 employment.dicotomised, data = new.df.cov4)

model.y4.cov <- lm(CAPE_dep ~ Resta + Loneliness+ gender + age + ethnicity.dicotomised + sexuality.dicotomised + relationship.dicotomised +
 employment.dicotomised, data = new.df.cov4)

Check assumptions and for outliers

new.df.cov4$standardized.residuals <- rstandard(model.y4.cov)
plot(model.y4.cov)


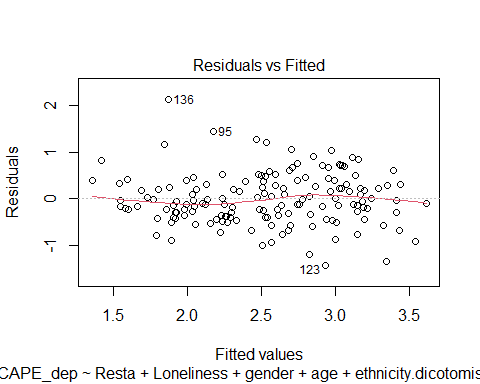

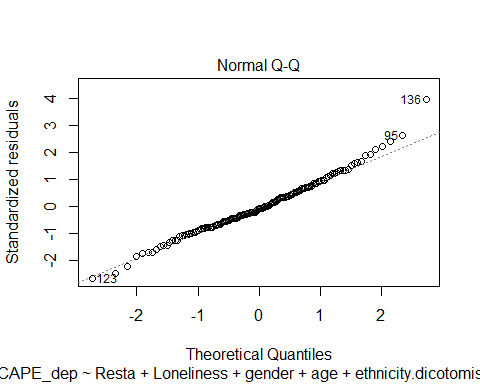

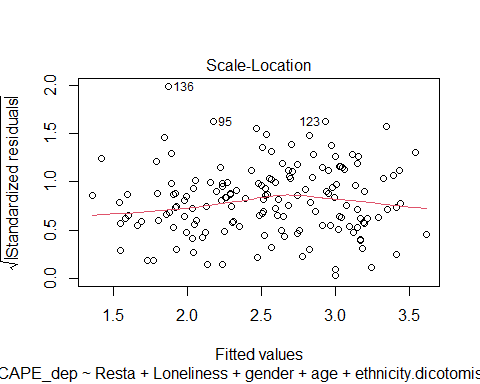

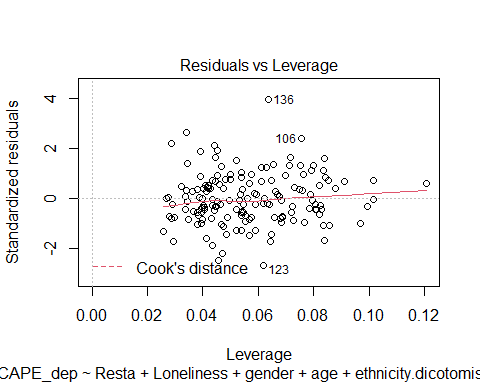


hist(new.df.cov4$standardized.residuals)


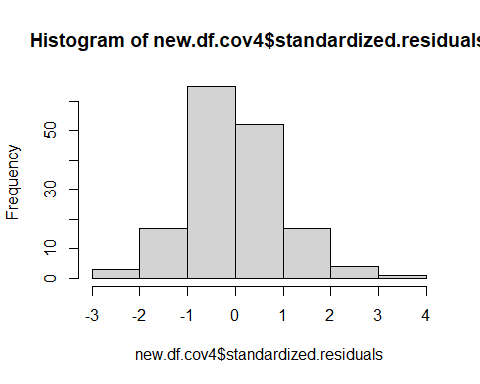


shapiro.test(new.df.cov4$standardized.residuals)

##
## Shapiro-Wilk normality test
##
## data: new.df.cov4$standardized.residuals
## W = 0.98411, p-value = 0.06543

new.df.cov4$standardized.residuals > 2 |new.df.cov4$standardized.residuals < -2

## 1 2 3 4 5 6 7 8 9 10 11 12 13
## FALSE FALSE FALSE TRUE FALSE FALSE FALSE FALSE FALSE FALSE FALSE FALSE FALSE
## 14 15 16 17 18 19 20 21 22 23 24 25 26
## FALSE FALSE FALSE FALSE FALSE FALSE FALSE FALSE FALSE FALSE FALSE FALSE FALSE
## 27 28 29 30 31 32 33 34 35 36 37 38 39
## FALSE FALSE FALSE FALSE FALSE FALSE FALSE FALSE FALSE FALSE FALSE FALSE FALSE
## 40 41 42 43 44 45 46 47 48 49 50 51 52
## FALSE FALSE FALSE FALSE FALSE FALSE FALSE FALSE FALSE FALSE FALSE FALSE FALSE
## 53 54 55 56 57 58 59 60 61 62 63 64 65
## FALSE FALSE FALSE FALSE FALSE FALSE FALSE FALSE FALSE FALSE FALSE FALSE FALSE
## 66 67 68 69 70 71 72 73 74 75 76 77 78
## TRUE FALSE FALSE FALSE FALSE FALSE FALSE FALSE FALSE FALSE FALSE FALSE FALSE
## 79 80 81 82 83 84 85 86 87 88 89 90 91
## FALSE FALSE FALSE FALSE FALSE FALSE FALSE FALSE FALSE FALSE FALSE FALSE TRUE
## 92 93 94 95 96 97 98 99 100 101 102 103 104
## FALSE FALSE TRUE TRUE FALSE FALSE FALSE FALSE FALSE FALSE FALSE FALSE FALSE
## 105 106 107 108 109 110 111 112 113 114 115 116 117
## FALSE TRUE FALSE FALSE FALSE FALSE FALSE FALSE FALSE FALSE FALSE FALSE FALSE
## 118 119 120 121 122 123 124 125 126 127 128 129 130
## FALSE FALSE FALSE FALSE FALSE TRUE FALSE FALSE FALSE FALSE FALSE FALSE FALSE
## 131 132 133 134 135 136 137 138 139 140 141 142 143
## FALSE FALSE FALSE FALSE FALSE TRUE FALSE FALSE FALSE FALSE FALSE FALSE FALSE
## 144 145 146 147 148 149 150 151 152 153 154 155 156
## FALSE FALSE FALSE FALSE FALSE FALSE FALSE FALSE FALSE FALSE FALSE FALSE FALSE
## 157 158 159
## FALSE FALSE FALSE

new.df.cov4$large.residual <- new.df.cov4$standardized.residuals > 2 | new.df.cov4$standardized.residuals < -2
sum(new.df.cov4$large.residual)

## [1] 8

new.df.cov4[new.df.cov4$large.residual, c("X1", "standardized.residuals")]

## # A tibble: 8 x 2
## X1 standardized.residuals
## <dbl> <dbl>
## 1 4 2.21
## 2 73 2.12
## 3 101 -2.20
## 4 104 -2.47
## 5 105 2.64
## 6 116 2.40
## 7 137 -2.65
## 8 154 3.95

new.df.cov4$cooks.distance <- cooks.distance(model.y4.cov)
new.df.cov4$leverage <- hatvalues(model.y4.cov)
new.df.cov4$covariance <- covratio(model.y4.cov)

new.df.cov4[new.df.cov4$large.residual, c("X1", "cooks.distance", "leverage", "covariance" )]

## # A tibble: 8 x 4
## X1 cooks.distance leverage covariance
## <dbl> <dbl> <dbl> <dbl>
## 1 4 0.0162 0.0288 0.811
## 2 73 0.0231 0.0441 0.844
## 3 101 0.0266 0.0471 0.830
## 4 104 0.0326 0.0458 0.766
## 5 105 0.0275 0.0342 0.716
## 6 116 0.0521 0.0754 0.808
## 7 137 0.0513 0.0618 0.736
## 8 154 0.118 0.0636 0.422

Remove outlier and re build models

new.df.cov4 %>%
 filter (! (X1 == 154)) -> new.df.cov4

model.m4.cov <- lm(Loneliness ~ Resta + gender + age + ethnicity.dicotomised +
 sexuality.dicotomised + relationship.dicotomised +
 employment.dicotomised, data = new.df.cov4)

model.y4.cov <- lm(CAPE_dep ~ Resta + Loneliness+ gender + age + ethnicity.dicotomised + sexuality.dicotomised + relationship.dicotomised +
 employment.dicotomised, data = new.df.cov4)

Re-check assumptions

new.df.cov4$standardized.residuals <- rstandard(model.y4.cov)
plot(model.y4.cov)


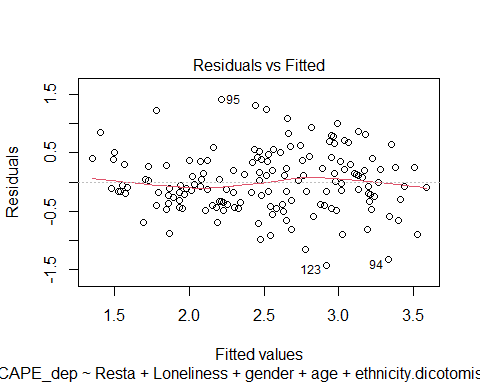

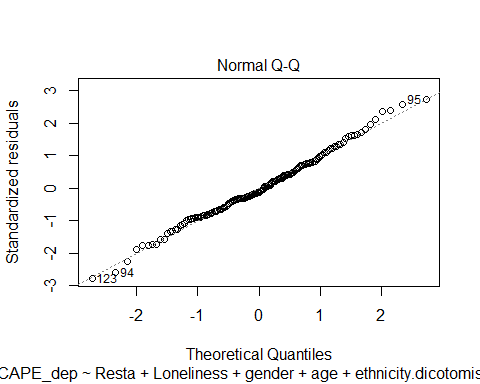

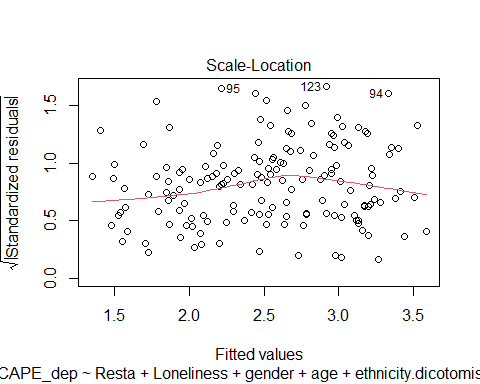

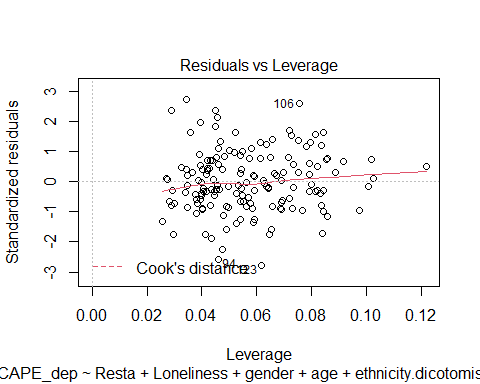


hist(new.df.cov4$standardized.residuals)


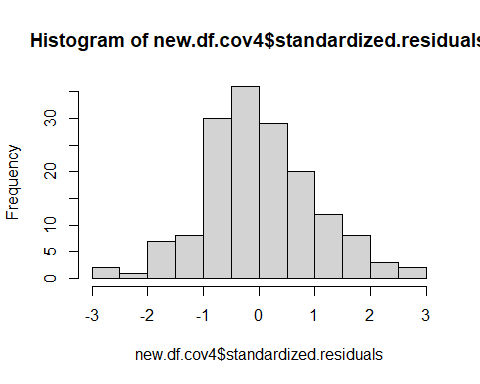


shapiro.test(new.df.cov4$standardized.residuals)

##
## Shapiro-Wilk normality test
##
## data: new.df.cov4$standardized.residuals
## W = 0.99237, p-value = 0.5674

Run mediation analysis

med.cov4 <- mediate(model.m4.cov, model.y4.cov, sims = 5000, treat = "Resta", mediator = "Loneliness")

summary(med.cov4)

##
## Causal Mediation Analysis
##
## Quasi-Bayesian Confidence Intervals
##
## Estimate 95% CI Lower 95% CI Upper p-value
## ACME -0.0618 -0.0837 -0.04 <2e-16 ***
## ADE -0.0145 -0.0415 0.01 0.29
## Total Effect -0.0763 -0.1018 -0.05 <2e-16 ***
## Prop. Mediated 0.8099 0.5435 1.19 <2e-16 ***
## ---
## Signif. codes: 0 '***' 0.001 '**' 0.01 '*' 0.05 '.' 0.1 ' ' 1
##
## Sample Size Used: 158
##
##
## Simulations: 5000
