# Supplementary material for: Being more satisfied with romantic relationship status is associated with increased mental wellbeing in people with experience of psychosis
Source: Front Psychiatry. 2023 Sep 28;14:1232973. doi: 10.3389/fpsyt.2023.1232973 (PMC10569177; doi:10.3389/fpsyt.2023.1232973)
Supplement: Supplementary file 10 [file Data_Sheet_10.DOCX]

Hypothesis 3, part 2

Rebecca White

08/07/2022

Investigating part 2 of hypothesis 3 - that greater satisfaction with current romantic relationship status (ReSta) will be increased wellbeing i.e. reduced psychotic experiences. Specifically, reduced scores on CAPE depressive subscale (CAPE positive and negative subscales were not significantly correlated with ReSta and so are not being tested)

Install packages and import the dataset

library(tidyverse)

## -- Attaching packages --------------------------------------- tidyverse 1.3.0 --

## v ggplot2 3.3.2 v purrr 0.3.4
## v tibble 3.0.4 v dplyr 1.0.2
## v tidyr 1.1.2 v stringr 1.4.0
## v readr 1.4.0 v forcats 0.5.0

## -- Conflicts ------------------------------------------ tidyverse_conflicts() --
## x dplyr::filter() masks stats::filter()
## x dplyr::lag() masks stats::lag()

library(ggplot2)
library(Hmisc)

## Loading required package: lattice

## Loading required package: survival

## Loading required package: Formula

##
## Attaching package: 'Hmisc'

## The following objects are masked from 'package:dplyr':
##
## src, summarize

## The following objects are masked from 'package:base':
##
## format.pval, units

library(MASS)

##
## Attaching package: 'MASS'

## The following object is masked from 'package:dplyr':
##
## select

library(car)

## Loading required package: carData

##
## Attaching package: 'car'

## The following object is masked from 'package:dplyr':
##
## recode

## The following object is masked from 'package:purrr':
##
## some

library(olsrr)

##
## Attaching package: 'olsrr'

## The following object is masked from 'package:MASS':
##
## cement

## The following object is masked from 'package:datasets':
##
## rivers

library(QuantPsyc)

## Loading required package: boot

##
## Attaching package: 'boot'

## The following object is masked from 'package:car':
##
## logit

## The following object is masked from 'package:survival':
##
## aml

## The following object is masked from 'package:lattice':
##
## melanoma

##
## Attaching package: 'QuantPsyc'

## The following object is masked from 'package:base':
##
## norm

library(pastecs)

##
## Attaching package: 'pastecs'

## The following objects are masked from 'package:dplyr':
##
## first, last

## The following object is masked from 'package:tidyr':
##
## extract

library(dplyr)
library(readr)

Dataset_Missing_Removed <- read_csv("Z:/Online study IRAS ID 271957/Online analysis/Dataset_190_obs_2.9.21.csv")

## Warning: Missing column names filled in: 'X1' [1]

##
## -- Column specification --------------------------------------------------------
## cols(
## .default = col_double(),
## redcap_survey_identifier = col_logical(),
## pis_timestamp = col_datetime(format = ""),
## screening_questions_timestamp = col_datetime(format = ""),
## demographic_information_timestamp = col_datetime(format = ""),
## nationality = col_character(),
## ethnicity_other = col_character(),
## gender_self_describe = col_character(),
## sexual_orientation_selfdescribe = col_character(),
## rr_selfdescribe = col_character(),
## last_rr_end = col_character(),
## current_rr_length = col_character(),
## the_community_assessment_of_psychic_experiences_ca_timestamp = col_datetime(format = ""),
## the_short_warwick_mental_health_wellbeing_scale_timestamp = col_datetime(format = ""),
## adapted_satisfaction_with_relationships_scale_rest_timestamp = col_datetime(format = ""),
## three_item_loneliness_scale_timestamp = col_datetime(format = ""),
## internalised_stigma_of_mental_illness_inventory_10_timestamp = col_datetime(format = ""),
## multidimensional_scale_of_perceived_social_support_timestamp = col_datetime(format = ""),
## self_esteem_rating_scale_short_form_serssf_timestamp = col_datetime(format = ""),
## relationships_questionnaire_timestamp = col_datetime(format = ""),
## Screening_Qs_result = col_character()
## # ... with 7 more columns
## )
## i Use `spec()` for the full column specifications.

Build data frame to work from

data.frame2 <- data.frame(Dataset_Missing_Removed$Resta.total,
 Dataset_Missing_Removed$GenderF,
 Dataset_Missing_Removed$age,
 Dataset_Missing_Removed$EthnicityF,
 Dataset_Missing_Removed$ethnicity_other,
 Dataset_Missing_Removed$SexualityF,
 Dataset_Missing_Removed$sexual_orientation_selfdescribe,
 Dataset_Missing_Removed$R_Status_simplified,
 Dataset_Missing_Removed$rr_selfdescribe,
 Dataset_Missing_Removed$CAPE_depressive,
 Dataset_Missing_Removed$EmploymentF)

#rename columns
names(data.frame2)[names(data.frame2) == "Dataset_Missing_Removed.CAPE_depressive"]<- "CAPE.depressive"
names(data.frame2)[names(data.frame2) == "Dataset_Missing_Removed.Resta.total"]<- "Resta"
names(data.frame2)[names(data.frame2) == "Dataset_Missing_Removed.GenderF"]<- "gender"
names(data.frame2)[names(data.frame2) == "Dataset_Missing_Removed.age"]<- "age"
names(data.frame2)[names(data.frame2) == "Dataset_Missing_Removed.EthnicityF"]<- "ethnicity"
names(data.frame2)[names(data.frame2) == "Dataset_Missing_Removed.SexualityF"]<- "sexuality"
names(data.frame2)[names(data.frame2) == "Dataset_Missing_Removed.R_Status_simplified"]<- "relationship.status"
names(data.frame2)[names(data.frame2) == "Dataset_Missing_Removed.EmploymentF"]<- "employment"

#Create dichotomous variables
#GENDER
#Gender is categorised as 1= female, 2 = male, 3 = prefer not to say, 4 = prefer to self describe
table(data.frame2$gender)

##
## female male Self_describe
## 105 69 6

#remove 'prefer not to say & self describe
data.frame2$gender <- factor(data.frame2$gender,
 c("female","male"), c("female", "male"))
table(data.frame2$gender)

##
## female male
## 105 69

#ETHNICITY
table(data.frame2$ethnicity)

##
## asian black mixed other white
## 10 11 5 5 148

data.frame2 %>%
 mutate(ethnicity.dicotomised = case_when(ethnicity == "other" & Dataset_Missing_Removed.ethnicity_other == "White Scottish" ~ "white",
 ethnicity == "white" ~ "white",
 ethnicity == "mixed" ~ "PGM",
 ethnicity == "asian" ~ "PGM",
 ethnicity == "black" ~ "PGM",
 ethnicity == "chinese" ~ "PGM",
 ethnicity == "other" ~ "PGM")) -> data.frame2

table(data.frame2$ethnicity.dicotomised)

##
## PGM white
## 29 150

#SEXUALITY
table(data.frame2$sexuality)

##
## bisexual gay/lesbian heterosexual prefer not to say
## 24 9 132 6
## self-describe
## 9

#checks done where participants have chosen to self-describe their sexuality & that happy to put all in LGBQ+ group

data.frame2 %>%
 mutate(sexuality.dicotomised = case_when(sexuality == "heterosexual" ~ "heterosexual",
 sexuality == "bisexual" ~ "LGBQ+",
 sexuality == "gay/lesbian" ~ "LGBQ+",
 sexuality == "self-describe" ~ "LGBQ+")) -> data.frame2
table(data.frame2$sexuality.dicotomised)

##
## heterosexual LGBQ+
## 132 42

#RELATIONSHIP STATUS
table(data.frame2$relationship.status)

##
## dating partner self describe separated single
## 5 78 5 2 89

data.frame2 %>%
 mutate(relationship.dicotomised = case_when(relationship.status == "single" ~ "single",
 relationship.status == "dating" ~ "single",
 relationship.status == "separated" ~ "single",
 relationship.status == "widowed" ~ "single",
 relationship.status == "partner" ~ "partner",
 relationship.status == "self describe" & Dataset_Missing_Removed.rr_selfdescribe == "Living with a wife, queerplatonic partner, and steady, with one long-distance relationship as well" ~ "partner",
 relationship.status == "self describe" ~ "single")) -> data.frame2

table(data.frame2$relationship.dicotomised)

##
## partner single
## 79 100

#EMPLOYMENT
table(data.frame2$employment)

##
## employee FT education
## 56 21
## looking after home/family rec. sickness/disability benefits
## 6 58
## retired self-employed
## 5 5
## unemployed
## 26

data.frame2 %>%
 mutate(employment.dicotomised = case_when ( employment == "employee" ~ "working/FT.education",
 employment == "self-employed" ~ "working/FT.education",
 employment == "FT education" ~ "working/FT.education",
 employment == "unemployed" ~ "unemployed",
 employment == "looking after home/family" ~ "working/FT.education",
 employment == "rec. sickness/disability benefits" ~ "unemployed",
 employment == "retired" ~ "unemployed")) -> data.frame2

table(data.frame2$employment.dicotomised)

##
## unemployed working/FT.education
## 89 88

#remove columns that are mainly NA e.g. rr_self describe so they don't interfere with next stages
drops2 <- c("Dataset_Missing_Removed.rr_selfdescribe", "Dataset_Missing_Removed.ethnicity_other",
 "Dataset_Missing_Removed.sexual_orientation_selfdescribe")
data.frame2[ , !(names(data.frame2) %in% drops2)] -> data.frame2

#identify missing data
apply(data.frame2, 1, function(X) sum(is.na(X)))

## [1] 0 0 0 0 0 0 1 0 3 0 0 0 0 0 0 0 0 0 0 0 0 0 0 0 0
## [26] 0 0 0 0 0 0 0 0 0 0 0 0 1 0 0 0 0 0 0 0 0 0 0 1 0
## [51] 0 0 0 0 0 0 0 0 1 0 0 0 0 0 0 0 0 0 1 0 0 0 0 2 0
## [76] 0 0 0 0 0 0 10 0 0 0 0 0 0 0 0 0 0 0 0 0 0 0 0 0 0
## [101] 0 0 0 0 0 0 0 0 0 0 0 0 0 0 0 0 0 0 0 0 0 0 0 0 0
## [126] 1 0 3 0 0 0 0 0 0 0 0 0 0 1 1 2 0 0 0 0 0 0 0 0 0
## [151] 0 0 0 0 0 0 1 0 0 1 0 0 0 0 0 0 0 0 0 0 0 0 0 0 10
## [176] 10 10 0 0 0 0 0 10 10 10 10 10 10 0 3

data.frame2$nmiss <- apply(data.frame2, 1, function(X) sum(is.na(X)))
table(data.frame2$nmiss)

##
## 0 1 2 3 10
## 165 10 2 3 10

#remove rows with missing data
data.frame2 <- na.omit(data.frame2)
#check rows with missing data have been removed
table(data.frame2$nmiss)

##
## 0
## 165

# need to create dummy variables / convert new dictomised variables
 # to factors
names <- c('ethnicity.dicotomised', 'sexuality.dicotomised', 'relationship.dicotomised', 'employment.dicotomised')
data.frame2[,names]<- lapply(data.frame2[,names],factor)
str(data.frame2)

## 'data.frame': 165 obs. of 13 variables:
## $ Resta : num 15 14 15 13 10 10 13 14 7 14 ...
## $ gender : Factor w/ 2 levels "female","male": 1 1 1 1 1 2 1 1 2 1 ...
## $ age : num 32 22 31 27 45 50 32 39 62 54 ...
## $ ethnicity : chr "white" "white" "mixed" "white" ...
## $ sexuality : chr "heterosexual" "bisexual" "heterosexual" "heterosexual" ...
## $ relationship.status : chr "partner" "partner" "partner" "partner" ...
## $ CAPE.depressive : num 1.88 3.38 1.88 3.75 2.5 ...
## $ employment : chr "FT education" "employee" "employee" "FT education" ...
## $ ethnicity.dicotomised : Factor w/ 2 levels "PGM","white": 2 2 1 2 2 1 2 2 2 2 ...
## $ sexuality.dicotomised : Factor w/ 2 levels "heterosexual",..: 1 2 1 1 1 1 1 1 1 1 ...
## $ relationship.dicotomised: Factor w/ 2 levels "partner","single": 1 1 1 1 1 1 1 1 2 1 ...
## $ employment.dicotomised : Factor w/ 2 levels "unemployed","working/FT.education": 2 2 2 2 2 2 1 2 1 1 ...
## $ nmiss : int 0 0 0 0 0 0 0 0 0 0 ...
## - attr(*, "na.action")= 'omit' Named int [1:25] 7 9 38 49 59 69 74 82 126 128 ...
## ..- attr(*, "names")= chr [1:25] "7" "9" "38" "49" ...

#gender
Female0_v_Male1 <- c(0, 1)
contrasts(data.frame2$gender) <- cbind(Female0_v_Male1)

#ethnicity
White0_v_PGM1 <- c(1, 0)
contrasts(data.frame2$ethnicity.dicotomised) <- cbind(White0_v_PGM1)

#sexuality
heter0_v_LGBQ1 <- c(0, 1)
contrasts(data.frame2$sexuality.dicotomised) <- cbind(heter0_v_LGBQ1)

#relationship status
single0_v_partner1 <- c(1,0)
contrasts(data.frame2$relationship.dicotomised) <- cbind(single0_v_partner1)

#employment status
unemploy.0_v_work1 <- c(0,1)
contrasts(data.frame2$employment.dicotomised) <- cbind(unemploy.0_v_work1)

## convert dichotomous variables into numeric values -this will allow for beta scores to be generated

#gender
data.frame2%>%
 mutate (gender.num = case_when ( gender == "male" ~ 1,
 gender == "female" ~ 0)) -> data.frame2

#relationship status
data.frame2 %>%
 mutate (rel.num = case_when (relationship.dicotomised == "single" ~ 0,
 relationship.dicotomised == "partner" ~ 1)) -> data.frame2

#ethnicity
data.frame2 %>%
 mutate (ethnicity.num = case_when ( ethnicity.dicotomised == "white" ~ 0,
 ethnicity.dicotomised == "PGM" ~ 1)) -> data.frame2

#sexuality
data.frame2 %>%
 mutate (sex.num = case_when ( sexuality.dicotomised == "heterosexual" ~ 0,
 sexuality.dicotomised == "LGBQ+" ~ 1)) -> data.frame2

#employment
data.frame2 %>%
 mutate (employ.num = case_when ( employment.dicotomised == "unemployed" ~ 0,
 employment.dicotomised == "working/FT.education" ~ 1)) -> data.frame2

Build models

mod1 <- lm(CAPE.depressive ~ Resta, data = data.frame2)

summary(mod1)

##
## Call:
## lm(formula = CAPE.depressive ~ Resta, data = data.frame2)
##
## Residuals:
## Min 1Q Median 3Q Max
## -1.41903 -0.59871 -0.02562 0.60049 1.65129
##
## Coefficients:
## Estimate Std. Error t value Pr(>|t|)
## (Intercept) 2.87610 0.13015 22.099 < 2e-16 ***
## Resta -0.03516 0.01210 -2.906 0.00417 **
## ---
## Signif. codes: 0 '***' 0.001 '**' 0.01 '*' 0.05 '.' 0.1 ' ' 1
##
## Residual standard error: 0.7371 on 163 degrees of freedom
## Multiple R-squared: 0.04925, Adjusted R-squared: 0.04342
## F-statistic: 8.444 on 1 and 163 DF, p-value: 0.004171

lm.beta(mod1)

## Resta
## -0.2219333

mod2<- lm(CAPE.depressive ~ Resta + gender.num + age + ethnicity.num +
 sex.num + rel.num + employ.num, data = data.frame2)

summary(mod2)

##
## Call:
## lm(formula = CAPE.depressive ~ Resta + gender.num + age + ethnicity.num +
## sex.num + rel.num + employ.num, data = data.frame2)
##
## Residuals:
## Min 1Q Median 3Q Max
## -1.30472 -0.42805 -0.06878 0.42975 1.65627
##
## Coefficients:
## Estimate Std. Error t value Pr(>|t|)
## (Intercept) 3.697205 0.235688 15.687 < 2e-16 ***
## Resta -0.065912 0.012592 -5.234 5.24e-07 ***
## gender.num -0.445948 0.106102 -4.203 4.41e-05 ***
## age -0.009600 0.004474 -2.146 0.033442 *
## ethnicity.num -0.179892 0.137359 -1.310 0.192229
## sex.num 0.415046 0.132890 3.123 0.002131 **
## rel.num 0.442490 0.124663 3.550 0.000509 ***
## employ.num -0.484061 0.112045 -4.320 2.75e-05 ***
## ---
## Signif. codes: 0 '***' 0.001 '**' 0.01 '*' 0.05 '.' 0.1 ' ' 1
##
## Residual standard error: 0.6384 on 157 degrees of freedom
## Multiple R-squared: 0.3131, Adjusted R-squared: 0.2824
## F-statistic: 10.22 on 7 and 157 DF, p-value: 1.609e-10

lm.beta(mod2)

## Resta gender.num age ethnicity.num sex.num
## -0.41604933 -0.29076812 -0.15977461 -0.08857412 0.22814697
## rel.num employ.num
## 0.29250407 -0.32212092

anova(mod1, mod2)

## Analysis of Variance Table
##
## Model 1: CAPE.depressive ~ Resta
## Model 2: CAPE.depressive ~ Resta + gender.num + age + ethnicity.num +
## sex.num + rel.num + employ.num
## Res.Df RSS Df Sum of Sq F Pr(>F)
## 1 163 88.559
## 2 157 63.986 6 24.573 10.049 2.187e-09 ***
## ---
## Signif. codes: 0 '***' 0.001 '**' 0.01 '*' 0.05 '.' 0.1 ' ' 1

Anova suggest that mod2 accounts for significantly more of the variance in CAPE depressive scores than mod1

Test for outliers and influential cases

data.frame2$residuals.mod2 <- resid(mod2)
data.frame2$standardized.residuals.mod2 <- rstandard(mod2)
data.frame2$studentized.residuals.mod2 <- rstudent(mod2)
data.frame2$cooks.distance.mod2 <- cooks.distance(mod2)
data.frame2$dfbeta.mod2 <- dfbeta(mod2)
data.frame2$dffit.mod2 <- dffits(mod2)
data.frame2$leverage.mod2 <- hatvalues(mod2)
data.frame2$covariance.mod2 <- covratio(mod2)

data.frame2$standardized.residuals.mod2 > 2 | data.frame2$standardized.residuals.mod2< -2

## [1] FALSE FALSE FALSE FALSE FALSE FALSE FALSE FALSE FALSE FALSE FALSE FALSE
## [13] FALSE FALSE FALSE FALSE FALSE FALSE FALSE FALSE FALSE FALSE FALSE FALSE
## [25] FALSE FALSE FALSE FALSE FALSE FALSE FALSE FALSE FALSE FALSE FALSE FALSE
## [37] FALSE FALSE FALSE FALSE FALSE FALSE FALSE FALSE FALSE FALSE FALSE FALSE
## [49] FALSE FALSE FALSE FALSE FALSE FALSE FALSE FALSE FALSE FALSE FALSE FALSE
## [61] FALSE FALSE FALSE FALSE FALSE FALSE FALSE FALSE FALSE FALSE FALSE FALSE
## [73] FALSE FALSE FALSE FALSE FALSE FALSE FALSE FALSE FALSE FALSE FALSE FALSE
## [85] FALSE FALSE FALSE FALSE FALSE FALSE FALSE FALSE FALSE FALSE FALSE FALSE
## [97] TRUE FALSE FALSE FALSE FALSE FALSE FALSE FALSE FALSE FALSE FALSE FALSE
## [109] FALSE FALSE FALSE FALSE FALSE FALSE FALSE FALSE FALSE FALSE FALSE FALSE
## [121] FALSE FALSE FALSE FALSE FALSE FALSE FALSE FALSE FALSE FALSE FALSE FALSE
## [133] FALSE FALSE FALSE FALSE FALSE FALSE FALSE FALSE TRUE TRUE FALSE FALSE
## [145] FALSE FALSE FALSE FALSE TRUE FALSE FALSE FALSE FALSE FALSE FALSE FALSE
## [157] FALSE FALSE FALSE FALSE FALSE FALSE FALSE FALSE FALSE

# would expect 95% to be within this range (156 / 165)

data.frame2$large.residual.mod2 <- data.frame2$standardized.residuals.mod2 > 2 | data.frame2$standardized.residuals.mod2 < -2
sum(data.frame2$large.residual.mod2) # 4 cases have a large residual

## [1] 4

data.frame2[data.frame2$large.residual.mod2, c( "standardized.residuals.mod2" )]

## [1] 2.339279 2.658895 -2.070751 2.075219

# one has standardised residual over +/-2.5,
#none have a standardised residual larger than +/- 3

#look at leverage and cooks distance for these 4 cases

data.frame2[data.frame2$large.residual.mod2, c("cooks.distance.mod2", "leverage.mod2", "covariance.mod2" )]

## cooks.distance.mod2 leverage.mod2 covariance.mod2
## 97 0.02384034 0.03367905 0.8200112
## 141 0.04447136 0.04791210 0.7646118
## 142 0.01426835 0.02592974 0.8657609
## 149 0.02436265 0.04329758 0.8806230

#none have cooks distance greater than 1 so none are having an undue influence on the model

#all have leverage below 2 x (0.097) and 3x (0.145) (formula = k +1 /n = 8/165 =0.048)

#check covariance
# should be between 1+[3(k+1)/n] and 1- [3(k+1)/n] so in this case 0.855 - 1.145 (b/c k = 7)
# two cases covariance outside this, however given cooks distance this is probably not something to worry about (Field, p.291)
# so conclude - no outliers

#check assumption of independence
dwt(mod2)

## lag Autocorrelation D-W Statistic p-value
## 1 -0.0477494 2.090861 0.51
## Alternative hypothesis: rho != 0

# assumption met

#assumption of no multicollinearity
vif(mod2) #VIF

## Resta gender.num age ethnicity.num sex.num
## 1.443914 1.093841 1.267365 1.045420 1.219573
## rel.num employ.num
## 1.552078 1.270592

1/vif(mod2) #tolerance

## Resta gender.num age ethnicity.num sex.num
## 0.6925619 0.9142097 0.7890387 0.9565532 0.8199589
## rel.num employ.num
## 0.6442976 0.7870349

mean(vif(mod2)) #mean VIF

## [1] 1.270398

#check assumptions about the residuals
plot(mod2)


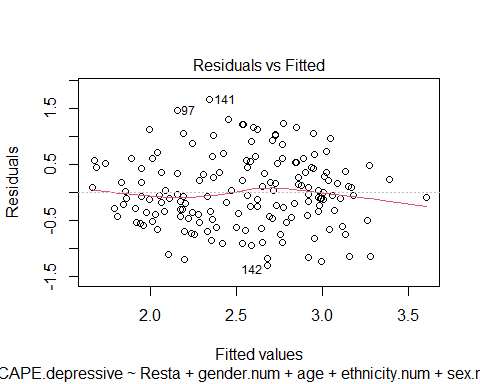

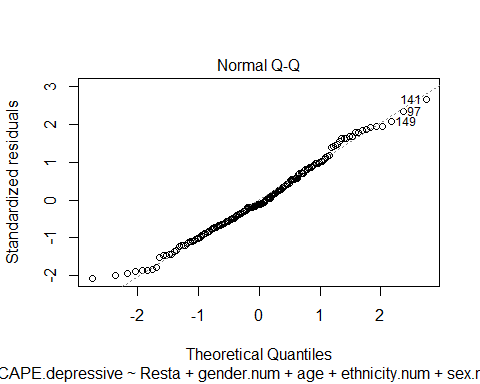

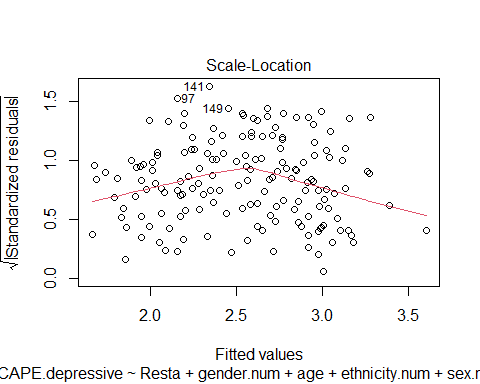

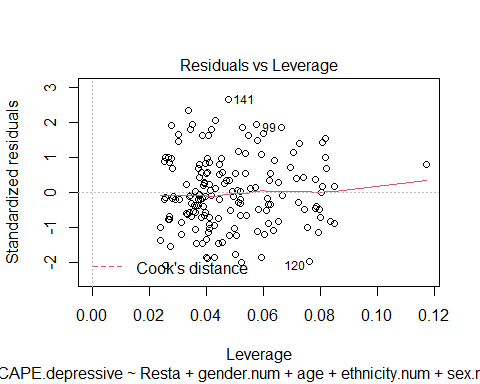


hist(data.frame2$standardized.residuals.mod2)


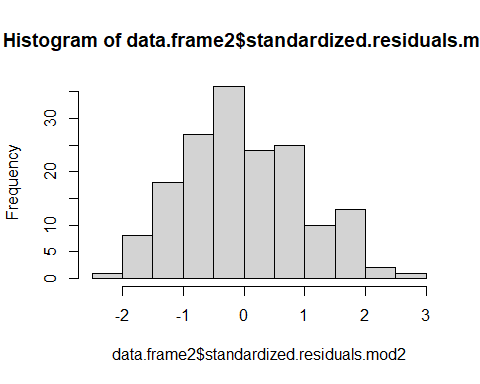


shapiro.test(data.frame2$standardized.residuals.mod2)

##
## Shapiro-Wilk normality test
##
## data: data.frame2$standardized.residuals.mod2
## W = 0.98755, p-value = 0.1511

Assumptions met, no outliers

Further analysis to check for a suppressor variable given the unexpected direction of association between having a partner and mental wellbeing in the regression model

#install necessary packages
library(pastecs)
library(foreign)
library(effsize)

## Warning: package 'effsize' was built under R version 4.0.5

First look at association between relationship status satisfaction (ReSta) and relationship status

by(data.frame2$Resta, data.frame2$relationship.dicotomised, stat.desc,
 basic = FALSE, norm = TRUE)

## data.frame2$relationship.dicotomised: partner
## median mean SE.mean CI.mean.0.95 var
## 1.400000e+01 1.247945e+01 3.576249e-01 7.129122e-01 9.336377e+00
## std.dev coef.var skewness skew.2SE kurtosis
## 3.055549e+00 2.448464e-01 -1.592266e+00 -2.832919e+00 2.574080e+00
## kurt.2SE normtest.W normtest.p
## 2.318060e+00 7.948125e-01 1.056445e-08
## ------------------------------------------------------------
## data.frame2$relationship.dicotomised: single
## median mean SE.mean CI.mean.0.95 var
## 7.0000000000 7.4130434783 0.4880688698 0.9694888528 21.9154323937
## std.dev coef.var skewness skew.2SE kurtosis
## 4.6813921427 0.6315074445 0.0523708764 0.1041823484 -1.2488429308
## kurt.2SE normtest.W normtest.p
## -1.2545871214 0.9425567573 0.0005114647

t.test1 <- t.test(Resta ~ relationship.dicotomised, data = data.frame2,
 paired = FALSE)
t.test1

##
## Welch Two Sample t-test
##
## data: Resta by relationship.dicotomised
## t = 8.3733, df = 157.55, p-value = 2.874e-14
## alternative hypothesis: true difference in means is not equal to 0
## 95 percent confidence interval:
## 3.871318 6.261499
## sample estimates:
## mean in group partner mean in group single
## 12.479452 7.413043

#SD
binVar <- data.frame2$relationship.dicotomised
scaleVar <- data.frame2$Resta

partner.sd <- aggregate(scaleVar~binVar, FUN =sd)[1,2]
single.sd <- aggregate(scaleVar~binVar, FUN = sd)[2,2]

partner.sd

## [1] 3.055549

single.sd

## [1] 4.681392

##cohen's d
cohen.d(scaleVar ~ binVar)

##
## Cohen's d
##
## d estimate: 1.252626 (large)
## 95 percent confidence interval:
## lower upper
## 0.914492 1.590759

Suggests that participants who had a partner were significantly more satisfied with their relationship status that participants who did not have a partner.

Visualise data using violin plot:

data.frame2 %>%
 group_by(relationship.dicotomised) %>%
 summarise(median = median(Resta))

## `summarise()` ungrouping output (override with `.groups` argument)

## # A tibble: 2 x 2
## relationship.dicotomised median
## <fct> <dbl>
## 1 partner 14
## 2 single 7

data.frame2 %>%
 group_by(relationship.dicotomised) %>%
 ggplot(aes(x= fct_reorder(relationship.dicotomised, Resta, median), y = Resta,colour = relationship.dicotomised)) +
 geom_violin() +
 geom_boxplot(alpha =.5) +
 geom_jitter(alpha =.2, width = .1) +
 guides(colour = FALSE) +
 labs (x = "\nRelationship status", y= "ReSta score\n") +
 theme(axis.text=element_text(size = 14))+
 theme(axis.title = element_text(size = 14))


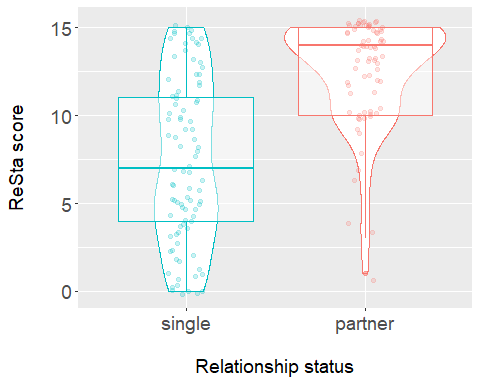


Correlation between ReSta and CAPE depressive scores

cor.test(data.frame2$Resta, data.frame2$CAPE.depressive,
 method = "spearman")

## Warning in cor.test.default(data.frame2$Resta, data.frame2$CAPE.depressive, :
## Cannot compute exact p-value with ties

##
## Spearman's rank correlation rho
##
## data: data.frame2$Resta and data.frame2$CAPE.depressive
## S = 914693, p-value = 0.004199
## alternative hypothesis: true rho is not equal to 0
## sample estimates:
## rho
## -0.2217733

Satisfaction with current relationship status is significantly negatively correlated with CAPE depressive scores

Finally, look at the associate between CAPE depressive subscale scores and relationship status

by(data.frame2$CAPE.depressive, data.frame2$relationship.dicotomised, stat.desc,
 basic = FALSE, norm = TRUE)

## data.frame2$relationship.dicotomised: partner
## median mean SE.mean CI.mean.0.95 var std.dev
## 2.50000000 2.56727006 0.08550551 0.17045212 0.53371705 0.73055941
## coef.var skewness skew.2SE kurtosis kurt.2SE normtest.W
## 0.28456664 -0.03325468 -0.05916588 -1.00758050 -0.90736600 0.97176050
## normtest.p
## 0.10036248
## ------------------------------------------------------------
## data.frame2$relationship.dicotomised: single
## median mean SE.mean CI.mean.0.95 var std.dev
## 2.464285714 2.512357660 0.080755034 0.160409954 0.599966542 0.774575072
## coef.var skewness skew.2SE kurtosis kurt.2SE normtest.W
## 0.308306052 0.268250092 0.533634846 -1.089760391 -1.094772864 0.952787930
## normtest.p
## 0.002179058

t.test2 <- t.test(CAPE.depressive ~ relationship.dicotomised,
 data = data.frame2,
 paired = FALSE)

t.test2

##
## Welch Two Sample t-test
##
## data: CAPE.depressive by relationship.dicotomised
## t = 0.46689, df = 158.16, p-value = 0.6412
## alternative hypothesis: true difference in means is not equal to 0
## 95 percent confidence interval:
## -0.1773801 0.2872049
## sample estimates:
## mean in group partner mean in group single
## 2.567270 2.512358

#SD
binVar2 <- data.frame2$relationship.dicotomised
scaleVar2 <- data.frame2$CAPE.depressive

partner.sd2 <- aggregate(scaleVar2~binVar2, FUN =sd)[1,2]
single.sd2 <- aggregate(scaleVar2~binVar2, FUN = sd)[2,2]

partner.sd2

## [1] 0.7305594

single.sd2

## [1] 0.7745751

# effect size cohens d
cohen.d(scaleVar2~binVar2)

##
## Cohen's d
##
## d estimate: 0.07268844 (negligible)
## 95 percent confidence interval:
## lower upper
## -0.2369199 0.3822968

Results of t-test suggest significant association between CAPE depressive scores and relationship status

Overall, the analysis suggests that as in the previous model, relationship status was a suppressor variable.

Finally, save data frame

write.csv(data.frame2, file ="Z:/Online study IRAS ID 271957/Online analysis/H3_table3.7_data.frame2.csv", row.names =TRUE)
