# Supplementary material for: Being more satisfied with romantic relationship status is associated with increased mental wellbeing in people with experience of psychosis
Source: Front Psychiatry. 2023 Sep 28;14:1232973. doi: 10.3389/fpsyt.2023.1232973 (PMC10569177; doi:10.3389/fpsyt.2023.1232973)
Supplement: Supplementary file 11 [file Data_Sheet_11.DOCX]

Hypothesis 3, part 1

Rebecca White

07/07/2022

Investigating part 1 of hypothesis 3 - that greater satisfaction with current romantic relationship status will be associated with increased mental wellbeing as measured by SWEMWBS

(regression models are being run for SWEMWBS and CAPE depressive subscale only as the CAPE positive and negative subscales were not significantly correlated with ReSta)

Install relevant packages and import the dataset

library(tidyverse)

## -- Attaching packages --------------------------------------- tidyverse 1.3.0 --

## v ggplot2 3.3.2 v purrr 0.3.4
## v tibble 3.0.4 v dplyr 1.0.2
## v tidyr 1.1.2 v stringr 1.4.0
## v readr 1.4.0 v forcats 0.5.0

## -- Conflicts ------------------------------------------ tidyverse_conflicts() --
## x dplyr::filter() masks stats::filter()
## x dplyr::lag() masks stats::lag()

library(ggplot2)
library(Hmisc)

## Loading required package: lattice

## Loading required package: survival

## Loading required package: Formula

##
## Attaching package: 'Hmisc'

## The following objects are masked from 'package:dplyr':
##
## src, summarize

## The following objects are masked from 'package:base':
##
## format.pval, units

library(MASS)

##
## Attaching package: 'MASS'

## The following object is masked from 'package:dplyr':
##
## select

library(car)

## Loading required package: carData

##
## Attaching package: 'car'

## The following object is masked from 'package:dplyr':
##
## recode

## The following object is masked from 'package:purrr':
##
## some

library(olsrr)

##
## Attaching package: 'olsrr'

## The following object is masked from 'package:MASS':
##
## cement

## The following object is masked from 'package:datasets':
##
## rivers

library(QuantPsyc)

## Loading required package: boot

##
## Attaching package: 'boot'

## The following object is masked from 'package:car':
##
## logit

## The following object is masked from 'package:survival':
##
## aml

## The following object is masked from 'package:lattice':
##
## melanoma

##
## Attaching package: 'QuantPsyc'

## The following object is masked from 'package:base':
##
## norm

library(pastecs)

##
## Attaching package: 'pastecs'

## The following objects are masked from 'package:dplyr':
##
## first, last

## The following object is masked from 'package:tidyr':
##
## extract

library(dplyr)

library(readr)
Dataset_Missing_Removed<- read_csv("Z:/Online study IRAS ID 271957/Online analysis/Dataset_190_obs_2.9.21.csv")

## Warning: Missing column names filled in: 'X1' [1]

##
## -- Column specification --------------------------------------------------------
## cols(
## .default = col_double(),
## redcap_survey_identifier = col_logical(),
## pis_timestamp = col_datetime(format = ""),
## screening_questions_timestamp = col_datetime(format = ""),
## demographic_information_timestamp = col_datetime(format = ""),
## nationality = col_character(),
## ethnicity_other = col_character(),
## gender_self_describe = col_character(),
## sexual_orientation_selfdescribe = col_character(),
## rr_selfdescribe = col_character(),
## last_rr_end = col_character(),
## current_rr_length = col_character(),
## the_community_assessment_of_psychic_experiences_ca_timestamp = col_datetime(format = ""),
## the_short_warwick_mental_health_wellbeing_scale_timestamp = col_datetime(format = ""),
## adapted_satisfaction_with_relationships_scale_rest_timestamp = col_datetime(format = ""),
## three_item_loneliness_scale_timestamp = col_datetime(format = ""),
## internalised_stigma_of_mental_illness_inventory_10_timestamp = col_datetime(format = ""),
## multidimensional_scale_of_perceived_social_support_timestamp = col_datetime(format = ""),
## self_esteem_rating_scale_short_form_serssf_timestamp = col_datetime(format = ""),
## relationships_questionnaire_timestamp = col_datetime(format = ""),
## Screening_Qs_result = col_character()
## # ... with 7 more columns
## )
## i Use `spec()` for the full column specifications.

Build data frame to work from

data.frame1 <- data.frame(Dataset_Missing_Removed$Resta.total,
 Dataset_Missing_Removed$GenderF,
 Dataset_Missing_Removed$age,
 Dataset_Missing_Removed$EthnicityF,
 Dataset_Missing_Removed$SexualityF,
 Dataset_Missing_Removed$R_Status_simplified,
 Dataset_Missing_Removed$rr_selfdescribe,
 Dataset_Missing_Removed$SWEMWBS_metric,
 Dataset_Missing_Removed$EmploymentF,
 Dataset_Missing_Removed$ethnicity_other)

#Rename columns
names(data.frame1)[names(data.frame1) == "Dataset_Missing_Removed.SWEMWBS_metric"]<- "SWEMWBS"
names(data.frame1)[names(data.frame1) == "Dataset_Missing_Removed.Resta.total"]<- "Resta"
names(data.frame1)[names(data.frame1) == "Dataset_Missing_Removed.GenderF"]<- "gender"
names(data.frame1)[names(data.frame1) == "Dataset_Missing_Removed.age"]<- "age"
names(data.frame1)[names(data.frame1) == "Dataset_Missing_Removed.EthnicityF"]<- "ethnicity"
names(data.frame1)[names(data.frame1) == "Dataset_Missing_Removed.SexualityF"]<- "sexuality"
names(data.frame1)[names(data.frame1) == "Dataset_Missing_Removed.R_Status_simplified"]<- "relationship.status"
names(data.frame1)[names(data.frame1) == "Dataset_Missing_Removed.EmploymentF"]<- "employment"

#Create dichotomous variables
#GENDER
#Gender is categorised as 1= female, 2 = male, 3 = prefer not to say, 4 = prefer to self describe
table(data.frame1$gender)

##
## female male Self_describe
## 105 69 6

#remove 'prefer not to say' & 'self describe'
data.frame1$gender <- factor(data.frame1$gender,
 c("female","male"), c("female", "male"))
table(data.frame1$gender)

##
## female male
## 105 69

#ETHNICITY
table(data.frame1$ethnicity)

##
## asian black mixed other white
## 10 11 5 5 148

Dataset_Missing_Removed$ethnicity_other

## [1] NA NA NA NA
## [5] NA NA NA NA
## [9] NA NA NA NA
## [13] NA NA NA NA
## [17] NA "White Scottish" "White Scottish" NA
## [21] NA NA NA NA
## [25] NA NA NA NA
## [29] NA NA NA NA
## [33] NA NA NA NA
## [37] NA NA NA NA
## [41] NA NA NA NA
## [45] NA NA NA NA
## [49] NA NA NA NA
## [53] NA NA NA NA
## [57] NA NA NA NA
## [61] NA NA NA "Roma"
## [65] NA NA NA NA
## [69] NA NA NA NA
## [73] NA NA NA NA
## [77] NA NA "Brazilian" NA
## [81] NA NA NA NA
## [85] NA NA NA NA
## [89] NA NA NA NA
## [93] NA NA NA NA
## [97] NA NA NA NA
## [101] NA NA NA NA
## [105] NA NA NA NA
## [109] NA NA NA NA
## [113] NA NA NA NA
## [117] NA NA NA NA
## [121] NA NA NA NA
## [125] NA NA NA NA
## [129] NA NA NA NA
## [133] NA NA NA NA
## [137] NA NA NA NA
## [141] NA NA NA NA
## [145] NA NA NA NA
## [149] NA NA NA NA
## [153] NA NA NA NA
## [157] NA NA NA NA
## [161] NA NA NA NA
## [165] NA NA NA NA
## [169] NA NA NA NA
## [173] NA "Greek Orthodox" NA NA
## [177] NA NA NA NA
## [181] NA NA NA NA
## [185] NA NA NA NA
## [189] NA NA

data.frame1 %>%
 mutate(ethnicity.dicotomised = case_when(ethnicity == "other" & Dataset_Missing_Removed.ethnicity_other == "White Scottish" ~ "white",
 ethnicity == "white" ~ "white",
 ethnicity == "mixed" ~ "PGM",
 ethnicity == "asian" ~ "PGM",
 ethnicity == "black" ~ "PGM",
 ethnicity == "chinese" ~ "PGM",
 ethnicity == "other" ~ "PGM")) -> data.frame1

table(data.frame1$ethnicity.dicotomised)

##
## PGM white
## 29 150

#SEXUALITY
table(data.frame1$sexuality)

##
## bisexual gay/lesbian heterosexual prefer not to say
## 24 9 132 6
## self-describe
## 9

#checks done where participants have chosen to self-describe their sexuality & that happy to put all in LGBQ+ group. One participant's answer is ambiguous however this will be removed from analysis as their gender was not within the male or female dichotomy

data.frame1 %>%
 mutate(sexuality.dicotomised = case_when(sexuality == "heterosexual" ~ "heterosexual",
 sexuality == "bisexual" ~ "LGBQ+",
 sexuality == "gay/lesbian" ~ "LGBQ+",
 sexuality == "self-describe" ~ "LGBQ+")) -> data.frame1
table(data.frame1$sexuality.dicotomised)

##
## heterosexual LGBQ+
## 132 42

#RELATIONSHIP STATUS, single vs partner
table(data.frame1$relationship.status)

##
## dating partner self describe separated single
## 5 78 5 2 89

data.frame1 %>%
 mutate(relationship.dicotomised = case_when(relationship.status == "single" ~ "single",
 relationship.status == "dating" ~ "single",
 relationship.status == "separated" ~ "single",
 relationship.status == "widowed" ~ "single",
 relationship.status == "partner" ~ "partner",
 relationship.status == "self describe" & Dataset_Missing_Removed.rr_selfdescribe == "Living with a wife, queerplatonic partner, and steady, with one long-distance relationship as well" ~ "partner",
 relationship.status == "self describe" ~ "single")) -> data.frame1

table(data.frame1$relationship.dicotomised)

##
## partner single
## 79 100

#EMPLOYMENT
table(data.frame1$employment)

##
## employee FT education
## 56 21
## looking after home/family rec. sickness/disability benefits
## 6 58
## retired self-employed
## 5 5
## unemployed
## 26

data.frame1 %>%
 mutate(employment.dicotomised = case_when ( employment == "employee" ~ "working/FT.education",
 employment == "self-employed" ~ "working/FT.education",
 employment == "FT education" ~ "working/FT.education",
 employment == "unemployed" ~ "unemployed",
 employment == "looking after home/family" ~ "working/FT.education",
 employment == "rec. sickness/disability benefits" ~ "unemployed",
 employment == "retired" ~ "unemployed")) -> data.frame1

table(data.frame1$employment.dicotomised)

##
## unemployed working/FT.education
## 89 88

#remove columns that are mainly NA e.g. rr_self describe so they don't interfere with next stages
drops <- c("Dataset_Missing_Removed.rr_selfdescribe", "Dataset_Missing_Removed.ethnicity_other")
data.frame1[ , !(names(data.frame1) %in% drops)] -> data.frame1

#identify missing data
apply(data.frame1, 1, function(X) sum(is.na(X)))

## [1] 0 0 0 0 0 0 1 0 3 0 0 0 0 0 0 0 0 0 0 0 0 0 0 0 0
## [26] 0 0 0 0 0 0 0 0 0 0 0 0 1 0 0 0 0 0 0 0 0 0 0 1 0
## [51] 0 0 0 0 0 0 0 0 1 0 0 0 0 0 0 0 0 0 1 0 0 0 0 2 0
## [76] 0 1 0 0 0 0 10 0 0 0 0 0 0 0 0 0 0 0 0 0 0 0 0 0 0
## [101] 0 0 0 0 0 0 0 0 0 0 0 0 0 0 0 0 0 0 0 0 0 0 0 0 0
## [126] 1 0 3 0 0 0 0 0 0 0 0 0 0 1 1 2 0 0 0 0 0 0 0 0 0
## [151] 0 0 0 0 0 0 1 0 0 1 0 0 0 0 0 0 0 0 0 0 0 0 0 0 10
## [176] 10 10 0 0 0 0 0 10 10 10 10 10 10 0 3

data.frame1$nmiss <- apply(data.frame1, 1, function(X) sum(is.na(X)))
table(data.frame1$nmiss)

##
## 0 1 2 3 10
## 164 11 2 3 10

#remove rows with missing data to allow for anova
data.frame1 <- na.omit(data.frame1)
table(data.frame1$nmiss)

##
## 0
## 164

#Create dummy variables/convert variables to factors
names <- c('ethnicity.dicotomised', 'sexuality.dicotomised', 'relationship.dicotomised', 'employment.dicotomised')
data.frame1[,names]<- lapply(data.frame1[,names],factor)
str(data.frame1)

## 'data.frame': 164 obs. of 13 variables:
## $ Resta : num 15 14 15 13 10 10 13 14 7 14 ...
## $ gender : Factor w/ 2 levels "female","male": 1 1 1 1 1 2 1 1 2 1 ...
## $ age : num 32 22 31 27 45 50 32 39 62 54 ...
## $ ethnicity : chr "white" "white" "mixed" "white" ...
## $ sexuality : chr "heterosexual" "bisexual" "heterosexual" "heterosexual" ...
## $ relationship.status : chr "partner" "partner" "partner" "partner" ...
## $ SWEMWBS : num 19.2 16.9 25 14.1 19.2 ...
## $ employment : chr "FT education" "employee" "employee" "FT education" ...
## $ ethnicity.dicotomised : Factor w/ 2 levels "PGM","white": 2 2 1 2 2 1 2 2 2 2 ...
## $ sexuality.dicotomised : Factor w/ 2 levels "heterosexual",..: 1 2 1 1 1 1 1 1 1 1 ...
## $ relationship.dicotomised: Factor w/ 2 levels "partner","single": 1 1 1 1 1 1 1 1 2 1 ...
## $ employment.dicotomised : Factor w/ 2 levels "unemployed","working/FT.education": 2 2 2 2 2 2 1 2 1 1 ...
## $ nmiss : int 0 0 0 0 0 0 0 0 0 0 ...
## - attr(*, "na.action")= 'omit' Named int [1:26] 7 9 38 49 59 69 74 77 82 126 ...
## ..- attr(*, "names")= chr [1:26] "7" "9" "38" "49" ...

#gender
Female0_v_Male1 <- c(0, 1)
contrasts(data.frame1$gender) <- cbind(Female0_v_Male1)

#ethnicity
White0_v_PGM1 <- c(1, 0)
contrasts(data.frame1$ethnicity.dicotomised) <- cbind(White0_v_PGM1)

#sexuality
heter0_v_LGBQ1 <- c(0, 1)
contrasts(data.frame1$sexuality.dicotomised) <- cbind(heter0_v_LGBQ1)

#relationship status
single0_v_partner1 <- c(1,0)
contrasts(data.frame1$relationship.dicotomised) <- cbind(single0_v_partner1)

#employment status
unemploy.0_v_work1 <- c(0,1)
contrasts(data.frame1$employment.dicotomised) <- cbind(unemploy.0_v_work1)

## convert dichotomous variables into numeric values to allow for beta scores to be generated

#gender
data.frame1 %>%
 mutate (gender.num = case_when ( gender == "male" ~ 1,
 gender == "female" ~ 0)) -> data.frame1

#relationship status
data.frame1 %>%
 mutate (rel.num = case_when (relationship.dicotomised == "single" ~ 0,
 relationship.dicotomised == "partner" ~ 1)) -> data.frame1

#ethnicity
data.frame1 %>%
 mutate (ethnicity.num = case_when ( ethnicity.dicotomised == "white" ~ 0,
 ethnicity.dicotomised == "PGM" ~ 1)) -> data.frame1

#sexuality
data.frame1 %>%
 mutate (sex.num = case_when ( sexuality.dicotomised == "heterosexual" ~ 0,
 sexuality.dicotomised == "LGBQ+" ~ 1)) -> data.frame1

#employment
data.frame1 %>%
 mutate (employ.num = case_when ( employment.dicotomised == "unemployed" ~ 0,
 employment.dicotomised == "working/FT.education" ~ 1)) -> data.frame1

Build models

mod1 <- lm(SWEMWBS ~ Resta, data = data.frame1, na.action = na.exclude)
mod2 <- lm(SWEMWBS ~ Resta + gender + age + ethnicity.dicotomised + sexuality.dicotomised + relationship.dicotomised + employment.dicotomised , data = data.frame1, na.action = na.exclude)

summary(mod1)

##
## Call:
## lm(formula = SWEMWBS ~ Resta, data = data.frame1, na.action = na.exclude)
##
## Residuals:
## Min 1Q Median 3Q Max
## -13.2179 -2.7879 -0.6924 3.0293 13.7926
##
## Coefficients:
## Estimate Std. Error t value Pr(>|t|)
## (Intercept) 16.12843 0.79516 20.283 < 2e-16 ***
## Resta 0.29211 0.07403 3.946 0.000118 ***
## ---
## Signif. codes: 0 '***' 0.001 '**' 0.01 '*' 0.05 '.' 0.1 ' ' 1
##
## Residual standard error: 4.503 on 162 degrees of freedom
## Multiple R-squared: 0.08768, Adjusted R-squared: 0.08205
## F-statistic: 15.57 on 1 and 162 DF, p-value: 0.0001182

summary(mod2)

##
## Call:
## lm(formula = SWEMWBS ~ Resta + gender + age + ethnicity.dicotomised +
## sexuality.dicotomised + relationship.dicotomised + employment.dicotomised,
## data = data.frame1, na.action = na.exclude)
##
## Residuals:
## Min 1Q Median 3Q Max
## -13.6221 -2.6868 -0.2536 2.5525 16.5578
##
## Coefficients:
## Estimate Std. Error t value
## (Intercept) 14.1927319 1.5406855 9.212
## Resta 0.4399982 0.0826326 5.325
## genderFemale0_v_Male1 1.8070109 0.6945856 2.602
## age 0.0007371 0.0293601 0.025
## ethnicity.dicotomisedWhite0_v_PGM1 -0.1227145 0.8970168 -0.137
## sexuality.dicotomisedheter0_v_LGBQ1 -2.5377262 0.8673115 -2.926
## relationship.dicotomisedsingle0_v_partner1 -2.5577637 0.8183136 -3.126
## employment.dicotomisedunemploy.0_v_work1 2.9084586 0.7315686 3.976
## Pr(>|t|)
## (Intercept) < 2e-16 ***
## Resta 3.47e-07 ***
## genderFemale0_v_Male1 0.010173 *
## age 0.980003
## ethnicity.dicotomisedWhite0_v_PGM1 0.891363
## sexuality.dicotomisedheter0_v_LGBQ1 0.003947 **
## relationship.dicotomisedsingle0_v_partner1 0.002116 **
## employment.dicotomisedunemploy.0_v_work1 0.000107 ***
## ---
## Signif. codes: 0 '***' 0.001 '**' 0.01 '*' 0.05 '.' 0.1 ' ' 1
##
## Residual standard error: 4.166 on 156 degrees of freedom
## Multiple R-squared: 0.2481, Adjusted R-squared: 0.2143
## F-statistic: 7.353 on 7 and 156 DF, p-value: 1.266e-07

Run anova to check whether mod2 is a significantly better predictor of mental wellbeing than mod1

anova(mod1, mod2)

## Analysis of Variance Table
##
## Model 1: SWEMWBS ~ Resta
## Model 2: SWEMWBS ~ Resta + gender + age + ethnicity.dicotomised + sexuality.dicotomised +
## relationship.dicotomised + employment.dicotomised
## Res.Df RSS Df Sum of Sq F Pr(>F)
## 1 162 3284.8
## 2 156 2707.3 6 577.53 5.5464 3.097e-05 ***
## ---
## Signif. codes: 0 '***' 0.001 '**' 0.01 '*' 0.05 '.' 0.1 ' ' 1

Test for outliers and influential cases

data.frame1$residuals <- resid(mod2)
data.frame1$standardized.residuals <- rstandard(mod2)
data.frame1$studentized.residuals <- rstudent(mod2)
data.frame1$cooks.distance <- cooks.distance(mod2)
data.frame1$dfbeta <- dfbeta(mod2)
data.frame1$dffit <- dffits(mod2)
data.frame1$leverage <- hatvalues(mod2)
data.frame1$covariance <- covratio(mod2)

data.frame1$standardized.residuals > 2 | data.frame1$standardized.residuals < -2

## [1] FALSE FALSE FALSE FALSE FALSE FALSE FALSE FALSE FALSE FALSE FALSE FALSE
## [13] FALSE FALSE FALSE FALSE FALSE FALSE FALSE FALSE FALSE FALSE FALSE FALSE
## [25] FALSE FALSE FALSE FALSE FALSE FALSE FALSE FALSE FALSE FALSE FALSE FALSE
## [37] FALSE FALSE FALSE FALSE TRUE FALSE FALSE FALSE FALSE FALSE FALSE FALSE
## [49] FALSE FALSE FALSE FALSE FALSE FALSE TRUE FALSE FALSE FALSE TRUE FALSE
## [61] FALSE FALSE FALSE FALSE FALSE FALSE FALSE FALSE FALSE FALSE FALSE FALSE
## [73] FALSE FALSE FALSE FALSE FALSE FALSE FALSE FALSE FALSE FALSE FALSE FALSE
## [85] FALSE FALSE FALSE FALSE FALSE FALSE FALSE FALSE FALSE FALSE FALSE FALSE
## [97] FALSE TRUE FALSE FALSE FALSE FALSE FALSE FALSE FALSE FALSE FALSE FALSE
## [109] FALSE FALSE FALSE FALSE FALSE FALSE FALSE FALSE FALSE FALSE FALSE FALSE
## [121] FALSE FALSE FALSE FALSE TRUE FALSE FALSE FALSE FALSE FALSE FALSE FALSE
## [133] FALSE FALSE FALSE FALSE FALSE FALSE FALSE FALSE FALSE FALSE FALSE FALSE
## [145] FALSE FALSE FALSE FALSE FALSE FALSE FALSE FALSE FALSE FALSE FALSE FALSE
## [157] FALSE FALSE FALSE FALSE FALSE FALSE FALSE TRUE

# would expect 95% to be within this range (so approx 156/164)

data.frame1$large.residual <- data.frame1$standardized.residuals > 2 | data.frame1$standardized.residuals < -2
sum(data.frame1$large.residual) # 6 cases have a large residual

## [1] 6

data.frame1[data.frame1$large.residual, c( "standardized.residuals" )]

## [1] 2.049242 4.074017 -3.410850 -2.093569 2.209585 -2.429692

# none have standardised residual +/- 2.5,
# two cases have a standardised residual larger than +/- 3

#look at leverage, cooks distance and covariance for these cases
data.frame1[data.frame1$large.residual, c("cooks.distance", "leverage", "covariance" )]

## cooks.distance leverage covariance
## 41 0.03380932 0.06051065 0.9008295
## 55 0.10505763 0.04819690 0.4497394
## 59 0.12804370 0.08092351 0.6161929
## 98 0.03908909 0.06659489 0.8979635
## 125 0.02746448 0.04306482 0.8530754
## 164 0.03016762 0.03927601 0.8048493

#none have cooks distance greater than one so none are having an undue influence on the model
#none have leverage that is problematic (k +1 /n = 8/164 = 0.049 (x2 = 0.098, x 3 = 0.147))
#check covariance 1 + /- [3(k+1) / n] -> 0.85 - 1.15
#3 potential outliers have CVR values outside this range

#check assumption of independence
dwt(mod2) # assumption met

## lag Autocorrelation D-W Statistic p-value
## 1 0.04582434 1.870638 0.402
## Alternative hypothesis: rho != 0

#assumption of no multicollinearity
vif(mod2) #VIF

## Resta gender age
## 1.455792 1.096393 1.260451
## ethnicity.dicotomised sexuality.dicotomised relationship.dicotomised
## 1.045756 1.217888 1.562962
## employment.dicotomised
## 1.264206

1/vif(mod2) #tolerance

## Resta gender age
## 0.6869114 0.9120817 0.7933668
## ethnicity.dicotomised sexuality.dicotomised relationship.dicotomised
## 0.9562464 0.8210936 0.6398107
## employment.dicotomised
## 0.7910102

mean(vif(mod2)) #mean VIF shouldn't be 'substantially greater than 1'

## [1] 1.271921

#check assumptions about the residuals
plot(mod2)


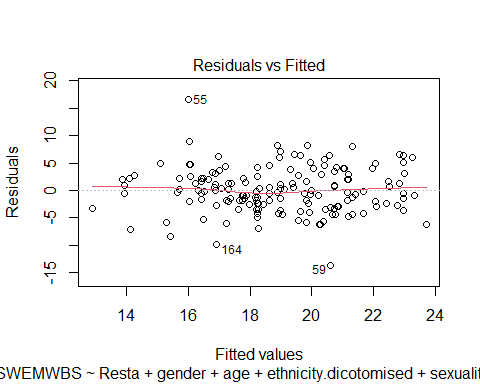

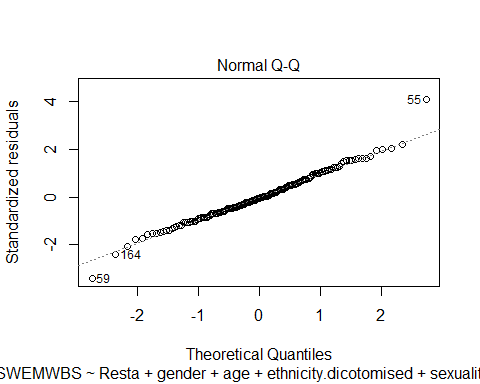

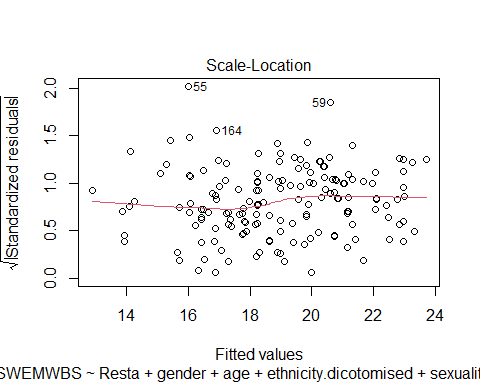

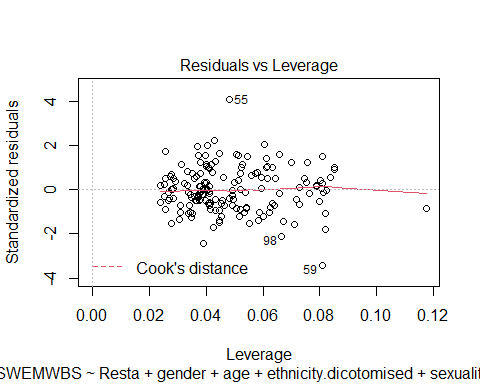


hist(data.frame1$standardized.residuals)


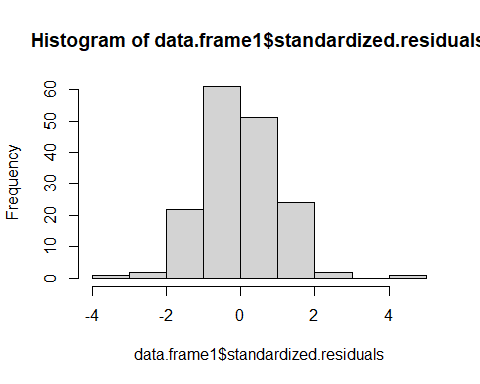


shapiro.test(data.frame1$standardized.residuals)

##
## Shapiro-Wilk normality test
##
## data: data.frame1$standardized.residuals
## W = 0.98484, p-value = 0.07121

Remove the two cases that have a standardised residual +/-3 and create a new data frame without them

test3 <- data.frame1
threshold1 <- 3
threshold2 <- -3

test3 <- subset(test3, test3$standardized.residuals < threshold1)
test3 <- subset(test3, test3$standardized.residuals > threshold2)

data.frame1_OR <- test3

Re-run the regression models without outliers using the new dataframe. Use numeric versions of sociodemographic variables so that beta values can also be calculated

mod1num <- lm(SWEMWBS ~ Resta, data = data.frame1_OR)
summary(mod1num)

##
## Call:
## lm(formula = SWEMWBS ~ Resta, data = data.frame1_OR)
##
## Residuals:
## Min 1Q Median 3Q Max
## -10.784 -2.800 -0.713 2.884 9.838
##
## Coefficients:
## Estimate Std. Error t value Pr(>|t|)
## (Intercept) 15.9514 0.7553 21.120 < 2e-16 ***
## Resta 0.3102 0.0704 4.406 1.92e-05 ***
## ---
## Signif. codes: 0 '***' 0.001 '**' 0.01 '*' 0.05 '.' 0.1 ' ' 1
##
## Residual standard error: 4.271 on 160 degrees of freedom
## Multiple R-squared: 0.1082, Adjusted R-squared: 0.1026
## F-statistic: 19.41 on 1 and 160 DF, p-value: 1.924e-05

lm.beta(mod1num)

## Resta
## 0.3289132

mod2num <- lm (SWEMWBS ~ Resta + gender.num + age + ethnicity.num + sex.num + rel.num +
 employ.num, data = data.frame1_OR)
summary(mod2num)

##
## Call:
## lm(formula = SWEMWBS ~ Resta + gender.num + age + ethnicity.num +
## sex.num + rel.num + employ.num, data = data.frame1_OR)
##
## Residuals:
## Min 1Q Median 3Q Max
## -9.5991 -2.7421 -0.2474 2.5878 9.3722
##
## Coefficients:
## Estimate Std. Error t value Pr(>|t|)
## (Intercept) 13.913007 1.411277 9.858 < 2e-16 ***
## Resta 0.499392 0.076105 6.562 7.65e-10 ***
## gender.num 1.840590 0.634429 2.901 0.004263 **
## age -0.002738 0.026867 -0.102 0.918975
## ethnicity.num 0.423111 0.831201 0.509 0.611455
## sex.num -2.716144 0.807729 -3.363 0.000974 ***
## rel.num -3.217586 0.755496 -4.259 3.56e-05 ***
## employ.num 3.008393 0.670167 4.489 1.39e-05 ***
## ---
## Signif. codes: 0 '***' 0.001 '**' 0.01 '*' 0.05 '.' 0.1 ' ' 1
##
## Residual standard error: 3.801 on 154 degrees of freedom
## Multiple R-squared: 0.3201, Adjusted R-squared: 0.2892
## F-statistic: 10.36 on 7 and 154 DF, p-value: 1.283e-10

lm.beta(mod2num)

## Resta gender.num age ethnicity.num sex.num
## 0.529589501 0.201210896 -0.007612168 0.034553958 -0.246084473
## rel.num employ.num
## -0.355717009 0.334662145

anova(mod1num, mod2num)

## Analysis of Variance Table
##
## Model 1: SWEMWBS ~ Resta
## Model 2: SWEMWBS ~ Resta + gender.num + age + ethnicity.num + sex.num +
## rel.num + employ.num
## Res.Df RSS Df Sum of Sq F Pr(>F)
## 1 160 2918.7
## 2 154 2225.0 6 693.69 8.0022 1.589e-07 ***
## ---
## Signif. codes: 0 '***' 0.001 '**' 0.01 '*' 0.05 '.' 0.1 ' ' 1

The direction of the assoication between having a partner and mental wellbeing was unexpected. Further analysis was conducted to check for a suppressor variable.

#install necessary packages
library(pastecs)
library(foreign)
library(effsize)

## Warning: package 'effsize' was built under R version 4.0.5

First, look at association between relationship status satisfaction (ReSta) and relationship status

by(data.frame1_OR$Resta, data.frame1_OR$relationship.dicotomised, stat.desc,
 basic = FALSE, norm = TRUE)

## data.frame1_OR$relationship.dicotomised: partner
## median mean SE.mean CI.mean.0.95 var
## 1.400000e+01 1.252778e+01 3.593009e-01 7.164256e-01 9.294992e+00
## std.dev coef.var skewness skew.2SE kurtosis
## 3.048769e+00 2.433607e-01 -1.651426e+00 -2.918765e+00 2.781593e+00
## kurt.2SE normtest.W normtest.p
## 2.488759e+00 7.858712e-01 7.284691e-09
## ------------------------------------------------------------
## data.frame1_OR$relationship.dicotomised: single
## median mean SE.mean CI.mean.0.95 var
## 7.0000000000 7.2777777778 0.4893440737 0.9723162511 21.5511860175
## std.dev coef.var skewness skew.2SE kurtosis
## 4.6423254967 0.6378767858 0.0902560595 0.1776470486 -1.2117701301
## kurt.2SE normtest.W normtest.p
## -1.2046963775 0.9450291290 0.0008362931

t.test1 <- t.test(Resta ~ relationship.dicotomised, data = data.frame1_OR,
 paired = FALSE)
t.test1

##
## Welch Two Sample t-test
##
## data: Resta by relationship.dicotomised
## t = 8.6479, df = 154.53, p-value = 6.305e-15
## alternative hypothesis: true difference in means is not equal to 0
## 95 percent confidence interval:
## 4.050739 6.449261
## sample estimates:
## mean in group partner mean in group single
## 12.527778 7.277778

table(data.frame1_OR$relationship.dicotomised)

##
## partner single
## 72 90

#SD
binVar <- data.frame1_OR$relationship.dicotomised
scaleVar <- data.frame1_OR$Resta

partner.sd <- aggregate(scaleVar~binVar, FUN =sd)[1,2]
single.sd <- aggregate(scaleVar~binVar, FUN = sd)[2,2]

partner.sd

## [1] 3.048769

single.sd

## [1] 4.642325

#Cohen's D
cohen.d(scaleVar~binVar)

##
## Cohen's d
##
## d estimate: 1.30791 (large)
## 95 percent confidence interval:
## lower upper
## 0.964256 1.651564

Suggests that participants who had a partner were significantly more satisfied with their relationship status that participants who did not have a partner.

Visualise data using violin plot:

data.frame1_OR %>%
 group_by(relationship.dicotomised) %>%
 summarise(median = median(Resta))

## `summarise()` ungrouping output (override with `.groups` argument)

## # A tibble: 2 x 2
## relationship.dicotomised median
## <fct> <dbl>
## 1 partner 14
## 2 single 7

data.frame1_OR %>%
 group_by(relationship.dicotomised) %>%
 ggplot(aes(x= fct_reorder(relationship.dicotomised, Resta, median), y = Resta,colour = relationship.dicotomised)) +
 geom_violin() +
 geom_boxplot(alpha =.5) +
 geom_jitter(alpha =.2, width = .1) +
 guides(colour = FALSE) +
 labs (x = "\nRelationship status", y= "ReSta score\n") +
 theme(axis.text=element_text(size = 14))+
 theme(axis.title = element_text(size = 14))


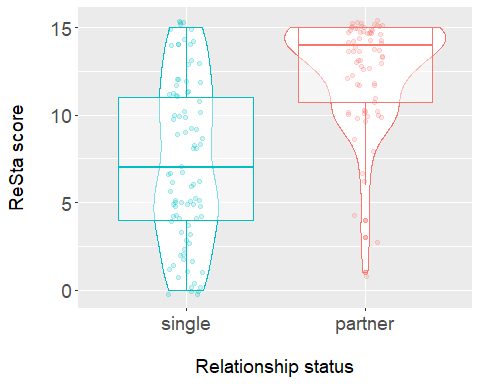


Look at correlation between ReSta and SWEMWBS

cor.test(data.frame1_OR$Resta, data.frame1_OR$SWEMWBS,
 method = "spearman")

## Warning in cor.test.default(data.frame1_OR$Resta, data.frame1_OR$SWEMWBS, :
## Cannot compute exact p-value with ties

##
## Spearman's rank correlation rho
##
## data: data.frame1_OR$Resta and data.frame1_OR$SWEMWBS
## S = 490393, p-value = 6.723e-05
## alternative hypothesis: true rho is not equal to 0
## sample estimates:
## rho
## 0.3079023

Relationship status satisfaction and mental wellbeing scores are significantly positively correlated

Look at association between SWEMWBS and relationship status

by(data.frame1_OR$SWEMWBS, data.frame1_OR$relationship.dicotomised, stat.desc,
 basic = FALSE, norm = TRUE)

## data.frame1_OR$relationship.dicotomised: partner
## median mean SE.mean CI.mean.0.95 var std.dev
## 18.5900000 18.9500000 0.4888927 0.9748243 17.2091549 4.1483918
## coef.var skewness skew.2SE kurtosis kurt.2SE normtest.W
## 0.2189125 0.1869716 0.3304576 -0.2640605 -0.2362614 0.9821489
## normtest.p
## 0.3993507
## ------------------------------------------------------------
## data.frame1_OR$relationship.dicotomised: single
## median mean SE.mean CI.mean.0.95 var std.dev
## 17.98000000 18.91822222 0.50599977 1.00541077 23.04321928 4.80033533
## coef.var skewness skew.2SE kurtosis kurt.2SE normtest.W
## 0.25374135 0.11421196 0.22479840 0.07246817 0.07204513 0.96367686
## normtest.p
## 0.01290420

t.test2 <- t.test(SWEMWBS ~ relationship.dicotomised, data = data.frame1_OR,
 paired = FALSE)

t.test2

##
## Welch Two Sample t-test
##
## data: SWEMWBS by relationship.dicotomised
## t = 0.045165, df = 159.02, p-value = 0.964
## alternative hypothesis: true difference in means is not equal to 0
## 95 percent confidence interval:
## -1.357827 1.421382
## sample estimates:
## mean in group partner mean in group single
## 18.95000 18.91822

#SD
binVar2 <- data.frame1_OR$relationship.dicotomised
scaleVar2 <- data.frame1_OR$SWEMWBS

partner.sd2 <- aggregate(scaleVar2~binVar2, FUN =sd)[1,2]
single.sd2 <- aggregate(scaleVar2~binVar2, FUN = sd)[2,2]

partner.sd2

## [1] 4.148392

single.sd2

## [1] 4.800335

#Cohens d effect size
cohen.d(scaleVar2~ binVar2)

##
## Cohen's d
##
## d estimate: 0.007026364 (negligible)
## 95 percent confidence interval:
## lower upper
## -0.3052339 0.3192867

Suggests no significant difference in mental wellbeing scores between those who have a partner and those who do not have a partner

Overall, results of these tests suggest that relationship status was a suppressor variable and only appeared to be significantly negatively associated with mental wellbeing (SWEMWBS) because of the effect of relationship status satisfaction (ReSta)on mental wellbeing was being held constant.

Finally, save dataframe

write.csv(data.frame1_OR, file = "Z:/Online study IRAS ID 271957/Online analysis/H3_table3.6_data.frame1_OR.csv",
 row.names = T)
